# Views on democracy and political violence in the United States in 2025: findings from a nationally representative survey

**DOI:** 10.1186/s40621-026-00684-3

**Published:** 2026-05-27

**Authors:** Garen J. Wintemute, Sonia L. Robinson, Andrew Crawford, Julia P. Schleimer, Daniel J. Tancredi, Aaron B. Shev, Elizabeth A. Tomsich, Mona A. Wright, Bradley Velasquez, Shaina Sta. Cruz, Veronica A. Pear

**Affiliations:** 1 Department of Emergency Medicine, School of Medicine, UC Davis Medical Center, 2315 Stockton Blvd, Sacramento, CA 95817 USA; 2 Centers for Violence Prevention, UC Davis, Sacramento, CA USA; 3 Department of Pediatrics, School of Medicine, UC Davis, Sacramento, CA USA

**Keywords:** Political violence, Firearm violence, Violence and society, Domestic violent extremism, Civil war, Political parties, Republican party, Democratic party, Make America Great Again (MAGA) movement, Longitudinal survey

## Abstract

**Background:**

From 2022 to 2024, an annual, nationally representative, longitudinal survey in the United States (US) has found concerning prevalences of support for and willingness to engage in political violence. This study examines changes in those prevalences from mid-2024 to mid-2025.

**Methods:**

Survey participants were members of Ipsos Knowledge Panel, ≥ 18 years old as of recruitment in 2022. Wave 4 was conducted May 23–June 13, 2025. The primary analysis generated findings for the entire cohort; a secondary analysis categorized respondents by political party and Make America Great Again (MAGA) movement affiliations. Principal outcome measures comprised measures of justification for, willingness to engage in, and expectation of firearm use in political violence. Results for 2025 were presented as weighted prevalences, with comparisons based on adjusted prevalence differences. Change from 2024 to 2025 was estimated based on mean change scores.

**Results:**

The 2025 completion rate was 89.9% (8,248 respondents/9,179 invitees). For the cohort as a whole, there were only small increases from 2024 to 2025 in the prevalence of the belief that violence was usually or always justified to advance at least 1 of 20 political objectives (2024: 32.3%, 95% confidence interval (CI) 31.0%, 33.6%; 2025: 35.6%, 95% CI 34.1%, 37.0%) and to advance 16 of 20 individual objectives. There were few changes in willingness to commit political violence and none in expectation of firearm use. Despite some increases among Democrats, MAGA Republicans in 2025 were substantially more likely than strong Democrats to consider violence usually or always justified to advance at least 1 of 20 political objectives (MAGA Republicans 52.2%, 95% CI 48.4%, 56.1%; strong Democrats 32.1%, 95% CI 28.6%, 35.6%) and to advance 10 of those objectives when objectives were considered individually. A small group of non-Republican MAGA supporters had higher prevalences than most other groups on many measures.

**Conclusions:**

Support for and willingness to commit political violence increased only modestly from 2024 to 2025 and, where differences existed, remained generally higher among MAGA movement affiliates and Republicans than among Democrats. These findings can help advance prevention efforts, which are urgently needed in this US election year.

**Supplementary Information:**

The online version contains supplementary material available at 10.1186/s40621-026-00684-3.

## Background

The possibility of widespread political violence in the United States (US) is a subject of increasing concern among experts and the public [1–6]. Both concern and risk are likely to increase in 2026, as important mid-term elections approach and as the US celebrates the 250th anniversary of the protracted period of large-scale political violence—the American Revolution—that established the country. A recurrence of large-scale violence would constitute a significant public health problem in itself, and the social instability it reflected and created would have even greater effects.

Since 2022, we have conducted an annual, nationally representative, longitudinal survey on attitudes toward democracy and authoritarianism and support for and willingness to engage in political violence in the US [7, 8]. In 2024, 26.2% of respondents considered violence usually or always justifiable to advance at least 1 of 17 specified political objectives [8]; 6.5% strongly or very strongly agreed that there would be civil war in the next few years; and 3.6% strongly or very strongly agreed that such a war was needed “to set things right” [9].

The survey has also identified a wide array of respondent characteristics that are associated with increased support for and willingness to engage in political violence. Among others, these have included younger age and male sex [7, 8]; racism, xenophobia, and other forms of fear and hatred of others [10]; some aspects of firearm ownership and use, particularly assault-type rifle ownership and habitual carrying of firearms in public [11]; and affiliation with right-wing extremist political and religious organizations and movements [12]. Similar associations have been found by others [13–16].

Political ideology and party affiliation were strongly associated with political violence in the 2022—2024 waves of the survey, with prevalences higher among Republicans than among Democrats, higher among “Make America Great Again” (MAGA) Republicans than all others, and higher among conservatives (particularly among extreme conservatives) than among liberals [7, 17–19]. Again, others have obtained similar results [13, 20–27]. A Democratic party administration was in power during those years, and approximately 5% of our survey respondents each year considered violence usually or always justified to return Donald Trump to the White House that same year [8].

The 2024 US election, of course, did return Mr. Trump and a Republican administration to power. This development might be expected to produce an increase in support for political violence among Democrats and a decrease among Republicans, as political violence in the US is ordinarily more a tool of the opposition than of those in power. But Mr. Trump himself has repeatedly advocated the use of force by the federal government (and his supporters) against civilians in the US [28–30], and in 2025, his administration converted his advocacy into practice [31–33]. Because pro-violence rhetoric—let alone violent acts—by political leaders and governments results in increased support for violence among their followers [28, 34], it is plausible that support for political violence increased from 2024 to 2025 among Republicans, and particularly among MAGA Republicans.

With these possibilities in mind, 2025’s Wave 4 of the survey was designed in part to identify pre/post-election changes in support for and willingness to engage in political violence in the US. The survey repeated prior years’ questions on democracy and authoritarianism, on political violence, and on civil war. This report focuses primarily on findings for 2025 and change from 2024 to 2025 for the cohort as a whole. We also performed subgroup analyses with respondents categorized by political party and MAGA affiliation, given their associations with political violence [7, 13, 17–27]. Findings for political ideology closely parallelled those for party affiliation in a previous analysis [17], and that analysis was not repeated here.

The overarching aim of this study is to advance efforts to prevent widespread political violence in the US. The need for such efforts is reinforced by other findings from the 2025 survey [35]: nearly 10% of respondents agreed strongly or very strongly that the current federal administration “should use the military to help enforce its policies in the United States,” and between 2 and 3% were very or completely willing to commit violence themselves in support of or opposition to “the government’s enforcement of its policies.”

## Methods

Methods for Wave 4 of the survey closely followed those for Waves 1–3 [7, 8, 36]. The questionnaires were designed by the authors and administered online in English and Spanish by the survey research firm Ipsos [37]: Wave 4 from May 23 to June 13, 2025, and Wave 3 from May 23 to June 14, 2024. The study was reviewed by the University of California, Davis Institutional Review Board (protocol 187,125: exempt from full review, category 2, survey research). The IRB waived a requirement for written or verbal consent. Before participants accessed the questionnaire, they were provided informed consent language that concluded, “[by] continuing, you are agreeing to participate in this study.” The study is reported following American Association for Public Opinion Research guidelines [38].

### Participants

Participants were drawn from Ipsos KnowledgePanel, an online research panel that has been widely used in population-based research on violence and firearm ownership [39–44]. To establish a nationally representative panel, KnowledgePanel members are recruited on an ongoing basis through address-based probability sampling using data from the US Postal Service’s Delivery Sequence File [45, 46]. Recruitment into KnowledgePanel involves repeated contact attempts, if necessary, by mail and telephone. Recruited adults in households without internet access are provided a web-enabled device and free internet service, and a modest, primarily points-based incentive program seeks to encourage participation and promote participants’ retention in KnowledgePanel over time [45, 46].

A probability-proportional-to-size procedure was used to select a study-specific sample for Wave 1. All panel members who were ≥ 18 years old were eligible for selection. Invitations were sent by e-mail; automatic reminders were delivered to non-respondents by e-mail and telephone beginning 3 days later [45, 46].

The Wave 1 survey was conducted May 13 to June 2, 2022. It included a main sample, which provided the study population for our initial report [6], and oversamples of firearm owners, transgender people, combat veterans, and California residents that were recruited to ensure adequate power for planned subset analyses. Compared with main sample nonrespondents, main sample respondents were older and more frequently white and non-Hispanic, were more often married, had higher education and income, and were less likely to be working [7].

Including the main sample and oversamples, Wave 1 comprised 12,947 respondents (the completion rate for the main sample was 56.7%). Details of the survey cohort’s subsequent participation history are presented in the supplemental methods text and Figure S1 (see Supplement, Additional file 1). Invitations to participate in Wave 4 were sent to all 9,179 Wave 1 respondents who remained active members of KnowledgePanel on Wave 4’s launch date.

A final Wave 4 survey weight variable for longitudinal analyses was provided by Ipsos. It adjusted for the initial probability of selection into KnowledgePanel, for survey-specific nonresponse and over- or under-coverage, and for differential attrition using design weights with post-stratification raking ratio adjustments. As with prior samples, the weighted 2025 sample is designed to be statistically representative of the noninstitutionalized US adult population as reflected in the 2021 March supplement of the Current Population Survey [45, 46].

### Measures

Sociodemographic data were collected by Ipsos from profiles created and maintained by KnowledgePanel members. Political party and MAGA movement affiliations were reported by respondents in 2024; the categorization scheme is presented in the Supplement (see Supplement, Additional file 1).

Survey questions that supplied outcome data for this analysis covered 2 broad topics: beliefs regarding democracy and the potential for violence and civil war in the US, and support for and willingness to engage in political violence. As in prior years, our primary outcome measures concerned political violence. Violence was represented by the phrase “force or violence,” defined in the questionnaire as “physical force strong enough that it could cause pain or injury to a person.” “Force or violence to advance an important political objective that you support” was used in questions about respondents’ support for and willingness to engage in political violence.

Respondents were again asked about the extent to which they considered political violence to be justified “in general” and then about justification for its use to advance specified political objectives. Example objectives include “to preserve an American way of life based on Western European traditions,” and “to stop police violence.” Responses for 20 objectives were collected in both years. The objective “to return Donald Trump to the presidency this year” had been included in 2024; in 2025, this was replaced by “to remove Donald Trump from the presidency this year.”

Respondents in 2025 who considered political violence to be at least sometimes justified to advance at least 1 objective were asked about their personal willingness to engage in political violence: by type of violence (to “damage property,” “threaten or intimidate a person,” “injure a person,” “kill a person”), by target population (examples: “an elected federal or state government official,” “a police officer,” “a person who does not share your religion”), and by social context (examples: “on your own,” “as part of a group”).

All respondents were asked about the likelihood of their future use of firearms in a situation where they consider political violence to be justified (examples: “I will be armed with a gun,” “I will shoot someone with a gun”).

The full text of all questions reported on here, including sources for questions from prior surveys by other investigators, is in the Supplement (see Supplement, Additional file 1).

### Implementation

Ipsos translated the questionnaire into Spanish. Twenty-three KnowledgePanel members participated in a pretest of the English language version that was administered May 16–20, 2025.

Respondents were randomized 1:1 to receive response options in order from either negative to positive valence (example: from ‘do not agree’ to ‘strongly agree’) or the reverse throughout the questionnaire. Where a question presented multiple statements for respondents to consider, the order in which those statements were presented was randomized unless ordering was necessary. Logic-driving questions (those to which responses might invoke a skip pattern) included non-response prompts.

We employed unipolar response arrays without a neutral midpoint (example: do not agree, somewhat agree, strongly agree, very strongly agree). The literature is not in agreement on whether such midpoints should be included [47, 48]. We were persuaded by the studies reviewed by Chyung et. al [47], which suggest that such midpoints allow respondents to choose “a minimally acceptable response as soon as it is found, instead of putting effort to find an optimal response,” a behavior known as satisficing. According to those authors, satisficing is particularly common when respondents are uncomfortable with the topics of the survey or under social desirability pressures, and both conditions apply here. Our analyses focus on responses above the “somewhat” or “sometimes” level to minimize the impact of potential satisficing on the results.

### Statistical analysis

SAS version 9.4 (SAS Institute, Inc., Cary, NC) and IBM SPSS Statistics, version 30 (IBM Corp., Armonk, NY) were used for all analyses.

Survey items had ordinal response options. We combined the response options usually justified and always justified, very willing and completely willing, and very likely and extremely likely for the analysis. We reported unweighted frequencies and weighted prevalences for each item for each possible response. The percentage of respondents reporting that violence was usually or always justified to advance at least 1 political objective was calculated based on the 20 objectives presented to respondents in both years.

Prevalence estimates and 95% confidence intervals (CI) were calculated using PROC SURVEYFREQ in SAS and Complex Samples Frequencies in SPSS; mean differences and mean scores were calculated using PROC SURVEYMEANS in SAS and Complex Samples Descriptives in SPSS.

We summarized each item’s non-missing responses for each year by assigning integer values (1, 2, or 3) to ordinal levels to produce an item score for each respondent and then averaging these individual scores across the cohort and subgroups characterized by political party and MAGA movement affiliation.

To assess change from 2024 to 2025 for the cohort and subgroups, we first computed the within-individual change score between the two years for each item. We then calculated cohort-level year-to-year change based on the means of aggregated within-individual change scores. These mean change scores have a potential range from −2 to 2 (with 0 indicating no change). A change of 1 for an item would correspond to a 1-level increase in the sample’s mean response to that item (such as from “never justified” to “sometimes justified”); a change of −1 would correspond to a change in the opposite direction. We use the notation “change *x*, 95% CI *y*, *z*” [49] to report changes in mean scores. We considered changes to differ among subgroups (one larger than the other, for example) when their 95% confidence intervals did not overlap, which is a very conservative test [50].

There are no standard definitions for changes that are “small” or “large.” We have elected to characterize mean change scores on any political violence measure whose absolute value does not exceed 0.100 (10% of a 1-level change) as “small,” noting that a small cohort-level change could result from frequent but offsetting individual-level changes.

To compute adjusted prevalence differences (aPDs) and their 95% CIs for the political party/MAGA affiliation subgroups, we defined outcomes dichotomously and used PROC SURVEYREG in SAS, employing robust standard errors to correct for design effects and heteroskedasticity in binary outcomes and weights to account for the complex survey design. We employed a model that included respondent age, race and ethnicity, gender, income, education, Census division, marital status, homeownership, rurality, firearm ownership, alcohol consumption, military service, and history of non-traffic arrest (see Supplement, Additional file 1 for further detail). aPDs are measured in absolute percentage points (pp).

We controlled for multiple comparisons by bounding the false discovery rate (FDR) using the Benjamini-Yekutieli method [51]. The adjusted p-values are known as FDR-adjusted (or FDR-corrected) p-values or as q-values [52]; we employ the latter term here. For a given value of the false discovery rate, Q (Q = 0.05 was chosen here), the q-values are chosen so that, of comparisons that reject the null hypothesis (decided by q < α), the expected proportion of these comparisons that incorrectly reject the null hypothesis is at most Q.

## Results

The 2025 completion rate was 89.9% (8,248 respondents/9,179 invitees). The median survey completion time for all Wave 4 respondents was 24 min (interquartile range, 17 min). Item non-response in 2025 for items included in this analysis ranged from 0.4% to 3.4% and exceeded 3.0% for 1 item (see Supplement, Additional file 1).

After weighting, half of the respondents (50.7%, 95% CI 49.2%, 52.2%) were female; 62.7% (95% CI 61.2%, 64.3%) were white, non-Hispanic (see Supplement, Additional file 1, Table S1). The weighted mean (SD) respondent age was 51.5 (25.7) years. Nonrespondents were younger than respondents (unweighted mean (SD) ages 52.6 (17.6) and 59.7 (16.3), respectively) and less frequently male and white, non-Hispanic (see Supplement, Additional file 1, Table S2).

### Democracy and authoritarianism

From 2024 to 2025, there was a small increase in agreement with the view that there is no threat to democracy in the US (change 0.077, 95% CI 0.054, 0.100) (see Supplement, Additional file 1, Table S3). There was no change in the view that it was important “for the United States to remain a democracy” (change 0.012, 95% CI −0.003, 0.026) or that “democracy is the best form of government” (change 0.003, 95% CI −0.016, 0.022), but there was a small increase in agreement that “democracy only serves the interests of the wealthy and powerful” (change 0.068, 95% CI 0.042, 0.094). There were small decreases in agreement with 2 expressions of authoritarianism: that “having a strong leader for America is more important than having a democracy” (change −0.038, 95% CI −0.061, −0.014) and that “we should suspend Congress for a few years” (change −0.100, 95% CI −0.121, −0.079).

### Political violence

There were small decreases in agreement from 2024 to 2025 with 2 of 3 statements about conditions in the US creating a potential need for political violence (Table 1); a decrease for the third statement was not statistically significant. There was a small increase in agreement with the statement that “in the next few years, there will be civil war in the United States” (change 0.043, 95% CI 0.023, 0.064), but not with the statement that “United States needs a civil war to set things right” (change −0.002, 95% CI −0.017, 0.013) (Table 1).

**Table 1:** Beliefs concerning violence to effect social change and civil war

Statement	2024 Respondents* (n = 8896)		2025 Respondents* (n = 8248)		Mean difference,* 2024–2025	
	Unweighted	Weighted % (95% CI)	Unweighted	Weighted % (95% CI)	Unweighted	Weighted % (95% CI)
	n	*Mean score (95% CI)*	n	*Mean score (95% CI)*	n	*Mean score (95% CI)*
If elected leaders will not protect American democracy, the people must do it themselves, even if it requires taking violent actions						
Do not agree (1)	5546	62.4 (61.0, 63.8)	5140	62.1 (60.7, 63.5)	7767	−0.6 (−2.1, 1.0)
Somewhat agree (2)	2310	24.7 (23.5, 25.9)	2261	25.8 (24.5, 27.0)	7767	1.4 (−0.2, 3.0)
Strongly or very strongly agree (3)	901	10.3 (9.4, 11.2)	700	9.3 (8.4, 10.2)	7767	−0.9 (−2.0, 0.1)
Non-response	139	2.6 (2.0, 3.1)	147	2.8 (2.3, 3.4)	7767	0.0 (−0.5, 0.6)
*Mean score†*	*8757*	*1.47 (1.45, 1.49)*	*8101*	*1.46 (1.44, 1.48)*	*7578*	−*0.001 (*−*0.023, 0.021)*
Our American way of life is disappearing so fast that we may have to use force to save it						
Do not agree (1)	5583	61.9 (60.5, 63.3)	5236	61.2 (59.7, 62.7)	7767	−0.3 (−1.9, 1.3)
Somewhat agree (2)	2184	23.7 (22.5, 24.9)	2174	26.4 (25.1, 27.7)	7767	2.8 (1.1, 4.4)
Strongly or very strongly agree (3)	1005	12.1 (11.1, 13.1)	687	9.3 (8.4, 10.2)	7767	−3.1 (−4.3, −1.9)
Non-response	124	2.3 (1.8, 2.8)	151	3.1 (2.4, 3.7)	7767	0.6 (0.0, 1.1)
*Mean score†*	*8772*	*1.49 (1.47, 1.51)*	*8097*	*1.46 (1.44, 1.49)*	*7583*	−*0.028 (*−*0.050, *−*0.005)*
Because things have gotten so far off track, true American patriots may have to resort to violence in order to save our country						
Do not agree (1)	6578	72.1 (70.7, 73.4)	6225	73.2 (71.9, 74.6)	7767	1.3 (−0.2, 2.7)
Somewhat agree (2)	1539	17.4 (16.3, 18.5)	1469	18.1 (16.9, 19.2)	7767	0.8 (−0.7, 2.2)
Strongly or very strongly agree (3)	642	8.1 (7.3, 9.0)	418	5.9 (5.2, 6.7)	7767	−2.2 (−3.2, −1.2)
Non-response	137	2.4 (1.9, 2.9)	136	2.8 (2.2, 3.3)	7767	0.2 (−0.3, 0.6)
*Mean score†*	*8759*	*1.34 (1.33, 1.36)*	*8112*	*1.31 (1.29, 1.33)*	*7596*	−*0.035 (*−*0.055, *−*0.015)*
In the next few years, there will be civil war in the United States						
Do not agree (1)	5768	62.2 (60.8, 63.6)	5200	59.0 (57.5, 60.4)	7767	−3.4 (−5.0, −1.8)
Somewhat agree (2)	2524	28.8 (27.5, 30.1)	2433	31.3 (29.9, 32.6)	7767	2.7 (1.0, 4.4)
Strongly or very strongly agree (3)	465	6.5 (5.8, 7.3)	473	7.1 (6.3, 8.0)	7767	0.7 (−0.3, 1.6)
Non-response	139	2.4 (1.9, 2.9)	142	2.6 (2.1, 3.1)	7767	0.1 (−0.4, 0.6)
*Mean score†*	*8757*	*1.43 (1.41, 1.45)*	*8106*	*1.47 (1.45, 1.49)*	*7582*	*0.043 (0.023, 0.064)*
The United States needs a civil war to set things right						
Do not agree (1)	7745	84.9 (83.8, 86.0)	7278	85.1 (84.0, 86.2)	7767	0.1 (−1.0, 1.2)
Somewhat agree (2)	766	9.2 (8.3, 10.0)	634	9.2 (8.3, 10.1)	7767	0.5 (−0.6, 1.6)
Strongly or very strongly agree (3)	254	3.6 (3.0, 4.2)	211	3.3 (2.7, 3.9)	7767	−0.4 (−1.2, 0.3)
Non-response	131	2.3 (1.8, 2.8)	125	2.4 (1.9, 2.9)	7767	−0.2 (−0.7, 0.3)
*Mean score†*	*8765*	*1.17 (1.15, 1.18)*	*8123*	*1.16 (1.15, 1.18)*	*7603*	−*0.002 (*−*0.017, 0.013)*

^*^Among respondents to both surveys (n = 7767)

^†^Mean scores in 2024 and 2025 were scored as indicated in the response lines for individual questions, with non-responses excluded. To assess cohort-level change from 2024 to 2025, we computed within-individual change scores for each item and then calculated year-to-year cohort-level change scores based on the means of aggregated within-individual change scores. Mean change scores have a range from −2 to 2 (with 0 indicating no change)

There was a small increase from 2024 to 2025 in support for the uncommon view that political violence is usually or always justified “in general” (change 0.033, 95% CI 0.016, 0.051) and in the prevalence of the belief that violence was usually or always justified to advance at least 1 of 20 political objectives (2024: 32.3%, 95% CI 31.0%, 33.6%; 2025: 35.6%, 95% CI 34.1%, 37.0%; change 0.032, 95% CI 0.016, 0.046) (Table 2). There were small increases in the view that violence was usually or always justified to advance 16 of 20 objectives when these were considered individually (Tables 2 and 3).

**Table 2:** Justification for political violence “in general” and to advance specific political objectives

What do you think about the use of force or violence in the following situations?	2024 Respondents* (n = 8896)		2025 Respondents* (n = 8248)		Mean difference,* 2024–2025	
	Unweighted	Weighted % (95% CI)	Unweighted	Weighted % (95% CI)	Unweighted	Weighted % (95% CI)
	n	*Mean score (95% CI)*	n	*Mean score (95% CI)*	n	*Mean score (95% CI)*
In general…to advance an important political objective that you support						
Never justified (1)	7147	77.3 (76.0, 78.6)	6435	74.5 (73.1, 75.8)	7767	−3.0 (−4.4, −1.5)
Sometimes justified (2)	1583	19.5 (18.3, 20.7)	1635	21.8 (20.6, 23.1)	7767	2.6 (1.2, 4.0)
Usually or always justified (3)	108	2.0 (1.6, 2.5)	121	2.5 (1.9, 3.0)	7767	0.4 (−0.3, 1.1)
Non-response	58	1.2 (0.8, 1.5)	57	1.2 (0.8, 1.6)	7767	0.0 (−0.4, 0.4)
*Mean score†*	8838	*1.24 (1.22, 1.25)*	8191	*1.27 (1.26, 1.29)*	*7696*	*0.033 (0.016, 0.051)*
Violence is usually or always justified to advance at least 1 of 20 objectives	2982	32.3 (31.0, 33.6)	2908	35.6 (34.1, 37.0)	7696	*0.032 (0.016, 0.046)*
To stop an election from being stolen‡						
Never justified (1)	6722	76.9 (75.7, 78.1)	5853	71.6 (70.3, 73.0)	7696	−5.3 (−6.8, −3.8)
Sometimes justified (2)	1433	15.4 (14.4, 16.4)	1557	18.4 (17.3, 19.6)	7696	3.1 (1.7, 4.5)
Usually or always justified (3)	624	6.8 (6.1, 7.5)	724	9.0 (8.1, 9.9)	7696	2.1 (1.0, 3.1)
Non-response	59	0.9 (0.6, 1.2)	57	1.0 (0.6, 1.3)	7696	0.0 (−0.3, 0.4)
*Mean score†*	8779	*1.29 (1.28, 1.31)*	8134	*1.37 (1.35, 1.39)*	*7622*	*0.073 (0.051, 0.095)*
To stop people who do not share my beliefs from voting‡						
Never justified (1)	8415	92.9 (92.1, 93.8)	7777	92.3 (91.3, 93.2)	7696	−0.5 (−1.5, 0.4)
Sometimes justified (2)	244	4.0 (3.3, 4.6)	231	4.1 (3.4, 4.8)	7696	0.0 (−0.9, 0.8)
Usually or always justified (3)	133	2.4 (1.8, 2.9)	142	3.0 (2.4, 3.6)	7696	0.5 (−0.2, 1.2)
Non-response	46	0.8 (0.5, 1.0)	41	0.7 (0.4, 0.9)	7696	0.0 (−0.3, 0.3)
*Mean score†*	8792	*1.09 (1.08, 1.10)*	8150	*1.10 (1.09, 1.12)*	*7639*	*0.009 (*−*0.006, 0.023)*
To prevent discrimination based on race or ethnicity‡						
Never justified (1)	6580	71.9 (70.6, 73.2)	5595	65.0 (63.6, 66.4)	7696	−7.4 (−9.1, −5.8)
Sometimes justified (2)	1691	20.4 (19.2, 21.5)	1909	24.7 (23.4, 26.0)	7696	4.8 (3.2, 6.4)
Usually or always justified (3)	513	7.0 (6.2, 7.8)	641	9.6 (8.6, 10.5)	7696	2.5 (1.4, 3.6)
Non-response	54	0.8 (0.5, 1.0)	46	0.7 (0.5, 1.0)	7696	0.1 (−0.1, 0.4)
*Mean score†*	8784	*1.35 (1.33, 1.36)*	8145	*1.44 (1.42, 1.46)*	*7632*	*0.098 (0.075, 0.120)*
To preserve an American way of life based on Western European traditions‡						
Never justified (1)	6723	78.1 (76.9, 79.2)	6089	76.1 (74.8, 77.3)	7696	−1.9 (−3.4, −0.5)
Sometimes justified (2)	1608	16.2 (15.2, 17.3)	1623	17.8 (16.7, 18.9)	7696	1.4 (0.1, 2.8)
Usually or always justified (3)	443	4.8 (4.1, 5.4)	408	5.1 (4.4, 5.7)	7696	0.3 (−0.5, 1.2)
Non-response	64	1.0 (0.7, 1.3)	71	1.0 (0.7, 1.4)	7696	0.2 (−0.2, 0.5)
*Mean score†*	*8774*	*1.26 (1.24, 1.28)*	*8120*	*1.28 (1.27, 1.30)*	*7603*	*0.020 (0.000, 0.039)*
To preserve an American way of life I believe in‡						
Never justified (1)	5876	69.5 (68.2, 70.8)	5093	65.3 (63.9, 66.7)	7696	−4.4 (−5.9, −2.9)
Sometimes justified (2)	2188	21.8 (20.6, 22.9)	2275	24.4 (23.2, 25.6)	7696	2.8 (1.3, 4.3)
Usually or always justified (3)	714	7.8 (7.1, 8.6)	759	9.3 (8.4, 10.1)	7696	1.4 (0.4, 2.5)
Non-response	60	0.9 (0.6, 1.2)	64	1.0 (0.7, 1.3)	7696	0.1 (−0.2, 0.5)
*Mean score†*	*8778*	*1.38 (1.36, 1.40)*	*8127*	*1.43 (1.41, 1.45)*	*7611*	*0.055 (0.034, 0.077)*
To oppose Americans who do not share my beliefs‡						
Never justified (1)	8135	89.7 (88.7, 90.6)	7519	89.1 (88.0, 90.1)	7696	−0.6 (−1.7, 0.5)
Sometimes justified (2)	487	6.9 (6.1, 7.7)	473	7.2 (6.3, 8.0)	7696	0.3 (−0.8, 1.3)
Usually or always justified (3)	170	2.8 (2.2, 3.3)	151	2.9 (2.3, 3.5)	7696	0.2 (−0.4, 0.9)
Non-response	46	0.7 (0.4, 1.0)	48	0.8 (0.5, 1.2)	7696	0.1 (−0.1, 0.4)
*Mean score†*	*8792*	*1.12 (1.11, 1.14)*	*8143*	*1.13 (1.12, 1.15)*	*7637*	*0.007 (*−*0.008, 0.021)*
To oppose the government when it does not share my beliefs‡						
Never justified (1)	7459	82.3 (81.2, 83.5)	6856	81.2 (79.9, 82.4)	7696	−1.6 (−3.0, −0.2)
Sometimes justified (2)	1095	13.6 (12.5, 14.6)	1058	13.9 (12.8, 15.0)	7696	0.7 (−0.7, 2.1)
Usually or always justified (3)	230	3.3 (2.8, 3.9)	222	4.1 (3.4, 4.8)	7696	0.8 (0.0, 1.6)
Non-response	54	0.8 (0.5, 1.0)	55	0.8 (0.6, 1.1)	7696	0.1 (−0.2, 0.5)
*Mean score†*	*8784*	*1.20 (1.19, 1.22)*	*8136*	*1.22 (1.21, 1.24)*	*7623*	*0.021 (0.003, 0.039)*
To oppose the government when it tries to take private land for public purposes‡						
Never justified (1)	5795	65.7 (64.4, 67.1)	5108	62.1 (60.6, 63.5)	7696	−3.9 (−5.6, -2.2)
Sometimes justified (2)	2295	24.9 (23.7, 26.2)	2364	27.6 (26.3, 28.9)	7696	3.1 (1.4, 4.7)
Usually or always justified (3)	693	8.5 (7.7, 9.4)	664	9.4 (8.5, 10.4)	7696	0.7 (−0.4, 1.8)
Non-response	55	0.8 (0.5, 1.1)	55	0.9 (0.6, 1.1)	7696	0.1 (−0.2, 0.4)
*Mean score†*	*8783*	*1.42 (1.40, 1.44)*	*8136*	*1.47 (1.45, 1.49)*	*7624*	*0.045 (0.021, 0.068)*

^*^ Among respondents to both surveys (n = 7767)

^†^ Mean scores in 2024 and 2025 were scored as indicated in the response lines for individual questions, with non-responses excluded. To assess cohort-level change from 2024 to 2025, we computed within-individual change scores for each item and then calculated year-to-year cohort-level change scores based on the means of aggregated within-individual change scores. Mean change scores have a range from −2 to 2 (with 0 indicating no change)

^‡^ Respondents who did not answer the question "In general…to advance an important political objective that you support" in 2024 (n = 58) or 2025 (n = 57) were not asked these questions

**Table 3:** Justification for violence to advance additional specific political objectives

What do you think about the use of force or violence in the following situations?	2024 Respondents* (n = 8896)		2025 Respondents* (n = 8248)		Mean difference,* 2024–2025	
	Unweighted	Weighted % (95% CI)	Unweighted	Weighted % (95% CI)	Unweighted	Weighted % (95% CI)
	n	*Mean score (95% CI)*	n	*Mean score (95% CI)*	n	*Mean score (95% CI)*
To stop voter fraud						
Never justified (1)	6762	77.2 (76.0, 78.4)	5892	72.8 (71.5, 74.1)	7696	−4.5 (−6.0, −3.1)
Sometimes justified (2)	1328	14.4 (13.4, 15.4)	1451	16.6 (15.5, 17.7)	7696	2.4 (1.1, 3.8)
Usually or always justified (3)	703	7.7 (7.0, 8.5)	795	9.7 (8.8, 10.6)	7696	1.9 (0.9, 2.9)
Non-response	45	0.7 (0.5, 1.0)	53	0.9 (0.7, 1.3)	7696	0.2 (−0.1, 0.5)
*Mean score†*	*8793*	*1.30 (1.28, 1.32)*	*8138*	*1.36 (1.34, 1.38)*	*7633*	*0.063 (0.042, 0.084)*
To stop voter intimidation						
Never justified (1)	5997	68.9 (67.5, 70.2)	5138	64.4 (62.9, 65.8)	7696	−4.8 (−6.4, −3.2)
Sometimes justified (2)	2129	22.9 (21.7, 24.1)	2232	25.8 (24.6, 27.1)	7696	2.9 (1.3, 4.5)
Usually or always justified (3)	658	7.5 (6.7, 8.3)	773	9.0 (8.2, 9.9)	7696	1.9 (0.8, 2.9)
Non-response	54	0.8 (0.5, 1.1)	48	0.8 (0.5, 1.1)	7696	0.0 (−0.2, 0.3)
*Mean score†*	*8784*	*1.38 (1.36, 1.40)*	*8143*	*1.44 (1.42, 1.46)*	*7631*	*0.065 (0.044, 0.087)*
To stop police violence						
Never justified (1)	4934	55.3 (53.9, 56.8)	4362	51.1 (49.6, 52.6)	7696	−4.8 (−6.5, −3.1)
Sometimes justified (2)	3064	33.8 (32.4, 35.1)	2893	34.9 (33.5, 36.3)	7696	1.7 (0.0, 3.4)
Usually or always justified (3)	786	10.1 (9.2, 11.1)	880	13.2 (12.2, 14.3)	7696	3.0 (1.8, 4.2)
Non-response	54	0.8 (0.5, 1.1)	56	0.8 (0.6, 1.2)	7696	0.1 (−0.1, 0.4)
*Mean score†*	*8784*	*1.54 (1.52, 1.56)*	*8135*	*1.62 (1.60, 1.64)*	*7624*	*0.077 (0.053, 0.101)*
To reinforce the police						
Never justified (1)	4346	56.5 (55.1, 57.9)	3922	53.1 (51.7, 54.6)	7696	−3.2 (−4.9, −1.5)
Sometimes justified (2)	3238	31.5 (30.2, 32.8)	3121	34.0 (32.6, 35.4)	7696	2.5 (0.8, 4.1)
Usually or always justified (3)	1200	11.3 (10.4, 12.1)	1084	11.9 (11.0, 12.9)	7696	0.4 (−0.8, 1.6)
Non-response	54	0.8 (0.5, 1.1)	64	1.0 (0.7, 1.3)	7696	0.3 (0.0, 0.6)
*Mean score†*	*8784*	*1.54 (1.53, 1.56)*	*8127*	*1.58 (1.56, 1.60)*	*7616*	*0.032 (0.009, 0.056)*
To stop illegal immigration						
Never justified (1)	5204	63.3 (62.0, 64.7)	4691	61.2 (59.8, 62.6)	7696	−2.8 (−4.3, −1.3)
Sometimes justified (2)	2249	22.8 (21.7, 24.0)	2185	23.7 (22.5, 24.9)	7696	1.9 (0.4, 3.4)
Usually or always justified (3)	1339	13.0 (12.1, 14.0)	1269	14.3 (13.3, 15.4)	7696	0.7 (−0.4, 1.8)
Non-response	46	0.8 (0.6, 1.2)	46	0.8 (0.5, 1.2)	7696	0.2 (0.0, 0.5)
*Mean score†*	*8792*	*1.49 (1.47, 1.51)*	*8145*	*1.53 (1.51, 1.55)*	*7637*	*0.034 (0.012, 0.055)*
To keep borders open						
Never justified (1)	7107	79.1 (77.9, 80.3)	6375	75.5 (74.2, 76.8)	7696	−4.5 (−5.9, −3.0)
Sometimes justified (2)	1187	14.0 (13.0, 15.0)	1232	16.2 (15.1, 17.3)	7696	2.7 (1.3, 4.1)
Usually or always justified (3)	491	5.9 (5.3, 6.7)	527	7.4 (6.6, 8.3)	7696	1.6 (0.6, 2.6)
Non-response	53	1.0 (0.7, 1.4)	57	0.9 (0.6, 1.2)	7696	0.2 (−0.1, 0.5)
*Mean score†*	*8785*	*1.26 (1.24, 1.28)*	*8134*	*1.31 (1.29, 1.33)*	*7620*	*0.059 (0.038, 0.079)*
To stop a protest or demonstration						
Never justified (1)	5749	67.1 (65.7, 68.4)	5505	68.1 (66.7, 69.5)	7696	0.9 (−0.7, 2.5)
Sometimes justified (2)	2557	26.5 (25.3, 27.8)	2255	25.3 (24.1, 26.6)	7696	−1.0 (−2.6, 0.6)
Usually or always justified (3)	485	5.7 (5.1, 6.4)	378	5.7 (5.0, 6.5)	7696	−0.1 (−1.0, 0.8)
Non-response	47	0.7 (0.5, 1.0)	53	0.9 (0.6, 1.2)	7696	0.2 (−0.1, 0.4)
*Mean score†*	*8791*	*1.38 (1.36, 1.40)*	*8138*	*1.37 (1.35, 1.39)*	*7632*	−*0.012 (*−*0.033, 0.008)*
To support a protest or demonstration						
Never justified (1)	7389	80.7 (79.4, 81.8)	6682	78.2 (76.9, 79.4)	7696	−3.0 (−4.4, −1.5)
Sometimes justified (2)	1142	14.9 (13.9, 16.1)	1191	16.3 (15.2, 17.5)	7696	1.7 (0.3, 3.0)
Usually or always justified (3)	260	3.7 (3.1, 4.3)	269	4.7 (4.0, 5.4)	7696	1.1 (0.3, 1.9)
Non-response	47	0.7 (0.5, 1.1)	49	0.8 (0.6, 1.2)	7696	0.2 (−0.1, 0.5)
*Mean score†*	*8791*	*1.22 (1.21, 1.24)*	*8142*	*1.26 (1.24, 1.28)*	*7632*	*0.039 (0.020, 0.058)*
To protect the environment or stop climate change						
Never justified (1)	7189	78.0 (76.7, 79.2)	6323	73.6 (72.3, 75.0)	7696	−4.8 (−6.3, −3.4)
Sometimes justified (2)	1123	14.4 (13.4, 15.5)	1249	16.7 (15.6, 17.9)	7696	2.4 (1.1, 3.7)
Usually or always justified (3)	475	6.7 (6.0, 7.5)	569	8.9 (8.0, 9.8)	7696	2.3 (1.3, 3.3)
Non-response	51	0.9 (0.6, 1.3)	50	0.8 (0.5, 1.1)	7696	0.2 (−0.2, 0.5)
*Mean score†*	*8787*	*1.28 (1.26, 1.30)*	*8141*	*1.35 (1.33, 1.37)*	*7630*	*0.070 (0.050, 0.090)*
To protect the rights of animals						
Never justified (1)	6198	68.8 (67.4, 70.1)	5583	65.5 (64.0, 66.9)	7696	−3.6 (−5.1, −2.1)
Sometimes justified (2)	1812	20.3 (19.1, 21.4)	1741	21.9 (20.7, 23.2)	7696	1.6 (0.1, 3.1)
Usually or always justified (3)	776	10.1 (9.2, 11.0)	821	11.9 (10.9, 12.9)	7696	1.8 (0.7, 2.9)
Non-response	52	0.9 (0.7, 1.3)	46	0.8 (0.6, 1.2)	7696	0.1 (−0.2, 0.4)
*Mean score†*	*8786*	*1.41 (1.39, 1.43)*	*8145*	*1.46 (1.44, 1.48)*	*7633*	*0.053 (0.032, 0.075)*
To support women’s reproductive rights						
Never justified (1)	7015	76.5 (75.2, 77.7)	6124	71.3 (69.9, 72.7)	7696	−5.6 (−7.0, −4.1)
Sometimes justified (2)	1138	14.5 (13.5, 15.6)	1311	17.7 (16.5, 18.9)	7696	3.3 (1.9, 4.7)
Usually or always justified (3)	634	8.2 (7.4, 9.0)	697	10.1 (9.2, 11.1)	7696	2.0 (1.0, 3.1)
Non-response	51	0.9 (0.6, 1.3)	59	0.9 (0.6, 1.2)	7696	0.2 (0.0, 0.5)
*Mean score†*	*8787*	*1.31 (1.29, 1.33)*	*8132*	*1.38 (1.36, 1.40)*	*7625*	*0.074 (0.053, 0.095)*
To support the right to life						
Never justified (1)	6824	75.7 (74.4, 76.9)	6107	72.5 (71.1, 73.8)	7696	−3.7 (−5.3, -2.2)
Sometimes justified (2)	1213	14.1 (13.1, 15.1)	1239	15.8 (14.7, 16.9)	7696	2.1 (0.7, 3.5)
Usually or always justified (3)	747	9.2 (8.4, 10.1)	788	10.9 (10.0, 11.9)	7696	1.5 (0.4, 2.5)
Non-response	54	1.0 (0.7, 1.4)	57	0.8 (0.6, 1.2)	7696	0.2 (-0.2, 0.5)
*Mean score†*	*8784*	*1.33 (1.31, 1.35)*	*8134*	*1.38 (1.36, 1.40)*	*7621*	*0.050 (0.029, 0.072)*

^*^ Among respondents to both surveys (n = 7767). Respondents who did not answer the question "In general…to advance an important political objective that you support" in 2024 (n = 58) or 2025 (n = 57) were not asked these questions

^†^ Mean scores in 2024 and 2025 were scored as indicated in the response lines for individual questions, with non-responses excluded. To assess cohort-level change from 2024 to 2025, we computed within-individual change scores for each item and then calculated year-to-year cohort-level change scores based on the means of aggregated within-individual change scores. Mean change scores have a range from −2 to 2 (with 0 indicating no change)

Among respondents who considered political violence to be justified, there was no change from 2024 to 2025 in personal willingness to “damage property,” “threaten or intimidate a person,” or “injure a person,” and there was a small decrease in willingness to “kill a person” (Table 4). There were no increases in willingness to use force or violence against specified target populations (Table 5), and there was a small decrease in support for violence against police officers (change −0.016, 95% CI −0.030, −0.001). There were small increases in willingness to engage in violence as part of a group (change 0.060, 95% CI 0.037, 0.084) and as a lone actor (change 0.063, 95% CI 0.039, 0.087), and in willingness to organize group violence (change 0.064, 95% CI 0.041, 0.087) (Table 6). There were no changes in expectations of firearm possession and use in future situations where respondents considered political violence to be justified (Table 7).

**Table 4:** Personal willingness to commit political violence, by type of violence

In a situation where you think force or violence is justified to advance an important political objective…How willing would you personally be to use force or violence in each of these ways?	2024 Respondents* (n = 8896)		2025 Respondents* (n = 8248)		Mean difference,* 2024–2025	
	Unweighted	Weighted % (95% CI)	Unweighted	Weighted % (95% CI)	Unweighted	Weighted % (95% CI)
	n	*Mean score (95% CI)*	n	*Mean score (95% CI)*	n	*Mean score (95% CI)*
To damage property						
Not asked the question†	2013	27.0 (25.8, 28.4)	1653	22.3 (21.1, 23.6)	7696	−5.1 (−6.7, −3.5)
Not willing (1)	5978	61.5 (60.1, 62.9)	5736	66.3 (64.8, 67.7)	7696	4.8 (2.9, 6.6)
Somewhat willing (2)	619	8.2 (7.4, 9.1)	596	7.8 (7.0, 8.6)	7696	−0.3 (−1.4, 0.8)
Very or completely willing (3)	188	2.6 (2.2, 3.2)	162	2.7 (2.2, 3.3)	7696	0.2 (−0.4, 0.9)
Non-response	40	0.6 (0.4, 0.9)	44	0.9 (0.6, 1.3)	7696	0.4 (0.0, 0.7)
*Mean score‡*	6785	1.19 (1.17, 1.20)	6494	1.17 (1.16, 1.19)	*5145*	−*0.008 (*−*0.028, 0.013)*
To threaten or intimidate a person						
Not asked the question†	2013	27.0 (25.8, 28.4)	1653	22.3 (21.1, 23.6)	7696	−5.1 (−6.7, −3.5)
Not willing (1)	5954	62.1 (60.7, 63.5)	5715	66.5 (65.1, 67.9)	7696	4.9 (3.1, 6.7)
Somewhat willing (2)	663	7.8 (7.0, 8.6)	629	7.9 (7.1, 8.8)	7696	−0.1 (−1.1, 1.0)
Very or completely willing (3)	161	2.4 (1.9, 2.9)	141	2.4 (2.0, 3.0)	7696	0.0 (−0.6, 0.6)
Non-response	47	0.7 (0.5, 1.0)	53	0.8 (0.6, 1.2)	7696	0.3 (−0.1, 0.6)
*Mean score‡*	*6778*	*1.17 (1.16, 1.19)*	*6485*	*1.17 (1.15, 1.18)*	*5136*	−*0.016 (*−*0.036, 0.003)*
To injure a person						
Not asked the question†	2013	27.0 (25.8, 28.4)	1653	22.3 (21.1, 23.6)	7696	−5.1 (−6.7, −3.5)
Not willing (1)	6146	64.2 (62.8, 65.6)	5888	68.4 (67.0, 69.8)	7696	4.6 (2.8, 6.3)
Somewhat willing (2)	474	5.9 (5.2, 6.6)	484	6.6 (5.8, 7.4)	7696	0.8 (−0.1, 1.7)
Very or completely willing (3)	155	2.2 (1.8, 2.7)	118	1.8 (1.4, 2.3)	7696	−0.5 (−1.1, 0.1)
Non-response	50	0.7 (0.5, 1.0)	48	0.8 (0.6, 1.2)	7696	0.3 (−0.1, 0.6)
*Mean score‡*	*6775*	*1.14 (1.13, 1.16)*	*6490*	*1.13 (1.12, 1.15)*	*5136*	−*0.017 (*−*0.036, 0.002)*
To kill a person						
Not asked the question†	2013	27.0 (25.8, 28.4)	1653	22.3 (21.1, 23.6)	7696	−5.1 (−6.7, -3.5)
Not willing (1)	6317	66.4 (65.0, 67.7)	6094	71.3 (69.9, 72.6)	7696	5.4 (3.7, 7.1)
Somewhat willing (2)	318	3.8 (3.3, 4.5)	284	3.8 (3.2, 4.4)	7696	−0.2 (−0.9, 0.6)
Very or completely willing (3)	136	2.0 (1.6, 2.5)	117	1.8 (1.5, 2.3)	7696	−0.2 (−0.8, 0.4)
Non-response	54	0.8 (0.6, 1.1)	43	0.8 (0.6, 1.2)	7696	0.0 (−0.3, 0.4)
*Mean score‡*	*6771*	*1.11 (1.09, 1.12)*	*6495*	*1.10 (1.08, 1.11)*	*5132*	−*0.018 (*−*0.035, *−*0.001)*

^*^ Among respondents to both surveys (n = 7767). Respondents who did not answer the question "In general…to advance an important political objective that you support" in 2024 (n = 58) or 2025 (n = 57) were not asked these questions

^†^ Respondents answered “never justified” to all prior questions on the use of force or violence to advance specific political objectives were not asked questions on their personal willingness to use political violence

^‡^ Mean scores in 2024 and 2025 were scored as indicated in the response lines for individual questions, with non-responses excluded. To assess cohort-level change from 2024 to 2025, we computed within-individual change scores for each item and then calculated year-to-year cohort-level change scores based on the means of aggregated within-individual change scores. Mean change scores have a range from −2 to 2 (with 0 indicating no change)

**Table 5:** Personal willingness to engage in political violence, by target of violence

In a situation where you think force or violence is justified to advance an important political objective…How willing would you personally be to use force or violence against a person because they are…	2024 Respondents* (n = 8896)		2025 Respondents* (n = 8248)		Mean difference,* 2024–2025	
	Unweighted	Weighted % (95% CI)	Unweighted	Weighted % (95% CI)	Unweighted	Weighted % (95% CI)
	n	*Mean score (95% CI)*	n	*Mean score (95% CI)*	n	*Mean score (95% CI)*
An elected federal or state government official						
Not asked the question†	2013	27.0 (25.8, 28.4)	1653	22.3 (21.1, 23.6)	7696	−5.1 (−6.7, −3.5)
Not willing (1)	6266	65.4 (64.0, 66.8)	5988	69.8 (68.4, 71.2)	7696	4.8 (3.0, 6.5)
Somewhat willing (2)	356	4.7 (4.1, 5.4)	352	4.6 (4.0, 5.3)	7696	−0.1 (−1.0, 0.7)
Very or completely willing (3)	143	2.0 (1.6, 2.5)	115	1.9 (1.5, 2.4)	7696	−0.1 (−0.7, 0.5)
Non-response	60	0.8 (0.6, 1.1)	83	1.3 (1.0, 1.8)	7696	0.6 (0.2, 1.0)
*Mean score‡*	*6765*	*1.12 (1.11, 1.14)*	*6455*	*1.11 (1.10, 1.12)*	*5106*	−*0.009 (*−*0.026, 0.008)*
An elected local government official						
Not asked the question†	2013	27.0 (25.8, 28.4)	1653	22.3 (21.1, 23.6)	7696	−5.1 (−6.7, −3.5)
Not willing (1)	6302	66.0 (64.6, 67.3)	6039	70.2 (68.8, 71.5)	7696	4.7 (2.9, 6.4)
Somewhat willing (2)	333	4.3 (3.7, 5.0)	296	3.8 (3.3, 4.4)	7696	−0.6 (−1.4, 0.2)
Very or completely willing (3)	127	1.9 (1.5, 2.4)	114	2.2 (1.7, 2.8)	7696	0.3 (−0.3, 1.0)
Non-response	63	0.8 (0.6, 1.1)	89	1.5 (1.1, 2.0)	7696	0.7 (0.3, 1.1)
*Mean score‡*	*6762*	*1.11 (1.10, 1.13)*	*6449*	*1.11 (1.09, 1.12)*	*5094*	−*0.006 (*−*0.024, 0.013)*
An election worker, such as a poll worker or vote counter						
Not asked the question†	2013	27.0 (25.8, 28.4)	1653	22.3 (21.1, 23.6)	7696	−5.1 (−6.7, −3.5)
Not willing (1)	6472	67.7 (66.3, 69.0)	6179	71.5 (70.1, 72.8)	7696	4.6 (2.8, 6.3)
Somewhat willing (2)	184	2.5 (2.1, 3.1)	187	3.0 (2.5, 3.6)	7696	−0.3 (−1.0, 0.5)
Very or completely willing (3)	107	1.9 (1.4, 2.4)	90	1.8 (1.4, 2.3)	7696	0.3 (−0.3, 0.9)
Non-response	62	0.9 (0.7, 1.2)	82	1.4 (1.1, 1.9)	7696	0.5 (0.1, 0.9)
*Mean score‡*	*6763*	*1.09 (1.07, 1.10)*	*6456*	*1.09 (1.07, 1.10)*	*5094*	*0.001 (*−*0.017, 0.019)*
A public health official						
Not asked the question†	2013	27.0 (25.8, 28.4)	1653	22.3 (21.1, 23.6)	7696	−5.1 (−6.7, −3.5)
Not willing (1)	6398	66.8 (65.4, 68.2)	6132	71.0 (69.6, 72.3)	7696	4.5 (2.7, 6.2)
Somewhat willing (2)	237	3.2 (2.7, 3.9)	211	3.0 (2.5, 3.6)	7696	0.1 (−0.7, 0.9)
Very or completely willing (3)	117	1.9 (1.5, 2.5)	107	2.3 (1.8, 2.9)	7696	0.0 (−0.5, 0.5)
Non-response	73	1.0 (0.7, 1.3)	88	1.4 (1.1, 1.8)	7696	0.5 (0.1, 0.9)
*Mean score‡*	*6752*	*1.10 (1.08, 1.11)*	*6450*	*1.10 (1.08, 1.12)*	*5090*	−*0.003 (*−*0.018, 0.013)*
A member of the military or National Guard						
Not asked the question†	2013	27.0 (25.8, 28.4)	1653	22.3 (21.1, 23.6)	7696	−5.1 (−6.7, −3.5)
Not willing (1)	6361	66.3 (64.9, 67.7)	6053	70.4 (69.0, 71.7)	7696	4.2 (2.4, 5.9)
Somewhat willing (2)	267	3.8 (3.2, 4.4)	274	3.8 (3.2, 4.5)	7696	0.3 (−0.7, 1.3)
Very or completely willing (3)	126	2.0 (1.6, 2.5)	121	2.1 (1.6, 2.6)	7696	0.0 (−0.6, 0.7)
Non-response	71	0.9 (0.7, 1.2)	90	1.4 (1.1, 1.9)	7696	0.6 (0.2, 1.1)
*Mean score‡*	*6754*	*1.11 (1.09, 1.12)*	*6448*	*1.10 (1.09, 1.12)*	*5097*	−*0.002 (*−*0.019, 0.016)*
A police officer						
Not asked the question†	2013	27.0 (25.8, 28.4)	1653	22.3 (21.1, 23.6)	7696	−5.1 (−6.7, −3.5)
Not willing (1)	6285	65.1 (63.6, 66.5)	5978	68.8 (67.3, 70.2)	7696	5.1 (3.4, 6.8)
Somewhat willing (2)	338	4.9 (4.2, 5.6)	347	5.2 (4.5, 6.0)	7696	−0.3 (−1.0, 0.4)
Very or completely willing (3)	143	2.2 (1.8, 2.8)	124	2.3 (1.8, 2.8)	7696	−0.1 (−0.6, 0.4)
Non-response	59	0.8 (0.6, 1.1)	89	1.4 (1.1, 1.9)	7696	0.3 (−0.1, 0.7)
*Mean score‡*	*6766*	*1.13 (1.11, 1.15)*	*6449*	*1.13 (1.11, 1.14)*	*5102*	−*0.016 (*−*0.030, *−*0.001)*
A person who does not share your race or ethnicity						
Not asked the question†	2013	27.0 (25.8, 28.4)	1653	22.3 (21.1, 23.6)	7696	4.8 (3.2, 6.5)
Not willing (1)	6454	67.5 (66.1, 68.9)	6204	72.3 (71.0, 73.7)	7696	0.0 (−0.6, 0.7)
Somewhat willing (2)	210	2.9 (2.4, 3.4)	160	2.5 (2.0, 3.0)	7696	−0.2 (−0.8, 0.4)
Very or completely willing (3)	97	1.7 (1.3, 2.2)	89	1.7 (1.3, 2.2)	7696	0.4 (0.0, 0.8)
Non-response	64	0.9 (0.6, 1.2)	85	1.2 (0.9, 1.6)	7696	−5.1 (−6.7, −3.5)
*Mean score‡*	*6761*	*1.09 (1.07, 1.10)*	*6453*	*1.08 (1.06, 1.09)*	*5102*	−*0.009 (*−*0.024, 0.006)*
A person who does not share your religion						
Not asked the question†	2013	27.0 (25.8, 28.4)	1653	22.3 (21.1, 23.6)	7696	4.1 (2.4, 5.8)
Not willing (1)	6479	67.7 (66.3, 69.1)	6199	72.1 (70.7, 73.5)	7696	0.6 (−0.1, 1.3)
Somewhat willing (2)	177	2.6 (2.1, 3.2)	182	2.7 (2.2, 3.3)	7696	−0.2 (−0.8, 0.4)
Very or completely willing (3)	103	1.8 (1.4, 2.3)	73	1.5 (1.1, 2.1)	7696	0.6 (0.2, 1.0)
Non-response	66	0.9 (0.6, 1.2)	84	1.3 (1.0, 1.8)	7696	−5.1 (−6.7, −3.5)
*Mean score‡*	*6759*	*1.09 (1.07, 1.10)*	*6454*	*1.08 (1.06, 1.09)*	*5104*	*0.001 (*−*0.014, 0.016)*
A person who does not share your political beliefs						
Not asked the question†	2013	27.0 (25.8, 28.4)	1653	22.3 (21.1, 23.6)	7696	4.6 (2.8, 6.3)
Not willing (1)	6383	66.3 (64.9, 67.7)	6095	70.5 (69.1, 71.9)	7696	0.2 (−0.5, 0.9)
Somewhat willing (2)	265	3.9 (3.3, 4.6)	272	4.1 (3.5, 4.8)	7696	0.1 (−0.5, 0.7)
Very or completely willing (3)	107	1.8 (1.4, 2.3)	95	1.9 (1.5, 2.4)	7696	0.3 (−0.1, 0.7)
Non-response	70	0.9 (0.7, 1.3)	76	1.1 (0.8, 1.5)	7696	−5.1 (−6.7, −3.5)
*Mean score‡*	*6755*	*1.10 (1.09, 1.12)*	*6462*	*1.10 (1.09, 1.12)*	*5103*	−*0.002 (*−*0.018, 0.013)*

^*^ Among respondents to both surveys (n = 7767). Respondents who did not answer the question "In general…to advance an important political objective that you support" in 2024 (n = 58) or 2025 (n = 57) were not asked these questions

^†^ Respondents answered “never justified” to all prior questions on the use of force or violence to advance specific political objectives were not asked questions on their personal willingness to use political violence

^‡^ Mean scores in 2024 and 2025 were scored as indicated in the response lines for individual questions, with non-responses excluded. To assess cohort-level change from 2024 to 2025, we computed within-individual change scores for each item and then calculated year-to-year cohort-level change scores based on the means of aggregated within-individual change scores. Mean change scores have a range from −2 to 2 (with 0 indicating no change)

**Table 6:** Personal willingness to commit political violence, by social context of violence

You agreed that the use of force or violence could be justified to advance (one/some) of the political objectives we just discussed. In (that/those) (situation/situations), how willing would you personally be to…	2024 Respondents* (n = 8896)		2025 Respondents* (n = 8248)		Mean difference,* 2024–2025	
	Unweighted	Weighted % (95% CI)	Unweighted	Weighted % (95% CI)	Unweighted	Weighted % (95% CI)
	n	*Mean score (95% CI)*	n	*Mean score (95% CI)*	n	*Mean score (95% CI)*
Use force or violence as part of a group of people who share your beliefs						
Not asked the question†	2013	27.0 (25.7, 28.3)	1653	22.3 (21.1, 23.6)	7696	−5.1 (−6.7, −3.5)
Not willing (1)	5548	58.6 (57.2, 60.0)	5312	62.8 (61.4, 64.3)	7696	4.6 (2.8, 6.5)
Somewhat willing (2)	1042	11.1 (10.2, 12.0)	989	10.8 (9.9, 11.7)	7696	−0.3 (−1.4, 0.8)
Very or completely willing (3)	178	2.4 (1.9, 2.9)	178	3.0 (2.4, 3.6)	7696	0.5 (−0.1, 1.2)
Non-response	57	0.8 (0.6, 1.1)	59	1.1 (0.7, 1.4)	7696	0.2 (−0.2, 0.6)
*Mean score‡*	*8781*	*0.89 (0.87, 0.91)*	*8132*	*0.94 (0.92, 0.96)*	*7609*	*0.060 (0.037, 0.084)*
Use force or violence on your own, as an individual						
Not asked the question†	2013	27.0 (25.7, 28.3)	1653	22.3 (21.1, 23.6)	7696	−5.1 (−6.7, −3.5)
Not willing (1)	5199	55.5 (54.1, 57.0)	4964	59.4 (57.9, 60.8)	7696	3.8 (1.9, 5.7)
Somewhat willing (2)	1262	12.7 (11.8, 13.6)	1243	13.7 (12.7, 14.7)	7696	1.3 (0.1, 2.5)
Very or completely willing (3)	308	3.8 (3.2, 4.4)	276	3.7 (3.1, 4.3)	7696	−0.1 (−0.8, 0.7)
Non-response	56	0.9 (0.6, 1.2)	55	0.9 (0.6, 1.2)	7696	0.1 (−0.3, 0.4)
*Mean score‡*	*8782*	*0.93 (0.91, 0.96)*	*8136*	*0.99 (0.97, 1.01)*	*7613*	*0.063 (0.039, 0.087)*
Organize a group of people who share your beliefs to use force or violence						
Not asked the question†	2013	27.0 (25.7, 28.3)	1653	22.3 (21.1, 23.6)	7696	−5.1 (−6.7, −3.5)
Not willing (1)	6034	63.1 (61.7, 64.5)	5768	67.1 (65.7, 68.5)	7696	4.4 (2.7, 6.2)
Somewhat willing (2)	601	6.9 (6.1, 7.6)	555	6.9 (6.1, 7.7)	7696	0.0 (−0.9, 1.0)
Very or completely willing (3)	143	2.2 (1.8, 2.7)	162	2.8 (2.2, 3.3)	7696	0.5 (−0.2, 1.1)
Non-response	47	0.7 (0.5, 1.0)	53	0.9 (0.6, 1.2)	7696	0.1 (−0.2, 0.5)
*Mean score‡*	*8791*	*0.84 (0.82, 0.86)*	*8138*	*0.90 (0.88, 0.92)*	*7622*	*0.064 (0.041, 0.087)*

^*^ Among respondents to both surveys (n = 7767). Respondents who did not answer the question "In general…to advance an important political objective that you support" in 2024 (n = 58) or 2025 (n = 57) were not asked these questions

^†^ Respondents answered “never justified” to all prior questions on the use of force or violence to advance specific political objectives were not asked questions on their personal willingness to use political violence

^‡^ Mean scores in 2024 and 2025 were scored as indicated in the response lines for individual questions, with non-responses excluded. To assess cohort-level change from 2024 to 2025, we computed within-individual change scores for each item and then calculated year-to-year cohort-level change scores based on the means of aggregated within-individual change scores. Mean change scores have a range from −2 to 2 (with 0 indicating no change)

**Table 7:** Future firearm possession and use when political violence is perceived as justified

Thinking now about the future and all the changes it might bring, how likely is it that you will use a gun in any of the following ways in the next few years—in a situation where you think force or violence is justified to advance an important political objective?	2024 Respondents* (n = 8896)		2025 Respondents* (n = 8248)		Mean difference,* 2024–2025	
	Unweighted	Weighted % (95% CI)	Unweighted	Weighted % (95% CI)	Unweighted	Weighted mean (95% CI)
	n	*Mean score (95% CI)*	n	*Mean score (95% CI)*	n	*Mean score (95% CI)*
I will be armed with a gun						
Not likely (1)	6688	77.4 (76.2, 78.6)	6066	76.4 (75.1, 77.6)	7767	−0.7 (−2.0, 0.7)
Somewhat likely (2)	1108	11.5 (10.6, 12.4)	1092	12.0 (11.0, 12.9)	7767	0.4 (−0.8, 1.6)
Very or extremely likely (3)	954	8.8 (8.0, 9.5)	959	9.1 (8.3, 9.9)	7767	0.2 (−0.7, 1.2)
Non-response	146	2.3 (1.9, 2.8)	131	2.5 (2.0, 3.0)	7767	0.1 (−0.4, 0.6)
*Mean score†*	*8750*	*1.30 (1.28, 1.31)*	*8117*	*1.31 (1.29, 1.33)*	*7580*	*0.009 (−0.010, 0.029)*
I will carry a gun openly, so that people know I am armed						
Not likely (1)	7701	86.3 (85.2, 87.3)	7106	85.9 (84.8, 87.0)	7767	−0.2 (−1.3, 0.9)
Somewhat likely (2)	659	6.9 (6.1, 7.6)	616	7.2 (6.4, 7.9)	7767	0.1 (−0.8, 1.1)
Very or extremely likely (3)	390	4.4 (3.8, 5.1)	383	4.3 (3.7, 4.9)	7767	−0.2 (−0.9, 0.6)
Non-response	146	2.4 (1.9, 3.0)	143	2.6 (2.1, 3.2)	7767	0.2 (−0.3, 0.7)
*Mean score†*	*8750*	*1.16 (1.15, 1.18)*	*8105*	*1.16 (1.15, 1.18)*	*7570*	*−0.002 (−0.018, 0.014)*
I will threaten someone with a gun						
Not likely (1)	8498	94.0 (93.2, 94.8)	7883	93.6 (92.8, 94.5)	7767	−0.1 (−1.0, 0.7)
Somewhat likely (2)	165	2.2 (1.7, 2.7)	140	2.4 (1.9, 3.0)	7767	0.1 (−0.6, 0.8)
Very or extremely likely (3)	89	1.5 (1.1, 1.9)	87	1.5 (1.1, 1.9)	7767	0.0 (−0.6, 0.5)
Non-response	144	2.3 (1.8, 2.8)	138	2.5 (1.9, 3.0)	7767	0.1 (−0.4, 0.5)
*Mean score†*	*8752*	*1.05 (1.04, 1.06)*	*8110*	*1.06 (1.05, 1.07)*	*7574*	*−0.001 (−0.012, 0.010)*
I will shoot someone with a gun						
Not likely (1)	8372	92.9 (92.0, 93.7)	7703	92.2 (91.3, 93.0)	7767	−0.4 (−1.4, 0.5)
Somewhat likely (2)	264	3.2 (2.6, 3.7)	284	3.4 (2.8, 4.0)	7767	0.3 (−0.4, 1.0)
Very or extremely likely (3)	120	1.7 (1.3, 2.1)	126	2.0 (1.5, 2.4)	7767	0.1 (−0.5, 0.7)
Non-response	140	2.3 (1.8, 2.8)	135	2.5 (2.0, 3.0)	7767	0.0 (−0.5, 0.5)
*Mean score†*	*8756*	*1.07 (1.06, 1.08)*	*8113*	*1.07 (1.06, 1.09)*	*7579*	*0.005 (−0.008, 0.018)*

^*^ Among respondents to both surveys (n = 7767)

^†^ Mean scores in 2024 and 2025 were scored as indicated in the response lines for individual questions, with non-responses excluded. To assess cohort-level change from 2024 to 2025, we computed within-individual change scores for each item and then calculated year-to-year cohort-level change scores based on the means of aggregated within-individual change scores. Mean change scores have a range from −2 to 2 (with 0 indicating no change)

In 2025, 12.6% (95% CI, 11.5%, 13.6%) of respondents considered violence “to remove Donald Trump from the White House this year” to be usually or always justified (see Supplement, Additional file 1, Table S4). In 2024, 4.6% (95% CI, 4.0%, 5.2%) of respondents considered violence usually or always justified “to return Donald Trump to the White House this year.”

## Political party and MAGA affiliation

Categorization by political party and MAGA affiliation yielded 8 subgroups (see Supplement, Additional file 1, Table S5 for demographics). For simplicity, the following text presents results for MAGA Republicans and strong Democrats; the tables and figures present findings for all subgroups.

**Fig. 1:**
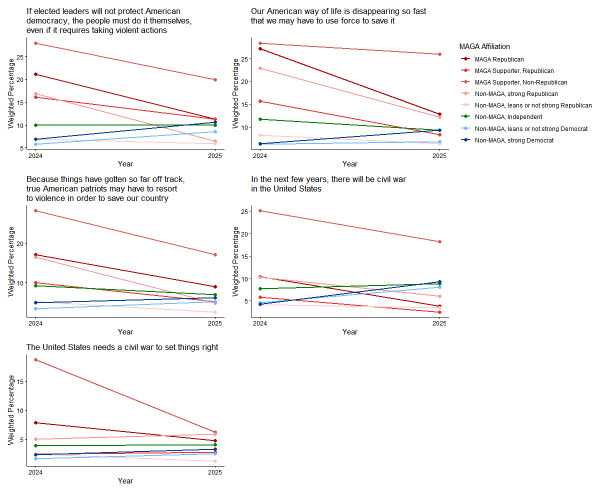
Party/MAGA affiliation and agreement* with violence to effect social change and civil war. *Weighted prevalence in 2024 and 2025 of strong or very strong agreement

**Fig. 2:**
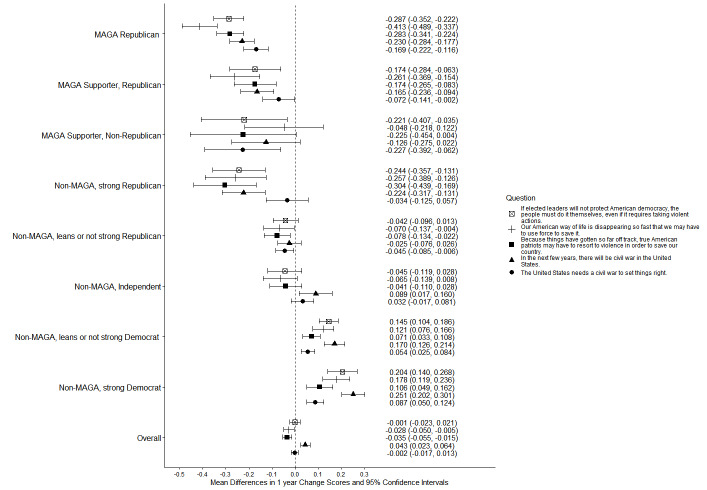
Party/MAGA affiliation and 2024–2025 change in agreement* with violence to effect social change and civil war. *Mean 2024–2025 single-year change scores

From 2024 to 2025, MAGA Republicans reported decreased agreement with all 3 statements about conditions in the US creating a potential need for political violence, with the prediction that civil war was coming, and with the assertion that it was needed (Figs. 1 and 2; see Supplement, Additional file 1, Tables S6 and S7). Strong Democrats had increased agreement with all 5 statements. In 2025, MAGA Republicans and strong Democrats did not differ significantly in agreement with statements about conditions in the US creating a potential need for political violence, the prediction that civil war was coming, or the view that it was needed (Fig. 1; see Supplement, Additional file 1, Table S6).

**Fig. 3:**
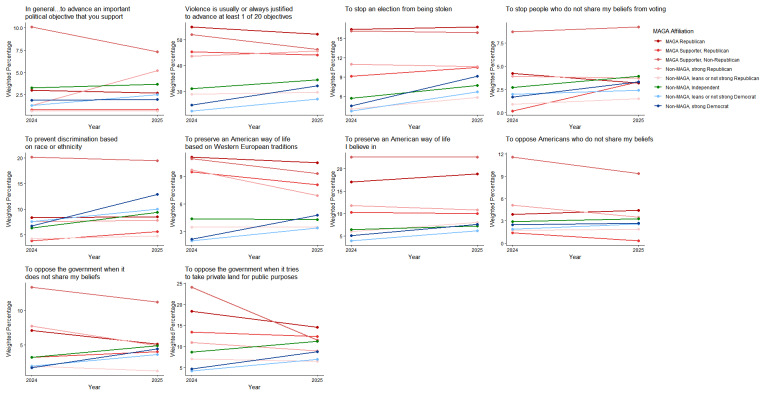
Party/MAGA affiliation and justification* for political violence. *Weighted prevalence in 2024 and 2025 of the view that political violence is usually or always justified “in general,” to advance at least 1 specific political objective, and to advance 8 objectives considered individually

**Fig. 4:**
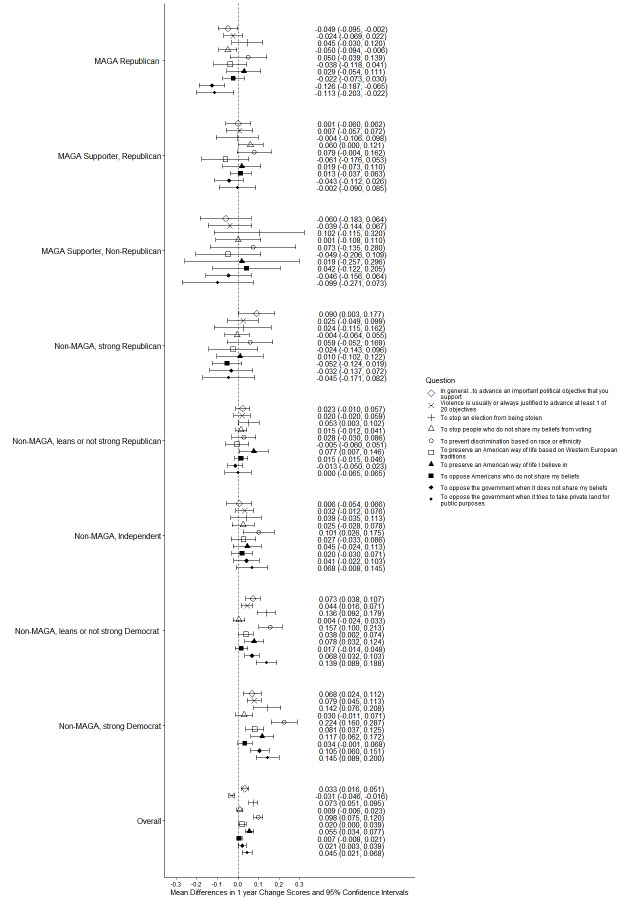
Party/MAGA affiliation and 2024–2025 change in justification* for political violence. *Mean 2024–2025 single-year change scores for justification of political violence “in general,” to advance at least 1 specific political objective, and to advance 8 objectives considered individually

**Fig. 5:**
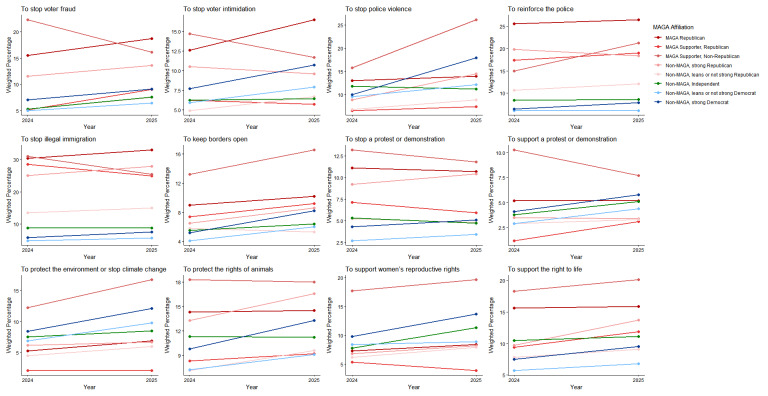
Party/MAGA affiliation and justification* for political violence to advance additional objectives. *Weighted prevalence in 2024 and 2025 of the view that political violence is usually or always justified to advance 12 additional specific political objectives

**Fig. 6:**
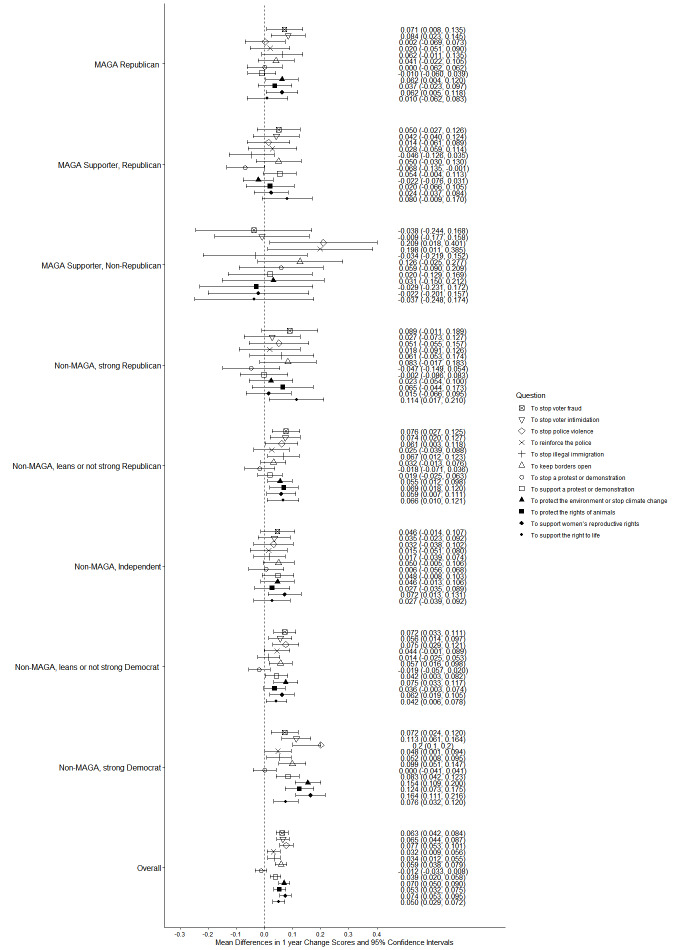
Party/MAGA affiliation and 2024–2025 change in justification* for political violence to advance additional objectives. *Mean 2024–2025 single-year change scores for justification of political violence to advance 12 additional specific political objectives

MAGA Republicans had small decreases from 2024 to 2025 in the views that political violence was justified in general or to advance at least 1 of 20 political objectives; strong Democrats had small increases (Figs. 3 and 4; see Supplement, Additional file 1, Tables S8 and S9). Among MAGA Republicans, the view that violence was justified increased for 4 of 20 individual objectives and decreased for 3 (Figs. 3, 4, 5 and 6; see Supplement, Additional file 1, Tables S8–S11); there were increases for 17 of 20 objectives among strong Democrats.

Even with these changes, MAGA Republicans were substantially more likely than strong Democrats in 2025 to consider violence usually or always justified to advance at least 1 of 20 political objectives (MAGA Republicans 52.2%, 95% CI 48.4%, 56.1%; strong Democrats 32.1%, 95% CI 28.6%, 35.6%; aPD 13.03pp, 95% CI 7.69pp, 18.36pp; q = 0.0003) and to advance 8 of 20 objectives when they were considered individually (Figs. 3 and 5; see Supplement, Additional file 1, Tables S8 and S10). Among these were “to preserve an American way of life based on Western European traditions,” “to stop a protest or demonstration,” and “to support the right to life.” Strong Democrats were not more likely than MAGA Republicans in 2025 to justify violence to advance any of the 20 objectives (Figs. 3 and 5; see Supplement, Additional file 1, Tables S8 and S10).

**Fig. 7:**
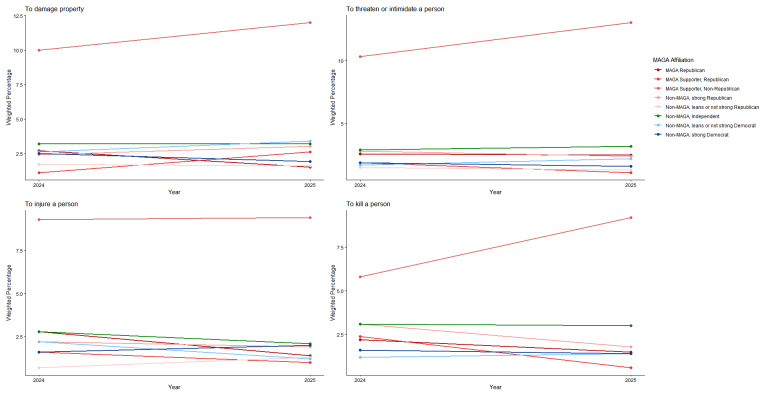
Party/MAGA affiliation and willingness* to commit political violence, by type of violence. *Weighted prevalence in 2024 and 2025 of being very willing or completely willing to commit violence

**Fig. 8:**
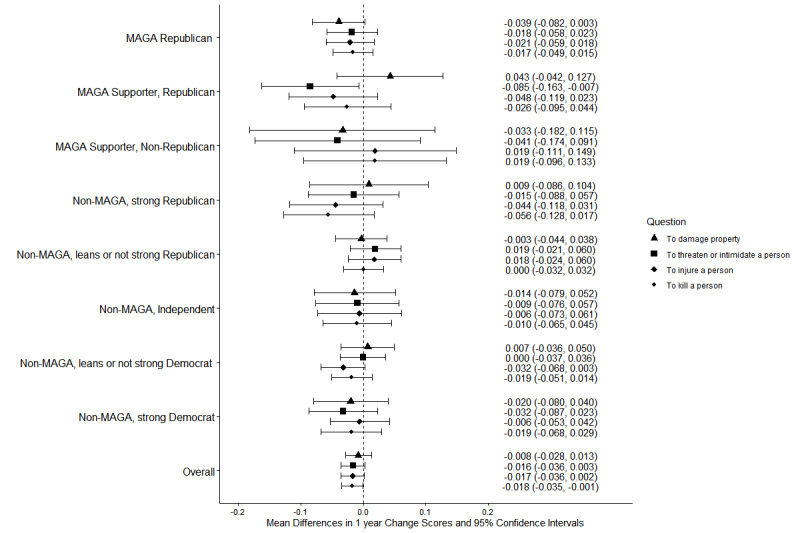
Party/MAGA affiliation and 2024–2025 change in willingness* to commit political violence, by type of violence. *Mean 2024–2025 single-year change scores for personal willingness to commit political violence

**Fig. 9:**
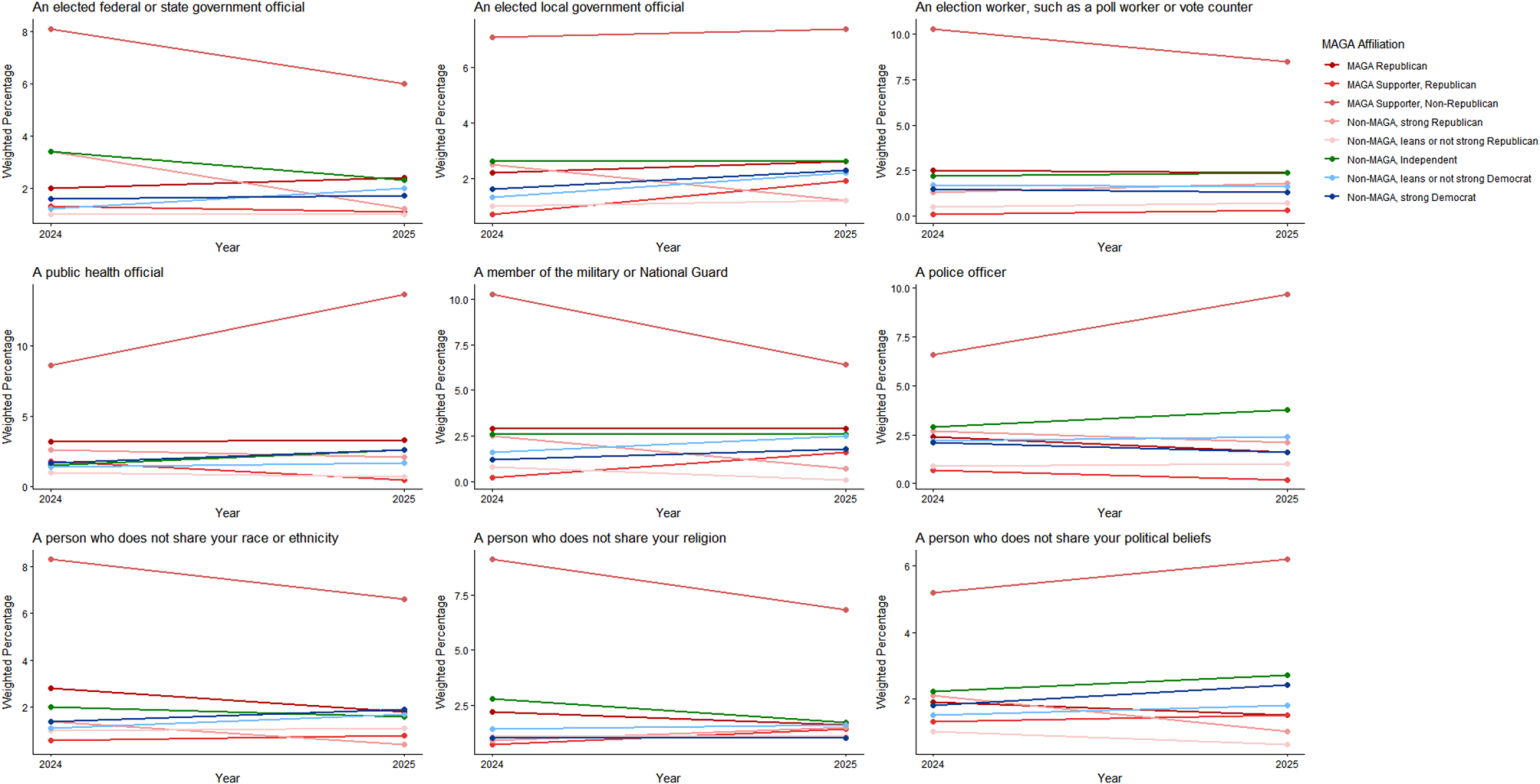
Party/MAGA affiliation and willingness* to commit political violence, by target population. *Weighted prevalence in 2024 and 2025 of being very willing or completely willing to commit violence

**Fig. 10:**
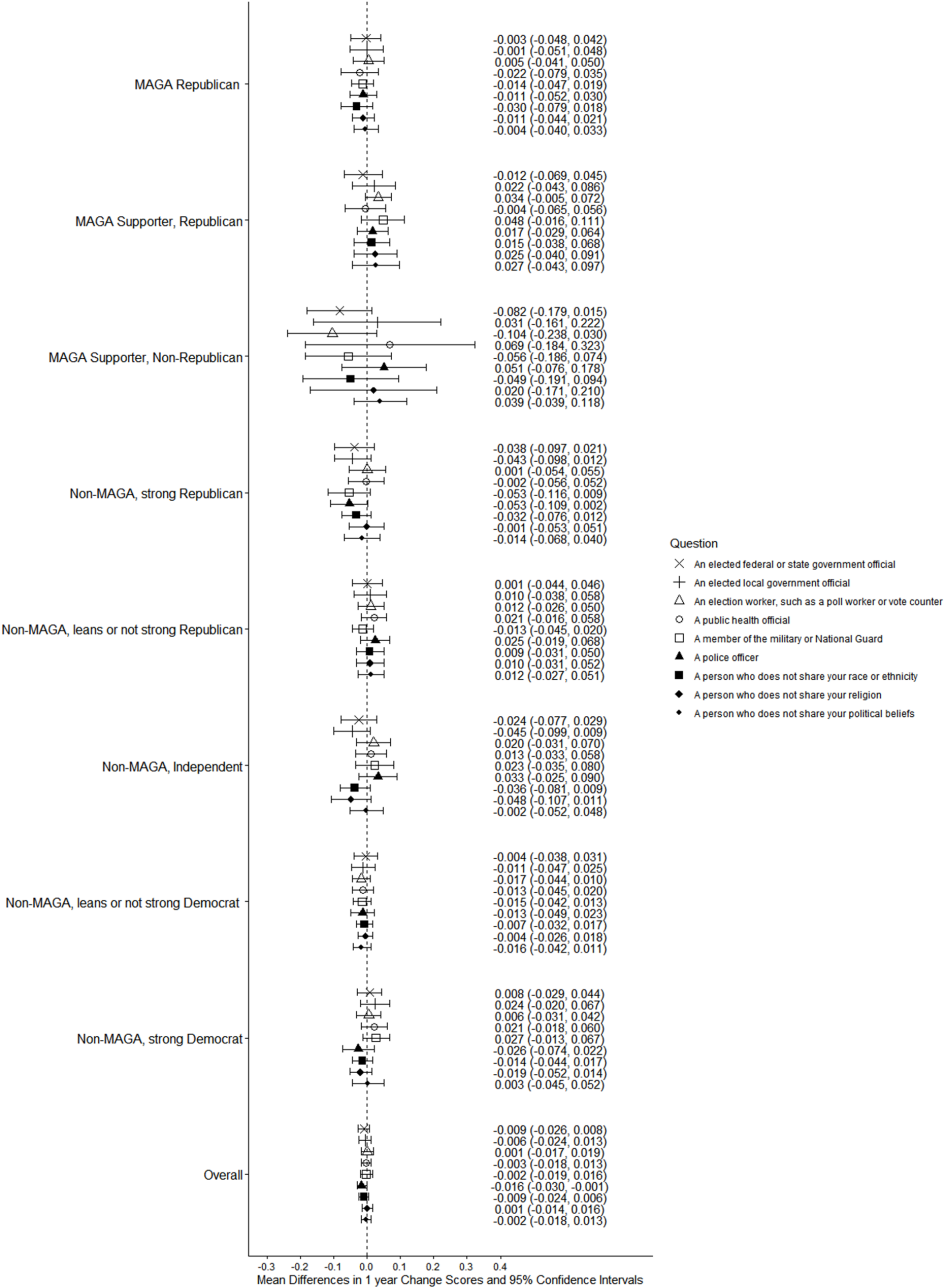
Party/MAGA affiliation and 2024–2025 change in willingness* to commit political violence, by target population. *Mean 2024–2025 single-year change scores for personal willingness to commit political violence

Both groups had non-significant decreases from 2024 to 2025 on all measures of willingness to engage in violence by type (Figs. 7 and 8; see Supplement, Additional file 1, Tables S12 and S13) and no significant changes for violence targeting specific populations (Figs. 9 and 10; see Supplement, Additional file 1, Tables S14 and S15). MAGA Republicans and strong Democrats did not differ in 2025 in their proportions that were very or completely willing to damage property, threaten a person, injure a person, or kill a person (Fig. 7; see Supplement, Additional file 1, Table S12). They also did not differ in their willingness to use violence against any target population (Fig. 9; see Supplement, Additional file 1, Table S14). Across all these measures, very or complete willingness was quite uncommon, exceeding 3% only for MAGA Republicans’ use of violence against public health officials.

**Fig. 11:**
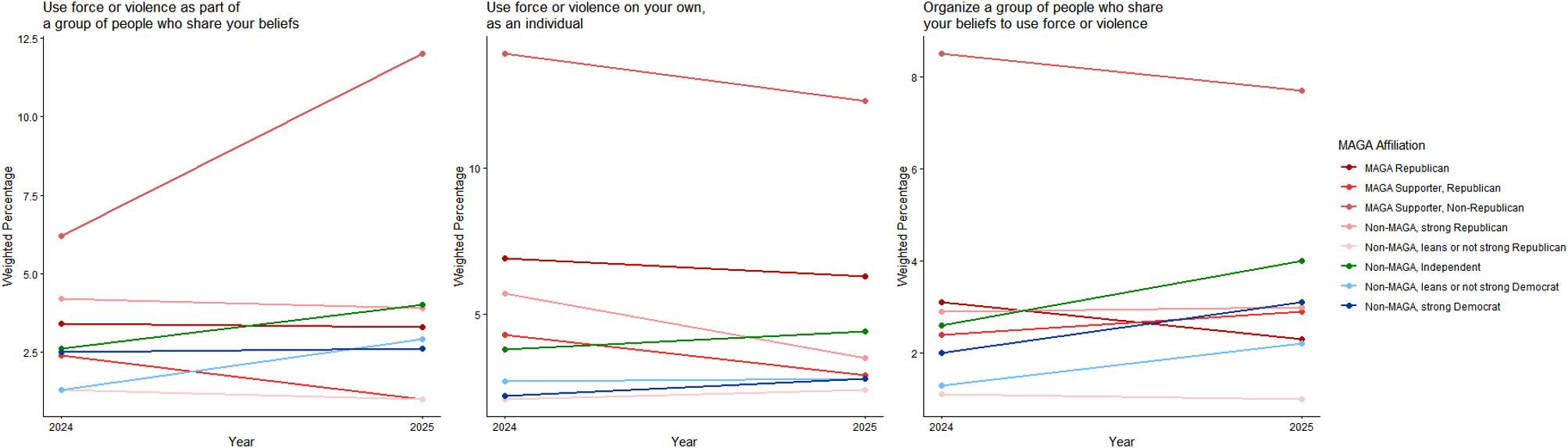
Party/MAGA affiliation and willingness* to commit political violence, by social context of violence. *Weighted prevalence in 2024 and 2025 of being very willing or completely willing to commit violence

**Fig. 12:**
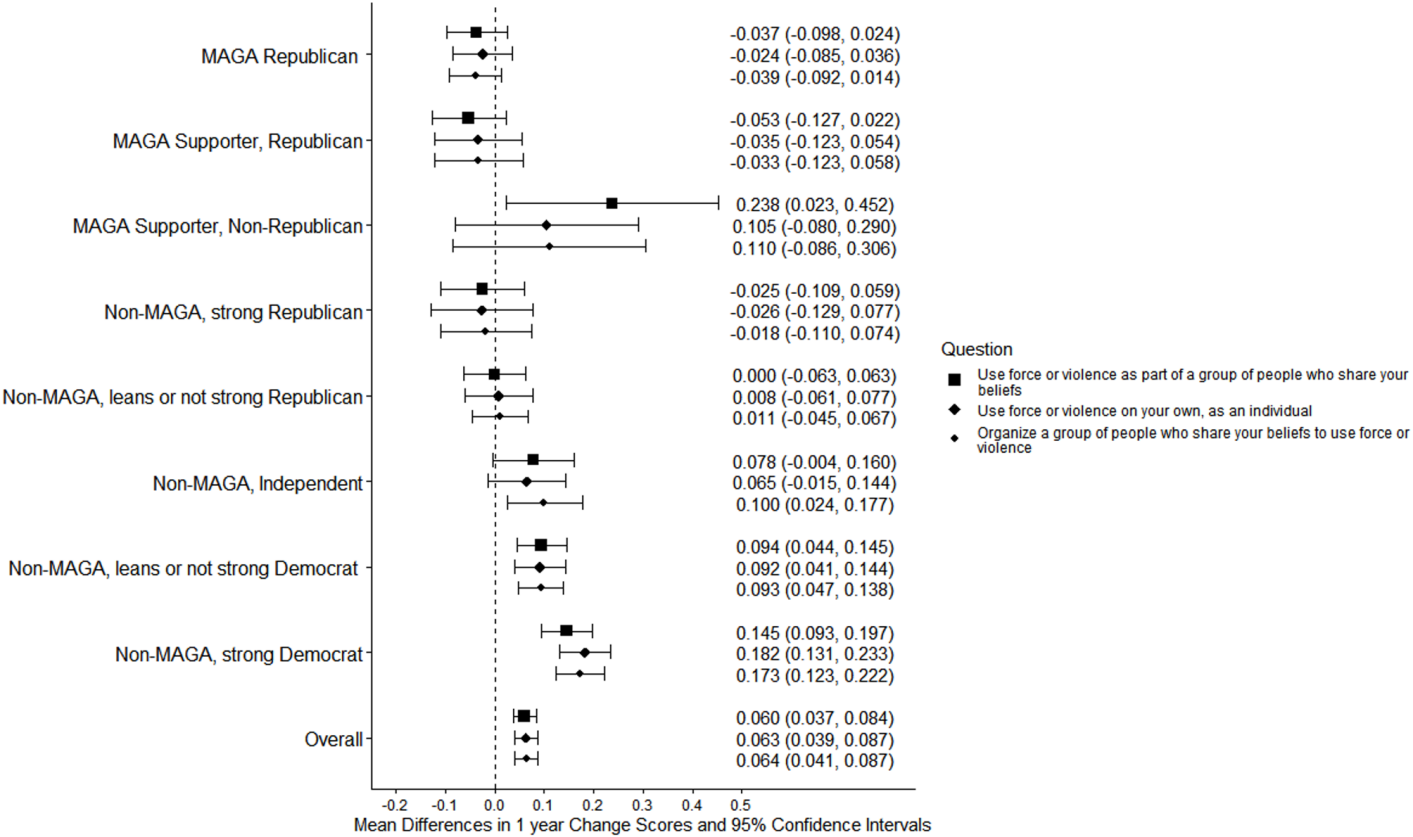
Party/MAGA affiliation and 2024–2025 change in willingness* to commit political violence, by social context of violence. *Mean 2024–2025 single-year change scores for personal willingness to commit political violence

MAGA Republicans reported no change from 2024 to 2025 in willingness to commit violence as a lone actor or with a group; strong Democrats had increases on these measures (Figs. 11 and 12; see Supplement, Additional file 1, Tables S16 and S17). MAGA Republicans nevertheless remained more likely than strong Democrats to report willingness to commit political violence as a lone actor (Fig. 11; see Supplement, Additional file 1, Table S16).

**Fig. 13:**
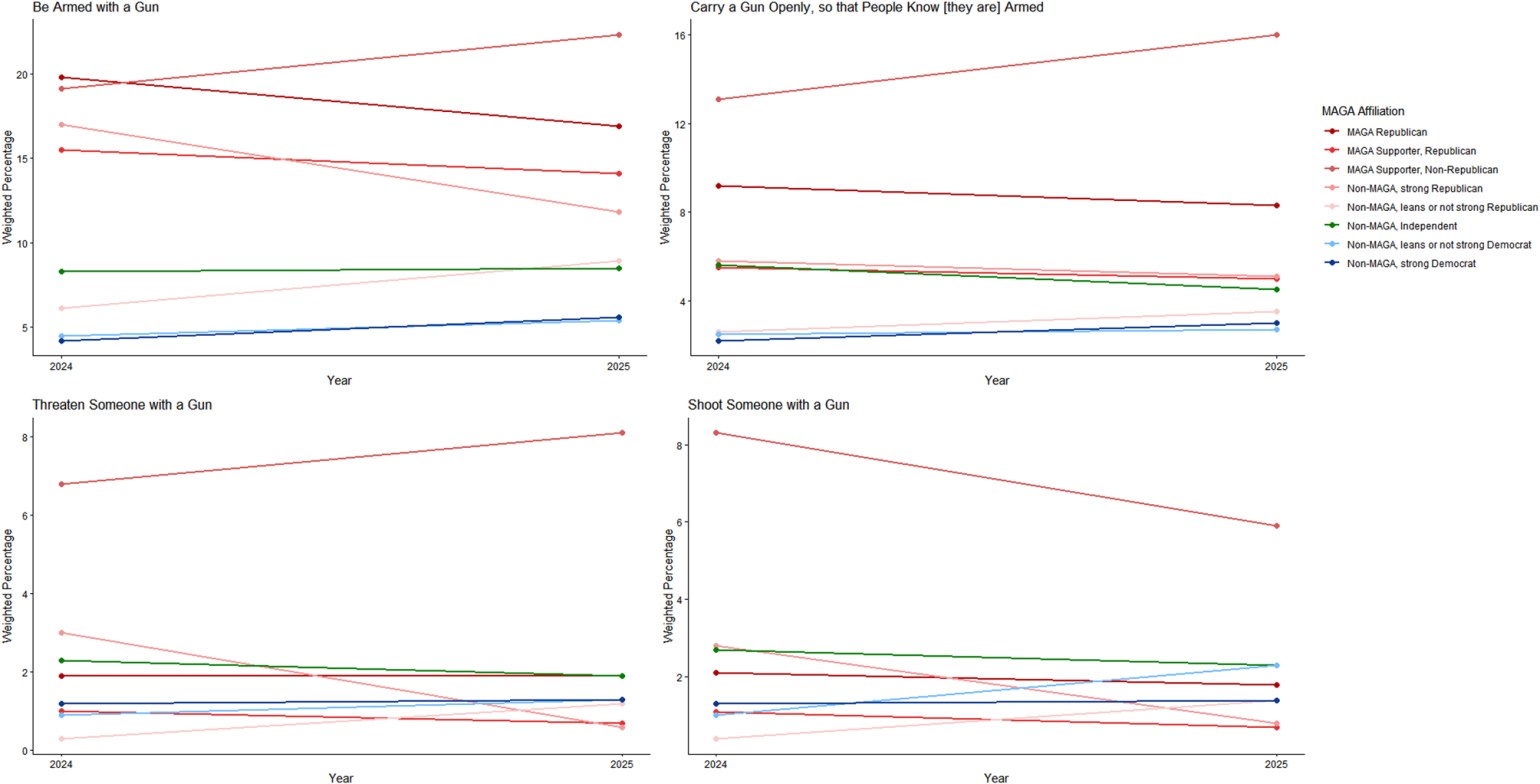
Party/MAGA affiliation and predicted firearm possession and use* in political violence. *Weighted prevalence in 2024 and 2025 of a prediction that firearm possession and use is very or extremely likely in a situation where political violence is perceived as justified

**Fig. 14:**
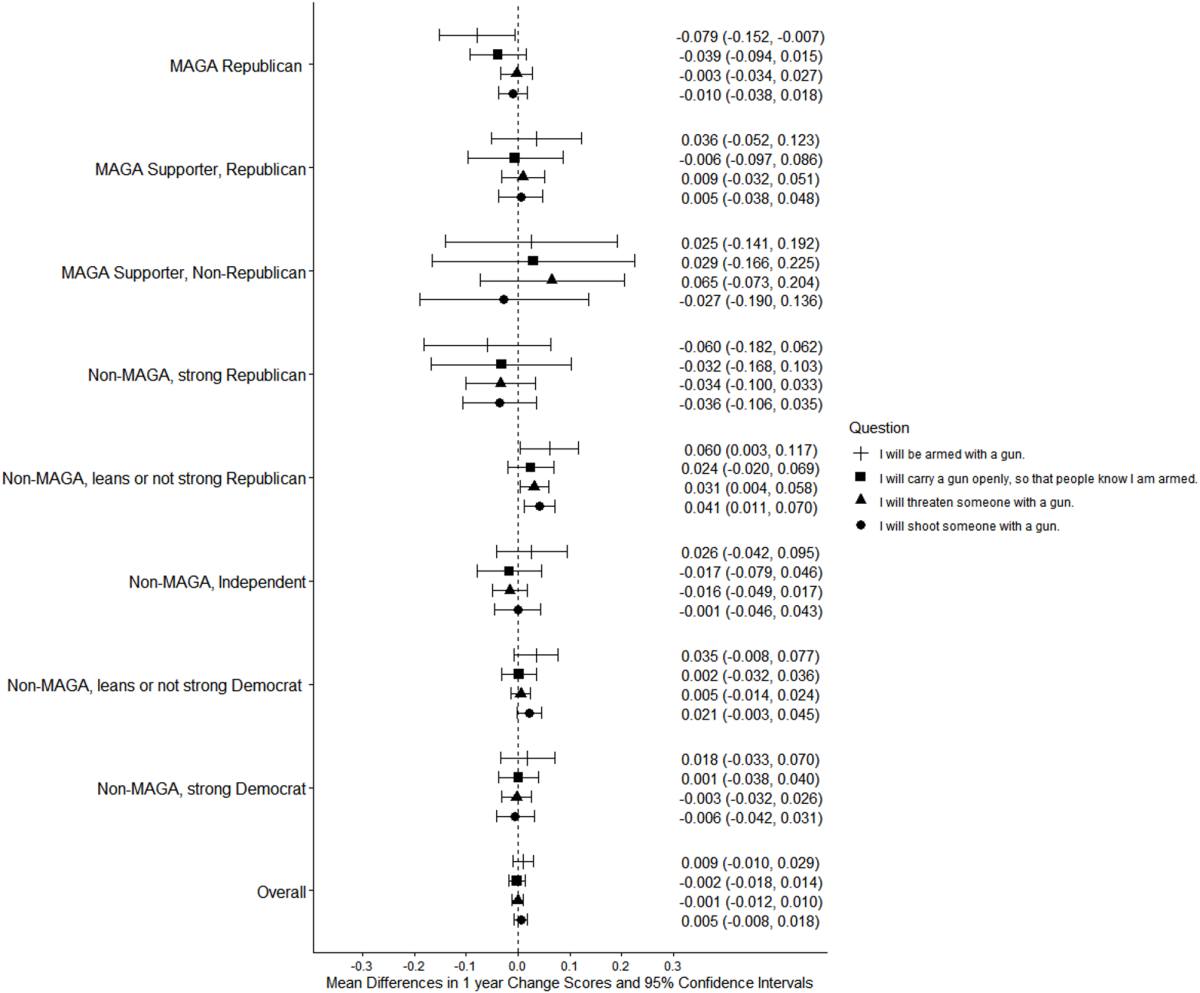
Party/MAGA affiliation and 2024–2025 change in predicted firearm possession and use* in political violence. *Mean 2024–2025 single-year change scores for a prediction that firearm possession and use is very or extremely likely in a situation where political violence is perceived as justified

MAGA Republicans were less likely in 2025 than in 2024 to predict that they would be armed in a future setting where they considered political violence to be justified; there were otherwise no changes in either group on measures of firearm possession and use (Figs. 13 and 14) (see Supplement, Additional file 1, Tables S18 and S19). MAGA Republicans remained more likely than strong Democrats to consider it very or extremely likely that they would be armed and carry a firearm openly in a setting where they considered political violence justified, but not that they would threaten or shoot someone (Fig. 13; see Supplement, Additional file 1, Table S18).

In both years, non-Republican MAGA supporters had higher prevalences than most or all other groups on a number of measures, including willingness to injure or kill someone. This is a small group (n = 197), and differences with other small groups were often not statistically significant.

**Fig. 15:**
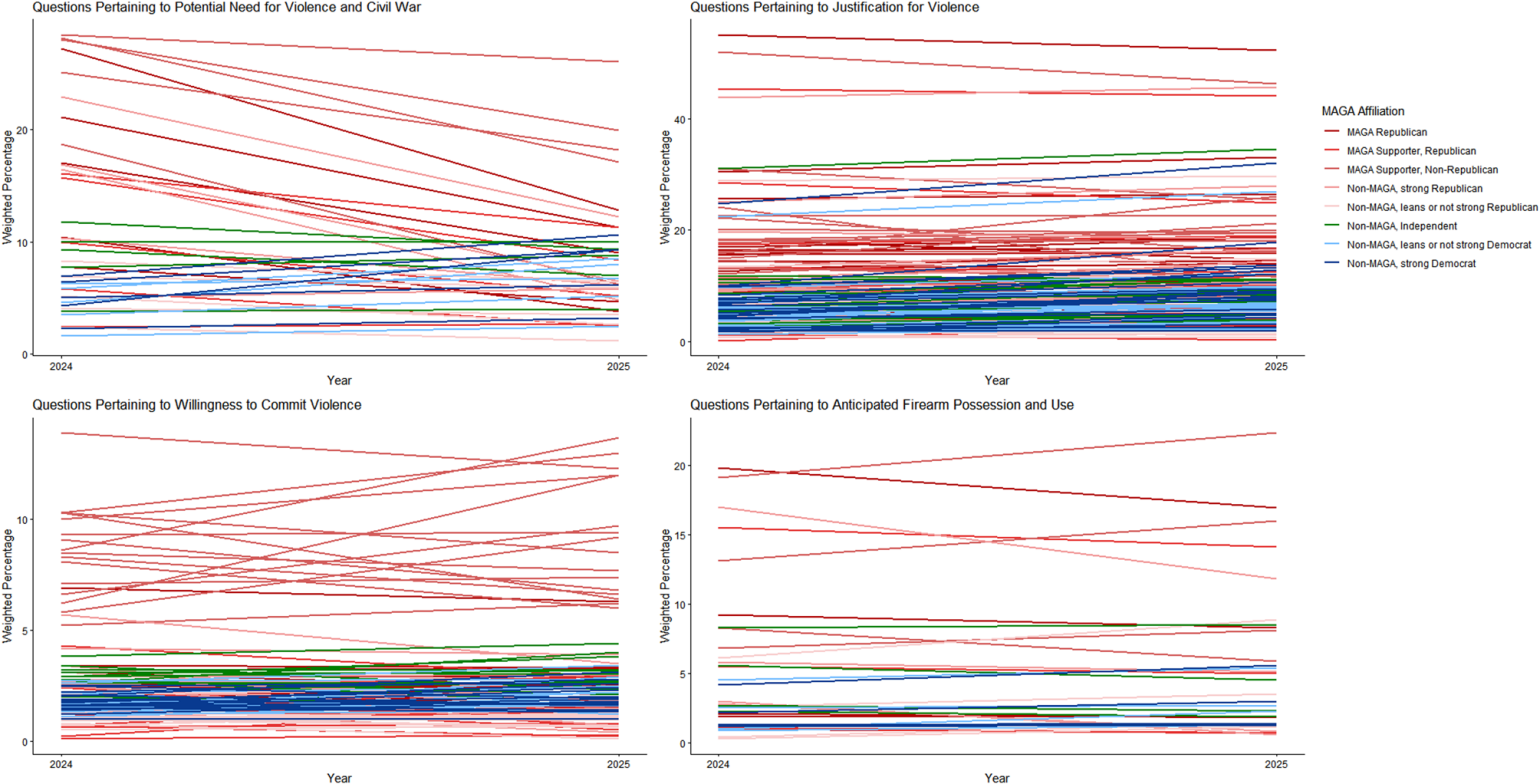
Summary of prevalence* findings for 2024 and 2025. *Prevalence of “strongly or very strongly agree” responses for questions on the potential need for violence and civil war, of “usually or always justified” responses for questions on justification for violence, of “very or completely willing” responses for questions on willingness to commit violence, and of “very or extremely likely” responses for questions on predicted firearm possession and use

Figure 15 summarizes the prevalence findings for 2024 and 2025, aggregating items by type: the potential need for violence and civil war, justification for violence, willingness to commit violence, and anticipated firearm possession and use.

## Discussion

Among the study cohort as a whole, there were decreases from 2024 to 2025 in the perception that democracy was under threat in the US, in support for expressions of authoritarianism, and in agreement with statements regarding a potential need for political violence. Increases in the view that violence was justified to advance specified political objectives were common but uniformly small, as we have defined that term. There were no changes in willingness to commit violence at varying levels of severity or against any specified target population—arguably the most concerning measures of risk for committing political violence in the survey—and there were only small increases in willingness to engage in lone actor or group violence.

These generally positive results are contrary to our a priori concern that, by different mechanisms, support for political violence in the US might have increased substantially following the 2024 elections. They are similar to findings for this cohort for 2023–2024 [8], with data tracking instances of political violence and related events in the US [53, 54], and with other surveys [2, 55, 56]. Given the role that firearms play (and would likely continue to play) in political violence in the US, and the role firearms play in violence generally [57], it is worth noting that the findings are reinforced by the decline of nationwide firearm purchasing patterns to pre-pandemic levels [58].

Furthermore, this overall stability in support for political violence did not mask major offsetting changes associated with MAGA movement and political party affiliation. While agreement with statements of the potential need for violence and possibility of civil war generally decreased for MAGA affiliates and Republicans and increased for Democrats, these changes were again small to moderate, and the decreases were often larger than the increases. A similar pattern was recently reported based on surveillance of terrorist incidents and plots [54].

Perhaps most importantly, across the spectrum of MAGA movement and political party affiliation and for the cohort as a whole, there were only small increases in justification for violence and no increases in personal willingness to commit political violence by type or against specific target populations. Not surprisingly, then, support for political violence in 2025 remained where it was in 2024: more common among MAGA affiliates than others and among Republicans than Democrats, where differences existed. As stated previously, this has been a consistent finding in our study cohort since 2022 [18, 19], and similar findings have been reported by many others [13, 20–27].

Some findings point to areas of concern. There were increases in justification for political violence to achieve some specified objectives among MAGA Republicans as well as Democrats. Non-Republicans—whether MAGA supporters or otherwise—reported increased willingness to commit group violence, and Democrats were more willing in 2025 than 2024 to engage in lone-actor violence (though MAGA Republicans remained more willing than strong Democrats). Some non-MAGA Republicans reported increased willingness to shoot someone.

But the view broadly and across time is hopeful. In each survey from 2022 to 2025, the vast majority of respondents have rejected political violence. Of the minority who considered it justifiable under specified circumstances, the vast majority were unwilling to commit political violence themselves [7, 8].

What are the implications of these findings? Entrapment in a spiral of escalating violence is not inevitable in the US. Those who promote political violence, whether in government [28–30] or not [59, 60], must recognize that—at least as of mid-2025—they do not have the public’s support. The large majority of the US population that rejects political violence must make their rejection known, creating a climate of intolerance for violence that will help prevent its occurrence. Where necessary, members of the public can intervene on an individual level with others in their families or social networks; many of those who are willing to engage in political violence are also open to suasion [9]. For those who are not, there is “if you see something, say something.” Extreme risk protection orders may have a role to play. California requires judges evaluating ERPO petitions to consider any evidence regarding “threats of violence to advance a political objective” [61]. Other states should follow suit. The media, whose crisis-oriented portrayal of political violence may be fueling the false perception that it is pervasive and increasing, must understand that their actions may help convert that false perception into reality. Fortunately, there are guidelines [62] for news coverage that documents the problem of political violence without contributing to it.

### Limitations

The survey was in the field from May 23 to June 13, 2025. There have been prominent instances of political violence since then: shootings of 2 Minnesota state legislators and their spouses on June 14 [63], conservative activist Charlie Kirk’s murder on September 10 [64], and the attack on a Mormon church in Michigan on September 28 [65]. Perhaps most important are President Trump’s and his administration’s repeated, escalating threats and acts of violence, particularly against immigrants, protesters, and persons and organizations on the political left [66–71]. The federal government is now arguably the leading instigator of political violence in the US. These developments may have affected the public’s views on political violence since our survey was in the field. The government’s actions in particular may have resulted in increased support for political violence among both its supporters and opponents [35].

It is important to note that a respondent’s support for and willingness to commit political violence do not deterministically indicate that the respondent will commit (or has committed) such violence. In a research setting, measurement artifacts can affect the magnitude of associations between attitude and behavior [48], but the existence of the association is well established [13]. Outside the research setting, of course, support for violence might fail to result in violence for a great many reasons—including, as mentioned earlier, being dissuaded by others [9].

There are several technical limitations. There are no standard definitions for changes that are “small” or “large,” and a finding of small or nonexistent cohort-level change could result from frequent—perhaps even large—but offsetting individual-level changes. The findings are subject to sampling error, inattentive or strategic responses, and nonresponse bias.

This is a longitudinal survey; respondents were ≥ 18 years old as of recruitment in 2022 but ≥ 21 years old as of 2025, and findings for those aged 18–20 in 2025 would be of interest. A few outcomes are uncommon, with weighted prevalences below 5%, which affects the precision of related estimates. The large study sample and small prevalence estimates result in relatively narrow confidence intervals in these cases. The Benjamini-Yekutieli method [51] used here for controlling the false discovery rate in aPD calculations is somewhat more conservative than the Benjamini–Hochberg method [72] used previously [17, 18]—roughly equivalent to using the Benjamini–Hochberg method with the threshold for statistical significance set at < 0.01. Comparisons to findings from earlier studies [18, 19] should be based on the aPDs and their CIs, not the q-values.

In 2024, the survey was in the field when Donald Trump was convicted on felony charges, but a sensitivity analysis found no effect of that event on support for or willingness to participate in political violence [8]. In both years, Russia’s war against Ukraine and ongoing conflict in the Middle East may have influenced responses on violence and democracy.

## Conclusions

Findings from this large, nationally representative longitudinal survey indicate that from mid-2024 to mid-2025, there was little to no change in support for or willingness to commit political violence in the US—despite the changes resulting from the 2024 elections. Support remained generally stronger among MAGA movement affiliates and Republicans than among Democrats. It remains to be seen whether this stability will persist.

## Supplementary Information

Supplementary Material 1

## Data Availability

The datasets generated and/or analyzed during the current study are not publicly available as analyses are continuing but will be made available to qualified researchers subject to the terms of a data use agreement.

## Abbreviations

aPD: Adjusted prevalence difference
CI: Confidence interval
PP: Percentage point
SD: Standard deviation
