# Supplementary material for: Views on democracy and political violence in the United States in 2025: findings from a nationally representative survey
Source: Inj Epidemiol. 2026 May 27;13:43. doi: 10.1186/s40621-026-00684-3 (PMC13214363; doi:10.1186/s40621-026-00684-3)
Supplement: Supplementary file 1 — Supplementary Material 1 [file 40621_2026_684_MOESM1_ESM.pdf]

Supplement

Views on Democracy and Political Violence in the United States in 2025: Findings from a Nationally Representative Survey

Garen J. Wintemute, MD, MPH; Sonia L. Robinson, PhD; Andrew Crawford, PhD;
Julia P. Schleimer, PhD, MPH; Daniel J. Tancredi, PhD; Aaron B. Shev, PhD; Elizabeth A. Tomsich, PhD; Mona A. Wright, MPH; Bradley Velasquez, MS; Shaina Sta. Cruz, PhD, MSPH; Veronica A. Pear, PhD, MPH, MA

This supplement has been provided by the authors to give readers additional information about the work.

Page	Title
3	Questions that supplied data for this study
9	References for the question list
10	Additional methods text
13	Additional results text
14	Figure S1. Flowchart of survey assignment and completion counts for Waves 1-4 in 2022-2025
15	Table S1. Sociodemographic characteristics of respondents in 2024 and 2025
17	Table S2. Sociodemographic characteristics of respondents and non-respondents (unweighted) in 2024 and 2025
19	Table S3. Views on democracy and authoritarianism
21	Table S4. Views on returning Donald Trump to the presidency (2024) and removing him from it (2025)
22	Table S5. Sociodemographic characteristics of respondents by party/MAGA affiliation
26	Table S6. Party/MAGA affiliation and 2025 prevalence of beliefs concerning violence to effect social change and civil war
29	Table S7. Party/MAGA affiliation and 2024-2025 change in beliefs concerning violence to effect social change and civil war
31	Table S8. Party/MAGA affiliation and 2025 prevalence of justification for political violence “in general” and to advance specific political objectives
36	Table S9. Party/MAGA affiliation and 2024-2025 change in justification for political violence “in general” and to advance specific political objectives
40	Table S10. Party/MAGA affiliation and 2025 prevalence of justification for violence to advance additional specific political objectives
45	Table S11. Party/MAGA affiliation and 2024-2025 change in justification for violence to advance additional specific political objectives
50	Table S12. Party/MAGA affiliation and 2025 prevalence of personal willingness to commit political violence, by type of violence

Page	Title
53	Table S13. Party/MAGA affiliation and 2024-2025 change in personal willingness to commit political violence, by type of violence
56	Table S14. Party/MAGA affiliation and 2025 prevalence of personal willingness to engage in political violence, by target population
61	Table S15. Party/MAGA affiliation and 2024-2025 change in personal willingness to engage in political violence, by target population
66	Table S16. Party/MAGA affiliation and 2025 prevalence of personal willingness to commit political violence, by social context of violence
69	Table S17. Party/MAGA affiliation and 2024-2025 change in personal willingness to commit political violence, by social context of violence
71	Table S18. Party/MAGA affiliation and 2025 prevalence of future firearm possession and use when political violence is perceived as justified
74	Table S19. Party/MAGA affiliation and 2024-2025 change in future firearm possession and use when political violence is perceived as justified

QUESTIONS THAT SUPPLIED DATA FOR THIS STUDY
Response options are presented here in order from negative to positive (e.g., "not important" to "extremely important"). Respondents were randomized 1:1 to receive responses in that order or the reverse.

In the list below, questions or items that were repeated or adapted from prior surveys by other investigators contain citations to those surveys.

Party affiliation and partisanship

Q: Generally speaking, do you think of yourself as...Select one answer only.

1. Republican
2. Democrat
3. Independent
6. Something else

(Asked if Republican)
Q: Would you call yourself a...Select one answer only.

1. Strong Republican
2. Not very strong Republican

(Asked if Democrat)
Q: Would you call yourself a...Select one answer only.

1. Strong Democrat
2. Not very strong Democrat

(Asked if Independent or Something else)
Q: Do you think of yourself as closer to the...Select one answer only.

1. Republican Party
2. Democratic Party
3. Do not lean either way

(Asked if Republican OR Leans Republican)
Q: Do you think of yourself as a MAGA Republican?

1. No
2. Yes

(Asked if Not MAGA Republican OR Democrat / Leans Democrat OR Does not lean either way)
Q: Do you think of yourself as a supporter of the MAGA movement?

1. No
2. Yes

Domain 1: democracy in the United States
Now we'd like to ask you a few questions about the United States as you see it now, in 2024.

Q: When thinking about democracy in the United States these days, do you believe…?[1]

1. There is a serious threat to our democracy.
2. There may be a threat to our democracy, but it is not serious.
3. There is no threat to our democracy.

Q: How important do you think it is for the United States to remain a democracy?[2]

1. Not important
2. Somewhat important
3. Very important
4. Extremely important

Q: How much do you agree or disagree with the following statements about democracy in the United States?

a. Democracy is the best form of government.[3]
b. These days, American democracy only serves the interests of the wealthy and powerful.[4]
c. Having a strong leader for America is more important than having a democracy.
d. The 2020 election was stolen from Donald Trump, and Joe Biden is an illegitimate president.
e. We should suspend Congress for a few years so a strong leader can clean up the mess made by politicians in Washington.[5]

1. Do not agree
2. Somewhat agree
3. Strongly agree
4. Very strongly agree

Q: Which is more important to you…?[6]

a. Having election outcomes determined democratically
b. Having political leaders I can trust to look out for my values and interests

Domain 2: violence, including political violence

Q: People have different views about violence in the United States. How much do you agree or disagree with each of the following statements?

a. If elected leaders will not protect American democracy, the people must do it themselves, even if it requires taking violent actions.[4]
b. Our American way of life is disappearing so fast that we may have to use force to save it.[4]
c. Because things have gotten so far off track, true American patriots may have to resort to violence in order to save our country.[7]

1. Do not agree
2. Somewhat agree
3. Strongly agree
4. Very strongly agree

Q: How much do you agree or disagree with each of the following statements?

a. In the next few years, there will be civil war in the United States.[10]
b. The United States needs a civil war to set things right.

1. Do not agree
2. Somewhat agree
3. Strongly agree
4. Very strongly agree

Now we have a few questions about the use of force or violence. "Force or violence" means physical force strong enough that it could cause pain or injury to a person. A reminder: your responses will be kept confidential and anonymous.

Q: People sometimes talk about using force or violence to achieve political objectives. In general, what do you think about using force or violence to advance an important political objective that you support—is it…?

1. Never justified
2. Sometimes justified
3. Usually justified
4. Always justified

Q: Your view of the use of force or violence to advance an important political objective might depend on the specific objective that was involved. What do you think about the use of force or violence in the following situations—is it never justified, sometimes justified, usually justified, or always justified?

a. (2025) To remove Donald Trump from the presidency this year (2024: To return Donald Trump to the presidency this year)
b. To stop an election from being stolen
c. To stop people who do not share my beliefs from voting

d. To prevent discrimination based on race or ethnicity
e. To preserve an American way of life based on Western European traditions
f. To oppose the government when it does not share my beliefs
g. To oppose the government when it tries to take private land for public purposes

1. Never justified
2. Sometimes justified
3. Usually justified
4. Always justified

Q: You said that in general, the use of force or violence was [prior response inserted] to advance an important political objective that you support. Your opinion might depend on the specific objective that was involved. What do you think about the use of force or violence in the following situations—is it never justified, sometimes justified, usually justified, or always justified?

a. To stop voter fraud
b. To stop voter intimidation
c. To stop police violence
d. To reinforce the police
e. To stop illegal immigration
f. To keep our borders open
g. To stop a protest or demonstration
h. To support a protest or demonstration
i. To preserve the American way of life l believe in
j. To oppose Americans who do not share my beliefs

1. Never justified
2. Sometimes justified
3. Usually justified
4. Always justified

Q: Here's another group of political objectives. What do you think about the use of force or violence in the following situations—is it never justified, sometimes justified, usually justified, or always justified?

a. To protect the environment or stop climate change
b. To protect the rights of animals
c. To support women's reproductive rights
d. To support the right to life

1. Never justified
2. Sometimes justified
3. Usually justified

4. Always justified

(Questions asked of respondents who reported that at least 1 use of violence to achieve a specific political objective was at least somewhat justified.)

Q: In a situation where you think force or violence is justified to advance an important political objective, how willing would you personally be to use force or violence in each of these ways?

a. To damage property
b. To threaten or intimidate a person
c. To injure a person
d. To kill a person

1. Not willing
2. Somewhat willing
3. Very willing
4. Completely willing

Q: In a situation where you think force or violence is justified to advance an important political objective, how willing would you personally be to use force or violence against a person because they are…

a. An elected federal or state government official
b. An elected local government official
c. A public health official
d. A member of the military or National Guard
e. A police officer
f. A person who does not share your race or ethnicity
g. A person who does not share your religion
h. An election worker, such as a poll worker or vote counter
i. A person who does not share your political beliefs

1. Not willing
2. Somewhat willing
3. Very willing
4. Completely willing

Q: You agreed that the use of force or violence could be justified to advance [one/some] of the political objectives we just discussed. In [that/those] [situation/situations], how willing would you personally be to…

a. Use force or violence as part of a group of people who share your beliefs
b. Use force or violence on your own, as an individual
c. Organize a group of people who share your beliefs to use force or violence

1. Not willing

2. Somewhat willing
3. Very willing
4. Completely willing

(Question asked of all respondents.)
Q: Thinking now about the future and all the changes it might bring, how likely is it that you will use a gun in any of the following ways in the next few years—in a situation where you think force or violence is justified to advance an important political objective?
a. I will be armed with a gun.
b. I will carry a gun openly, so that people know I am armed.
c. I will threaten someone with a gun.
d. I will shoot someone with a gun.

1. Not likely
2. Somewhat likely
3. Very likely
4. Extremely likely

REFERENCES FOR THE QUESTION LIST

1. NPR/PBS NewsHour/Marist National Poll. Trust in elections, threat to democracy, November 2021. 2021 November 1. https://maristpoll.marist.edu/polls/npr-pbs-newshour-marist-national-poll-trust-in-elections-threat-to-democracy-biden-approval-november-2021/.
2. Grinnell College National Poll. 52% of Americans believe democracy facing "major threat." Study #2243. 2021 October 20. https://www.grinnell.edu/news/52-americans-believe-democracy-facing-major-threat.
3. The Economist/YouGov Poll. 2021 June 13-16. https://docs.cdn.yougov.com/uagnfc262c/econTabReport.pdf.
4. Survey Center on American Life. January 2021 American Perspectives Survey topline questionnaire. https://www.americansurveycenter.org/wp-content/uploads/2021/03/January-2021-APS-Topline-Questionnaire.pdf.
5. Democracy Fund Voter Study Group. Guide to the views of the Electorate Research Survey. 2021 December. https://www.voterstudygroup.org/data/voter-survey.
6. Hart Research Associates. The New Republic Democracy Survey (Study #14230). 2022 March. https://newrepublic.com/article/166027/democracy-poll.
7. Public Religion Research Institute. The persistence of Q-Anon in the post-Trump era: an analysis of who believes the conspiracies. 2022 Feb 24. https://www.prri.org/research/the-persistence-of-qanon-in-the-post-trump-era-an-analysis-of-who-believes-the-conspiracies/.
8. Pew Research Center. Americans see advantages and challenges in country's growing racial and ethnic diversity. 2019 May. https://www.pewresearch.org/social-trends/2019/05/08/americans-see-advantages-and-challenges-in-countrys-growing-racial-and-ethnic-diversity/.
9. IFYC – PRRI Survey on Religion & COVID-19 Vaccine Trust. 2021 March. https://www.prri.org/wp-content/uploads/2021/05/Topline-IFYC-PRRI-Survey-on-Religion-and-COVID-19-Vaccine-Trust-v2_final.pdf.
10. Zogby. Will the US have another civil war? 2021 Feb 4. https://zogbyanalytics.com/news/997-the-zogby-poll-will-the-us-have-another-civil-war

ADDITIONAL METHODS TEXT

Participants

Details of the survey assignment and completion counts for Waves 1-4 are presented in Figure S1. Of 12,947 Wave 1 respondents, 11,140 (86.0%) remained active members of KnowledgePanel on Wave 2's launch date and were invited to participate in Wave 2. (The remaining 1807 Wave 1 respondents had left the cohort through normal attrition.)

Wave 2 had 9385 respondents (completion rate of 84.2%), of whom 8,932 (95.2%) remained active members of KnowledgePanel on Wave 3's launch date and were invited to participate in Wave 3. (Another 453 Wave 2 respondents had left the cohort through normal attrition.)

Invitations to participate in Wave 3 were also sent to 1132 Wave 1 respondents who had not participated in Wave 2 and remained active members of KnowledgePanel on Wave 3's launch date. (Another 716 Wave 1 respondents who did not respond to Wave 2 had left the cohort through normal attrition.)

Wave 3 had 8896 respondents (completion rate of 88.4%), of whom 8428 (94.7%) remained active members of KnowledgePanel on Wave 4's launch date and were invited to participate in Wave 4. (Another 468 Wave 3 respondents had left the cohort through normal attrition.)

Invitations to participate in Wave 4 were also sent to 751 Wave 1 respondents who had not participated in Wave 3 (or, in some cases, Wave 2) and remained active members of KnowledgePanel on Wave 4's launch date.

Measures

Party affiliation and support for the Make America Great Again (MAGA) movement were determined as follows. Participants were asked, "Generally speaking, do you think of yourself as..." with response options "Republican," "Democrat," "independent," and "something else." Those who chose Republican or Democrat were asked, "Would you call yourself a…" with response options "strong Republican" and "not very strong Republican" or "strong Democrat" and "not very strong Democrat." Those who chose "independent" or "something else" for the first question were asked, "Do you think of yourself as closer to the…" with response options "Republican Party," "Democratic Party," and "do not lean either way." Respondents who identified as Republican or closer to the Republican Party were asked, "Do you think of yourself as a MAGA Republican?" with response options "yes" and "no." Those who responded no, along with those who identified as Democrat, closer to the Democratic Party, or not leaning either way were asked, "Do you think of yourself as a supporter of the MAGA movement?" with response options "yes" and "no."

Respondents were then classified as MAGA Republicans, Republican MAGA supporters, non-Republican MAGA supporters, non-MAGA strong Republicans, non-MAGA leans/not strong Republicans, non-MAGA Independents, non-MAGA leans/not strong Democrats, and non-MAGA strong Democrats.

Statistical analysis

The following variables were included in the regression models for adjusted prevalence differences and were specified as noted in parentheses: age (continuous), gender (male,

female, other), race and ethnicity (non-Hispanic white, non-Hispanic Black, Hispanic, Asian American or Pacific Islander, and other), education (less than high school, high school, some college/associate degree, bachelor's degree, master's degree or higher), income (<$10,000, $10,000-$24,999, $25,000-$49,999, $50,000-$74,999, $75,000-$99,999, $100,000-$149,999, >$150,000), Census division (New England, Mid-Atlantic, East-North Central, West-North Central, South Atlantic, East-South Central, West-South Central, Mountain, Pacific), marital status (now married; widowed, divorced or separated; never married), home ownership (yes vs no), living in an urban census tract (yes vs no), firearm ownership in 2024 (firearm owner, non-owner living in a household with a firearm owner, other non-owner), total drinks per week (continuous), military service (yes vs no), and history of a non-traffic related arrest (yes vs no).

ADDITIONAL RESULTS TEXT

One item in the 2025 survey that was included in this analysis had a nonresponse percentage above 3.0%:

Q: Which is more important to you…?

Having election outcomes determined democratically

OR

Having political leaders I can trust

Nonresponse was 3.4% in 2025, 3.2% in 2024.

Figure S1. Flowchart of survey assignment and completion counts for Waves 1-4 in 2022-2025

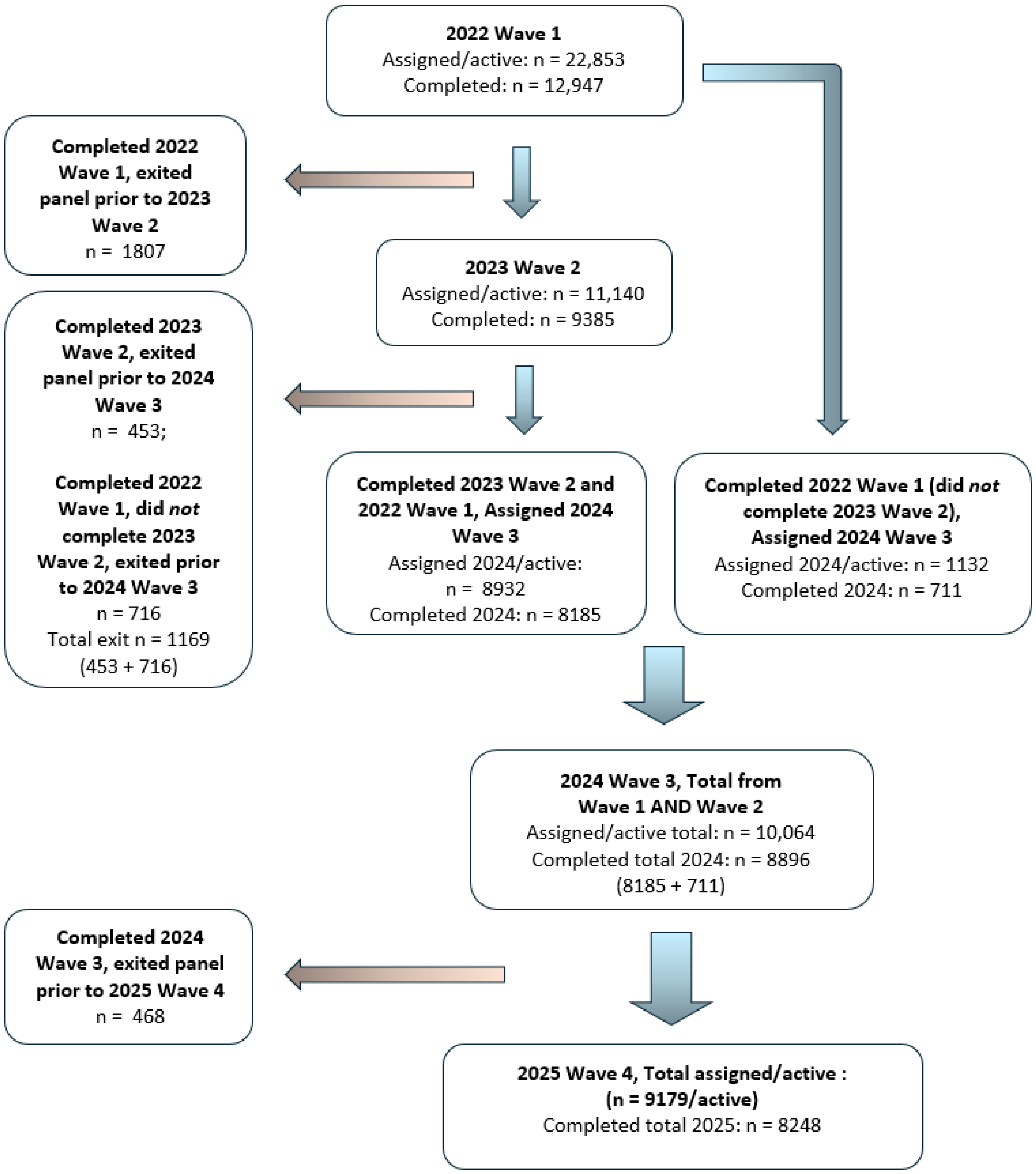

Table S1. Sociodemographic characteristics of respondents in 2024 and 2025

Characteristic	2024 Respondents* (n=8896)		2025 Respondents* (n=8248)	
	Unweighted n	Weighted % (95% CI)	Unweighted n	Weighted % (95% CI)
Age				
18-24	298	10.4 (9.2, 11.5)	278	10.5 (9.2, 11.7)
25-34	808	16.6 (15.4, 17.8)	739	16.3 (15.1, 17.6)
35-44	1208	18.6 (17.5, 19.8)	1126	18.7 (17.6, 19.9)
45-54	1173	13.9 (13.0, 14.8)	1098	13.9 (12.9, 14.8)
55-64	1969	17.8 (16.9, 18.8)	1864	18.0 (17.0, 19.0)
65-74	2219	14.6 (13.9, 15.4)	2064	15.0 (14.1, 15.8)
75+	1221	8.1 (7.5, 8.6)	1079	7.7 (7.0, 8.3)
Non-response	0	0.0 (0.0, 0.0)	0	0.0 (0.0, 0.0)
Gender				
Female	3667	50.6 (49.2, 52.0)	3390	50.7 (49.2, 52.2)
Male	5055	47.2 (45.8, 48.6)	4697	47.1 (45.6, 48.5)
Transgender	46	0.5 (0.3, 0.7)	42	0.5 (0.3, 0.6)
Non-binary	58	0.8 (0.5, 1.0)	55	0.8 (0.5, 1.1)
Other	20	0.3 (0.1, 0.5)	17	0.3 (0.1, 0.4)
Non-response	50	0.7 (0.4, 0.9)	47	0.7 (0.4, 1.0)
Race/Ethnicity				
White, Non-Hispanic	6663	62.7 (61.3, 64.2)	6154	62.7 (61.2, 64.3)
Black, Non-Hispanic	720	12.0 (10.9, 13.0)	683	12.0 (10.9, 13.1)
Hispanic, any race	940	16.9 (15.7, 18.1)	870	16.9 (15.6, 18.2)
American Indian or Alaska Native, Non-Hispanic	44	1.1 (0.7, 1.5)	44	1.1 (0.7, 1.6)
Asian American or Pacific Islander, non-Hispanic	261	5.4 (4.7, 6.2)	241	5.4 (4.6, 6.2)
Some other race, Non-Hispanic	18	0.1 (0.1, 0.2)	16	0.1 (0.0, 0.2)
2+ Races, Non-Hispanic	250	1.8 (1.4, 2.1)	240	1.8 (1.4, 2.2)
Non-response	0	0.0 (0.0, 0.0)	0	0.0 (0.0, 0.0)
Marital status				
Now married	5677	56.6 (55.2, 58.1)	5285	56.8 (55.3, 58.3)
Widowed	530	3.9 (3.5, 4.4)	477	3.8 (3.4, 4.3)
Divorced	963	8.2 (7.5, 8.8)	878	8.1 (7.4, 8.7)
Separated	122	1.5 (1.2, 1.9)	113	1.5 (1.1, 1.8)
Never married	1604	29.7 (28.3, 31.2)	1495	29.9 (28.4, 31.4)
Non-response	0	0.0 (0.0, 0.0)	0	0.0 (0.0, 0.0)
Education				
No high school diploma or GED	381	9.5 (8.4, 10.5)	342	9.3 (8.2, 10.4)
High school graduate (diploma, GED)	1856	28.2 (26.8, 29.6)	1716	28.3 (26.9, 29.7)
Some college or Associate's degree	2633	27.1 (25.9, 28.4)	2450	27.2 (25.9, 28.4)
Bachelor's degree	2244	20.1 (19.1, 21.1)	2080	19.9 (18.9, 21.0)
Master’s degree or higher	1782	15.1 (14.3, 16.0)	1660	15.3 (14.4, 16.2)
Non-response	0	0.0 (0.0, 0.0)	0	0.0 (0.0, 0.0)

Table S1, continued.

Characteristic	2024 Respondents* (n=8896)		2025 Respondents* (n=8248)	
	Unweighted n	Weighted % (95% CI)	Unweighted n	Weighted % (95% CI)
Household Income				
Less than $10,000	225	4.0 (3.3, 4.6)	205	4.0 (3.3, 4.7)
$10,000 to $24,999	646	8.8 (7.9, 9.7)	587	8.8 (7.9, 9.7)
$25,000 to $49,999	1493	16.9 (15.8, 18.0)	1372	16.9 (15.8, 18.0)
$50,000 to $74,999	1510	16.3 (15.2, 17.4)	1402	16.3 (15.2, 17.4)
$75,000 to $99,999	1439	13.2 (12.3, 14.1)	1326	13.2 (12.3, 14.1)
$100,000 to $149,999	1688	17.9 (16.9, 19.0)	1562	17.9 (16.8, 19.0)
$150,000 or more	1895	22.8 (21.7, 24.0)	1794	22.8 (21.6, 24.0)
Non-response	0	0.0 (0.0, 0.0)	0	0.0 (0.0, 0.0)
Employment				
Working - as a paid employee	4134	53.2 (51.8, 54.7)	3902	53.6 (52.1, 55.0)
Working - self-employed	669	7.2 (6.5, 8.0)	606	7.1 (6.4, 7.9)
Not working - on temporary layoff from a job	37	0.6 (0.4, 0.9)	32	0.5 (0.3, 0.8)
Not working - looking for work	251	4.9 (4.2, 5.7)	228	5.0 (4.2, 5.8)
Not working - retired	3154	21.4 (20.5, 22.4)	2877	21.3 (20.3, 22.2)
Not working - disabled	258	4.4 (3.7, 5.0)	237	4.4 (3.7, 5.1)
Not working - other	393	8.2 (7.3, 9.1)	366	8.1 (7.2, 9.1)
Non-response	0	0.0 (0.0, 0.0)	0	0.0 (0.0, 0.0)
Census division				
New England	362	4.6 (4.0, 5.2)	340	4.7 (4.0, 5.3)
Mid-Atlantic	960	12.4 (11.5, 13.4)	900	12.4 (11.5, 13.4)
East-North Central	1306	14.3 (13.3, 15.3)	1228	14.5 (13.4, 15.5)
West-North Central	647	6.4 (5.8, 7.1)	597	6.4 (5.8, 7.1)
South Atlantic	1754	20.6 (19.4, 21.7)	1624	20.4 (19.2, 21.6)
East-South Central	514	5.9 (5.2, 6.6)	463	5.9 (5.2, 6.6)
West-South Central	902	11.7 (10.7, 12.7)	854	11.8 (10.8, 12.8)
Mountain	796	7.7 (6.9, 8.4)	733	7.7 (6.9, 8.5)
Pacific	1655	16.3 (15.3, 17.3)	1509	16.1 (15.1, 17.2)
Non-response	0	0.0 (0.0, 0.0)	0	0.0 (0.0, 0.0)

* Most values are as of 2022; Census division values were updated for 2024 and 2025.

Table S2. Sociodemographic characteristics of respondents and non-respondents (unweighted) in 2024 and 2025*

Characteristics	2024 (Wave3)				Wave3 respondents who left the panel prior to Wave4 (n = 468)		2025 (Wave4)			
	Respondents* (n=8896)		Non-respondents (n = 1168)				Respondents* (n=8248)		Non-respondents (n = 931)	
	Unweighted n	Unweighted %	Unweighted n	Unweighted %	Unweighted n	Unweighted %	Unweighted n	Unweighted %	Unweighted n	Unweighted %
**Age**										
18-24	176	2.0	64	5.5	19	4.1	138	1.7	56	6
25-34	753	8.5	139	11.9	44	9.4	631	7.7	115	12.4
35-44	1094	12.3	192	16.4	51	10.9	977	11.8	168	18
45-54	1150	12.9	169	14.5	59	12.6	1077	13.1	136	14.6
55-64	1827	20.5	232	19.9	78	16.7	1672	20.3	174	18.7
65-74	2249	25.3	239	20.5	110	23.5	2089	25.3	177	19
75+	1647	18.5	133	11.4	107	22.9	1664	20.2	105	11.3
Non-response	0	0.0	0	0.0	0	0.0	0	0.0	0	0.0
**Gender**										
Male	5147	57.9	580	49.7	262	56	4780	58.9	457	49.1
Female	3749	42.1	588	50.3	206	44	3468	42.0	474	50.9
Non-response	0	0.0	0	0.0	0	0.0	0	0.0	0	0.0
**Race/Ethnicity**										
Black, non-Hispanic	702	7.9	128	11.0	32	6.8	670	8.1	88	9.5
Hispanic, any race	959	10.8	181	15.5	47	10	889	10.8	157	16.9
White, non-Hispanic	6673	75.0	778	66.6	361	77.1	6149	74.6	619	66.5
Other, non-Hispanic	316	3.6	49	4.2	13	2.8	301	3.6	44	4.7
2+races, non-Hispanic	246	2.8	32	2.7	15	3.2	239	2.9	23	2.5
Non-response	0	0.0	0	0.0	0	0.0	0	0.0	0	0.0
**Marital status**										
Now married	5655	63.6	681	58.3	281	60	5245	63.6	569	61.1
Widowed	630	7.1	83	7.1	33	7.1	604	7.3	48	5.2
Divorced	979	11.0	117	10	57	12.2	897	10.9	98	10.5
Separated	127	1.4	17	1.5	4	0.9	120	1.5	20	2.1
Never married	1505	16.9	270	23.1	93	19.9	1382	16.8	196	21.1
Non-response	0	0.0	0	0.0	0	0.0	0	0.0	0	0.0
**Education**										
No high school diploma or GED	330	3.7	66	5.7	23	4.9	287	3.5	59	6.3
High school graduate (diploma, GED	1784	20.1	253	21.7	100	21.4	1669	20.2	220	23.6
Some college or Associate's degree	2691	30.2	354	30.3	135	28.8	2471	30	280	30.1
Bachelor's degree	2257	25.4	279	23.9	108	23.1	2105	25.5	219	23.5
Master’s degree or higher	1834	20.6	216	18.5	102	21.8	1716	20.8	153	16.4
Non-response	0	0.0	0	0.0	0	0.0	0	0.0	0	0.0

Table S2, continued.

Characteristics	2024 (Wave3)				Wave3 respondents who left the panel prior to Wave4 (n = 468)		2025 (Wave4)			
	Respondents* (n=8896)		Non-respondents (n = 1168)				Respondents* (n=8248)		Non-respondents (n = 931)	
	Unweighted n	Unweighted %	Unweighted n	Unweighted %	Unweighted n	Unweighted %	Unweighted n	Unweighted %	Unweighted n	Unweighted %
**Household Income**										
Less than $10,000	265	3	40	3.4	11	2.4	196	2.4	37	4
$10,000 to $24,999	609	6.8	132	11.3	43	9.2	570	6.9	98	10.5
$25,000 to $49,999	1446	16.3	220	18.8	92	19.7	1253	15.2	173	18.6
$50,000 to $74,999	1424	16	220	18.8	93	19.9	1262	15.3	139	14.9
$75,000 to $99,999	1367	15.4	160	13.7	78	16.7	1246	15.1	136	14.6
$100,000 to $149,999	1799	20.2	206	17.6	71	15.2	1674	20.3	180	19.3
$150,000 or more	1986	22.3	190	16.3	80	17.1	2047	24.8	168	18
Non-response	0	0.0	0	0	0	0.0	0	0.0	0	0.0
**Employment**										
Working full-time	3593	40.4	525	44.9	157	33.5	3268	39.6	408	43.8
Working part-time	1038	11.7	160	13.7	50	10.7	973	11.8	142	15.3
Not working	4265	47.9	483	41.4	261	55.8	4007	48.6	381	40.9
Non-response	0	0.0	0	0.0	0	0.0	0	0.0	0	0.0
**Census division**										
New England	362	4.1	42	3.6	19	4.1	340	4.1	30	3.2
Mid-Atlantic	960	10.8	148	12.7	42	9	900	10.9	101	10.8
East-North Central	1306	14.7	178	15.2	61	13	1228	14.9	144	15.5
West-North Central	647	7.3	72	6.2	37	7.9	597	7.2	55	5.9
South Atlantic	1754	19.7	233	19.9	89	19	1624	19.7	190	20.4
East-South Central	514	5.8	63	5.4	32	6.8	463	5.6	56	6
West-South Central	902	10.1	123	10.5	47	10	854	10.4	96	10.3
Mountain	796	8.9	87	7.4	38	8.1	733	8.9	71	7.6
Pacific	1655	18.6	222	19.0	103	22	1509	18.3	188	20.2
Non-response	0	0.0	0	0.0	0	0.0	0	0.0	0	0.0

* Most values are as of 2022; Census division values were updated for 2024 and 2025. Mean (SD) ages were as follows: Wave 3 responders, 56.8 (16.5); Wave 3 non-responders, 53.8 (17.5); Wave 3 respondents who left the panel prior to Wave 4, 58.9 (18.1); Wave 4 responders, 59.7 (16.3); Wave 4 non-responders 52.6 (17.6).

Table S3. Views on democracy and authoritarianism

Statement	2024 Respondents* (n=8896)		2025 Respondents* (n=8248)		Mean Difference,* 2024-2025	
	**Unweighted n**	**Weighted % (95% CI)** *Mean score (95% CI)*	**Unweighted n**	**Weighted % (95% CI)** *Mean score (95% CI)*	**Unweighted n**	**Weighted % (95% CI)** *Mean score (95% CI)*
When thinking about democracy in the United States these days, do you believe…						
There is a serious threat to our democracy. (1)	6256	64.8 (63.4, 66.2)	5236	61.1 (59.7, 62.6)	7767	-3.5 (-5.1, -1.9)
There may be a threat to our democracy, but it is not serious. (2)	1953	25.9 (24.6, 27.2)	1878	25.0 (23.7, 26.3)	7767	-1.1 (-2.7, 0.5)
There is no threat to our democracy. (3)	573	7.3 (6.5, 8.1)	999	11.2 (10.2, 12.1)	7767	4.2 (3.1, 5.2)
Non-response	114	2.0 (1.5, 2.4)	135	2.7 (2.1, 3.3)	7767	0.5 (-0.1, 1.0)
Mean score†	*8782*	*1.41 (1.39, 1.43)*	*8113*	*1.49 (1.47, 1.51)*	*7600*	*0.077 (0.054, 0.100)*
How important do you think it is for the United States to remain a democracy?						
Not important (1)	193	3.5 (2.8, 4.1)	192	3.3 (2.7, 3.9)	7767	-0.4 (-1.2, 0.3)
Somewhat important (2)	411	7.6 (6.7, 8.5)	363	7.1 (6.3, 8.0)	7767	-0.4 (-1.5, 0.6)
Very or extremely important (3)	8208	87.5 (86.4, 88.6)	7594	88.0 (86.9, 89.1)	7767	0.8 (-0.3, 1.9)
Non-response	84	1.4 (1.0, 1.8)	99	1.6 (1.2, 2.0)	7767	0.1 (-0.4, 0.5)
Mean score†	*8812*	*2.85 (2.84, 2.87)*	*8149*	*2.86 (2.85, 2.88)*	*7645*	*0.012 (-0.003, 0.026)*
How much do you agree or disagree with the following statements about democracy in the United States?						
Democracy is the best form of government.						
Do not agree (1)	493	7.2 (6.3, 8.0)	468	6.9 (6.1, 7.7)	7767	-0.3 (-1.2, 0.6)
Somewhat agree (2)	1507	22.0 (20.7, 23.3)	1408	21.9 (20.6, 23.2)	7767	-0.1 (-1.5, 1.4)
Strongly or very strongly agree (3)	6775	68.7 (67.3, 70.1)	6252	68.8 (67.3, 70.2)	7767	0.4 (-1.1, 1.8)
Non-response	121	2.2 (1.7, 2.6)	120	2.4 (1.9, 2.9)	7767	0.0 (-0.5, 0.5)
Mean score†	*8775*	*2.63 (2.61, 2.65)*	*8128*	*2.63 (2.61, 2.65)*	*7615*	*0.003 (-0.016, 0.022)*
These days, American democracy only serves the interest of the wealthy and powerful.						
Do not agree (1)	3301	31.8 (30.6, 33.1)	2988	30.0 (28.8, 31.3)	7767	-1.7 (-3.2, -0.2)
Somewhat agree (2)	3239	37.3 (35.9, 38.6)	2662	33.6 (32.3, 35.0)	7767	-3.5 (-5.3, -1.6)
Strongly or very strongly agree (3)	2259	29.2 (27.9, 30.5)	2488	34.0 (32.5, 35.4)	7767	4.7 (3.1, 6.4)
Non-response	97	1.7 (1.3, 2.1)	110	2.3 (1.8, 2.8)	7767	0.4 (0.0, 0.9)
Mean score†	*8799*	*1.97 (1.95, 2.00)*	*8138*	*2.04 (2.02, 2.06)*	*7638*	*0.068 (0.042, 0.094)*
Having a strong leader for America is more important than having a democracy.						
Do not agree (1)	6076	63.0 (61.6, 64.4)	5709	64.9 (63.5, 66.4)	7767	2.5 (1.0, 4.0)
Somewhat agree (2)	1403	18.8 (17.6, 19.9)	1279	17.5 (16.4, 18.7)	7767	-1.5 (-3.1, 0.0)
Strongly or very strongly agree (3)	1280	15.9 (14.9, 17.0)	1120	15.0 (13.9, 16.0)	7767	-1.2 (-2.4, 0.1)
Non-response	137	2.3 (1.8, 2.8)	140	2.6 (2.0, 3.1)	7767	0.2 (-0.3, 0.7)
Mean score†	*8759*	*1.52 (1.50, 1.54)*	*8108*	*1.49 (1.46, 1.51)*	*7577*	*-0.038 (-0.061, -0.014)*

Table S3, continued.

Statement	2024 Respondents* (n=8896)		2025 Respondents* (n=8248)		Mean Difference,* 2024-2025	
	Unweighted n	Weighted % (95% CI) *Mean score (95% CI)*	Unweighted n	Weighted % (95% CI) *Mean score (95% CI)*	Unweighted n	Weighted % (95% CI) *Mean score (95% CI)*
The 2020 election was stolen from Donald Trump, and Joe Biden is an illegitimate president.						
Do not agree (1)	5843	66.7 (65.3, 68.0)	5424	66.9 (65.5, 68.3)	7767	0.2 (-0.8, 1.3)
Somewhat agree (2)	1338	14.3 (13.3, 15.4)	1224	14.5 (13.4, 15.5)	7767	0.1 (-1.1, 1.3)
Strongly or very strongly agree (3)	1580	17.0 (16.0, 18.1)	1469	16.1 (15.1, 17.2)	7767	-0.8 (-1.8, 0.3)
Non-response	135	2.0 (1.5, 2.4)	131	2.5 (2.0, 3.1)	7767	0.4 (-0.1, 1.0)
Mean score†	*8761*	*1.49 (1.47, 1.52)*	*8117*	*1.48 (1.46, 1.50)*	*7595*	*-0.010 (-0.026, 0.007)*
We should suspend Congress for a few years so a strong leader can clean up the mess made by politicians in Washington.						
Do not agree (1)	6280	64.5 (63.1, 65.9)	6193	69.7 (68.3, 71.1)	7767	6.0 (4.6, 7.4)
Somewhat agree (2)	1366	18.7 (17.5, 19.8)	1094	16.0 (14.8, 17.1)	7767	-2.9 (-4.4, -1.4)
Strongly or very strongly agree (3)	1132	14.8 (13.8, 15.9)	827	11.6 (10.6, 12.6)	7767	-3.7 (-4.8, -2.5)
Non-response	118	2.0 (1.6, 2.5)	134	2.7 (2.2, 3.3)	7767	0.5 (0.0, 1.1)
Mean score†	*8778*	*1.49 (1.47, 1.52)*	*8114*	*1.40 (1.38, 1.42)*	*7600*	*-0.100 (-0.121, -0.079)*
Which is more important to you…?						
Having election outcomes determined democratically	5872	59.6 (58.1, 61.0)	5458	60.4 (58.9, 61.9)	7767	1.6 (-0.1, 3.2)
Having political leaders I can trust to look out for my values and interests	2722	34.9 (33.5, 36.3)	2496	33.7 (32.3, 35.1)	7767	-1.8 (-3.5, 0.0)
Non-response	302	5.5 (4.7, 6.2)	294	5.9 (5.1, 6.8)	7767	0.2 (-0.8, 1.2)
Mean score†	*8594*	*1.37 (1.36, 1.38)*	*7954*	*1.36 (1.34, 1.37)*	*7327*	*-0.024 (-0.041, -0.007)*

* Among respondents to both surveys (n=7767).

† Mean scores in 2024 and 2025 were scored as indicated in the response lines for individual questions, with non-responses excluded. To assess population-level change from 2024 to 2025, we computed within-individual change scores for each item and then calculated year-to-year population-level change scores based on the means of aggregated within-individual change scores. Mean change scores have a range from -2 to 2 (with 0 indicating no change).

Table S4. Views on returning Donald Trump to the presidency (2024) and removing him from it (2025)

**What do you think about the use of force or violence in the following situations?**	**2024 Respondents* (n=8896)**		**2025 Respondents* (n=8248)**	
	**Unweighted n**	**Weighted % (95% CI)** ***Mean score (95% CI)***	**Unweighted n**	**Weighted % (95% CI)** ***Mean score (95% CI)***
To return (2024) / remove (2025) Donald Trump to/from the presidency this year				
Never justified (1)	7977	88.6 (87.6, 89.6)	6321	73.7 (72.3, 75.0)
Sometimes justified (2)	395	5.9 (5.1, 6.6)	911	13.0 (11.9, 14.0)
Usually or always justified (3)	400	4.6 (4.0, 5.2)	902	12.6 (11.5, 13.6)
Non-response	66	1.0 (0.7, 1.3)	57	0.8 (0.6, 1.1)
Mean score†	8772	*1.15 (1.14, 1.17)*	8134	*1.38 (1.36, 1.41)*

* Among respondents to both surveys (n=7767). Respondents who did not answer the question "In general...to advance an important political objective that you support" in 2024 (n = 58) or 2025 (n = 57) were not asked these questions.

† Mean scores in 2024 and 2025 were scored as indicated in the response lines for individual questions, with non-responses excluded.

Table S5. Sociodemographic characteristics of respondents by party/MAGA affiliation *

Characteristic	MAGA Affiliation						No MAGA Affiliation	
	MAGA Republican (n = 1183)		MAGA Supporter, Republican (n = 565)		MAGA Supporter, Non-Republican (n = 197)		Non-MAGA, strong Republican (n = 417)	
	Unweighted n	Weighted % (95% CI)	Unweighted n	Weighted % (95% CI)	Unweighted n	Weighted % (95% CI)	Unweighted n	Weighted % (95% CI)
Age								
18-24	22	5.6 (3.5, 8.7)	13	10.1 (5.8, 17.0)	7	9.5 (4.4, 19.1)	12	9.2 (5.2, 15.8)
25-34	49	9.8 (7.0, 13.5)	32	10.6 (7.1, 15.6)	28	23.6 (15.7, 33.9)	32	12.8 (8.5, 18.8)
35-44	107	12.6 (10.2, 15.5)	56	13.0 (9.6, 17.4)	29	24.9 (17.0, 35.0)	59	20.2 (15.2, 26.3)
45-54	163	15.4 (12.9, 18.2)	79	14.8 (11.1, 19.4)	26	11.9 (7.4, 18.5)	50	11.4 (8.3, 15.5)
55-64	293	21.9 (19.2, 24.9)	139	21.1 (17.4, 25.3)	39	14.0 (9.5, 20.1)	127	25.8 (20.8, 31.5)
65-74	345	21.1 (18.4, 24.0)	158	19.8 (16.3, 23.8)	46	11.1 (7.2, 16.8)	85	12.3 (9.5, 15.8)
75+	204	13.6 (11.4, 16.2)	88	10.5 (8.0, 13.8)	22	4.9 (3.0, 8.1)	52	8.4 (5.9, 11.8)
Non-response	0	0.0 (0.0, 0.0)	0	0.0 (0.0, 0.0)	0	0.0 (0.0, 0.0)	0	0.0 (0.0, 0.0)
Gender								
Female	419	44.7 (40.9, 48.6)	149	36.9 (31.3, 42.8)	80	53.9 (44.1, 63.3)	191	57.0 (50.5, 63.3)
Male	754	54.9 (51.0, 58.7)	410	62.3 (56.3, 67.9)	107	39.2 (30.5, 48.7)	223	42.9 (36.7, 49.4)
Transgender	2	0.2 (0.0, 0.8)	1	0.4 (0.1, 2.7)	5	4.9 (1.6, 14.3)	0	0.0 (0.0, 0.0)
Non-binary	0	0.0 (0.0, 0.0)	0	0.0 (0.0, 0.0)	2	1.8 (0.4, 6.9)	1	0.0 (0.0, 0.3)
Other	2	0.2 (0.0, 0.9)	2	0.5 (0.1, 1.8)	1	0.3 (0.0, 1.8)	0	0.0 (0.0, 0.0)
Non-response	0	0.0 (0.0, 0.0)	0	0.0 (0.0, 0.0)	0	0.0 (0.0, 0.0)	0	0.0 (0.0, 0.0)
Race/Ethnicity								
White, Non-Hispanic	1036	82.0 (78.1, 85.4)	497	83.2 (77.4, 87.8)	138	56.8 (46.7, 66.5)	360	81.0 (74.4, 86.2)
Black, Non-Hispanic	15	2.0 (1.1, 3.6)	5	1.6 (0.7, 3.8)	14	17.0 (9.5, 28.4)	3	1.0 (0.3, 3.6)
Hispanic, any race	75	9.5 (7.1, 12.5)	27	5.1 (3.3, 7.9)	28	17.0 (10.8, 25.7)	35	13.9 (9.2, 20.5)
American Indian or Alaska Native, Non-Hispanic	7	1.2 (0.4, 3.7)	4	3.3 (1.0, 10.5)	3	2.8 (0.7, 10.8)	2	0.8 (0.2, 3.3)
Asian American or Pacific Islander, non-Hispanic	17	3.3 (1.8, 6.3)	11	3.7 (1.8, 7.3)	5	4.6 (1.7, 11.9)	5	1.5 (0.6, 4.1)
Some other race, Non-Hispanic	2	0.2 (0.1, 0.9)	3	0.6 (0.2, 2.2)	0	0.0 (0.0, 0.0)	1	0.3 (0.0, 2.0)
2+ Races, Non-Hispanic	31	1.8 (1.0, 3.1)	18	2.5 (1.1, 5.4)	9	1.8 (0.8, 4.1)	11	1.5 (0.7, 3.1)
Non-response	0	0.0 (0.0, 0.0)	0	0.0 (0.0, 0.0)	0	0.0 (0.0, 0.0)	0	0.0 (0.0, 0.0)
Marital status								
Now married	807	63.1 (59.1, 66.9)	413	64.9 (58.7, 70.7)	113	44.4 (35.3, 53.9)	317	73.9 (67.4, 79.4)
Widowed	81	5.9 (4.3, 8.0)	22	2.9 (1.7, 5.0)	9	5.5 (2.4, 11.8)	28	4.8 (3.1, 7.4)
Divorced	137	9.2 (7.5, 11.2)	61	10.2 (7.6, 13.5)	29	11.1 (7.0, 17.1)	27	3.9 (2.4, 6.1)
Separated	18	1.9 (1.0, 3.6)	5	0.4 (0.1, 0.9)	4	1.6 (0.5, 4.8)	9	1.4 (0.6, 2.9)
Never married	140	19.9 (16.5, 23.8)	64	21.6 (16.2, 28.3)	42	37.4 (27.8, 48.1)	36	16.1 (11.1, 22.7)
Non-response	0	0.0 (0.0, 0.0)	0	0.0 (0.0, 0.0)	0	0.0 (0.0, 0.0)	0	0.0 (0.0, 0.0)
Education								
No high school diploma or GED	36	6.3 (4.2, 9.3)	13	6.1 (3.2, 11.4)	17	19.4 (11.9, 30.1)	17	8.7 (5.0, 14.6)
High school graduate (diploma, GED)	314	36.4 (32.6, 40.3)	98	23.6 (18.5, 29.7)	45	27.4 (19.6, 36.9)	103	29.9 (24.1, 36.3)
Some college or Associate's degree	434	33.9 (30.4, 37.5)	164	29.9 (25.0, 35.3)	64	26.8 (19.3, 35.9)	123	29.5 (23.9, 35.9)
Bachelor's degree	258	15.4 (13.1, 17.9)	178	25.3 (21.1, 30.0)	46	18.9 (12.9, 26.8)	108	21.9 (17.4, 27.3)
Master's degree or higher	141	8.1 (6.6, 9.9)	112	15.1 (11.9, 18.8)	25	7.5 (4.6, 12.1)	66	10.0 (7.3, 13.5)
Non-response	0	0.0 (0.0, 0.0)	0	0.0 (0.0, 0.0)	0	0.0 (0.0, 0.0)	0	0.0 (0.0, 0.0)

Table S5, continued.

Characteristic	MAGA Affiliation						No MAGA Affiliation	
	MAGA Republican (n = 1183)		MAGA Supporter, Republican (n = 565)		MAGA Supporter, Non-Republican (n = 197)		Non-MAGA, strong Republican (n = 417)	
Household Income								
Less than $10,000	21	3.1 (1.9, 5.0)	8	1.6 (0.7, 3.7)	20	17.7 (10.8, 27.7)	8	2.4 (0.9, 5.9)
$10,000 to $24,999	79	8.8 (6.5, 11.9)	15	3.1 (1.7, 5.7)	16	9.4 (5.2, 16.4)	23	5.7 (3.1, 10.2)
$25,000 to $49,999	226	20.3 (17.3, 23.6)	74	14.7 (10.7, 19.9)	30	15.5 (9.8, 23.8)	60	14.8 (10.5, 20.4)
$50,000 to $74,999	232	17.0 (14.5, 19.9)	98	17.1 (13.1, 22.0)	31	9.7 (6.0, 15.3)	77	18.0 (13.6, 23.4)
$75,000 to $99,999	195	12.5 (10.5, 14.9)	98	12.8 (9.9, 16.4)	40	14.4 (9.2, 21.7)	76	15.1 (11.4, 19.6)
$100,000 to $149,999	255	21.6 (18.6, 24.9)	107	19.9 (15.5, 25.1)	32	12.7 (8.0, 19.5)	76	20.4 (15.4, 26.4)
$150,000 or more	175	16.7 (14.1, 19.8)	165	30.9 (26.0, 36.3)	28	20.6 (13.3, 30.4)	97	23.7 (18.6, 29.7)
Non-response	0	0.0 (0.0, 0.0)	0	0.0 (0.0, 0.0)	0	0.0 (0.0, 0.0)	0	0.0 (0.0, 0.0)
Employment								
Working - as a paid employee	486	46.0 (42.2, 49.9)	255	50.5 (44.8, 56.3)	83	48.6 (39.1, 58.3)	212	55.6 (49.0, 62.0)
Working - self-employed	76	6.2 (4.7, 8.3)	48	9.9 (6.9, 14.2)	11	5.0 (2.0, 11.9)	28	8.2 (4.9, 13.4)
Not working - on temporary layoff from a job	4	0.5 (0.2, 1.4)	2	0.2 (0.0, 1.1)	2	1.6 (0.3, 8.3)	1	0.3 (0.0, 2.3)
Not working - looking for work	24	3.0 (1.8, 4.9)	14	6.0 (3.1, 11.6)	10	7.5 (3.7, 14.7)	6	1.5 (0.4, 6.0)
Not working - retired	536	35.1 (31.7, 38.7)	223	26.8 (22.7, 31.4)	67	18.2 (13.0, 25.0)	136	21.0 (17.0, 25.7)
Not working - disabled	22	3.2 (1.8, 5.6)	9	2.7 (1.3, 5.8)	13	9.3 (4.9, 17.0)	14	4.7 (2.5, 8.6)
Not working - other	35	5.9 (3.9, 8.7)	14	3.7 (1.9, 7.1)	11	9.8 (4.9, 18.6)	20	8.6 (5.0, 14.6)
Non-response	0	0.0 (0.0, 0.0)	0	0.0 (0.0, 0.0)	0	0.0 (0.0, 0.0)	0	0.0 (0.0, 0.0)
Census division								
New England	42	4.4 (3.0, 6.3)	24	5.1 (3.0, 8.3)	7	3.8 (1.6, 9.0)	10	2.2 (1.0, 4.7)
Mid-Atlantic	123	11.5 (9.2, 14.4)	72	16.9 (12.9, 21.9)	29	17.4 (11.4, 25.6)	53	14.6 (10.5, 20.0)
East-North Central	166	12.0 (10.0, 14.4)	83	12.6 (9.5, 16.4)	36	15.6 (9.6, 24.3)	58	15.8 (11.5, 21.4)
West-North Central	89	7.9 (6.1, 10.3)	39	6.0 (3.9, 9.0)	9	5.2 (2.0, 12.9)	38	8.4 (5.5, 12.7)
South Atlantic	273	24.3 (21.0, 27.9)	107	17.0 (13.3, 21.5)	40	22.8 (15.4, 32.4)	78	17.5 (13.3, 22.6)
East-South Central	85	7.9 (6.0, 10.3)	42	10.0 (6.4, 15.3)	11	7.4 (3.6, 14.6)	24	8.5 (5.0, 14.2)
West-South Central	139	12.6 (10.2, 15.4)	69	14.3 (10.7, 19.0)	16	7.1 (3.9, 12.6)	43	7.9 (5.4, 11.3)
Mountain	119	8.0 (6.3, 10.1)	51	9.2 (6.2, 13.5)	14	4.2 (2.0, 8.9)	53	12.2 (8.4, 17.4)
Pacific	147	11.4 (9.2, 14.0)	78	9.0 (6.7, 11.9)	35	16.6 (10.6, 24.9)	60	12.9 (9.2, 17.8)
Non-response	0	0.0 (0.0, 0.0)	0	0.0 (0.0, 0.0)	0	0.0 (0.0, 0.0)	0	0.0 (0.0, 0.0)

Table S5, continued.

Characteristic	No MAGA Affiliation							
	Non-MAGA, leans or not strong Republican (n = 1083)		Non-MAGA, Independent (n = 932)		Non-MAGA, leans or not strong Democrat (n = 1849)		Non-MAGA, strong Democrat (n = 1332)	
	Unweighted n	Weighted % (95% CI)	Unweighted n	Weighted % (95% CI)	Unweighted n	Weighted % (95% CI)	Unweighted n	Weighted % (95% CI)
Age								
18-24	23	5.0 (3.1, 7.7)	47	14.1 (10.7, 18.3)	70	11.0 (8.7, 13.8)	41	9.9 (7.3, 13.4)
25-34	88	16.9 (13.6, 20.9)	109	19.7 (16.2, 23.8)	207	18.5 (16.0, 21.3)	126	16.6 (13.8, 19.9)
35-44	165	19.9 (16.9, 23.4)	183	23.8 (20.4, 27.5)	286	20.4 (17.9, 23.0)	117	12.5 (10.2, 15.2)
45-54	163	16.4 (13.8, 19.3)	137	12.7 (10.5, 15.3)	247	14.1 (12.2, 16.3)	133	11.3 (9.3, 13.7)
55-64	275	21.7 (18.7, 25.0)	194	15.4 (12.9, 18.3)	369	15.2 (13.4, 17.2)	292	19.8 (17.4, 22.4)
65-74	237	13.4 (11.4, 15.7)	181	10.4 (8.5, 12.5)	460	14.3 (12.8, 16.0)	412	20.1 (17.8, 22.5)
75+	132	6.8 (5.2, 8.7)	81	4.0 (3.0, 5.2)	210	6.5 (5.4, 7.7)	211	9.8 (8.3, 11.6)
Non-response	0	0.0 (0.0, 0.0)	0	0.0 (0.0, 0.0)	0	0.0 (0.0, 0.0)	0	0.0 (0.0, 0.0)
Gender								
Female	392	49.8 (45.8, 53.8)	396	51.9 (47.6, 56.1)	760	49.1 (46.1, 52.2)	655	56.3 (52.7, 59.7)
Male	679	49.9 (45.9, 54.0)	510	46.1 (41.9, 50.4)	1044	48.3 (45.3, 51.4)	645	42.2 (38.7, 45.7)
Transgender	0	0.0 (0.0, 0.0)	3	0.2 (0.1, 0.7)	12	0.4 (0.2, 0.8)	15	0.8 (0.4, 1.4)
Non-binary	3	0.1 (0.0, 0.4)	11	1.3 (0.6, 2.6)	24	1.7 (1.0, 2.8)	9	0.6 (0.3, 1.4)
Other	2	0.1 (0.0, 0.6)	4	0.5 (0.1, 2.1)	4	0.4 (0.1, 1.3)	2	0.2 (0.0, 0.7)
Non-response	0	0.0 (0.0, 0.0)	0	0.0 (0.0, 0.0)	0	0.0 (0.0, 0.0)	0	0.0 (0.0, 0.0)
Race/Ethnicity								
White, Non-Hispanic	915	77.8 (73.8, 81.4)	620	52.9 (48.6, 57.2)	1247	53.8 (50.7, 56.9)	883	55.5 (51.8, 59.1)
Black, Non-Hispanic	26	3.4 (2.1, 5.5)	107	15.7 (12.7, 19.4)	195	13.9 (11.7, 16.3)	237	22.3 (19.3, 25.6)
Hispanic, any race	85	10.8 (8.4, 13.7)	125	20.7 (17.2, 24.8)	267	23.0 (20.2, 26.0)	133	16.6 (13.8, 20.0)
American Indian or Alaska Native, Non-Hispanic	4	1.0 (0.3, 3.5)	11	2.7 (1.3, 5.5)	7	0.5 (0.2, 1.0)	3	0.1 (0.0, 0.4)
Asian American or Pacific Islander, non-Hispanic	31	5.8 (3.9, 8.8)	37	5.8 (4.0, 8.5)	74	6.5 (5.0, 8.4)	37	4.5 (3.1, 6.6)
Some other race, Non-Hispanic	1	0.0 (0.0, 0.3)	3	0.1 (0.0, 0.3)	5	0.1 (0.0, 0.3)	1	0.0 (0.0, 0.3)
2+ Races, Non-Hispanic	21	1.2 (0.4, 3.2)	29	2.0 (1.1, 3.8)	54	2.3 (1.5, 3.5)	38	0.9 (0.6, 1.4)
Non-response	0	0.0 (0.0, 0.0)	0	0.0 (0.0, 0.0)	0	0.0 (0.0, 0.0)	0	0.0 (0.0, 0.0)
Marital status								
Now married	747	67.5 (63.5, 71.3)	547	49.1 (44.9, 53.4)	1126	54.4 (51.3, 57.5)	790	52.9 (49.3, 56.5)
Widowed	64	3.6 (2.4, 5.4)	56	3.8 (2.7, 5.5)	94	3.0 (2.4, 3.9)	87	4.3 (3.3, 5.5)
Divorced	113	9.0 (7.1, 11.2)	90	6.7 (5.2, 8.7)	212	8.4 (7.1, 9.9)	143	7.9 (6.4, 9.6)
Separated	12	1.4 (0.7, 2.8)	16	2.4 (1.2, 4.4)	21	1.1 (0.6, 2.0)	17	1.1 (0.6, 2.0)
Never married	147	18.6 (15.4, 22.3)	223	37.9 (33.6, 42.4)	396	33.1 (30.0, 36.3)	295	33.8 (30.2, 37.7)
Non-response	0	0.0 (0.0, 0.0)	0	0.0 (0.0, 0.0)	0	0.0 (0.0, 0.0)	0	0.0 (0.0, 0.0)
Education								
No high school diploma or GED	36	7.5 (5.1, 10.9)	55	11.9 (8.9, 15.8)	78	9.3 (7.3, 11.8)	52	9.1 (6.7, 12.2)
High school graduate (diploma, GED)	194	22.6 (19.3, 26.3)	230	35.1 (31.0, 39.5)	305	23.6 (20.9, 26.6)	227	22.5 (19.5, 25.8)
Some college or Associate's degree	319	27.2 (23.8, 30.8)	290	29.5 (25.9, 33.4)	499	24.5 (22.0, 27.1)	353	24.1 (21.3, 27.2)
Bachelor's degree	326	26.5 (23.3, 30.0)	190	12.7 (10.7, 15.2)	515	23.0 (20.8, 25.4)	334	22.3 (19.6, 25.2)
Master’s degree or higher	208	16.2 (13.6, 19.2)	167	10.7 (8.8, 12.8)	452	19.6 (17.5, 21.9)	366	22.0 (19.5, 24.8)
Non-response	0	0.0 (0.0, 0.0)	0	0.0 (0.0, 0.0)	0	0.0 (0.0, 0.0)	0	0.0 (0.0, 0.0)

Table S5, continued.

Characteristic	No MAGA Affiliation							
	Non-MAGA, leans or not strong Republican (n = 1083)		Non-MAGA, Independent (n = 932)		Non-MAGA, leans or not strong Democrat (n = 1849)		Non-MAGA, strong Democrat (n = 1332)	
	Unweighted n	Weighted % (95% CI)	Unweighted n	Weighted % (95% CI)	Unweighted n	Weighted % (95% CI)	Unweighted n	Weighted % (95% CI)
Household Income								
Less than $10,000	19	3.2 (1.8, 5.6)	40	6.0 (4.2, 8.4)	35	2.5 (1.6, 3.8)	28	3.3 (2.0, 5.2)
$10,000 to $24,999	54	6.8 (4.7, 9.7)	102	13.4 (10.5, 16.9)	130	7.8 (6.2, 9.7)	97	7.7 (6.0, 9.9)
$25,000 to $49,999	148	12.9 (10.6, 15.6)	167	20.0 (16.7, 23.8)	291	16.1 (13.9, 18.5)	232	16.6 (14.1, 19.4)
$50,000 to $74,999	177	14.0 (11.5, 16.8)	159	18.5 (15.3, 22.3)	293	16.0 (13.8, 18.6)	209	16.7 (14.0, 19.7)
$75,000 to $99,999	168	11.9 (9.7, 14.4)	143	13.5 (10.9, 16.6)	301	14.2 (12.3, 16.4)	212	12.2 (10.3, 14.3)
$100,000 to $149,999	227	21.5 (18.3, 25.1)	141	12.0 (9.8, 14.7)	373	19.4 (17.1, 21.9)	246	17.7 (15.2, 20.5)
$150,000 or more	290	29.7 (26.2, 33.5)	180	16.5 (13.9, 19.6)	426	24.0 (21.6, 26.6)	308	25.9 (22.9, 29.2)
Non-response	0	0.0 (0.0, 0.0)	0	0.0 (0.0, 0.0)	0	0.0 (0.0, 0.0)	0	0.0 (0.0, 0.0)
Employment								
Working - as a paid employee	586	59.9 (55.9, 63.8)	469	52.7 (48.4, 57.0)	927	58.1 (55.1, 61.0)	531	48.0 (44.4, 51.6)
Working - self-employed	77	6.8 (5.1, 9.1)	82	9.1 (6.8, 12.2)	130	5.9 (4.8, 7.3)	103	6.9 (5.4, 8.8)
Not working - on temporary layoff from a job	2	0.2 (0.0, 0.9)	4	0.7 (0.2, 2.1)	6	0.3 (0.1, 0.9)	8	0.8 (0.4, 2.0)
Not working - looking for work	13	2.1 (1.1, 4.2)	44	8.7 (6.2, 12.0)	61	5.4 (4.0, 7.3)	31	3.7 (2.4, 5.7)
Not working - retired	338	19.9 (17.3, 22.8)	238	13.9 (11.7, 16.3)	590	18.2 (16.4, 20.1)	567	27.8 (25.1, 30.6)
Not working - disabled	19	1.8 (1.0, 3.1)	37	6.0 (4.1, 8.7)	55	4.1 (2.9, 5.6)	40	5.3 (3.7, 7.6)
Not working - other	48	9.3 (6.8, 12.6)	58	8.9 (6.7, 11.7)	80	8.0 (6.2, 10.1)	52	7.5 (5.4, 10.2)
Non-response	0	0.0 (0.0, 0.0)	0	0.0 (0.0, 0.0)	0	0.0 (0.0, 0.0)	0	0.0 (0.0, 0.0)
Census division								
New England	37	3.1 (2.1, 4.6)	43	4.7 (3.2, 6.8)	97	5.9 (4.6, 7.6)	56	5.2 (3.8, 7.1)
Mid-Atlantic	118	13.1 (10.6, 16.1)	87	9.0 (7.0, 11.6)	200	12.5 (10.5, 14.7)	140	13.5 (11.1, 16.3)
East-North Central	174	16.5 (13.7, 19.9)	115	12.4 (9.9, 15.5)	289	14.7 (12.7, 16.9)	188	14.9 (12.6, 17.6)
West-North Central	102	8.8 (6.8, 11.2)	58	5.4 (3.8, 7.5)	126	6.2 (4.9, 7.7)	91	5.5 (4.2, 7.3)
South Atlantic	209	18.3 (15.5, 21.6)	201	23.2 (19.7, 27.2)	330	19.1 (16.7, 21.7)	250	20.9 (18.1, 24.0)
East-South Central	57	5.3 (3.8, 7.3)	52	6.1 (4.3, 8.6)	74	3.9 (2.9, 5.2)	71	5.4 (3.9, 7.3)
West-South Central	129	14.1 (11.4, 17.3)	113	14.9 (11.9, 18.4)	155	10.6 (8.7, 12.7)	111	8.9 (7.1, 11.2)
Mountain	107	8.9 (7.0, 11.4)	84	7.2 (5.4, 9.5)	156	7.1 (5.7, 8.9)	100	7.1 (5.4, 9.4)
Pacific	150	11.9 (9.5, 14.8)	179	17.0 (14.2, 20.3)	422	20.1 (17.9, 22.6)	325	18.5 (16.1, 21.2)
Non-response	0	0.0 (0.0, 0.0)	0	0.0 (0.0, 0.0)	0	0.0 (0.0, 0.0)	0	0.0 (0.0, 0.0)

* Most values are as of 2022; Census division values were updated for 2024-2025.

Table S6. Party/MAGA affiliation and 2025 prevalence of beliefs concerning violence to effect social change and civil war

Statement	Population Estimates by Party Category for 2025*							
	MAGA Affiliation						No MAGA Affiliation	
	MAGA Republican (n = 1183)		MAGA Supporter, Republican (n = 565)		MAGA Supporter, Non-Republican (n = 197)		Non-MAGA, strong Republican (n = 417)	
If elected leaders will not protect American democracy, the people must do it themselves, even if it requires taking violent actions.	Unweighted n	Weighted % (95% CI) *Mean score (95% CI)*	Unweighted n	Weighted % (95% CI) *Mean score (95% CI)*	Unweighted n	Weighted % (95% CI) *Mean score (95% CI)*	Unweighted n	Weighted % (95% CI) *Mean score (95% CI)*
Do not agree (1)	646	57.6 (53.8, 61.4)	333	60.6 (55.0, 66.3)	101	49.3 (39.6, 58.9)	275	63.4 (56.9, 69.9)
Somewhat agree (2)	386	30.0 (26.6, 33.4)	181	28.1 (23.4, 32.8)	59	29.0 (20.1, 38.0)	109	28.6 (22.4, 34.7)
Strongly or very strongly agree (3)	140	11.3 (8.7, 13.9)	50	11.3 (6.6, 15.9)	34	19.9 (11.6, 28.2)	29	6.5 (3.2, 9.8)
Non-response	11	1.1 (0.4, 1.8)	1	0.0 (0.0, 0.1)	3	1.8 (0.0, 4.8)	4	1.5 (0.0, 3.2)
Mean score†	1172	*1.53 (1.48, 1.59)*	564	*1.51 (1.41, 1.60)*	194	*1.70 (1.54, 1.86)*	413	*1.42 (1.34, 1.50)*
aPD; q-value‡		*1.77 (-2.06, 5.61); 1.00*	*0.34 (-4.77, 5.45); 1.00*		*6.20 (-2.53, 14.93); 1.00*		*-2.98 (-7.16, 1.20); 1.00*	
Our American way of life is disappearing so fast that we may have to use force to save it.								
Do not agree (1)	603	50.2 (46.3, 54.0)	347	60.1 (54.4, 65.8)	87	42.5 (33.0, 52.0)	248	53.0 (46.4, 59.5)
Somewhat agree (2)	406	34.8 (31.1, 38.5)	169	29.0 (24.0, 34.0)	60	29.6 (20.8, 38.4)	122	32.6 (26.3, 38.9)
Strongly or very strongly agree (3)	159	12.8 (10.4, 15.1)	45	8.4 (5.2, 11.6)	46	26.0 (17.2, 34.7)	43	12.2 (7.7, 16.7)
Non-response	15	2.2 (0.6, 3.9)	4	2.5 (0.0, 5.9)	4	1.9 (0.0, 4.9)	4	2.2 (0.0, 4.8)
Mean score†	1168	*1.62 (1.57, 1.67)*	561	*1.47 (1.40, 1.54)*	193	*1.83 (1.67, 1.99)*	413	*1.58 (1.49, 1.68)*
aPD; q-value‡		*4.98 (1.28, 8.68); 0.40*	*1.26 (-2.91, 5.43); 1.00*		*13.16 (3.99, 22.33); 0.29*		*4.53 (-0.70, 9.76); 1.00*	
Because things have gotten so far off track, true American patriots may have to resort to violence in order to save our country.								
Do not agree (1)	745	64.3 (60.6, 67.9)	422	70.1 (64.4, 75.8)	122	60.8 (51.4, 70.3)	311	68.0 (61.4, 74.6)
Somewhat agree (2)	320	25.9 (22.6, 29.2)	114	23.8 (18.4, 29.1)	44	20.3 (12.8, 27.7)	83	25.0 (18.8, 31.1)
Strongly or very strongly agree (3)	111	9.0 (6.7, 11.2)	27	5.2 (2.6, 7.9)	28	17.1 (9.4, 24.7)	19	4.8 (1.7, 7.8)
Non-response	7	0.9 (0.0, 1.8)	2	0.9 (0.0, 2.5)	3	1.8 (0.0, 4.8)	4	2.2 (0.0, 4.8)
Mean score†	1176	*1.44 (1.39, 1.49)*	563	*1.35 (1.28, 1.41)*	194	*1.55 (1.40, 1.71)*	413	*1.35 (1.27, 1.43)*
aPD; q-value‡		*4.56 (1.30, 7.81); 0.32*	*1.13 (-2.53, 4.78); 1.00*		*7.74 (-0.01, 15.50); 1.00*		*0.31 (-3.37, 3.99); 1.00*	
In the next few years, there will be civil war in the United States.								
Do not agree (1)	812	68.0 (64.4, 71.6)	438	76.4 (71.5, 81.2)	119	54.8 (45.0, 64.5)	300	68.4 (62.2, 74.7)
Somewhat agree (2)	316	26.9 (23.5, 30.3)	106	19.8 (15.3, 24.3)	45	26.3 (17.4, 35.1)	96	24.6 (18.9, 30.3)
Strongly or very strongly agree (3)	45	3.8 (2.1, 5.5)	18	2.5 (1.0, 4.1)	30	18.2 (10.1, 26.2)	19	6.1 (2.5, 9.7)
Non-response	10	1.3 (0.3, 2.2)	3	1.3 (0.0, 3.1)	3	0.8 (0.0, 1.8)	2	0.9 (0.0, 2.4)
Mean score†	1173	*1.35 (1.31, 1.39)*	562	*1.25 (1.20, 1.30)*	194	*1.63 (1.47, 1.79)*	415	*1.37 (1.29, 1.46)*
aPD; q-value‡		*-4.08 (-7.10, -1.05); 0.40*	*-4.72 (-7.71, -1.74); 0.14*		*4.97 (-3.47, 13.41); 1.00*		*-1.75 (-6.09, 2.60); 1.00*	
The United States needs a civil war to set things right.								
Do not agree (1)	989	82.5 (79.4, 85.5)	513	89.2 (85.3, 93.1)	151	73.2 (64.0, 82.4)	373	85.2 (79.8, 90.6)
Somewhat agree (2)	145	12.6 (10.0, 15.2)	37	6.7 (3.8, 9.6)	28	20.3 (11.4, 29.2)	25	8.3 (4.1, 12.5)
Strongly or very strongly agree (3)	45	4.7 (2.8, 6.6)	11	2.7 (0.5, 4.9)	16	6.2 (2.2, 10.2)	18	5.8 (2.2, 9.4)
Non-response	4	0.2 (0.0, 0.5)	4	1.4 (0.0, 3.2)	2	0.3 (0.0, 0.7)	1	0.7 (0.0, 2.2)
Mean score†	1179	*1.22 (1.18, 1.26)*	561	*1.12 (1.07, 1.18)*	195	*1.33 (1.22, 1.44)*	416	*1.20 (1.12, 1.28)*
aPD; q-value‡		*2.47 (-0.13, 5.07); 1.00*	*0.30 (-2.37, 2.97); 1.00*		*0.12 (-4.45, 4.69); 1.00*		*3.38 (-0.52, 7.29); 1.00*	

Table S6, continued.

Statement	Population Estimates by Party Category for 2025*							
	No MAGA Affiliation							
	Non-MAGA, leans or not strong Republican (n = 1083)		Non-MAGA, Independent (n = 932)		Non-MAGA, leans or not strong Democrat (n = 1849)		Non-MAGA, strong Democrat (n = 1332)	
If elected leaders will not protect American democracy, the people must do it themselves, even if it requires taking violent actions.	Unweighted n	Weighted % (95% CI) *Mean score (95% CI)*	Unweighted n	Weighted % (95% CI) *Mean score (95% CI)*	Unweighted n	Weighted % (95% CI) *Mean score (95% CI)*	Unweighted n	Weighted % (95% CI) *Mean score (95% CI)*
Do not agree (1)	742	67.9 (64.1, 71.7)	584	62.9 (58.8, 67.0)	1191	64.0 (61.0, 66.9)	856	62.4 (58.8, 65.9)
Somewhat agree (2)	270	24.5 (21.1, 28.0)	243	23.3 (19.8, 26.8)	508	26.8 (24.1, 29.5)	348	25.8 (22.6, 29.0)
Strongly or very strongly agree (3)	59	5.9 (3.8, 7.9)	81	10.0 (7.4, 12.6)	138	8.5 (6.7, 10.3)	118	10.6 (8.1, 13.0)
Non-response	12	1.7 (0.6, 2.8)	24	3.8 (1.9, 5.7)	12	0.7 (0.2, 1.2)	10	1.3 (0.4, 2.1)
Mean score†	1071	*1.37 (1.32, 1.42)*	908	*1.45 (1.39, 1.51)*	1837	*1.44 (1.40, 1.48)*	1322	*1.48 (1.42, 1.53)*
aPD; q-value‡		*-3.63 (-6.97, -0.29); 1.00*		*-1.63 (-5.48, 2.22); 1.00*		*-2.47 (-5.56, 0.61); 1.00*		*Reference*
Our American way of life is disappearing so fast that we may have to use force to save it.								
Do not agree (1)	767	68.7 (64.9, 72.5)	616	64.3 (60.1, 68.4)	1269	66.0 (63.1, 69.0)	926	66.7 (63.2, 70.2)
Somewhat agree (2)	250	23.4 (20.0, 26.9)	220	22.8 (19.3, 26.3)	465	26.3 (23.6, 29.1)	297	22.8 (19.7, 25.9)
Strongly or very strongly agree (3)	56	6.4 (4.3, 8.6)	73	9.3 (6.8, 11.9)	101	6.8 (5.1, 8.5)	101	9.3 (6.9, 11.7)
Non-response	10	1.5 (0.5, 2.5)	23	3.6 (1.8, 5.5)	14	0.8 (0.3, 1.3)	8	1.1 (0.3, 2.0)
Mean score†	1073	*1.37 (1.32, 1.42)*	909	*1.43 (1.37, 1.49)*	1835	*1.40 (1.36, 1.44)*	1324	*1.42 (1.37, 1.47)*
aPD; q-value‡		*-0.94 (-4.31, 2.42); 1.00*		*-0.92 (-4.60, 2.75); 1.00*		*-2.18 (-5.18, 0.82); 1.00*		*Reference*
Because things have gotten so far off track, true American patriots may have to resort to violence in order to save our country.								
Do not agree (1)	900	81.9 (78.8, 85.1)	694	71.0 (67.1, 74.9)	1509	79.5 (76.9, 82.1)	1050	77.3 (74.2, 80.4)
Somewhat agree (2)	146	13.9 (11.2, 16.7)	163	18.4 (15.1, 21.7)	258	14.8 (12.5, 17.0)	213	15.2 (12.7, 17.7)
Strongly or very strongly agree (3)	26	2.6 (1.2, 4.1)	52	7.0 (4.7, 9.2)	71	5.2 (3.6, 6.7)	59	6.2 (4.1, 8.3)
Non-response	11	1.5 (0.5, 2.5)	23	3.6 (1.8, 5.5)	11	0.6 (0.1, 1.0)	10	1.3 (0.4, 2.2)
Mean score†	1072	*1.19 (1.16, 1.23)*	909	*1.34 (1.28, 1.39)*	1838	*1.25 (1.22, 1.29)*	1322	*1.28 (1.23, 1.33)*
aPD; q-value‡		*-2.04 (-4.60, 0.52); 1.00*		*0.12 (-3.02, 3.27); 1.00*		*-0.96 (-3.49, 1.58); 1.00*		*Reference*
In the next few years, there will be civil war in the United States.								
Do not agree (1)	801	71.7 (68.1, 75.4)	541	56.1 (51.9, 60.4)	1062	54.7 (51.6, 57.8)	741	51.5 (47.9, 55.0)
Somewhat agree (2)	245	23.8 (20.4, 27.3)	294	31.2 (27.3, 35.1)	659	36.8 (33.8, 39.7)	479	38.3 (34.8, 41.8)
Strongly or very strongly agree (3)	29	3.5 (1.7, 5.2)	70	8.8 (6.3, 11.2)	116	8.0 (6.1, 9.8)	102	9.3 (7.1, 11.5)
Non-response	8	1.0 (0.2, 1.7)	27	3.9 (2.0, 5.8)	12	0.6 (0.2, 0.9)	10	0.9 (0.2, 1.7)
Mean score†	1075	*1.31 (1.26, 1.36)*	905	*1.51 (1.45, 1.56)*	1837	*1.53 (1.49, 1.57)*	1322	*1.57 (1.53, 1.62)*
aPD; q-value‡		*-4.13 (-7.03, -1.22); 0.30*		*-1.48 (-5.08, 2.11); 1.00*		*-1.37 (-4.26, 1.52); 1.00*		*Reference*
The United States needs a civil war to set things right.								
Do not agree (1)	1005	90.8 (88.2, 93.3)	792	81.1 (77.6, 84.6)	1686	88.7 (86.6, 90.8)	1210	87.8 (85.2, 90.4)
Somewhat agree (2)	61	7.1 (4.9, 9.3)	84	11.0 (8.2, 13.8)	124	8.5 (6.7, 10.3)	87	8.3 (6.1, 10.5)
Strongly or very strongly agree (3)	10	1.2 (0.1, 2.3)	29	4.0 (2.3, 5.7)	33	2.5 (1.4, 3.6)	30	3.2 (1.8, 4.6)
Non-response	7	0.9 (0.2, 1.7)	27	3.9 (2.0, 5.7)	6	0.3 (0.0, 0.6)	5	0.7 (0.0, 1.4)
Mean score†	1076	*1.10 (1.06, 1.13)*	905	*1.20 (1.15, 1.24)*	1843	*1.14 (1.11, 1.16)*	1327	*1.15 (1.11, 1.18)*
aPD; q-value‡		*-1.24 (-3.10, 0.62); 1.00*		*-0.04 (-2.40, 2.32); 1.00*		*-0.90 (-2.75, 0.96); 1.00*		*Reference*

* Among respondents to both the 2024 and 2025 surveys (n=7767). Prevalences for 2024 among respondents to that survey were reported previously: Wintemute GJ, Velasquez B, Robinson SL, Tomsich EA, Wright MA, Shev AB. The MAGA movement and political violence in 2024: findings from a nationally representative survey. Inj Epidemiol. 2025;12(1):78.

† Mean scores in 2024 and 2025 were scored as indicated in the response lines for individual questions, with non-responses excluded.

‡ Adjusted prevalence differences (aPDs) are absolute percentage point (pp) differences for “strongly or very strongly agree” responses and are adjusted for age, race and ethnicity, gender, income, education, Census division, marital status, homeownership, rurality, firearm ownership, alcohol consumption, military service, and history of non-traffic arrest. Q-values, also known as FDR-adjusted (or FDR-corrected) p-values, represent the probability that the given difference would be a false discovery; they represent the expected proportion of “false positives” that would be seen among the collection of all differences whose q-values were at or below the given q-value. Item non-responses are not reported in the tables but are included in the prevalence calculations.

Table S7. Party/MAGA affiliation and 2024-2025 change in beliefs concerning violence to effect social change and civil war

Statement	Mean Differences* by Party Category 2024-2025							
	**MAGA Affiliation**						**No MAGA Affiliation**	
	**MAGA Republican (n = 1183)**		**MAGA Supporter, Republican (n = 565)**		**MAGA Supporter, Non-Republican (n = 197)**		**Non-MAGA, strong Republican (n = 417)**	
If elected leaders will not protect American democracy, the people must do it themselves, even if it requires taking violent actions.	**Unweighted n**	**Weighted mean (95% CI)** ***Mean score (95% CI)***	**Unweighted n**	**Weighted mean (95% CI)** ***Mean score (95% CI)***	**Unweighted n**	**Weighted mean (95% CI)** ***Mean score (95% CI)***	**Unweighted n**	**Weighted mean (95% CI)** ***Mean score (95% CI)***
Do not agree (1)	1183	15.9 (11.8, 20.0)	565	12.7 (6.5, 19.0)	197	12.8 (3.6, 22.1)	417	7.7 (1.0, 14.4)
Somewhat agree (2)	1183	-5.6 (-9.8, -1.4)	565	-6.0 (-11.6, -0.3)	197	-6.4 (-16.9, 4.1)	417	2.6 (-5.5, 10.7)
Strongly or very strongly agree (3)	1183	-6.8 (-9.4, -4.2)	565	-2.0 (-6.2, 2.2)	197	-7.8 (-17.3, 1.7)	417	-6.4 (-11.6, -1.2)
Non-response	1183	-0.2 (-1.5, 1.0)	565	-2.5 (-5.2, 0.1)	197	1.1 (-2.0, 4.3)	417	0.3 (-2.4, 2.9)
Mean score†	1165	*-0.287 (-0.352, -0.222)*	557	*-0.174 (-0.284, -0.063)*	192	*-0.221 (-0.407, -0.035)*	412	*-0.244 (-0.357, -0.131)*
Our American way of life is disappearing so fast that we may have to use force to save it.								
Do not agree (1)	1183	19.6 (15.4, 23.7)	565	17.1 (10.9, 23.3)	197	0.3 (-8.5, 9.1)	417	7.6 (0.6, 14.6)
Somewhat agree (2)	1183	-5.8 (-10.4, -1.2)	565	-10.2 (-16.5, -3.9)	197	-0.1 (-11.1, 11.0)	417	1.7 (-6.9, 10.4)
Strongly or very strongly agree (3)	1183	-8.7 (-12.3, -5.2)	565	-4.5 (-9.5, 0.4)	197	0.3 (-9.4, 10.1)	417	-5.1 (-11.6, 1.3)
Non-response	1183	1.2 (-0.3, 2.7)	565	0.8 (-3.2, 4.8)	197	1.4 (-1.7, 4.5)	417	1.8 (-0.8, 4.4)
Mean score†	1163	*-0.413 (-0.489, -0.337)*	555	*-0.261 (-0.369, -0.154)*	192	*-0.048 (-0.218, 0.122)*	412	*-0.257 (-0.389, -0.126)*
Because things have gotten so far off track, true American patriots may have to resort to violence in order to save our country.								
Do not agree (1)	1183	16.2 (12.5, 19.8)	565	12.1 (5.9, 18.3)	197	10.2 (-1.0, 21.4)	417	7.7 (0.3, 15.1)
Somewhat agree (2)	1183	-8.2 (-12.1, -4.3)	565	-5.0 (-11.7, 1.8)	197	-0.9 (-10.5, 8.7)	417	3.3 (-4.7, 11.4)
Strongly or very strongly agree (3)	1183	-3.3 (-6.0, -0.6)	565	-4.4 (-8.6, -0.2)	197	-10.4 (-20.1, -0.6)	417	-5.1 (-9.5, -0.7)
Non-response	1183	-0.2 (-0.8, 0.5)	565	-1.7 (-3.8, 0.4)	197	1.3 (-1.8, 4.4)	417	1.6 (-1.0, 4.2)
Mean score†	1170	*-0.283 (-0.341, -0.224)*	557	*-0.174 (-0.265, -0.083)*	193	*-0.225 (-0.454, 0.004)*	411	*-0.304 (-0.439, -0.169)*
In the next few years, there will be civil war in the United States.								
Do not agree (1)	1183	14.5 (10.4, 18.6)	565	12.9 (6.5, 19.3)	197	5.2 (-2.5, 12.9)	417	14.7 (7.8, 21.6)
Somewhat agree (2)	1183	-8.9 (-13.2, -4.6)	565	-9.2 (-16.2, -2.3)	197	1.2 (-10.0, 12.4)	417	-11.0 (-19.0, -3.1)
Strongly or very strongly agree (3)	1183	-3.5 (-5.1, -1.9)	565	-3.3 (-6.4, -0.3)	197	-5.7 (-15.4, 4.0)	417	-1.3 (-6.0, 3.4)
Non-response	1183	0.1 (-0.8, 1.1)	565	0.1 (-2.7, 2.8)	197	0.1 (-0.4, 0.6)	417	0.5 (-1.0, 2.0)
Mean score†	1164	*-0.230 (-0.284, -0.177)*	559	*-0.165 (-0.236, -0.094)*	193	*-0.126 (-0.275, 0.022)*	414	*-0.224 (-0.317, -0.131)*
The United States needs a civil war to set things right.								
Do not agree (1)	1183	11.1 (7.8, 14.4)	565	9.1 (3.5, 14.6)	197	4.5 (-3.0, 12.1)	417	5.2 (-1.1, 11.4)
Somewhat agree (2)	1183	-6.8 (-10.0, -3.6)	565	-8.2 (-13.2, -3.2)	197	7.2 (-3.0, 17.3)	417	-6.7 (-13.2, -0.2)
Strongly or very strongly agree (3)	1183	-1.5 (-3.3, 0.2)	565	-0.1 (-2.7, 2.6)	197	-4.7 (-10.6, 1.3)	417	0.3 (-2.8, 3.5)
Non-response	1183	-0.4 (-1.0, 0.2)	565	-1.0 (-4.2, 2.2)	197	-0.2 (-1.2, 0.8)	417	0.3 (-1.2, 1.9)
Mean score†	1172	*-0.169 (-0.222, -0.116)*	555	*-0.072 (-0.141, -0.002)*	194	*-0.227 (-0.392, -0.062)*	414	*-0.034 (-0.125, 0.057)*

Table S7, continued.

Statement	Mean Differences* by Party Category 2024-2025							
	No MAGA Affiliation							
	Non-MAGA, leans or not strong Republican (n = 1083)		Non-MAGA, Independent (n = 932)		Non-MAGA, leans or not strong Democrat (n = 1849)		Non-MAGA, strong Democrat (n = 1332)	
If elected leaders will not protect American democracy, the people must do it themselves, even if it requires taking violent actions.	**Unweighted n**	**Weighted mean (95% CI)** ***Mean score (95% CI)***	**Unweighted n**	**Weighted mean (95% CI)** ***Mean score (95% CI)***	**Unweighted n**	**Weighted mean (95% CI)** ***Mean score (95% CI)***	**Unweighted n**	**Weighted mean (95% CI)** ***Mean score (95% CI)***
Do not agree (1)	1083	2.5 (-1.4, 6.4)	932	2.7 (-1.7, 7.2)	1849	-9.7 (-12.8, -6.6)	1332	-13.1 (-16.9, -9.4)
Somewhat agree (2)	1083	-2.2 (-6.3, 1.8)	932	-3.2 (-8.0, 1.5)	1849	6.5 (3.3, 9.8)	1332	8.0 (4.5, 11.6)
Strongly or very strongly agree (3)	1083	0.4 (-1.7, 2.5)	932	2.1 (-0.4, 4.6)	1849	1.1 (-0.7, 2.8)	1332	2.0 (-0.4, 4.3)
Non-response	1083	0.3 (-0.9, 1.4)	932	0.7 (-0.9, 2.3)	1849	0.3 (-0.2, 0.8)	1332	0.5 (-0.7, 1.7)
Mean score†	1063	*-0.042 (-0.096, 0.013)*	899	*-0.045 (-0.119, 0.028)*	1833	*0.145 (0.104, 0.186)*	1318	*0.204 (0.140, 0.268)*
Our American way of life is disappearing so fast that we may have to use force to save it.								
Do not agree (1)	1083	4.3 (0.2, 8.5)	932	1.7 (-2.6, 6.0)	1849	-11.6 (-14.6, -8.5)	1332	-12.1 (-15.7, -8.5)
Somewhat agree (2)	1083	-2.6 (-7.0, 1.7)	932	0.4 (-4.2, 4.9)	1849	10.5 (7.1, 13.8)	1332	8.4 (5.0, 11.9)
Strongly or very strongly agree (3)	1083	-1.5 (-4.2, 1.1)	932	-0.3 (-3.0, 2.3)	1849	0.3 (-1.4, 1.9)	1332	0.2 (-2.1, 2.5)
Non-response	1083	0.4 (-0.7, 1.5)	932	0.7 (-0.9, 2.2)	1849	0.6 (0.1, 1.1)	1332	0.6 (-0.5, 1.7)
Mean score†	1069	*-0.070 (-0.137, -0.004)*	900	*-0.065 (-0.139, 0.008)*	1833	*0.121 (0.076, 0.166)*	1321	*0.178 (0.119, 0.236)*
Because things have gotten so far off track, true American patriots may have to resort to violence in order to save our country.								
Do not agree (1)	1083	5.0 (1.2, 8.8)	932	0.6 (-3.5, 4.7)	1849	-5.3 (-8.0, -2.7)	1332	-8.6 (-11.8, -5.4)
Somewhat agree (2)	1083	-3.0 (-6.7, 0.7)	932	1.1 (-2.9, 5.1)	1849	3.5 (0.8, 6.2)	1332	6.0 (2.9, 9.1)
Strongly or very strongly agree (3)	1083	-1.8 (-3.8, 0.1)	932	-1.7 (-4.4, 1.0)	1849	1.5 (-0.3, 3.2)	1332	0.5 (-1.7, 2.7)
Non-response	1083	0.2 (-1.0, 1.4)	932	1.0 (-0.4, 2.4)	1849	0.2 (-0.4, 0.7)	1332	1.0 (-0.1, 2.0)
Mean score†	1066	*-0.078 (-0.134, -0.022)*	904	*-0.041 (-0.110, 0.028)*	1833	*0.071 (0.033, 0.108)*	1319	*0.106 (0.049, 0.162)*
In the next few years, there will be civil war in the United States.								
Do not agree (1)	1083	0.8 (-2.9, 4.5)	932	-6.2 (-10.6, -1.8)	1849	-12.4 (-15.6, -9.3)	1332	-16.3 (-19.9, -12.6)
Somewhat agree (2)	1083	-0.3 (-4.2, 3.5)	932	3.9 (-0.8, 8.7)	1849	8.6 (5.3, 11.9)	1332	10.8 (6.7, 15.0)
Strongly or very strongly agree (3)	1083	0.0 (-2.2, 2.1)	932	0.8 (-2.1, 3.6)	1849	2.3 (0.6, 4.1)	1332	2.1 (0.1, 4.1)
Non-response	1083	0.4 (-0.6, 1.3)	932	0.9 (-0.6, 2.5)	1849	0.3 (0.0, 0.6)	1332	0.2 (-0.7, 1.1)
Mean score†	1071	*-0.025 (-0.076, 0.026)*	894	*0.089 (0.017, 0.160)*	1834	*0.170 (0.126, 0.214)*	1317	*0.251 (0.202, 0.301)*
The United States needs a civil war to set things right.								
Do not agree (1)	1083	2.4 (-0.4, 5.3)	932	-3.7 (-6.7, -0.6)	1849	-4.0 (-6.1, -1.9)	1332	-5.8 (-7.8, -3.9)
Somewhat agree (2)	1083	-1.4 (-3.9, 1.2)	932	2.8 (-0.4, 5.9)	1849	3.0 (1.0, 5.0)	1332	4.3 (2.3, 6.3)
Strongly or very strongly agree (3)	1083	-1.2 (-2.9, 0.6)	932	0.6 (-1.3, 2.6)	1849	0.6 (-0.6, 1.8)	1332	-0.6 (-2.0, 0.8)
Non-response	1083	0.4 (-0.7, 1.5)	932	0.7 (-0.9, 2.4)	1849	0.0 (-0.3, 0.2)	1332	0.3 (-0.6, 1.2)
Mean score†	1073	*-0.045 (-0.085, -0.006)*	895	*0.032 (-0.017, 0.081)*	1839	*0.054 (0.025, 0.084)*	1324	*0.087 (0.050, 0.124)*

* Among respondents to both the 2024 and 2025 surveys (n=7767).

† To assess population-level change from 2024 to 2025, we computed within-individual change scores for each item and then calculated year-to-year population-level change scores based on the means of aggregated within-individual change scores. Mean change scores have a range from -2 to 2 (with 0 indicating no change).

Table S8. Party/MAGA affiliation and 2025 prevalence of justification for political violence "in general" and to advance specific political objectives

**What do you think about the use of force or violence in the following situations?**	**Population Estimates by Party Category for 2025***							
	**MAGA Affiliation**						**No MAGA Affiliation**	
	**MAGA Republican (n = 1183)**		**MAGA Supporter, Republican (n = 565)**		**MAGA Supporter, Non-Republican (n = 197)**		**Non-MAGA, strong Republican (n = 417)**	
	**Unweighted n**	**Weighted % (95% CI)** ***Mean score (95% CI)***	**Unweighted n**	**Weighted % (95% CI)** ***Mean score (95% CI)***	**Unweighted n**	**Weighted % (95% CI)** ***Mean score (95% CI)***	**Unweighted n**	**Weighted % (95% CI)** ***Mean score (95% CI)***
In general...to advance an important political objective that you support								
Never justified (1)	900	75.1 (71.8, 78.4)	433	73.6 (68.2, 79.0)	144	67.6 (58.2, 77.0)	325	73.9 (67.7, 80.0)
Sometimes justified (2)	259	22.0 (18.9, 25.2)	128	25.6 (20.3, 31.0)	40	24.4 (15.8, 33.0)	81	20.7 (15.3, 26.0)
Usually or always justified (3)	21	2.7 (1.3, 4.0)	3	0.8 (0.0, 1.8)	11	7.3 (1.5, 13.1)	9	5.2 (1.1, 9.2)
Non-response	3	0.2 (0.0, 0.4)	1	0.0 (0.0, 0.1)	2	0.7 (0.0, 1.7)	2	0.3 (0.0, 0.7)
Mean score†	1180	*1.27 (1.23, 1.31)*	564	*1.27 (1.22, 1.33)*	195	*1.39 (1.26, 1.52)*	415	*1.31 (1.22, 1.40)*
aPD; q-value‡		*1.69 (-0.43, 3.82); 1.00*		*0.04 (-2.01, 2.09); 1.00*		*3.28 (-2.42, 8.97); 1.00*		*3.42 (-0.78, 7.62); 1.00*
Violence is usually or always justified to advance at least 1 of 20 objectives	631	52.2 (48.4, 56.1)	244	44.1 (38.3, 49.9)	92	46.3 (36.6, 56.1)	189	45.6 (39.1, 52.2)
aPD; q-value‡		*13.03 (7.69, 18.36); 0.0003*		*12.04 (5.13, 18.94); 0.06*		*5.32 (-4.99, 15.62); 1.00*		*10.51 (3.04, 17.99); 0.32*
To stop an election from being stolen§								
Never justified (1)	647	55.3 (51.5, 59.2)	357	63.4 (57.8, 69.0)	117	55.0 (45.1, 64.9)	290	67.1 (60.8, 73.4)
Sometimes justified (2)	337	27.6 (24.1, 31.0)	146	25.0 (20.0, 29.9)	49	27.5 (18.3, 36.6)	87	21.5 (15.9, 27.0)
Usually or always justified (3)	192	16.8 (13.8, 19.7)	59	10.5 (6.9, 14.1)	27	15.9 (8.1, 23.7)	36	10.6 (6.3, 14.9)
Non-response	4	0.3 (0.0, 0.7)	2	1.1 (0.0, 2.8)	2	1.6 (0.0, 4.6)	2	0.8 (0.0, 2.2)
Mean score†	1176	*1.61 (1.55, 1.67)*	562	*1.47 (1.39, 1.54)*	193	*1.60 (1.45, 1.76)*	413	*1.43 (1.34, 1.52)*
aPD; q-value‡		*8.60 (4.93, 12.26); 0.0007*		*3.76 (-0.80, 8.32); 1.00*		*4.90 (-2.94, 12.75); 1.00*		*3.57 (-1.35, 8.48); 1.00*
To stop people who do not share my beliefs from voting§								
Never justified (1)	1115	92.1 (89.5, 94.8)	541	92.9 (89.2, 96.5)	169	81.7 (72.9, 90.5)	389	90.9 (86.4, 95.3)
Sometimes justified (2)	42	4.5 (2.4, 6.7)	14	2.8 (0.9, 4.7)	9	7.4 (1.0, 13.9)	17	4.8 (1.5, 8.1)
Usually or always justified (3)	21	3.2 (1.6, 4.8)	7	3.3 (0.5, 6.1)	15	9.2 (2.9, 15.6)	8	3.7 (0.7, 6.7)
Non-response	2	0.1 (0.0, 0.3)	2	1.1 (0.0, 2.8)	2	1.6 (0.0, 4.6)	1	0.6 (0.0, 1.9)
Mean score†	1178	*1.11 (1.07, 1.15)*	562	*1.09 (1.04, 1.15)*	193	*1.26 (1.13, 1.40)*	414	*1.12 (1.06, 1.19)*
aPD; q-value‡		*1.42 (-1.09, 3.92); 1.00*		*1.93 (-1.53, 5.38); 1.00*		*4.55 (-1.98, 11.08); 1.00*		*1.94 (-1.60, 5.49); 1.00*
To prevent discrimination based on race or ethnicity§								
Never justified (1)	844	69.5 (65.9, 73.2)	395	68.7 (63.0, 74.3)	128	55.6 (45.7, 65.5)	311	71.1 (64.9, 77.3)
Sometimes justified (2)	246	21.8 (18.6, 25.1)	135	24.4 (19.1, 29.7)	43	23.3 (15.0, 31.7)	78	20.4 (14.8, 26.0)
Usually or always justified (3)	88	8.5 (6.1, 10.9)	31	5.6 (3.0, 8.3)	23	19.5 (10.6, 28.4)	25	7.8 (4.1, 11.6)
Non-response	2	0.2 (0.0, 0.4)	3	1.3 (0.0, 3.1)	1	1.5 (0.0, 4.5)	1	0.6 (0.0, 1.9)
Mean score†	1178	*1.39 (1.34, 1.44)*	561	*1.36 (1.29, 1.43)*	194	*1.63 (1.46, 1.80)*	414	*1.36 (1.28, 1.45)*
aPD; q-value‡		*-1.18 (-4.91, 2.55); 1.00*		*-3.30 (-7.26, 0.65); 1.00*		*4.19 (-4.78, 13.15); 1.00*		*-1.97 (-6.81, 2.86); 1.00*

Table S8, continued.

What do you think about the use of force or violence in the following situations?	Population Estimates by Party Category for 2025*							
	MAGA Affiliation						No MAGA Affiliation	
	MAGA Republican (n = 1183)		MAGA Supporter, Republican (n = 565)		MAGA Supporter, Non-Republican (n = 197)		Non-MAGA, strong Republican (n = 417)	
	Unweighted n	Weighted % (95% CI) *Mean score (95% CI)*	Unweighted n	Weighted % (95% CI) *Mean score (95% CI)*	Unweighted n	Weighted % (95% CI) *Mean score (95% CI)*	Unweighted n	Weighted % (95% CI) *Mean score (95% CI)*
To preserve an American way of life based on Western European traditions§								
Never justified (1)	662	59.2 (55.5, 63.0)	346	64.1 (58.7, 69.6)	128	64.0 (54.3, 73.8)	276	65.9 (59.7, 72.2)
Sometimes justified (2)	385	30.1 (26.8, 33.5)	169	26.3 (21.4, 31.2)	47	25.0 (16.0, 34.0)	113	26.4 (20.6, 32.2)
Usually or always justified (3)	130	10.5 (8.0, 13.0)	45	8.1 (5.0, 11.2)	18	9.3 (3.4, 15.2)	24	6.9 (3.4, 10.3)
Non-response	3	0.1 (0.0, 0.3)	4	1.5 (0.0, 3.3)	2	1.6 (0.0, 4.6)	2	0.8 (0.0, 2.2)
Mean score†	1177	*1.51 (1.46, 1.57)*	560	*1.43 (1.36, 1.50)*	193	*1.44 (1.31, 1.58)*	413	*1.40 (1.32, 1.49)*
aPD; q-value‡		*7.19 (3.97, 10.41); 0.002*		*5.64 (1.92, 9.35); 0.19*		*3.27 (-3.37, 9.91); 1.00*		*3.93 (-0.05, 7.9); 1.00*
To preserve an American way of life I believe in§								
Never justified (1)	516	46.8 (42.9, 50.7)	279	52.8 (47.0, 58.5)	95	49.7 (40.0, 59.4)	234	56.6 (50.0, 63.1)
Sometimes justified (2)	448	34.1 (30.6, 37.5)	226	36.1 (30.7, 41.6)	64	26.1 (17.6, 34.6)	136	31.1 (25.0, 37.2)
Usually or always justified (3)	212	18.8 (15.6, 22.0)	57	10.0 (6.6, 13.4)	34	22.6 (13.7, 31.4)	42	10.8 (6.9, 14.7)
Non-response	4	0.3 (0.0, 0.7)	2	1.1 (0.0, 2.8)	2	1.6 (0.0, 4.6)	3	1.6 (0.0, 3.5)
Mean score†	1176	*1.72 (1.66, 1.78)*	562	*1.57 (1.49, 1.64)*	193	*1.72 (1.56, 1.89)*	412	*1.53 (1.45, 1.62)*
aPD; q-value‡		*11.33 (7.47, 15.18); <0.0001*		*4.44 (0.15, 8.74); 1.00*		*13.32 (4.14, 22.5); 0.27*		*4.72 (0.18, 9.26); 1.00*
To oppose Americans who do not share my beliefs§								
Never justified (1)	1067	88.5 (85.9, 91.2)	527	89.7 (85.1, 94.2)	161	72.7 (62.9, 82.6)	373	87.7 (82.8, 92.6)
Sometimes justified (2)	83	6.8 (5.0, 8.6)	33	9.0 (4.7, 13.3)	17	16.2 (7.2, 25.2)	32	7.3 (3.7, 10.9)
Usually or always justified (3)	28	4.4 (2.4, 6.5)	2	0.3 (0.0, 0.6)	15	9.4 (3.5, 15.3)	7	3.5 (0.3, 6.6)
Non-response	2	0.3 (0.0, 0.6)	2	1.1 (0.0, 2.8)	2	1.6 (0.0, 4.6)	3	1.6 (0.0, 3.5)
Mean score†	1178	*1.16 (1.11, 1.20)*	562	*1.10 (1.05, 1.14)*	193	*1.36 (1.22, 1.49)*	412	*1.14 (1.07, 1.21)*
aPD; q-value‡		*3.32 (0.68, 5.96); 0.60*		*-0.79 (-2.39, 0.80); 1.00*		*5.13 (-1.38, 11.63); 1.00*		*2.28 (-1.13, 5.68); 1.00*
To oppose the government when it does not share my beliefs§								
Never justified (1)	957	80.6 (77.4, 83.7)	473	80.6 (75.5, 85.7)	149	73.2 (63.9, 82.6)	348	80.3 (74.8, 85.9)
Sometimes justified (2)	179	14.2 (11.6, 16.7)	76	14.3 (9.9, 18.6)	30	13.9 (7.1, 20.7)	52	14.1 (9.2, 19.1)
Usually or always justified (3)	41	5.1 (3.0, 7.1)	13	4.0 (1.2, 6.9)	14	11.2 (3.8, 18.7)	14	4.9 (1.8, 7.9)
Non-response	3	0.2 (0.0, 0.5)	2	1.1 (0.0, 2.8)	2	1.6 (0.0, 4.6)	1	0.6 (0.0, 1.9)
Mean score†	1177	*1.24 (1.20, 1.29)*	562	*1.23 (1.16, 1.29)*	193	*1.37 (1.22, 1.52)*	414	*1.24 (1.17, 1.31)*
aPD; q-value‡		*2.01 (-0.95, 4.98); 1.00*		*1.08 (-2.48, 4.65); 1.00*		*5.09 (-2.64, 12.81); 1.00*		*1.64 (-2.12, 5.4); 1.00*
To oppose the government when it tries to take private land for public purposes§								
Never justified (1)	634	54.3 (50.4, 58.1)	310	52.7 (46.9, 58.5)	98	50.3 (40.6, 60.0)	261	64.2 (58.0, 70.5)
Sometimes justified (2)	399	30.9 (27.5, 34.3)	207	33.8 (28.6, 39.0)	70	36.5 (26.9, 46.1)	120	26.1 (20.5, 31.7)
Usually or always justified (3)	144	14.6 (11.6, 17.7)	45	12.4 (7.7, 17.1)	25	11.5 (5.9, 17.1)	33	9.0 (5.1, 12.9)
Non-response	3	0.2 (0.0, 0.5)	2	1.1 (0.0, 2.8)	2	1.7 (0.0, 4.7)	1	0.6 (0.0, 1.9)
Mean score†	1177	*1.60 (1.54, 1.66)*	562	*1.59 (1.50, 1.68)*	193	*1.61 (1.48, 1.73)*	414	*1.44 (1.36, 1.53)*
aPD; q-value‡		*6.40 (2.29, 10.50); 0.16*		*5.33 (0.15, 10.51); 1.00*		*0.86 (-5.60, 7.33); 1.00*		*1.17 (-3.62, 5.95); 1.00*

Table S8, continued.

What do you think about the use of force or violence in the following situations?	Population Estimates by Party Category for 2025*							
	No MAGA Affiliation							
	Non-MAGA, leans or not strong Republican (n = 1083)		Non-MAGA, Independent (n = 932)		Non-MAGA, leans or not strong Democrat (n = 1849)		Non-MAGA, strong Democrat (n = 1332)	
	Unweighted n	Weighted % (95% CI) *Mean score (95% CI)*	Unweighted n	Weighted % (95% CI) *Mean score (95% CI)*	Unweighted n	Weighted % (95% CI) *Mean score (95% CI)*	Unweighted n	Weighted % (95% CI) *Mean score (95% CI)*
In general...to advance an important political objective that you support								
Never justified (1)	893	80.7 (77.3, 84.0)	727	74.7 (70.8, 78.6)	1438	74.2 (71.4, 76.9)	1048	73.7 (70.4, 77.1)
Sometimes justified (2)	181	18.3 (15.1, 21.6)	178	20.4 (16.9, 23.9)	383	23.1 (20.5, 25.7)	267	23.8 (20.6, 27.0)
Usually or always justified (3)	7	0.7 (0.0, 1.5)	20	3.7 (1.8, 5.6)	25	2.5 (1.4, 3.5)	13	2.0 (0.8, 3.3)
Non-response	2	0.3 (0.0, 0.8)	7	1.2 (0.0, 2.4)	3	0.3 (0.0, 0.6)	4	0.4 (0.0, 0.9)
Mean score†	1081	*1.20 (1.16, 1.23)*	925	*1.28 (1.23, 1.33)*	1846	*1.28 (1.25, 1.31)*	1328	*1.28 (1.24, 1.32)*
aPD; q-value‡		*-0.32 (-2.06, 1.43); 1.00*		*1.32 (-1.24, 3.88); 1.00*		*0.07 (-1.73, 1.86); 1.00*		*Reference*
Violence is usually or always justified to advance at least 1 of 20 objectives	331	29.7 (26.0, 33.5)	316	34.5 (30.4, 38.5)	478	27.0 (24.2, 29.8)	370	32.1 (28.6, 35.6)
aPD; q-value‡		*0.84 (-4.13, 5.81); 1.00*		*-0.51 (-5.78, 4.76); 1.00*		*-4.86 (-9.11, -0.61); 0.92*		*Reference*
To stop an election from being stolen§								
Never justified (1)	830	77.6 (74.4, 80.8)	696	76.0 (72.4, 79.7)	1423	76.5 (73.9, 79.0)	1033	75.2 (72.0, 78.5)
Sometimes justified (2)	179	15.8 (13.0, 18.7)	155	14.9 (12.0, 17.9)	305	16.6 (14.4, 18.9)	186	14.8 (12.2, 17.5)
Usually or always justified (3)	67	5.8 (4.1, 7.6)	64	7.7 (5.2, 10.1)	112	6.7 (5.1, 8.3)	103	9.1 (6.8, 11.3)
Non-response	5	0.7 (0.0, 1.4)	10	1.4 (0.5, 2.3)	6	0.2 (0.0, 0.4)	6	0.8 (0.1, 1.6)
Mean score†	1076	*1.28 (1.23, 1.32)*	915	*1.31 (1.25, 1.36)*	1840	*1.30 (1.26, 1.34)*	1322	*1.33 (1.28, 1.38)*
aPD; q-value‡		*-0.59 (-3.53, 2.35); 1.00*		*-1.45 (-4.87, 1.97); 1.00*		*-1.42 (-4.20, 1.36); 1.00*		*Reference*
To stop people who do not share my beliefs from voting§								
Never justified (1)	1045	95.5 (93.6, 97.3)	858	88.4 (85.3, 91.5)	1780	95.2 (93.8, 96.6)	1277	93.4 (91.1, 95.7)
Sometimes justified (2)	23	2.8 (1.3, 4.3)	36	6.4 (3.9, 8.9)	34	2.2 (1.4, 3.1)	27	3.2 (1.6, 4.8)
Usually or always justified (3)	10	1.5 (0.4, 2.6)	22	3.9 (2.0, 5.8)	28	2.4 (1.3, 3.5)	22	3.3 (1.6, 5.0)
Non-response	3	0.3 (0.0, 0.6)	9	1.3 (0.4, 2.3)	4	0.2 (0.0, 0.3)	2	0.1 (0.0, 0.3)
Mean score†	1078	*1.06 (1.03, 1.08)*	916	*1.14 (1.10, 1.19)*	1842	*1.07 (1.05, 1.09)*	1326	*1.10 (1.06, 1.14)*
aPD; q-value‡		*-0.25 (-2.41, 1.90); 1.00*		*0.04 (-2.64, 2.72); 1.00*		*-0.89 (-2.92, 1.14); 1.00*		*Reference*
To prevent discrimination based on race or ethnicity§								
Never justified (1)	810	75.1 (71.6, 78.6)	619	65.2 (61.1, 69.3)	1187	60.4 (57.4, 63.4)	856	58.9 (55.3, 62.5)
Sometimes justified (2)	222	19.9 (16.7, 23.0)	218	23.5 (19.9, 27.2)	510	29.4 (26.6, 32.2)	320	27.9 (24.6, 31.3)
Usually or always justified (3)	47	4.8 (2.9, 6.7)	77	9.4 (6.8, 12.1)	142	10.0 (8.0, 11.9)	148	12.9 (10.3, 15.4)
Non-response	2	0.2 (0.0, 0.5)	11	1.8 (0.7, 3.0)	7	0.2 (0.0, 0.4)	4	0.3 (0.0, 0.6)
Mean score†	1079	*1.30 (1.25, 1.34)*	914	*1.43 (1.37, 1.49)*	1839	*1.49 (1.45, 1.54)*	1324	*1.54 (1.49, 1.59)*
aPD; q-value‡		*-4.73 (-8.06, -1.39); 0.30*		*-4.09 (-7.92, -0.26); 1.00*		*-2.67 (-5.93, 0.59); 1.00*		*Reference*

Table S8, continued.

What do you think about the use of force or violence in the following situations?	Population Estimates by Party Category for 2025*							
	No MAGA Affiliation							
	Non-MAGA, leans or not strong Republican (n = 1083)		Non-MAGA, Independent (n = 932)		Non-MAGA, leans or not strong Democrat (n = 1849)		Non-MAGA, strong Democrat (n = 1332)	
	Unweighted n	Weighted % (95% CI) *Mean score (95% CI)*	Unweighted n	Weighted % (95% CI) *Mean score (95% CI)*	Unweighted n	Weighted % (95% CI) *Mean score (95% CI)*	Unweighted n	Weighted % (95% CI) *Mean score (95% CI)*
To preserve an American way of life based on Western European traditions§								
Never justified (1)	808	77.3 (74.0, 80.5)	704	76.0 (72.3, 79.7)	1560	84.8 (82.6, 87.0)	1130	83.9 (81.1, 86.8)
Sometimes justified (2)	229	18.6 (15.6, 21.6)	177	17.8 (14.6, 21.0)	233	11.5 (9.5, 13.4)	144	10.9 (8.4, 13.3)
Usually or always justified (3)	39	3.5 (2.0, 4.9)	30	4.3 (2.3, 6.3)	45	3.4 (2.2, 4.5)	47	4.8 (3.1, 6.4)
Non-response	5	0.7 (0.0, 1.4)	14	1.9 (0.8, 3.0)	8	0.3 (0.1, 0.6)	7	0.4 (0.0, 0.8)
Mean score†	1076	*1.26 (1.22, 1.30)*	911	*1.27 (1.22, 1.32)*	1838	*1.18 (1.15, 1.21)*	1321	*1.21 (1.17, 1.24)*
aPD; q-value‡	*0.63 (-1.65, 2.90); 1.00*		*-0.38 (-3.06, 2.31); 1.00*		*-1.09 (-3.14, 0.96); 1.00*			*Reference*
To preserve an American way of life I believe in§								
Never justified (1)	690	67.2 (63.6, 70.9)	600	67.9 (64.0, 71.8)	1314	74.0 (71.4, 76.6)	972	71.7 (68.3, 75.0)
Sometimes justified (2)	297	24.0 (20.7, 27.3)	240	22.9 (19.4, 26.5)	432	19.4 (17.2, 21.7)	257	20.5 (17.4, 23.6)
Usually or always justified (3)	87	8.0 (5.9, 10.1)	71	7.2 (5.0, 9.4)	93	6.2 (4.6, 7.9)	96	7.6 (5.7, 9.5)
Non-response	7	0.8 (0.0, 1.5)	14	2.0 (0.8, 3.1)	7	0.3 (0.1, 0.6)	3	0.2 (0.0, 0.5)
Mean score†	1074	*1.40 (1.35, 1.45)*	911	*1.38 (1.33, 1.43)*	1839	*1.32 (1.28, 1.36)*	1325	*1.36 (1.31, 1.40)*
aPD; q-value‡	*2.54 (-0.51, 5.59); 1.00*		*-0.91 (-4.02, 2.21); 1.00*		*-1.11 (-3.65, 1.43); 1.00*			*Reference*
To oppose Americans who do not share my beliefs§								
Never justified (1)	1014	92.5 (90.3, 94.7)	829	86.5 (83.4, 89.6)	1726	91.3 (89.5, 93.2)	1239	90.1 (87.6, 92.7)
Sometimes justified (2)	52	5.0 (3.3, 6.6)	62	8.5 (5.9, 11.0)	88	5.9 (4.4, 7.4)	59	7.1 (4.8, 9.4)
Usually or always justified (3)	10	1.9 (0.5, 3.2)	22	3.3 (1.6, 5.0)	28	2.6 (1.4, 3.8)	29	2.7 (1.4, 3.9)
Non-response	5	0.7 (0.0, 1.4)	12	1.7 (0.6, 2.8)	4	0.2 (0.0, 0.4)	1	0.1 (0.0, 0.3)
Mean score†	1076	*1.09 (1.06, 1.12)*	913	*1.15 (1.11, 1.20)*	1842	*1.11 (1.08, 1.14)*	1327	*1.12 (1.09, 1.16)*
aPD; q-value‡	*0.56 (-1.44, 2.56); 1.00*		*0.03 (-2.20, 2.26); 1.00*		*-0.43 (-2.18, 1.33); 1.00*			*Reference*
To oppose the government when it does not share my beliefs§								
Never justified (1)	948	88.1 (85.6, 90.6)	742	77.5 (73.8, 81.2)	1570	82.4 (79.9, 84.8)	1130	79.8 (76.5, 83.1)
Sometimes justified (2)	117	10.4 (8.0, 12.8)	140	15.7 (12.4, 18.9)	224	13.6 (11.5, 15.8)	159	15.5 (12.5, 18.5)
Usually or always justified (3)	12	1.2 (0.4, 1.9)	30	4.9 (2.9, 7.0)	44	3.6 (2.3, 5.0)	35	4.4 (2.6, 6.2)
Non-response	4	0.3 (0.0, 0.6)	13	1.9 (0.8, 3.0)	8	0.4 (0.1, 0.7)	4	0.3 (0.0, 0.7)
Mean score†	1077	*1.13 (1.10, 1.16)*	912	*1.26 (1.21, 1.31)*	1838	*1.21 (1.18, 1.24)*	1324	*1.24 (1.20, 1.29)*
aPD; q-value‡	*-1.69 (-3.79, 0.41); 1.00*		*-0.10 (-2.92, 2.73); 1.00*		*-0.98 (-3.22, 1.25); 1.00*			*Reference*
To oppose the government when it tries to take private land for public purposes§								
Never justified (1)	690	66.0 (62.3, 69.7)	567	62.2 (58.1, 66.4)	1215	63.7 (60.8, 66.7)	945	68.0 (64.6, 71.4)
Sometimes justified (2)	318	26.8 (23.5, 30.2)	253	25.1 (21.4, 28.8)	525	28.9 (26.2, 31.7)	282	22.9 (19.8, 26.0)
Usually or always justified (3)	68	6.5 (4.4, 8.6)	94	11.2 (8.4, 14.0)	100	7.0 (5.4, 8.7)	95	8.8 (6.5, 11.1)
Non-response	5	0.7 (0.0, 1.4)	11	1.5 (0.5, 2.4)	6	0.3 (0.0, 0.5)	6	0.3 (0.0, 0.6)
Mean score†	1076	*1.40 (1.35, 1.45)*	914	*1.48 (1.42, 1.54)*	1840	*1.43 (1.39, 1.47)*	1322	*1.41 (1.36, 1.46)*
aPD; q-value‡	*-0.85 (-4.17, 2.47); 1.00*		*0.84 (-2.95, 4.63); 1.00*		*-2.09 (-5.04, 0.86); 1.00*			*Reference*

* Among respondents to both the 2024 and 2025 surveys (n=7767). Prevalences for 2024 among respondents to that survey were reported previously: Wintemute GJ, Velasquez B, Robinson SL, Tomsich EA, Wright MA, Shev AB. The MAGA movement and political violence in 2024: findings from a nationally representative survey. Inj Epidemiol. 2025;12(1):78.

† Mean scores in 2024 and 2025 were scored as indicated in the response lines for individual questions, with non-responses excluded.

‡ Adjusted prevalence differences (aPDs) are absolute percentage point (pp) differences for “usually or always justified” responses and are adjusted for age, race and ethnicity, gender, income, education, Census division, marital status, homeownership, rurality, firearm ownership, alcohol consumption, military service, and history of non-traffic arrest. Q-values, also known as FDR-adjusted (or FDR-corrected) p-values, represent the probability that the given difference would be a false discovery; they represent the expected proportion of “false positives” that would be seen among the collection of all differences whose q-values were at or below the given q-value. Item non-responses are not reported in the tables but are included in the prevalence calculations.

§ Respondents who did not answer the question "In general...to advance an important political objective that you support" in 2024 (n =58) or 2025 (n = 57) were not asked these questions.

Table S9. Party/MAGA affiliation and 2024-2025 change in justification for political violence “in general” and to advance specific political objectives

What do you think about the use of force or violence in the following situations?	Mean Differences* by Party Category 2024-2025							
	MAGA Affiliation						No MAGA Affiliation	
	MAGA Republican (n = 1183)		MAGA Supporter, Republican (n = 565)		MAGA Supporter, Non-Republican (n = 197)		Non-MAGA, strong Republican (n = 417)	
	Unweighted n	Weighted mean (95% CI) *Mean score (95% CI)*	Unweighted n	Weighted mean (95% CI) *Mean score (95% CI)*	Unweighted n	Weighted mean (95% CI) *Mean score (95% CI)*	Unweighted n	Weighted mean (95% CI) *Mean score (95% CI)*
In general...to advance an important political objective that you support								
Never justified (1)	1183	3.9 (0.2, 7.6)	565	-0.5 (-5.5, 4.5)	197	1.5 (-8.6, 11.7)	417	-4.6 (-10.8, 1.6)
Sometimes justified (2)	1183	-3.4 (-6.9, 0.2)	565	0.8 (-4.3, 5.8)	197	1.2 (-9.1, 11.6)	417	0.7 (-6.0, 7.3)
Usually or always justified (3)	1183	-0.3 (-2.0, 1.5)	565	0.2 (-0.9, 1.2)	197	-1.7 (-5.2, 1.8)	417	3.2 (-1.1, 7.5)
Non-response	1183	0.1 (-0.2, 0.4)	565	-0.1 (-0.3, 0.1)	197	0.2 (-0.2, 0.6)	417	0.1 (-0.1, 0.4)
Mean score†	1179	*-0.049 (-0.095, -0.002)*	563	*0.001 (-0.060, 0.062)*	195	*-0.060 (-0.183, 0.064)*	415	*0.090 (0.003, 0.177)*
Violence is usually or always justified to advance at least 1 of 20 objectives	1179	-0.024 (-0.069, 0.022)	563	0.007 (-0.057, 0.072)	195	-0.039 (-0.144, 0.067)	415	0.025 (-0.049, 0.099)
To stop an election from being stolen‡								
Never justified (1)	1179	-2.6 (-6.9, 1.7)	563	0.6 (-4.8, 6.1)	195	-12.7 (-24.7, -0.7)	415	-3.2 (-10.2, 3.8)
Sometimes justified (2)	1179	3.0 (-1.2, 7.3)	563	-1.8 (-8.3, 4.7)	195	12.1 (0.7, 23.5)	415	2.7 (-4.3, 9.7)
Usually or always justified (3)	1179	0.5 (-1.8, 2.8)	563	1.5 (-3.5, 6.5)	195	1.1 (-7.1, 9.2)	415	-0.1 (-4.7, 4.5)
Non-response	1179	-1.1 (-2.2, 0.1)	563	0.2 (-0.2, 0.6)	195	0.9 (-2.3, 4.2)	415	0.8 (-0.5, 2.2)
Mean score†	1167	*0.045 (-0.030, 0.120)*	561	*-0.004 (-0.106, 0.098)*	192	*0.102 (-0.115, 0.320)*	413	*0.024 (-0.115, 0.162)*
To stop people who do not share my beliefs from voting‡								
Never justified (1)	1179	2.3 (-0.3, 5.0)	563	-3.1 (-6.6, 0.4)	195	-1.9 (-11.0, 7.2)	415	-0.5 (-4.0, 3.0)
Sometimes justified (2)	1179	-0.4 (-3.4, 2.5)	563	-0.3 (-2.3, 1.8)	195	0.4 (-9.5, 10.2)	415	0.3 (-2.0, 2.6)
Usually or always justified (3)	1179	0.0 (-2.5, 2.5)	563	3.1 (0.3, 5.9)	195	2.2 (-3.1, 7.4)	415	-0.5 (-3.3, 2.2)
Non-response	1179	-1.1 (-2.2, 0.1)	563	0.2 (-0.2, 0.6)	195	0.9 (-2.3, 4.2)	415	0.6 (-0.6, 1.9)
Mean score†	1171	*-0.050 (-0.094, -0.006)*	561	*0.060 (0.000, 0.121)*	192	*0.001 (-0.108, 0.110)*	414	*-0.004 (-0.064, 0.055)*
To prevent discrimination based on race or ethnicity‡								
Never justified (1)	1179	-4.1 (-8.8, 0.7)	563	-6.2 (-12.3, -0.1)	195	-6.5 (-17.3, 4.2)	415	-4.6 (-11.0, 1.9)
Sometimes justified (2)	1179	3.7 (-0.6, 8.0)	563	3.8 (-1.9, 9.4)	195	5.6 (-3.7, 14.8)	415	3.6 (-2.6, 9.8)
Usually or always justified (3)	1179	0.8 (-1.2, 2.8)	563	1.9 (-0.6, 4.3)	195	-1.3 (-4.8, 2.2)	415	-1.2 (-5.1, 2.7)
Non-response	1179	-0.3 (-0.9, 0.2)	563	0.5 (-0.2, 1.1)	195	0.9 (-2.4, 4.1)	415	0.6 (-0.6, 1.9)
Mean score†	1172	*0.050 (-0.039, 0.139)*	560	*0.079 (-0.004, 0.162)*	193	*0.073 (-0.135, 0.280)*	414	*0.059 (-0.052, 0.169)*
To preserve an American way of life based on Western European traditions‡								
Never justified (1)	1179	0.7 (-4.0, 5.5)	563	3.3 (-2.5, 9.1)	195	-0.2 (-10.7, 10.3)	415	-1.8 (-8.3, 4.7)
Sometimes justified (2)	1179	1.2 (-2.9, 5.3)	563	-1.1 (-7.0, 4.9)	195	0.1 (-10.5, 10.7)	415	4.7 (-1.9, 11.4)
Usually or always justified (3)	1179	0.6 (-2.7, 3.9)	563	-2.8 (-6.9, 1.3)	195	2.3 (-3.6, 8.2)	415	-2.4 (-7.1, 2.3)
Non-response	1179	-0.5 (-1.1, 0.1)	563	0.4 (-0.4, 1.2)	195	0.8 (-2.5, 4.1)	415	0.2 (-0.2, 0.6)
Mean score†	1170	*-0.038 (-0.118, 0.041)*	558	*-0.061 (-0.176, 0.053)*	191	*-0.049 (-0.206, 0.109)*	412	*-0.024 (-0.143, 0.096)*

Table S9, continued.

What do you think about the use of force or violence in the following situations?	Mean Differences* by Party Category 2024-2025							
	MAGA Affiliation						No MAGA Affiliation	
	MAGA Republican (n = 1183)		MAGA Supporter, Republican (n = 565)		MAGA Supporter, Non-Republican (n = 197)		Non-MAGA, strong Republican (n = 417)	
	Unweighted n	Weighted mean (95% CI) *Mean score (95% CI)*	Unweighted n	Weighted mean (95% CI) *Mean score (95% CI)*	Unweighted n	Weighted mean (95% CI) *Mean score (95% CI)*	Unweighted n	Weighted mean (95% CI) *Mean score (95% CI)*
To preserve an American way of life I believe in‡								
Never justified (1)	1179	-1.5 (-6.1, 3.0)	563	-0.8 (-6.0, 4.5)	195	-4.8 (-16.5, 7.0)	415	-1.1 (-8.2, 6.1)
Sometimes justified (2)	1179	0.0 (-4.1, 4.1)	563	1.8 (-3.7, 7.3)	195	4.3 (-5.5, 14.0)	415	0.1 (-6.4, 6.5)
Usually or always justified (3)	1179	2.0 (-1.2, 5.2)	563	-2.8 (-6.3, 0.7)	195	-0.7 (-7.6, 6.2)	415	-0.3 (-4.7, 4.2)
Non-response	1179	-0.2 (-0.8, 0.4)	563	-0.1 (-0.7, 0.5)	195	1.5 (-1.5, 4.5)	415	0.9 (-1.2, 3.1)
Mean score†	1170	*0.029 (-0.054, 0.111)*	559	*0.019 (-0.073, 0.110)*	192	*0.019 (-0.257, 0.296)*	410	*0.010 (-0.102, 0.122)*
To oppose Americans who do not share my beliefs‡								
Never justified (1)	1179	1.7 (-1.7, 5.2)	563	-2.6 (-6.8, 1.6)	195	-5.9 (-15.3, 3.5)	415	2.7 (-2.7, 8.0)
Sometimes justified (2)	1179	-2.0 (-5.0, 1.1)	563	3.0 (-1.3, 7.3)	195	6.0 (-5.0, 17.0)	415	-2.6 (-8.0, 2.8)
Usually or always justified (3)	1179	0.9 (-1.2, 2.9)	563	0.3 (-0.1, 0.6)	195	-2.7 (-9.2, 3.8)	415	-1.3 (-5.1, 2.5)
Non-response	1179	0.0 (-0.5, 0.5)	563	0.1 (-0.4, 0.6)	195	1.5 (-1.5, 4.5)	415	1.2 (-0.9, 3.3)
Mean score†	1175	*-0.022 (-0.073, 0.030)*	560	*0.013 (-0.037, 0.063)*	192	*0.042 (-0.122, 0.205)*	411	*-0.052 (-0.124, 0.019)*
To oppose the government when it does not share my beliefs‡								
Never justified (1)	1179	8.8 (4.9, 12.7)	563	3.2 (-1.3, 7.6)	195	1.3 (-7.4, 10.0)	415	-1.0 (-6.4, 4.5)
Sometimes justified (2)	1179	-5.8 (-9.5, -2.0)	563	-4.0 (-8.6, 0.5)	195	0.0 (-9.1, 9.1)	415	1.9 (-3.8, 7.5)
Usually or always justified (3)	1179	0.4 (-2.2, 3.0)	563	2.2 (-0.4, 4.8)	195	-2.3 (-8.0, 3.5)	415	0.4 (-3.1, 4.0)
Non-response	1179	-1.0 (-2.0, 0.0)	563	0.2 (-0.2, 0.6)	195	0.8 (-2.5, 4.1)	415	0.6 (-0.6, 1.9)
Mean score†	1166	*-0.126 (-0.187, -0.065)*	561	*-0.043 (-0.112, 0.026)*	191	*-0.046 (-0.156, 0.064)*	414	*-0.032 (-0.137, 0.072)*
To oppose the government when it tries to take private land for public purposes‡								
Never justified (1)	1179	8.3 (3.7, 12.9)	563	0.8 (-5.4, 7.0)	195	-4.8 (-15.5, 6.0)	415	2.5 (-4.6, 9.6)
Sometimes justified (2)	1179	-4.0 (-8.6, 0.5)	563	-0.4 (-6.4, 5.7)	195	14.9 (3.2, 26.5)	415	-0.4 (-7.3, 6.6)
Usually or always justified (3)	1179	-3.9 (-7.7, 0.0)	563	-2.2 (-5.9, 1.5)	195	-7.1 (-13.8, -0.4)	415	-3.5 (-8.1, 1.2)
Non-response	1179	-0.7 (-1.5, 0.1)	563	0.2 (-0.2, 0.6)	195	1.0 (-2.3, 4.3)	415	0.6 (-0.6, 1.9)
Mean score†	1169	*-0.113 (-0.203, -0.022)*	561	*-0.002 (-0.090, 0.085)*	192	*-0.099 (-0.271, 0.073)*	413	*-0.045 (-0.171, 0.082)*

Table S9, continued.

What do you think about the use of force or violence in the following situations?	**Mean Differences* by Party Category 2024-2025**							
	**No MAGA Affiliation**							
	**Non-MAGA, leans or not strong Republican (n = 1083)**		**Non-MAGA, Independent (n = 932)**		**Non-MAGA, leans or not strong Democrat (n = 1849)**		**Non-MAGA, strong Democrat (n = 1332)**	
	**Unweighted n**	**Weighted mean (95% CI)** ***Mean score (95% CI)***	**Unweighted n**	**Weighted mean (95% CI)** ***Mean score (95% CI)***	**Unweighted n**	**Weighted mean (95% CI)** ***Mean score (95% CI)***	**Unweighted n**	**Weighted mean (95% CI)** ***Mean score (95% CI)***
In general...to advance an important political objective that you support								
Never justified (1)	1083	-1.8 (-5.0, 1.4)	932	-1.3 (-5.9, 3.3)	1849	-6.0 (-8.7, -3.2)	1332	-7.7 (-11.2, -4.2)
Sometimes justified (2)	1083	2.3 (-1.0, 5.5)	932	0.8 (-3.6, 5.1)	1849	4.6 (2.0, 7.3)	1332	7.1 (3.7, 10.4)
Usually or always justified (3)	1083	0.3 (-0.5, 1.2)	932	1.0 (-1.3, 3.4)	1849	0.8 (-0.5, 2.0)	1332	1.0 (-0.4, 2.3)
Non-response	1083	-0.7 (-1.7, 0.2)	932	0.1 (-1.2, 1.5)	1849	0.2 (-0.2, 0.5)	1332	0.4 (-0.1, 0.9)
Mean score†	1078	*0.023 (-0.010, 0.057)*	920	*0.006 (-0.054, 0.066)*	1845	*0.073 (0.038, 0.107)*	1326	*0.068 (0.024, 0.112)*
Violence is usually or always justified to advance at least 1 of 20 objectives	1078	0.020 (-0.020, 0.059)	920	0.032 (-0.012, 0.076)	1845	0.044 (0.016, 0.071)	1326	0.079 (0.045, 0.113)
To stop an election from being stolen‡								
Never justified (1)	1078	-1.7 (-4.9, 1.6)	920	-2.7 (-7.1, 1.7)	1845	-9.0 (-11.7, -6.3)	1326	-9.1 (-12.9, -5.2)
Sometimes justified (2)	1078	-0.8 (-4.0, 2.5)	920	-0.1 (-3.9, 3.8)	1845	6.1 (3.5, 8.6)	1326	4.7 (1.5, 8.0)
Usually or always justified (3)	1078	1.5 (-0.3, 3.2)	920	2.4 (0.0, 4.8)	1845	1.3 (-0.3, 2.8)	1326	2.0 (-0.1, 4.2)
Non-response	1078	0.0 (-0.6, 0.5)	920	0.7 (0.0, 1.5)	1845	0.0 (-0.3, 0.3)	1326	0.2 (-0.9, 1.3)
Mean score†	1072	*0.053 (0.003, 0.102)*	910	*0.039 (-0.035, 0.113)*	1835	*0.136 (0.092, 0.179)*	1316	*0.142 (0.076, 0.208)*
To stop people who do not share my beliefs from voting‡								
Never justified (1)	1078	-0.5 (-2.0, 1.0)	920	-2.3 (-5.5, 1.0)	1845	0.2 (-1.4, 1.8)	1326	-1.9 (-4.2, 0.4)
Sometimes justified (2)	1078	-0.1 (-1.9, 1.6)	920	0.3 (-2.5, 3.0)	1845	-0.6 (-2.0, 0.7)	1326	0.9 (-0.9, 2.8)
Usually or always justified (3)	1078	0.4 (-0.8, 1.6)	920	1.0 (-0.7, 2.6)	1845	0.2 (-1.0, 1.5)	1326	1.1 (-0.3, 2.6)
Non-response	1078	0.0 (-0.4, 0.4)	920	0.9 (0.1, 1.8)	1845	0.0 (-0.2, 0.3)	1326	-0.6 (-1.4, 0.2)
Mean score†	1072	*0.015 (-0.012, 0.041)*	911	*0.025 (-0.028, 0.078)*	1838	*0.004 (-0.024, 0.033)*	1321	*0.030 (-0.011, 0.071)*
To prevent discrimination based on race or ethnicity‡								
Never justified (1)	1078	-1.4 (-5.4, 2.7)	920	-6.5 (-11.1, -2.0)	1845	-10.0 (-13.4, -6.5)	1326	-13.4 (-17.1, -9.8)
Sometimes justified (2)	1078	0.5 (-3.7, 4.8)	920	2.7 (-1.7, 7.2)	1845	7.2 (3.9, 10.6)	1326	7.7 (3.9, 11.4)
Usually or always justified (3)	1078	0.5 (-1.4, 2.4)	920	1.3 (-1.2, 3.8)	1845	-0.4 (-2.2, 1.5)	1326	2.5 (0.6, 4.4)
Non-response	1078	0.0 (-0.4, 0.4)	920	1.1 (0.1, 2.2)	1845	0.0 (-0.3, 0.3)	1326	-0.3 (-1.0, 0.5)
Mean score†	1074	*0.028 (-0.030, 0.086)*	908	*0.101 (0.026, 0.175)*	1834	*0.157 (0.100, 0.213)*	1319	*0.224 (0.160, 0.287)*
To preserve an American way of life based on Western European traditions‡								
Never justified (1)	1078	0.1 (-3.8, 4.1)	920	-3.4 (-7.4, 0.7)	1845	-2.2 (-4.7, 0.3)	1326	-5.4 (-8.4, -2.4)
Sometimes justified (2)	1078	0.0 (-3.9, 4.0)	920	2.4 (-1.6, 6.4)	1845	0.7 (-1.6, 3.0)	1326	2.8 (-0.1, 5.7)
Usually or always justified (3)	1078	0.3 (-1.4, 1.9)	920	0.3 (-1.8, 2.4)	1845	1.1 (0.1, 2.2)	1326	1.6 (0.3, 3.0)
Non-response	1078	-0.1 (-0.6, 0.4)	920	0.8 (-0.4, 2.1)	1845	0.1 (-0.3, 0.5)	1326	-0.2 (-1.0, 0.7)
Mean score†	1071	*-0.005 (-0.060, 0.051)*	901	*0.027 (-0.033, 0.086)*	1832	*0.038 (0.002, 0.074)*	1316	*0.081 (0.037, 0.125)*

Table S9, continued.

What do you think about the use of force or violence in the following situations?	Mean Differences* by Party Category 2024-2025							
	No MAGA Affiliation							
	Non-MAGA, leans or not strong Republican (n = 1083)		Non-MAGA, Independent (n = 932)		Non-MAGA, leans or not strong Democrat (n = 1849)		Non-MAGA, strong Democrat (n = 1332)	
	Unweighted n	Weighted mean (95% CI) *Mean score (95% CI)*	Unweighted n	Weighted mean (95% CI) *Mean score (95% CI)*	Unweighted n	Weighted mean (95% CI) *Mean score (95% CI)*	Unweighted n	Weighted mean (95% CI) *Mean score (95% CI)*
To preserve an American way of life I believe in‡								
Never justified (1)	1078	-5.0 (-9.1, -0.9)	920	-4.3 (-8.8, 0.1)	1845	-4.6 (-7.6, -1.6)	1326	-8.2 (-11.4, -5.0)
Sometimes justified (2)	1078	2.6 (-1.4, 6.6)	920	3.0 (-1.3, 7.3)	1845	2.1 (-0.9, 5.1)	1326	5.7 (2.5, 9.0)
Usually or always justified (3)	1078	1.6 (-0.6, 3.7)	920	-0.4 (-2.2, 1.4)	1845	1.9 (0.2, 3.7)	1326	0.8 (-0.6, 2.2)
Non-response	1078	0.2 (-0.3, 0.8)	920	0.9 (-0.2, 2.0)	1845	-0.1 (-0.5, 0.4)	1326	0.0 (-0.6, 0.6)
Mean score†	1070	*0.077 (0.007, 0.146)*	904	*0.045 (-0.024, 0.113)*	1831	*0.078 (0.032, 0.124)*	1322	*0.117 (0.062, 0.172)*
To oppose Americans who do not share my beliefs‡								
Never justified (1)	1078	-0.9 (-2.9, 1.2)	920	-1.0 (-4.3, 2.2)	1845	-0.6 (-2.8, 1.5)	1326	-2.6 (-5.0, -0.2)
Sometimes justified (2)	1078	0.4 (-1.5, 2.3)	920	-0.2 (-3.3, 2.9)	1845	-0.1 (-2.0, 1.8)	1326	2.4 (0.0, 4.9)
Usually or always justified (3)	1078	0.2 (-1.0, 1.4)	920	-0.5 (-2.4, 1.3)	1845	0.3 (-0.9, 1.4)	1326	-0.6 (-1.6, 0.3)
Non-response	1078	0.0 (-0.5, 0.6)	920	0.7 (-0.2, 1.5)	1845	0.0 (-0.2, 0.3)	1326	-0.2 (-0.7, 0.4)
Mean score†	1071	*0.015 (-0.015, 0.046)*	908	*0.020 (-0.030, 0.071)*	1838	*0.017 (-0.014, 0.048)*	1323	*0.034 (-0.001, 0.068)*
To oppose the government when it does not share my beliefs‡								
Never justified (1)	1078	0.3 (-2.6, 3.2)	920	-2.2 (-6.6, 2.1)	1845	-4.8 (-7.5, -2.1)	1326	-8.3 (-11.7, -4.9)
Sometimes justified (2)	1078	0.3 (-2.6, 3.2)	920	-1.3 (-5.2, 2.6)	1845	2.9 (0.2, 5.6)	1326	5.9 (2.4, 9.4)
Usually or always justified (3)	1078	-0.2 (-1.3, 0.9)	920	1.5 (-0.4, 3.4)	1845	1.1 (-0.3, 2.6)	1326	1.9 (0.4, 3.4)
Non-response	1078	0.1 (-0.3, 0.6)	920	1.2 (0.2, 2.2)	1845	0.2 (-0.1, 0.6)	1326	-0.2 (-1.0, 0.6)
Mean score†	1072	*-0.013 (-0.050, 0.023)*	907	*0.041 (-0.022, 0.103)*	1834	*0.068 (0.032, 0.103)*	1320	*0.105 (0.060, 0.151)*
To oppose the government when it tries to take private land for public purposes‡								
Never justified (1)	1078	0.4 (-4.0, 4.7)	920	-2.7 (-7.4, 2.0)	1845	-10.2 (-13.6, -6.8)	1326	-10.4 (-14.2, -6.6)
Sometimes justified (2)	1078	-0.5 (-4.9, 3.9)	920	-0.4 (-4.9, 4.2)	1845	7.2 (3.8, 10.5)	1326	7.0 (3.3, 10.8)
Usually or always justified (3)	1078	-0.2 (-2.7, 2.3)	920	0.1 (-2.5, 2.6)	1845	1.9 (0.3, 3.6)	1326	2.5 (0.4, 4.5)
Non-response	1078	0.2 (-0.3, 0.7)	920	0.6 (-0.6, 1.7)	1845	0.1 (-0.2, 0.4)	1326	-0.2 (-0.8, 0.5)
Mean score†	1072	*0.000 (-0.065, 0.065)*	907	*0.068 (-0.008, 0.145)*	1837	*0.139 (0.089, 0.188)*	1316	*0.145 (0.089, 0.200)*

* Among respondents to both the 2024 and 2025 surveys (n=7767).

† To assess population-level change from 2024 to 2025, we computed within-individual change scores for each item and then calculated year-to-year population-level change scores based on the means of aggregated within-individual change scores. Mean change scores have a range from -2 to 2 (with 0 indicating no change).

‡ Respondents who did not answer the question "In general...to advance an important political objective that you support" in 2024 (n = 58) or 2025 (n = 57) were not asked these questions.

Table S10. Party/MAGA affiliation and 2025 prevalence of justification for violence to advance additional specific political objectives

What do you think about the use of force or violence in the following situations?	Population Estimates by Party Category for 2025*							
	MAGA Affiliation						No MAGA Affiliation	
	MAGA Republican (n = 1183)		MAGA Supporter, Republican (n = 565)		MAGA Supporter, Non-Republican (n = 197)		Non-MAGA, strong Republican (n = 417)	
	Unweighted n	Weighted % (95% CI) *Mean score (95% CI)*	Unweighted n	Weighted % (95% CI) *Mean score (95% CI)*	Unweighted n	Weighted % (95% CI) *Mean score (95% CI)*	Unweighted n	Weighted % (95% CI) *Mean score (95% CI)*
To stop voter fraud								
Never justified (1)	659	57.8 (54.0, 61.5)	365	65.7 (59.9, 71.0)	122	58.2 (48.1, 67.7)	262	63.1 (56.6, 69.2)
Sometimes justified (2)	291	23.2 (20.2, 26.5)	151	24.2 (19.6, 29.5)	39	24.0 (16.3, 33.9)	95	22.6 (17.5, 28.6)
Usually or always justified (3)	227	18.7 (16.0, 21.8)	46	9.0 (6.1, 13.2)	32	16.1 (9.8, 25.4)	57	13.6 (9.9, 18.5)
Non-response	3	0.3 (0.1, 1.1)	2	1.1 (0.2, 5.2)	2	1.6 (0.3, 9.7)	1	0.7 (0.1, 5.0)
Mean score†	1177	*1.61 (1.55, 1.67)*	562	*1.43 (1.35, 1.51)*	193	*1.57 (1.42, 1.73)*	414	*1.50 (1.41, 1.59)*
aPD; q-value‡		*10.31 (6.59, 14.02); <0.0001*		*3.06 (-1.47, 7.59); 1.00*		*6.13 (-1.56, 13.83); 1.00*		*6.83 (1.85, 11.80); 0.37*
To stop voter intimidation								
Never justified (1)	673	58.7 (54.9, 62.4)	337	60.5 (54.6, 66.1)	122	60.9 (50.7, 70.1)	242	60.5 (54.0, 66.7)
Sometimes justified (2)	318	24.8 (21.7, 28.1)	182	32.7 (27.4, 38.5)	49	25.8 (17.8, 35.8)	128	28.9 (23.4, 35.2)
Usually or always justified (3)	189	16.5 (13.8, 19.7)	43	5.7 (3.8, 8.5)	22	11.7 (6.5, 20.1)	43	9.6 (6.7, 13.7)
Non-response	0	0.0 (0.0, 0.0)	2	1.1 (0.2, 5.2)	2	1.6 (0.3, 9.7)	2	0.9 (0.2, 4.5)
Mean score†	1180	*1.58 (1.52, 1.64)*	562	*1.45 (1.38, 1.51)*	193	*1.50 (1.36, 1.64)*	413	*1.49 (1.40, 1.57)*
aPD; q-value‡		*5.67 (1.94, 9.39); 0.19*		*-3.46 (-6.86, -0.06); 1.00*		*-1.30 (-8.91, 6.31); 1.00*		*1.06 (-3.28, 5.40); 1.00*
To stop police violence								
Never justified (1)	663	52.9 (49.0, 56.7)	323	55.3 (49.5, 61.0)	91	38.9 (30.2, 48.3)	233	55.4 (48.8, 61.8)
Sometimes justified (2)	368	32.9 (29.3, 36.8)	202	36.2 (30.8, 41.9)	68	33.4 (25.1, 42.9)	134	28.8 (23.4, 35.0)
Usually or always justified (3)	146	14.0 (11.5, 16.9)	37	7.4 (4.9, 11.2)	34	26.1 (17.6, 36.8)	45	14.5 (10.2, 20.3)
Non-response	3	0.2 (0.1, 0.7)	2	1.1 (0.2, 5.2)	2	1.6 (0.3, 9.7)	3	1.2 (0.3, 4.3)
Mean score†	1177	*1.61 (1.55, 1.67)*	562	*1.52 (1.44, 1.59)*	193	*1.87 (1.70, 2.04)*	412	*1.59 (1.49, 1.69)*
aPD; q-value‡		*-1.39 (-5.74, 2.96); 1.00*		*-6.98 (-11.76, -2.21); 0.26*		*5.26 (-4.24, 14.76); 1.00*		*-0.54 (-6.65, 5.56); 1.00*
To reinforce the police								
Never justified (1)	306	29.5 (26.0, 33.3)	157	31.1 (25.9, 36.7)	71	38.3 (29.5, 47.9)	138	37.5 (31.2, 44.3)
Sometimes justified (2)	537	43.8 (40.1, 47.7)	293	49.7 (44.0, 55.5)	84	38.6 (29.7, 48.4)	185	42.3 (36.0, 48.8)
Usually or always justified (3)	335	26.5 (23.4, 29.9)	113	19.0 (15.0, 23.9)	37	21.3 (13.9, 31.3)	88	18.4 (14.3, 23.3)
Non-response	2	0.1 (0.0, 0.6)	1	0.2 (0.0, 1.5)	3	1.8 (0.3, 9.2)	4	1.8 (0.6, 5.3)
Mean score†	1178	*1.97 (1.91, 2.03)*	563	*1.88 (1.80, 1.96)*	192	*1.83 (1.67, 1.98)*	411	*1.81 (1.71, 1.90)*
aPD; q-value‡		*18.87 (14.83, 22.92); <0.0001*		*13.49 (8.15, 18.83); 0.0002*		*14.21 (5.23, 23.18); 0.14*		*13.21 (8.03, 18.38); 0.0001*
To stop illegal immigration								
Never justified (1)	317	28.7 (25.3, 32.4)	189	35.4 (30.0, 41.2)	80	46.3 (36.8, 56.1)	164	40.1 (33.9, 46.7)
Sometimes justified (2)	456	37.8 (34.1, 41.5)	226	38.7 (33.3, 44.3)	63	26.8 (19.4, 35.8)	133	31.2 (25.6, 37.6)
Usually or always justified (3)	402	33.1 (29.6, 36.8)	147	24.9 (20.1, 30.4)	51	25.4 (17.6, 35.1)	117	27.9 (22.4, 34.1)
Non-response	5	0.4 (0.2, 1.2)	2	1.1 (0.2, 5.2)	1	1.5 (0.2, 10.1)	1	0.7 (0.1, 5.0)
Mean score†	1175	*2.04 (1.98, 2.11)*	562	*1.89 (1.80, 1.99)*	194	*1.79 (1.62, 1.95)*	414	*1.88 (1.77, 1.98)*
aPD; q-value‡		*25.27 (21.00, 29.54); <0.0001*		*19.57 (13.90, 25.23); <0.0001*		*16.47 (7.46, 25.48); 0.04*		*22.12 (15.72, 28.52); <0.0001*
To keep borders open								
Never justified (1)	930	77.7 (74.3, 80.8)	460	80.4 (75.3, 84.6)	134	61.9 (51.6, 71.3)	320	74.9 (68.4, 80.5)
Sometimes justified (2)	133	11.7 (9.3, 14.5)	62	9.7 (7.1, 13.1)	32	19.9 (12.5, 30.2)	54	15.7 (11.1, 21.6)
Usually or always justified (3)	112	10.2 (8.1, 12.8)	38	9.2 (6.0, 14.0)	28	16.6 (10.3, 25.7)	40	8.6 (5.5, 13.3)
Non-response	5	0.4 (0.1, 1.1)	4	0.6 (0.2, 1.9)	1	1.5 (0.2, 10.1)	1	0.7 (0.1, 5.0)
Mean score†	1175	*1.32 (1.27, 1.37)*	560	*1.28 (1.20, 1.36)*	194	*1.54 (1.39, 1.69)*	414	*1.33 (1.25, 1.42)*
aPD; q-value‡		*4.17 (0.77, 7.56); 0.67*		*4.43 (-0.38, 9.24); 1.00*		*4.98 (-2.86, 12.83); 1.00*		*2.71 (-1.94, 7.37); 1.00*

Table S10, continued.

What do you think about the use of force or violence in the following situations?	Population Estimates by Party Category for 2025*							
	MAGA Affiliation						No MAGA Affiliation	
	MAGA Republican (n = 1183)		MAGA Supporter, Republican (n = 565)		MAGA Supporter, Non-Republican (n = 197)		Non-MAGA, strong Republican (n = 417)	
	Unweighted n	Weighted % (95% CI) *Mean score (95% CI)*	Unweighted n	Weighted % (95% CI) *Mean score (95% CI)*	Unweighted n	Weighted % (95% CI) *Mean score (95% CI)*	Unweighted n	Weighted % (95% CI) *Mean score (95% CI)*
To stop a protest or demonstration								
Never justified (1)	594	50.2 (46.3, 54.0)	296	56.5 (50.8, 62.0)	107	51.9 (42.1, 61.5)	228	56.0 (49.5, 62.3)
Sometimes justified (2)	477	39.0 (35.3, 42.8)	235	36.6 (31.4, 42.1)	70	34.5 (25.9, 44.3)	151	32.2 (26.7, 38.3)
Usually or always justified (3)	107	10.7 (8.5, 13.3)	31	5.9 (3.8, 9.0)	15	11.8 (5.9, 22.2)	34	10.4 (6.9, 15.6)
Non-response	2	0.1 (0.0, 0.6)	2	1.1 (0.2, 5.2)	3	1.8 (0.3, 9.2)	2	1.4 (0.3, 5.3)
Mean score†	1178	*1.60 (1.55, 1.66)*	562	*1.49 (1.42, 1.56)*	192	*1.59 (1.44, 1.75)*	413	*1.54 (1.44, 1.63)*
aPD; q-value‡		*7.90 (4.80, 11.00); 0.0001*		*4.28 (0.76, 7.79); 0.69*		*5.32 (-2.20, 12.83); 1.00*		*8.23 (3.44, 13.02); 0.07*
To support a protest or demonstration								
Never justified (1)	972	80.6 (77.1, 83.7)	489	82.5 (76.8, 87.1)	143	65.3 (54.9, 74.4)	345	81.1 (75.2, 85.9)
Sometimes justified (2)	164	14.2 (11.5, 17.3)	64	13.3 (9.4, 18.5)	35	25.4 (17.1, 35.9)	58	14.1 (10.0, 19.5)
Usually or always justified (3)	44	5.2 (3.6, 7.4)	9	3.1 (1.4, 6.7)	15	7.7 (3.9, 14.5)	10	3.4 (1.6, 7.0)
Non-response	0	0.0 (0.0, 0.0)	2	1.1 (0.2, 5.2)	2	1.6 (0.3, 9.7)	2	1.4 (0.3, 5.3)
Mean score†	1180	*1.25 (1.20, 1.29)*	562	*1.20 (1.13, 1.26)*	193	*1.41 (1.29, 1.54)*	413	*1.21 (1.15, 1.28)*
aPD; q-value‡		*1.20 (-1.59, 3.99); 1.00*		*-0.31 (-3.73, 3.10); 1.00*		*0.05 (-5.73, 5.84); 1.00*		*-0.33 (-3.59, 2.93); 1.00*
To protect the environment or stop climate change§								
Never justified (1)	984	80.6 (77.0, 83.7)	512	89.1 (84.5, 92.5)	148	69.1 (58.9, 77.8)	342	79.8 (73.8, 84.8)
Sometimes justified (2)	131	12.1 (9.7, 15.1)	42	8.6 (5.6, 12.9)	25	12.6 (7.7, 20.0)	51	12.1 (8.4, 17.2)
Usually or always justified (3)	60	6.9 (4.8, 9.7)	9	2.1 (0.9, 4.8)	20	16.7 (9.8, 26.9)	20	6.7 (3.9, 11.2)
Non-response	5	0.4 (0.2, 1.2)	1	0.2 (0.0, 1.5)	2	1.6 (0.3, 9.7)	2	1.4 (0.3, 5.3)
Mean score†	1175	*1.26 (1.21, 1.31)*	563	*1.13 (1.08, 1.18)*	193	*1.47 (1.30, 1.64)*	413	*1.26 (1.18, 1.34)*
aPD; q-value‡		*-3.11 (-6.69, 0.46); 1.00*		*-6.08 (-9.62, -2.53); 0.07*		*1.70 (-6.93, 10.33); 1.00*		*-3.62 (-8.11, 0.88); 1.00*
To protect the rights of animals								
Never justified (1)	757	62.3 (58.4, 66.0)	423	70.9 (64.9, 76.3)	123	62.0 (52.0, 71.1)	278	62.9 (56.2, 69.2)
Sometimes justified (2)	268	22.8 (19.7, 26.2)	96	19.6 (14.9, 25.3)	42	18.5 (12.2, 27.1)	80	19.1 (14.2, 25.1)
Usually or always justified (3)	150	14.5 (11.9, 17.6)	43	9.2 (6.2, 13.5)	29	18.0 (11.2, 27.6)	55	16.6 (12.1, 22.4)
Non-response	5	0.4 (0.2, 1.2)	2	0.2 (0.0, 1.4)	1	1.5 (0.2, 10.1)	2	1.4 (0.3, 5.3)
Mean score†	1175	*1.52 (1.46, 1.58)*	562	*1.38 (1.30, 1.46)*	194	*1.55 (1.39, 1.72)*	413	*1.53 (1.43, 1.64)*
aPD; q-value‡		*2.20 (-1.85, 6.24); 1.00*		*-0.26 (-5.05, 4.52); 1.00*		*2.11 (-6.07, 10.29); 1.00*		*6.17 (0.15, 12.19); 1.00*
To support women's reproductive rights								
Never justified (1)	942	77.8 (74.2, 81.0)	490	85.1 (79.8, 89.3)	142	67.1 (56.6, 76.0)	334	78.3 (72.2, 83.4)
Sometimes justified (2)	145	12.8 (10.4, 15.8)	48	9.8 (6.4, 14.7)	24	11.7 (6.5, 20.3)	51	12.7 (8.9, 17.9)
Usually or always justified (3)	83	8.4 (6.3, 11.3)	23	3.9 (2.2, 6.9)	27	19.6 (12.3, 29.8)	29	8.2 (5.1, 13.0)
Non-response	10	0.9 (0.4, 1.8)	3	1.1 (0.2, 5.1)	2	1.6 (0.3, 9.7)	1	0.7 (0.1, 5.0)
Mean score†	1170	*1.30 (1.25, 1.35)*	561	*1.18 (1.12, 1.24)*	193	*1.52 (1.34, 1.69)*	414	*1.29 (1.21, 1.38)*
aPD; q-value‡		*-3.52 (-7.25, 0.20); 1.00*		*-6.21 (-9.98, -2.45); 0.10*		*3.35 (-5.62, 12.32); 1.00*		*-3.43 (-8.28, 1.41); 1.00*
To support the right to life								
Never justified (1)	757	62.3 (58.4, 66.0)	400	68.7 (62.8, 74.1)	128	65.6 (55.7, 74.3)	281	63.7 (57.1, 69.9)
Sometimes justified (2)	239	21.1 (18.1, 24.5)	111	19.2 (15.2, 24.1)	28	12.7 (7.6, 20.4)	85	21.9 (16.8, 28.0)
Usually or always justified (3)	176	15.9 (13.2, 19.1)	52	11.9 (7.9, 17.5)	37	20.1 (13.2, 29.4)	48	13.7 (9.6, 19.0)
Non-response	8	0.7 (0.3, 1.5)	1	0.2 (0.0, 1.5)	2	1.6 (0.3, 9.7)	1	0.7 (0.1, 5.0)
Mean score†	1172	*1.53 (1.47, 1.59)*	563	*1.43 (1.34, 1.53)*	193	*1.54 (1.37, 1.70)*	414	*1.50 (1.40, 1.59)*
aPD; q-value‡		*7.21 (3.39, 11.03); 0.03*		*5.64 (0.25, 11.04); 1.00*		*8.40 (0.22, 16.57); 1.00*		*5.85 (0.45, 11.24); 1.00*

Table S10, continued.

What do you think about the use of force or violence in the following situations?	Population Estimates by Party Category for 2025*							
	No MAGA Affiliation							
	Non-MAGA, leans or not strong Republican (n = 1083)		Non-MAGA, Independent (n = 932)		Non-MAGA, leans or not strong Democrat (n = 1849)		Non-MAGA, strong Democrat (n = 1332)	
	Unweighted n	Weighted % (95% CI) *Mean score (95% CI)*	Unweighted n	Weighted % (95% CI) *Mean score (95% CI)*	Unweighted n	Weighted % (95% CI) *Mean score (95% CI)*	Unweighted n	Weighted % (95% CI) *Mean score (95% CI)*
To stop voter fraud								
Never justified (1)	792	74.3 (70.6, 77.6)	699	76.1 (72.3, 79.5)	1478	79.8 (77.2, 82.2)	1064	79.5 (76.4, 82.2)
Sometimes justified (2)	202	17.5 (14.6, 20.8)	142	14.4 (11.7, 17.6)	259	13.6 (11.6, 15.9)	151	11.2 (9.2, 13.7)
Usually or always justified (3)	82	7.5 (5.7, 9.9)	72	7.6 (5.7, 10.2)	103	6.4 (5.0, 8.1)	110	9.1 (7.2, 11.4)
Non-response	5	0.7 (0.2, 2.0)	12	1.9 (1.0, 3.5)	6	0.2 (0.1, 0.6)	3	0.2 (0.1, 0.7)
Mean score†	1076	*1.33 (1.28, 1.38)*	913	*1.30 (1.25, 1.35)*	1840	*1.3 (1.2, 1.3)*	1325	*1.29 (1.25, 1.34)*
aPD; q-value‡		*0.93 (-2.20, 4.07); 1.00*		*-1.43 (-4.71, 1.85); 1.00*		*-1.90 (-4.51, 0.71); 1.00*		*Reference*
To stop voter intimidation								
Never justified (1)	731	69.2 (65.4, 72.8)	621	69.0 (64.9, 72.8)	1172	66.5 (63.7, 69.3)	832	61.1 (57.6, 64.6)
Sometimes justified (2)	264	23.4 (20.1, 26.9)	231	23.2 (19.8, 27.0)	521	25.3 (22.8, 27.9)	367	28.1 (24.9, 31.5)
Usually or always justified (3)	79	6.7 (5.0, 8.9)	63	6.4 (4.6, 8.8)	148	7.9 (6.5, 9.6)	127	10.7 (8.6, 13.2)
Non-response	7	0.8 (0.3, 2.0)	10	1.4 (0.7, 2.9)	5	0.2 (0.1, 0.6)	2	0.1 (0.0, 0.6)
Mean score†	1074	*1.37 (1.32, 1.42)*	915	*1.36 (1.31, 1.42)*	1841	*1.41 (1.38, 1.45)*	1326	*1.49 (1.45, 1.54)*
aPD; q-value‡		*-2.07 (-5.17, 1.04); 1.00*		*-4.18 (-7.45, -0.90); 0.55*		*-2.18 (-4.97, 0.60); 1.00*		*Reference*
To stop police violence								
Never justified (1)	617	57.0 (53.0, 61.0)	471	52.1 (47.8, 56.4)	953	50.6 (47.6, 53.7)	684	47.3 (43.8, 50.9)
Sometimes justified (2)	369	33.2 (29.5, 37.1)	341	35.1 (31.2, 39.3)	695	36.8 (33.9, 39.8)	478	34.7 (31.4, 38.1)
Usually or always justified (3)	88	8.9 (6.8, 11.7)	101	11.2 (8.8, 14.2)	190	12.2 (10.3, 14.4)	165	17.9 (14.9, 21.2)
Non-response	7	0.9 (0.3, 2.1)	12	1.5 (0.8, 3.0)	8	0.4 (0.2, 0.8)	1	0.1 (0.0, 0.7)
Mean score†	1074	*1.52 (1.46, 1.57)*	913	*1.58 (1.53, 1.64)*	1838	*1.61 (1.57, 1.66)*	1327	*1.71 (1.65, 1.76)*
aPD; q-value‡		*-5.96 (-9.94, -1.97); 0.22*		*-7.90 (-12.23, -3.58); 0.04*		*-5.98 (-9.70, -2.25); 0.13*		*Reference*
To reinforce the police								
Never justified (1)	439	44.9 (40.9, 48.9)	490	59.4 (55.2, 63.5)	1119	64.4 (61.5, 67.2)	896	69.2 (65.8, 72.4)
Sometimes justified (2)	493	42.1 (38.2, 46.1)	338	30.1 (26.5, 34.0)	605	28.9 (26.3, 31.7)	333	22.7 (19.9, 25.8)
Usually or always justified (3)	142	12.1 (9.6, 15.1)	83	8.6 (6.5, 11.4)	109	6.1 (4.7, 7.8)	97	7.9 (6.0, 10.3)
Non-response	7	0.9 (0.4, 2.2)	14	1.8 (1.0, 3.4)	13	0.6 (0.3, 1.1)	2	0.2 (0.0, 0.7)
Mean score†	1074	*1.67 (1.61, 1.73)*	911	*1.48 (1.43, 1.54)*	1833	*1.41 (1.38, 1.45)*	1326	*1.39 (1.34, 1.43)*
aPD; q-value‡		*6.99 (3.43, 10.55); 0.02*		*1.39 (-1.91, 4.70); 1.00*		*-0.36 (-2.93, 2.22); 1.00*		*Reference*
To stop illegal immigration								
Never justified (1)	540	53.3 (49.3, 57.3)	578	66.6 (62.6, 70.4)	1403	78.0 (75.4, 80.3)	1066	78.4 (75.1, 81.3)
Sometimes justified (2)	372	31.1 (27.6, 34.9)	243	23.2 (19.9, 26.8)	342	16.1 (14.1, 18.4)	184	14.1 (11.8, 16.8)
Usually or always justified (3)	165	15.0 (12.2, 18.2)	92	8.7 (6.6, 11.3)	95	5.6 (4.3, 7.3)	78	7.5 (5.6, 10.0)
Non-response	4	0.6 (0.2, 1.9)	12	1.5 (0.8, 2.9)	6	0.3 (0.1, 0.8)	0	0.0 (0.0, 0.0)
Mean score†	1077	*1.61 (1.55, 1.68)*	913	*1.41 (1.36, 1.47)*	1840	*1.27 (1.24, 1.31)*	1328	*1.29 (1.24, 1.34)*
aPD; q-value‡		*10.05 (6.36, 13.74); <0.0001*		*1.66 (-1.71, 5.04); 1.00*		*-0.91 (-3.36, 1.53); 1.00*		*Reference*
To keep borders open								
Never justified (1)	863	79.6 (76.2, 82.6)	693	72.9 (68.9, 76.5)	1441	76.6 (73.8, 79.1)	1042	73.8 (70.3, 77.1)
Sometimes justified (2)	156	14.5 (11.9, 17.5)	163	19.0 (15.8, 22.6)	316	16.8 (14.6, 19.2)	212	17.9 (15.1, 21.0)
Usually or always justified (3)	57	5.3 (3.8, 7.3)	55	6.4 (4.6, 8.8)	79	6.0 (4.6, 7.9)	72	8.2 (6.1, 10.9)
Non-response	5	0.6 (0.2, 1.9)	14	1.8 (1.0, 3.3)	10	0.6 (0.3, 1.1)	2	0.1 (0.0, 0.3)
Mean score†	1076	*1.25 (1.21, 1.29)*	911	*1.32 (1.27, 1.37)*	1836	*1.29 (1.25, 1.33)*	1326	*1.34 (1.29, 1.39)*
aPD; q-value‡		*-0.41 (-3.38, 2.57); 1.00*		*-2.23 (-5.55, 1.09); 1.00*		*-1.74 (-4.54, 1.07); 1.00*		*Reference*

Table S10, continued.

What do you think about the use of force or violence in the following situations?	Population Estimates by Party Category for 2025*							
	No MAGA Affiliation							
	Non-MAGA, leans or not strong Republican (n = 1083)		Non-MAGA, Independent (n = 932)		Non-MAGA, leans or not strong Democrat (n = 1849)		Non-MAGA, strong Democrat (n = 1332)	
	Unweighted n	Weighted % (95% CI) *Mean score (95% CI)*	Unweighted n	Weighted % (95% CI) *Mean score (95% CI)*	Unweighted n	Weighted % (95% CI) *Mean score (95% CI)*	Unweighted n	Weighted % (95% CI) *Mean score (95% CI)*
To stop a protest or demonstration								
Never justified (1)	680	65.7 (61.8, 69.3)	628	68.8 (64.7, 72.6)	1448	78.1 (75.4, 80.5)	1114	80.5 (77.3, 83.4)
Sometimes justified (2)	352	28.9 (25.5, 32.5)	247	24.8 (21.3, 28.6)	350	18.2 (16.0, 20.7)	172	14.2 (11.9, 17.0)
Usually or always justified (3)	44	4.8 (3.3, 7.1)	37	4.7 (3.1, 7.0)	40	3.4 (2.3, 4.9)	40	5.1 (3.5, 7.5)
Non-response	5	0.7 (0.2, 1.9)	13	1.7 (0.9, 3.2)	8	0.3 (0.2, 0.7)	2	0.1 (0.0, 0.6)
Mean score†	1076	*1.39 (1.34, 1.44)*	912	*1.35 (1.30, 1.40)*	1838	*1.25 (1.22, 1.28)*	1326	*1.25 (1.20, 1.29)*
aPD; q-value‡		*2.24 (-0.37, 4.86); 1.00*		*0.05 (-2.64, 2.74); 1.00*		*-0.76 (-2.87, 1.36); 1.00*		*Reference*
To support a protest or demonstration								
Never justified (1)	925	84.9 (81.8, 87.5)	737	76.6 (72.7, 80.2)	1504	78.6 (75.9, 81.1)	1056	74.3 (70.8, 77.5)
Sometimes justified (2)	129	11.4 (9.2, 14.0)	148	16.7 (13.8, 20.2)	279	16.7 (14.5, 19.2)	209	19.9 (17.0, 23.2)
Usually or always justified (3)	21	3.3 (1.9, 5.5)	30	5.1 (3.3, 7.8)	54	4.4 (3.2, 5.9)	63	5.8 (4.2, 7.9)
Non-response	6	0.5 (0.2, 1.2)	10	1.5 (0.8, 3.0)	9	0.4 (0.2, 0.8)	0	0.0 (0.0, 0.0)
Mean score†	1075	*1.18 (1.14, 1.22)*	915	*1.27 (1.22, 1.33)*	1837	*1.25 (1.22, 1.29)*	1328	*1.31 (1.27, 1.36)*
aPD; q-value‡		*-0.39 (-3.06, 2.28); 1.00*		*-1.17 (-4.18, 1.85); 1.00*		*-1.18 (-3.51, 1.14); 1.00*		*Reference*
To protect the environment or stop climate change§								
Never justified (1)	889	81.2 (77.8, 84.2)	692	72.4 (68.4, 76.2)	1348	70.6 (67.8, 73.4)	929	64.8 (61.2, 68.2)
Sometimes justified (2)	137	12.3 (9.9, 15.3)	146	17.6 (14.5, 21.2)	356	19.2 (17.0, 21.7)	262	23.1 (20.1, 26.5)
Usually or always justified (3)	50	6.0 (4.2, 8.5)	75	8.5 (6.4, 11.2)	136	9.8 (8.0, 12.0)	136	12.1 (9.8, 14.8)
Non-response	5	0.4 (0.2, 1.2)	12	1.5 (0.8, 2.9)	6	0.3 (0.1, 0.8)	1	0.0 (0.0, 0.1)
Mean score†	1076	*1.24 (1.20, 1.29)*	913	*1.35 (1.30, 1.41)*	1840	*1.39 (1.35, 1.43)*	1327	*1.47 (1.42, 1.52)*
aPD; q-value‡		*-3.24 (-6.71, 0.23); 1.00*		*-4.35 (-8.04, -0.65); 0.82*		*-1.80 (-5.08, 1.48); 1.00*		*Reference*
To protect the rights of animals								
Never justified (1)	774	70.3 (66.4, 73.9)	615	65.0 (60.8, 69.0)	1294	68.8 (65.9, 71.6)	902	62.2 (58.5, 65.7)
Sometimes justified (2)	210	19.4 (16.4, 22.9)	205	21.9 (18.6, 25.6)	404	21.8 (19.4, 24.3)	293	24.3 (21.3, 27.6)
Usually or always justified (3)	92	9.6 (7.5, 12.3)	93	11.2 (8.8, 14.2)	143	9.1 (7.4, 11.2)	131	13.3 (10.7, 16.3)
Non-response	5	0.6 (0.2, 1.9)	12	1.9 (1.0, 3.4)	5	0.3 (0.1, 0.7)	2	0.2 (0.0, 1.5)
Mean score†	1076	*1.39 (1.34, 1.44)*	913	*1.45 (1.39, 1.51)*	1841	*1.40 (1.36, 1.44)*	1326	*1.51 (1.45, 1.57)*
aPD; q-value‡		*-0.33 (-4.03, 3.37); 1.00*		*-2.85 (-6.85, 1.15); 1.00*		*-2.81 (-6.11, 0.50); 1.00*		*Reference*
To support women's reproductive rights								
Never justified (1)	865	78.4 (74.8, 81.6)	657	69.7 (65.6, 73.5)	1332	69.6 (66.6, 72.4)	902	62.4 (58.7, 65.9)
Sometimes justified (2)	146	12.9 (10.5, 15.9)	161	17.3 (14.3, 20.8)	361	21.2 (18.8, 23.9)	272	23.6 (20.6, 27.0)
Usually or always justified (3)	64	7.9 (5.9, 10.7)	95	11.3 (8.8, 14.3)	146	8.9 (7.3, 10.8)	152	13.7 (11.3, 16.7)
Non-response	6	0.7 (0.2, 1.9)	12	1.7 (0.9, 3.3)	7	0.3 (0.1, 0.8)	2	0.2 (0.0, 1.5)
Mean score†	1075	*1.29 (1.24, 1.34)*	913	*1.41 (1.35, 1.46)*	1839	*1.39 (1.35, 1.43)*	1326	*1.51 (1.46, 1.57)*
aPD; q-value‡		*-3.36 (-7.07, 0.34); 1.00*		*-3.31 (-7.35, 0.73); 1.00*		*-4.51 (-7.83, -1.18); 0.40*		*Reference*
To support the right to life								
Never justified (1)	834	75.0 (71.2, 78.5)	666	70.1 (66.0, 73.8)	1504	79.6 (77.0, 82.0)	1087	78.2 (74.9, 81.2)
Sometimes justified (2)	157	15.1 (12.3, 18.4)	150	17.3 (14.3, 20.8)	227	13.2 (11.2, 15.5)	136	12.0 (9.7, 14.7)
Usually or always justified (3)	84	9.1 (7.0, 11.8)	94	11.1 (8.7, 14.2)	107	6.8 (5.4, 8.5)	102	9.5 (7.4, 12.1)
Non-response	6	0.7 (0.3, 2.0)	15	1.5 (0.8, 2.9)	8	0.4 (0.2, 0.8)	3	0.3 (0.1, 1.6)
Mean score†	1075	*1.34 (1.28, 1.39)*	910	*1.40 (1.34, 1.46)*	1838	1.27 (1.23, 1.30)	1325	*1.31 (1.26, 1.36)*
aPD; q-value‡		*2.32 (-1.12, 5.76); 1.00*		*0.90 (-2.86, 4.66); 1.00*		*-2.15 (-4.96, 0.66); 1.00*		*Reference*

* Among respondents to both the 2024 and 2025 surveys (n=7767). Prevalences for 2024 among respondents to that survey were reported previously: Wintemute GJ, Velasquez B, Robinson SL, Tomsich EA, Wright MA, Shev AB. The MAGA movement and political violence in 2024: findings from a nationally representative survey. Inj Epidemiol. 2025;12(1):78. Respondents who did not answer the question "In general...to advance an important political objective that you support" in 2024 (n =58) or 2025 (n = 57) were not asked these questions.

† Mean scores in 2024 and 2025 were scored as indicated in the response lines for individual questions, with non-responses excluded.

‡ Adjusted prevalence differences (aPDs) are absolute percentage point (pp) differences for “usually or always justified” responses and are adjusted for age, race and ethnicity, gender, income, education, Census division, marital status, homeownership, rurality, firearm ownership, alcohol consumption, military service, and history of non-traffic arrest. Q-values, also known as FDR-adjusted (or FDR-corrected) p-values, represent the probability that the given difference would be a false discovery; they represent the expected proportion of “false positives” that would be seen among the collection of all differences whose q-values were at or below the given q-value. Item non-responses are not reported in the tables but are included in the prevalence calculations.

Table S11. Party/MAGA affiliation and 2024-2025 change in justification for violence to advance additional specific political objectives

What do you think about the use of force or violence in the following situations?	Mean Differences* by Party Category 2024-2025							
	MAGA Affiliation						No MAGA Affiliation	
	MAGA Republican (n = 1183)		MAGA Supporter, Republican (n = 565)		MAGA Supporter, Non-Republican (n = 197)		Non-MAGA, strong Republican (n = 417)	
	Unweighted n	Weighted mean (95% CI) *Mean score (95% CI)*	Unweighted n	Weighted mean (95% CI) *Mean score (95% CI)*	Unweighted n	Weighted mean (95% CI) *Mean score (95% CI)*	Unweighted n	Weighted mean (95% CI) *Mean score (95% CI)*
To stop voter fraud								
Never justified (1)	1179	-3.9 (-8.2, 0.5)	563	-1.2 (-6.8, 4.4)	195	-2.8 (-14.7, 9.1)	415	-7.0 (-13.9, -0.1)
Sometimes justified (2)	1179	0.2 (-4.0, 4.3)	563	-2.7 (-8.5, 3.2)	195	6.4 (-5.0, 17.8)	415	4.1 (-2.4, 10.6)
Usually or always justified (3)	1179	3.5 (0.4, 6.7)	563	3.8 (0.0, 7.6)	195	-5.1 (-16.8, 6.6)	415	2.5 (-2.4, 7.4)
Non-response	1179	0.2 (-0.3, 0.6)	563	0.1 (-0.4, 0.6)	195	1.5 (-1.5, 4.5)	415	0.3 (-1.3, 2.0)
Mean score†	1175	*0.071 (0.008, 0.135)*	560	*0.050 (-0.027, 0.126)*	192	*-0.038 (-0.244, 0.168)*	413	*0.089 (-0.011, 0.189)*
To stop voter intimidation								
Never justified (1)	1179	-3.7 (-7.9, 0.5)	563	-3.9 (-10.8, 2.9)	195	-1.5 (-14.6, 11.5)	415	-4.1 (-11.3, 3.2)
Sometimes justified (2)	1179	-0.7 (-5.0, 3.7)	563	4.0 (-3.6, 11.6)	195	0.9 (-11.0, 12.7)	415	4.3 (-3.0, 11.6)
Usually or always justified (3)	1179	4.6 (1.4, 7.8)	563	0.3 (-3.5, 4.2)	195	-0.8 (-7.4, 5.8)	415	-0.7 (-5.6, 4.2)
Non-response	1179	-0.2 (-0.5, 0.1)	563	-0.4 (-1.2, 0.5)	195	1.5 (-1.5, 4.5)	415	0.5 (-1.2, 2.2)
Mean score†	1177	*0.084 (0.023, 0.145)*	558	*0.042 (-0.040, 0.124)*	192	*-0.009 (-0.177, 0.158)*	411	*0.027 (-0.073, 0.127)*
To stop police violence								
Never justified (1)	1179	-0.6 (-5.2, 4.0)	563	-0.7 (-6.1, 4.8)	195	-9.0 (-20.7, 2.7)	415	-0.4 (-7.4, 6.6)
Sometimes justified (2)	1179	0.5 (-4.1, 5.1)	563	-0.3 (-6.0, 5.4)	195	-3.8 (-16.8, 9.1)	415	-5.8 (-13.1, 1.4)
Usually or always justified (3)	1179	-0.2 (-4.0, 3.6)	563	0.8 (-3.0, 4.7)	195	11.5 (0.6, 22.5)	415	5.5 (-0.3, 11.3)
Non-response	1179	0.2 (-0.1, 0.5)	563	0.1 (-0.4, 0.6)	195	1.3 (-1.7, 4.3)	415	0.8 (-1.0, 2.5)
Mean score†	1175	*0.002 (-0.069, 0.073)*	560	*0.014 (-0.061, 0.089)*	191	*0.209 (0.018, 0.401)*	410	*0.051 (-0.055, 0.157)*
To reinforce the police								
Never justified (1)	1179	-0.7 (-5.1, 3.6)	563	-1.5 (-7.1, 4.2)	195	-14.1 (-26.1, -2.1)	415	-5.1 (-12.7, 2.5)
Sometimes justified (2)	1179	-0.4 (-5.4, 4.6)	563	0.1 (-6.5, 6.8)	195	5.8 (-6.0, 17.7)	415	5.3 (-2.5, 13.0)
Usually or always justified (3)	1179	1.3 (-2.9, 5.6)	563	1.4 (-3.8, 6.6)	195	6.6 (-3.4, 16.6)	415	-1.6 (-7.2, 4.1)
Non-response	1179	-0.1 (-0.5, 0.2)	563	-0.1 (-0.7, 0.5)	195	1.6 (-1.4, 4.6)	415	1.4 (-0.7, 3.6)
Mean score†	1175	*0.020 (-0.051, 0.090)*	560	*0.028 (-0.059, 0.114)*	191	*0.198 (0.011, 0.385)*	410	*0.018 (-0.091, 0.126)*
To stop illegal immigration								
Never justified (1)	1179	-3.8 (-8.2, 0.6)	563	1.0 (-4.2, 6.2)	195	-1.6 (-11.9, 8.8)	415	-3.5 (-10.3, 3.4)
Sometimes justified (2)	1179	1.0 (-3.5, 5.6)	563	2.5 (-4.0, 8.9)	195	4.9 (-5.9, 15.7)	415	0.3 (-6.7, 7.3)
Usually or always justified (3)	1179	2.4 (-1.7, 6.6)	563	-3.3 (-8.4, 1.7)	195	-4.7 (-15.4, 5.9)	415	2.9 (-3.7, 9.5)
Non-response	1179	0.3 (-0.2, 0.8)	563	-0.1 (-0.8, 0.6)	195	1.4 (-1.6, 4.4)	415	0.3 (-1.3, 2.0)
Mean score†	1173	*0.062 (-0.011, 0.135)*	560	*-0.046 (-0.126, 0.035)*	193	*-0.034 (-0.219, 0.152)*	413	*0.061 (-0.053, 0.174)*
To keep borders open								
Never justified (1)	1179	-2.0 (-6.4, 2.3)	563	-2.3 (-7.4, 2.7)	195	-11.2 (-20.7, -1.8)	415	-5.6 (-12.5, 1.4)
Sometimes justified (2)	1179	0.1 (-3.6, 3.8)	563	0.4 (-3.9, 4.8)	195	7.2 (-1.7, 16.1)	415	2.3 (-3.9, 8.5)
Usually or always justified (3)	1179	1.9 (-0.9, 4.7)	563	2.1 (-2.2, 6.4)	195	2.7 (-5.5, 10.8)	415	3.0 (-1.8, 7.7)
Non-response	1179	0.0 (-0.5, 0.6)	563	-0.2 (-2.0, 1.6)	195	1.4 (-1.6, 4.4)	415	0.3 (-1.3, 2.0)
Mean score†	1171	*0.041 (-0.022, 0.105)*	558	*0.050 (-0.030, 0.130)*	193	*0.126 (-0.025, 0.277)*	413	*0.083 (-0.017, 0.183)*

Table S11, continued.

What do you think about the use of force or violence in the following situations?	Mean Differences* by Party Category 2024-2025							
	MAGA Affiliation						No MAGA Affiliation	
	MAGA Republican (n = 1183)		MAGA Supporter, Republican (n = 565)		MAGA Supporter, Non-Republican (n = 197)		Non-MAGA, strong Republican (n = 417)	
	Unweighted n	Weighted mean (95% CI) *Mean score (95% CI)*	Unweighted n	Weighted mean (95% CI) *Mean score (95% CI)*	Unweighted n	Weighted mean (95% CI) *Mean score (95% CI)*	Unweighted n	Weighted mean (95% CI) *Mean score (95% CI)*
To stop a protest or demonstration								
Never justified (1)	1179	-0.6 (-5.4, 4.2)	563	4.9 (-0.2, 10.1)	195	-7.2 (-17.9, 3.4)	415	4.0 (-3.6, 11.6)
Sometimes justified (2)	1179	1.2 (-3.9, 6.3)	563	-3.4 (-9.2, 2.3)	195	7.3 (-3.4, 18.0)	415	-5.8 (-13.7, 2.2)
Usually or always justified (3)	1179	-0.5 (-3.4, 2.4)	563	-1.6 (-5.2, 1.9)	195	-1.6 (-8.3, 5.2)	415	0.8 (-4.4, 6.0)
Non-response	1179	-0.1 (-0.5, 0.3)	563	0.1 (-0.4, 0.6)	195	1.4 (-1.6, 4.5)	415	1.0 (-1.1, 3.0)
Mean score†	1175	*0.000 (-0.062, 0.062)*	560	*-0.068 (-0.135, -0.001)*	190	*0.059 (-0.090, 0.209)*	412	*-0.047 (-0.149, 0.054)*
To support a protest or demonstration								
Never justified (1)	1179	1.0 (-2.7, 4.7)	563	-3.7 (-8.9, 1.4)	195	-4.8 (-15.6, 6.0)	415	-1.3 (-7.8, 5.2)
Sometimes justified (2)	1179	-0.7 (-4.4, 3.0)	563	1.9 (-3.5, 7.3)	195	6.1 (-4.4, 16.6)	415	0.4 (-5.1, 5.8)
Usually or always justified (3)	1179	0.0 (-2.4, 2.4)	563	1.7 (-0.5, 3.9)	195	-2.8 (-9.1, 3.5)	415	-0.1 (-3.3, 3.2)
Non-response	1179	-0.2 (-0.5, 0.1)	563	0.1 (-0.4, 0.6)	195	1.5 (-1.5, 4.5)	415	1.0 (-1.1, 3.0)
Mean score†	1177	*-0.010 (-0.060, 0.039)*	560	*0.054 (-0.004, 0.113)*	192	*0.020 (-0.129, 0.169)*	412	*-0.002 (-0.086, 0.083)*
To protect the environment or stop climate change								
Never justified (1)	1179	-4.4 (-8.1, -0.6)	563	2.9 (-2.0, 7.8)	195	-0.1 (-11.8, 11.5)	415	-2.3 (-7.6, 2.9)
Sometimes justified (2)	1179	2.2 (-1.0, 5.4)	563	-2.3 (-6.9, 2.3)	195	-5.9 (-16.4, 4.6)	415	1.1 (-2.9, 5.1)
Usually or always justified (3)	1179	2.0 (-0.9, 4.8)	563	0.1 (-1.8, 1.9)	195	4.5 (-4.8, 13.9)	415	0.2 (-3.1, 3.5)
Non-response	1179	0.2 (-0.4, 0.7)	563	-0.6 (-2.4, 1.1)	195	1.5 (-1.5, 4.5)	415	1.0 (-1.1, 3.0)
Mean score†	1172	*0.062 (0.004, 0.120)*	561	*-0.022 (-0.076, 0.031)*	192	*0.031 (-0.150, 0.212)*	412	*0.023 (-0.054, 0.100)*
To protect the rights of animals								
Never justified (1)	1179	-3.4 (-7.4, 0.6)	563	1.0 (-5.0, 7.1)	195	0.7 (-11.4, 12.8)	415	-3.0 (-9.7, 3.7)
Sometimes justified (2)	1179	2.9 (-0.9, 6.7)	563	-2.8 (-7.9, 2.3)	195	-1.4 (-9.3, 6.5)	415	-1.0 (-7.3, 5.2)
Usually or always justified (3)	1179	0.4 (-2.6, 3.4)	563	2.4 (-1.5, 6.2)	195	-0.7 (-10.1, 8.8)	415	3.0 (-3.0, 9.0)
Non-response	1179	0.2 (-0.4, 0.7)	563	-0.6 (-2.4, 1.1)	195	1.4 (-1.6, 4.4)	415	1.0 (-1.1, 3.0)
Mean score†	1172	*0.037 (-0.023, 0.097)*	560	*0.020 (-0.066, 0.105)*	193	*-0.029 (-0.231, 0.172)*	412	*0.065 (-0.044, 0.173)*
To support women's reproductive rights								
Never justified (1)	1179	-4.6 (-8.3, -0.9)	563	-2.4 (-6.2, 1.4)	195	1.8 (-9.3, 12.8)	415	-0.5 (-6.8, 5.9)
Sometimes justified (2)	1179	2.0 (-1.1, 5.0)	563	2.1 (-1.3, 5.5)	195	-4.4 (-12.0, 3.2)	415	-1.2 (-7.4, 5.0)
Usually or always justified (3)	1179	2.1 (-0.6, 4.8)	563	0.1 (-3.0, 3.2)	195	1.2 (-7.2, 9.6)	415	1.3 (-2.4, 5.0)
Non-response	1179	0.5 (-0.1, 1.2)	563	0.1 (-0.3, 0.6)	195	1.5 (-1.5, 4.5)	415	0.3 (-1.3, 2.0)
Mean score†	1167	*0.062 (0.005, 0.118)*	559	*0.024 (-0.037, 0.084)*	192	*-0.022 (-0.201, 0.157)*	413	*0.015 (-0.066, 0.095)*
To support the right to life								
Never justified (1)	1179	-1.0 (-5.7, 3.7)	563	-5.4 (-11.1, 0.3)	195	4.7 (-8.5, 17.8)	415	-7.0 (-13.5, -0.6)
Sometimes justified (2)	1179	0.2 (-4.0, 4.3)	563	3.3 (-2.0, 8.6)	195	-8.8 (-19.0, 1.4)	415	2.2 (-4.3, 8.6)
Usually or always justified (3)	1179	0.5 (-3.0, 4.0)	563	2.8 (-1.7, 7.2)	195	2.6 (-7.6, 12.8)	415	4.6 (-0.6, 9.8)
Non-response	1179	0.3 (-0.2, 0.8)	563	-0.6 (-2.4, 1.1)	195	1.5 (-1.5, 4.5)	415	0.3 (-1.4, 1.9)
Mean score†	1169	*0.010 (-0.062, 0.083)*	561	*0.080 (-0.009, 0.170)*	192	*-0.037 (-0.248, 0.174)*	412	*0.114 (0.017, 0.210)*

Table S11, continued.

What do you think about the use of force or violence in the following situations?	Mean Differences* by Party Category 2024-2025							
	No MAGA Affiliation							
	Non-MAGA, leans or not strong Republican (n = 1083)		Non-MAGA, Independent (n = 932)		Non-MAGA, leans or not strong Democrat (n = 1849)		Non-MAGA, strong Democrat (n = 1332)	
	Unweighted n	Weighted mean (95% CI) *Mean score (95% CI)*	Unweighted n	Weighted mean (95% CI) *Mean score (95% CI)*	Unweighted n	Weighted mean (95% CI) *Mean score (95% CI)*	Unweighted n	Weighted mean (95% CI) *Mean score (95% CI)*
To stop voter fraud								
Never justified (1)	1078	-5.0 (-8.7, -1.4)	920	-3.1 (-7.4, 1.3)	1845	-5.8 (-8.5, -3.0)	1326	-5.8 (-8.8, -2.7)
Sometimes justified (2)	1078	2.5 (-1.0, 6.0)	920	-0.3 (-4.2, 3.6)	1845	4.5 (2.1, 6.8)	1326	4.0 (1.5, 6.5)
Usually or always justified (3)	1078	2.5 (0.3, 4.7)	920	2.3 (-0.3, 4.9)	1845	1.4 (-0.3, 3.1)	1326	1.9 (-0.4, 4.2)
Non-response	1078	0.0 (-0.5, 0.6)	920	1.1 (0.2, 1.9)	1845	-0.1 (-0.5, 0.3)	1326	-0.1 (-0.6, 0.5)
Mean score†	1071	*0.076 (0.027, 0.125)*	911	*0.046 (-0.014, 0.107)*	1833	*0.072 (0.033, 0.111)*	1322	*0.072 (0.024, 0.120)*
To stop voter intimidation								
Never justified (1)	1078	-5.7 (-9.5, -1.9)	920	-3.9 (-8.4, 0.6)	1845	-3.6 (-6.7, -0.5)	1326	-8.3 (-12.1, -4.5)
Sometimes justified (2)	1078	3.7 (0.2, 7.2)	920	2.8 (-1.6, 7.2)	1845	1.8 (-1.3, 4.8)	1326	5.5 (1.3, 9.6)
Usually or always justified (3)	1078	2.0 (-0.5, 4.5)	920	0.3 (-2.2, 2.8)	1845	2.0 (0.0, 3.9)	1326	2.9 (0.1, 5.6)
Non-response	1078	0.0 (-0.7, 0.7)	920	0.8 (-0.3, 1.8)	1845	-0.1 (-0.5, 0.3)	1326	0.0 (-0.2, 0.3)
Mean score†	1068	*0.074 (0.020, 0.127)*	911	*0.035 (-0.023, 0.092)*	1834	*0.056 (0.014, 0.097)*	1322	*0.113 (0.061, 0.164)*
To stop police violence								
Never justified (1)	1078	-3.7 (-8.4, 1.0)	920	-4.2 (-9.4, 1.1)	1845	-5.2 (-8.4, -1.9)	1326	-10.2 (-13.9, -6.6)
Sometimes justified (2)	1078	1.2 (-3.5, 6.0)	920	3.8 (-1.4, 8.9)	1845	2.6 (-0.7, 6.0)	1326	1.7 (-2.2, 5.5)
Usually or always justified (3)	1078	2.3 (-0.2, 4.8)	920	-0.3 (-3.4, 2.8)	1845	2.5 (0.1, 4.8)	1326	8.7 (5.6, 11.9)
Non-response	1078	0.1 (-0.6, 0.8)	920	0.7 (-0.2, 1.6)	1845	0.0 (-0.3, 0.4)	1326	-0.2 (-0.7, 0.4)
Mean score†	1068	*0.061 (0.003, 0.118)*	909	*0.032 (-0.038, 0.102)*	1830	*0.075 (0.029, 0.121)*	1324	*0.187 (0.131, 0.243)*
To reinforce the police								
Never justified (1)	1078	-1.6 (-6.2, 3.1)	920	-1.9 (-6.8, 2.9)	1845	-4.5 (-7.8, -1.1)	1326	-3.7 (-7.0, -0.3)
Sometimes justified (2)	1078	0.2 (-4.8, 5.1)	920	0.4 (-4.2, 5.1)	1845	4.4 (1.2, 7.6)	1326	2.3 (-0.9, 5.5)
Usually or always justified (3)	1078	1.2 (-2.1, 4.5)	920	0.4 (-2.6, 3.4)	1845	0.0 (-1.9, 2.0)	1326	1.2 (-1.0, 3.5)
Non-response	1078	0.1 (-0.6, 0.9)	920	1.1 (0.1, 2.1)	1845	0.1 (-0.4, 0.6)	1326	0.2 (-0.1, 0.4)
Mean score†	1068	*0.025 (-0.039, 0.088)*	907	*0.015 (-0.051, 0.080)*	1824	*0.044 (-0.001, 0.089)*	1324	*0.048 (0.001, 0.094)*
To stop illegal immigration								
Never justified (1)	1078	-4.8 (-9.0, -0.5)	920	-2.9 (-7.3, 1.5)	1845	-0.8 (-3.8, 2.1)	1326	-3.7 (-6.7, -0.6)
Sometimes justified (2)	1078	2.7 (-1.6, 7.1)	920	2.0 (-2.3, 6.3)	1845	0.3 (-2.5, 3.2)	1326	2.8 (-0.1, 5.7)
Usually or always justified (3)	1078	2.0 (-0.6, 4.6)	920	-0.2 (-2.8, 2.4)	1845	0.5 (-1.1, 2.1)	1326	1.2 (-1.0, 3.4)
Non-response	1078	0.0 (-0.5, 0.5)	920	1.1 (0.0, 2.2)	1845	0.0 (-0.2, 0.3)	1326	-0.3 (-0.9, 0.3)
Mean score†	1073	*0.067 (0.012, 0.123)*	906	*0.017 (-0.039, 0.074)*	1835	*0.014 (-0.025, 0.053)*	1324	*0.052 (0.008, 0.095)*
To keep borders open								
Never justified (1)	1078	-3.1 (-6.5, 0.3)	920	-5.3 (-9.2, -1.3)	1845	-4.0 (-7.0, -1.0)	1326	-7.1 (-10.5, -3.8)
Sometimes justified (2)	1078	3.1 (-0.1, 6.3)	920	3.6 (-0.3, 7.5)	1845	1.9 (-1.0, 4.8)	1326	4.4 (1.3, 7.5)
Usually or always justified (3)	1078	0.1 (-1.9, 2.2)	920	0.7 (-2.1, 3.5)	1845	1.8 (-0.1, 3.8)	1326	3.0 (0.6, 5.3)
Non-response	1078	-0.1 (-0.8, 0.5)	920	1.0 (0.0, 1.9)	1845	0.3 (-0.1, 0.7)	1326	-0.3 (-0.8, 0.3)
Mean score†	1071	*0.032 (-0.013, 0.076)*	905	*0.050 (-0.005, 0.106)*	1830	*0.057 (0.016, 0.098)*	1320	*0.099 (0.051, 0.147)*

Table S11, continued.

What do you think about the use of force or violence in the following situations?	Mean Differences* by Party Category 2024-2025							
	No MAGA Affiliation							
	Non-MAGA, leans or not strong Republican (n = 1083)		Non-MAGA, Independent (n = 932)		Non-MAGA, leans or not strong Democrat (n = 1849)		Non-MAGA, strong Democrat (n = 1332)	
	Unweighted n	Weighted mean (95% CI) *Mean score (95% CI)*	Unweighted n	Weighted mean (95% CI) *Mean score (95% CI)*	Unweighted n	Weighted mean (95% CI) *Mean score (95% CI)*	Unweighted n	Weighted mean (95% CI) *Mean score (95% CI)*
To stop a protest or demonstration								
Never justified (1)	1078	1.6 (-2.5, 5.8)	920	-2.5 (-7.5, 2.4)	1845	2.7 (-0.4, 5.8)	1326	0.4 (-2.6, 3.4)
Sometimes justified (2)	1078	-1.5 (-5.6, 2.6)	920	2.2 (-2.5, 7.0)	1845	-3.4 (-6.4, -0.4)	1326	-1.0 (-4.0, 1.9)
Usually or always justified (3)	1078	-0.1 (-2.5, 2.3)	920	-0.8 (-3.4, 1.7)	1845	0.7 (-0.8, 2.3)	1326	0.8 (-1.3, 2.8)
Non-response	1078	0.0 (-0.6, 0.6)	920	1.2 (0.3, 2.1)	1845	0.0 (-0.4, 0.4)	1326	-0.2 (-0.7, 0.4)
Mean score†	1070	*-0.018 (-0.071, 0.036)*	909	*0.006 (-0.056, 0.068)*	1831	*-0.019 (-0.057, 0.020)*	1323	*0.000 (-0.041, 0.041)*
To support a protest or demonstration								
Never justified (1)	1078	-1.5 (-4.7, 1.7)	920	-3.6 (-7.8, 0.5)	1845	-2.8 (-5.8, 0.2)	1326	-6.4 (-9.7, -3.1)
Sometimes justified (2)	1078	1.0 (-1.8, 3.9)	920	1.3 (-2.7, 5.2)	1845	1.1 (-1.7, 3.9)	1326	4.6 (1.3, 7.9)
Usually or always justified (3)	1078	0.4 (-1.5, 2.4)	920	1.7 (-0.9, 4.3)	1845	1.5 (-0.1, 3.1)	1326	2.1 (0.3, 3.8)
Non-response	1078	0.0 (-0.6, 0.7)	920	0.7 (-0.5, 1.8)	1845	0.2 (-0.2, 0.6)	1326	-0.3 (-0.7, 0.2)
Mean score†	1068	*0.019 (-0.025, 0.063)*	908	*0.048 (-0.008, 0.103)*	1832	*0.042 (0.003, 0.082)*	1325	*0.083 (0.042, 0.123)*
To protect the environment or stop climate change								
Never justified (1)	1078	-3.0 (-6.2, 0.1)	920	-4.6 (-8.5, -0.6)	1845	-4.5 (-7.5, -1.5)	1326	-11.4 (-14.8, -8.0)
Sometimes justified (2)	1078	0.2 (-2.9, 3.2)	920	2.8 (-0.7, 6.3)	1845	1.3 (-1.6, 4.2)	1326	8.6 (5.3, 11.9)
Usually or always justified (3)	1078	2.7 (0.6, 4.7)	920	0.9 (-2.0, 3.9)	1845	3.1 (1.1, 5.1)	1326	3.6 (1.3, 5.8)
Non-response	1078	0.2 (-0.3, 0.8)	920	0.9 (-0.2, 1.9)	1845	0.1 (-0.3, 0.5)	1326	-0.8 (-1.6, 0.1)
Mean score†	1071	*0.055 (0.012, 0.098)*	908	*0.046 (-0.013, 0.106)*	1834	*0.075 (0.033, 0.117)*	1321	*0.154 (0.109, 0.200)*
To protect the rights of animals								
Never justified (1)	1078	-3.7 (-7.6, 0.2)	920	-3.9 (-8.2, 0.5)	1845	-1.7 (-4.6, 1.2)	1326	-8.4 (-12.0, -4.7)
Sometimes justified (2)	1078	0.3 (-4.1, 4.6)	920	3.3 (-1.0, 7.5)	1845	-0.1 (-3.0, 2.8)	1326	5.0 (1.4, 8.5)
Usually or always justified (3)	1078	3.4 (0.7, 6.1)	920	-0.4 (-3.4, 2.7)	1845	1.9 (0.2, 3.7)	1326	3.7 (1.2, 6.2)
Non-response	1078	0.1 (-0.4, 0.6)	920	1.0 (0.1, 1.9)	1845	-0.1 (-0.6, 0.3)	1326	-0.3 (-0.9, 0.3)
Mean score†	1072	*0.069 (0.018, 0.120)*	908	*0.027 (-0.035, 0.089)*	1833	*0.036 (-0.003, 0.074)*	1322	*0.124 (0.073, 0.175)*
To support women's reproductive rights								
Never justified (1)	1078	-3.9 (-7.4, -0.3)	920	-4.9 (-9.2, -0.6)	1845	-5.7 (-8.8, -2.7)	1326	-11.6 (-15.1, -8.1)
Sometimes justified (2)	1078	1.8 (-1.7, 5.3)	920	0.8 (-3.5, 5.1)	1845	5.0 (1.9, 8.2)	1326	7.5 (4.1, 10.9)
Usually or always justified (3)	1078	2.1 (-0.5, 4.8)	920	3.0 (0.1, 5.9)	1845	0.6 (-1.6, 2.8)	1326	4.4 (1.7, 7.1)
Non-response	1078	-0.1 (-0.7, 0.6)	920	1.1 (0.2, 2.1)	1845	0.1 (-0.3, 0.5)	1326	-0.3 (-0.9, 0.3)
Mean score†	1070	*0.059 (0.007, 0.111)*	910	*0.072 (0.013, 0.131)*	1833	*0.062 (0.019, 0.105)*	1322	*0.164 (0.111, 0.216)*
To support the right to life								
Never justified (1)	1078	-4.7 (-8.7, -0.7)	920	-2.7 (-7.3, 1.9)	1845	-3.1 (-5.9, -0.4)	1326	-5.4 (-8.4, -2.5)
Sometimes justified (2)	1078	2.6 (-1.4, 6.5)	920	1.5 (-2.9, 5.9)	1845	1.9 (-0.8, 4.6)	1326	3.3 (0.6, 5.9)
Usually or always justified (3)	1078	2.2 (-0.5, 4.8)	920	0.4 (-2.7, 3.6)	1845	1.1 (-0.5, 2.7)	1326	2.4 (0.1, 4.6)
Non-response	1078	0.0 (-0.7, 0.7)	920	0.8 (-0.1, 1.6)	1845	0.1 (-0.3, 0.5)	1326	-0.2 (-1.1, 0.7)
Mean score†	1070	*0.066 (0.010, 0.121)*	906	*0.027 (-0.039, 0.092)*	1832	*0.042 (0.006, 0.078)*	1320	*0.076 (0.032, 0.120)*

* Among respondents to both the 2024 and 2025 surveys (n=7767). Respondents who did not answer the question "In general...to advance an important political objective that you support" in 2024 (n = 58) or 2025 (n = 57) were not asked these questions.

† To assess population-level change from 2024 to 2025, we computed within-individual change scores for each item and then calculated year-to-year population-level change scores based on the means of aggregated within-individual change scores. Mean change scores have a range from -2 to 2 (with 0 indicating no change).

Table S12. Party/MAGA affiliation and 2025 prevalence of personal willingness to commit political violence, by type of violence

In a situation where you think force or violence is justified to advance an important political objective…How willing would you personally be to use force or violence in each of these ways?	Population Estimates by Party Category for 2025*							
	MAGA Affiliation						No MAGA Affiliation	
	MAGA Republican (n = 1183)		MAGA Supporter, Republican (n = 565)		MAGA Supporter, Non-Republican (n = 197)		Non-MAGA, strong Republican (n = 417)	
	Unweighted n	Weighted % (95% CI) *Mean score (95% CI)*	Unweighted n	Weighted % (95% CI) *Mean score (95% CI)*	Unweighted n	Weighted % (95% CI) *Mean score (95% CI)*	Unweighted n	Weighted % (95% CI) *Mean score (95% CI)*
To damage property								
Not asked the question†	128	11.7 (9.5, 14.4)	71	14.4 (10.7, 19.3)	27	19.7 (12.9, 28.7)	66	18.6 (13.7, 24.9)
Not willing (1)	963	80.0 (76.6, 83.0)	441	73.3 (67.2, 78.6)	137	63.1 (53.1, 72.1)	319	72.4 (65.6, 78.2)
Somewhat willing (2)	65	6.2 (4.3, 8.8)	40	8.5 (5.2, 13.7)	14	4.9 (2.7, 8.7)	21	5.3 (3.0, 9.2)
Very or completely willing (3)	18	1.5 (0.9, 2.6)	9	2.6 (1.1, 5.9)	15	12.0 (6.3, 21.8)	8	3.0 (1.2, 7.7)
Non-response	6	0.6 (0.2, 1.4)	3	1.2 (0.3, 5.1)	2	0.4 (0.1, 1.7)	1	0.6 (0.1, 4.4)
Mean score‡	1046	*1.10 (1.07, 1.14)*	490	*1.16 (1.09, 1.23)*	166	*1.36 (1.18, 1.54)*	348	*1.14 (1.06, 1.22)*
aPD; q-value§		*0.59 (-0.96, 2.14); 1.00*		*1.71 (-1.00, 4.43); 1.00*		*8.49 (0.96, 16.02); 0.97*		*2.00 (-1.12, 5.12); 1.00*
To threaten or intimidate a person								
Not asked the question†	128	11.7 (9.5, 14.4)	71	14.4 (10.7, 19.3)	27	19.7 (12.9, 28.7)	66	18.6 (13.7, 24.9)
Not willing (1)	922	76.9 (73.4, 80.0)	437	74.4 (68.7, 79.3)	134	61.3 (51.3, 70.4)	311	71.6 (64.9, 77.4)
Somewhat willing (2)	99	8.3 (6.3, 10.8)	47	8.5 (5.7, 12.5)	16	5.7 (3.2, 10.0)	29	6.8 (4.2, 10.8)
Very or completely willing (3)	24	2.5 (1.4, 4.3)	4	1.1 (0.4, 3.3)	16	13.0 (7.0, 22.8)	8	2.4 (1.0, 5.8)
Non-response	7	0.6 (0.3, 1.5)	5	1.6 (0.5, 5.0)	2	0.4 (0.1, 1.7)	1	0.6 (0.1, 4.4)
Mean score‡	1045	*1.15 (1.11, 1.19)*	488	*1.13 (1.08, 1.18)*	166	*1.40 (1.21, 1.58)*	348	*1.14 (1.08, 1.21)*
aPD; q-value§		*2.01 (0.15, 3.87); 1.00*		*0.31 (-1.60, 2.21); 1.00*		*10.34 (2.57, 18.12); 0.43*		*1.80 (-0.60, 4.21); 1.00*
To injure a person								
Not asked the question†	128	11.7 (9.5, 14.4)	71	14.4 (10.7, 19.3)	27	19.7 (12.9, 28.7)	66	18.6 (13.7, 24.9)
Not willing (1)	949	78.4 (74.9, 81.6)	445	75.3 (69.6, 80.2)	133	59.3 (49.3, 68.6)	318	74.2 (67.7, 79.8)
Somewhat willing (2)	76	7.5 (5.4, 10.4)	40	8.0 (5.2, 12.2)	19	11.3 (6.4, 19.1)	24	4.6 (2.6, 8.1)
Very or completely willing (3)	19	1.4 (0.8, 2.4)	4	1.0 (0.3, 3.0)	14	9.4 (4.7, 18.1)	6	1.9 (0.6, 5.4)
Non-response	8	0.9 (0.4, 2.2)	4	1.3 (0.3, 5.0)	2	0.4 (0.1, 1.7)	1	0.6 (0.1, 4.4)
Mean score‡	1044	*1.12 (1.09, 1.15)*	489	*1.12 (1.07, 1.16)*	166	*1.38 (1.21, 1.54)*	348	*1.10 (1.05, 1.16)*
aPD; q-value§		*0.46 (-1.05, 1.96); 1.00*		*-0.19 (-2.03, 1.66); 1.00*		*6.23 (-0.12, 12.59); 1.00*		*0.82 (-1.56, 3.20); 1.00*
To kill a person								
Not asked the question†	128	11.7 (9.5, 14.4)	71	14.4 (10.7, 19.3)	27	19.7 (12.9, 28.7)	66	18.6 (13.7, 24.9)
Not willing (1)	971	81.3 (78.1, 84.2)	462	78.0 (72.4, 82.7)	138	64.2 (54.3, 73.0)	329	76.0 (69.4, 81.5)
Somewhat willing (2)	58	4.8 (3.3, 7.0)	24	5.8 (3.4, 9.8)	10	6.6 (2.7, 15.2)	14	3.0 (1.3, 6.5)
Very or completely willing (3)	16	1.5 (0.8, 2.8)	4	0.6 (0.2, 2.2)	18	9.2 (5.2, 15.6)	5	1.8 (0.6, 5.3)
Non-response	7	0.7 (0.3, 1.5)	3	1.2 (0.3, 5.1)	2	0.4 (0.1, 1.7)	1	0.6 (0.1, 4.4)
Mean score‡	1045	*1.09 (1.06, 1.12)*	490	*1.08 (1.04, 1.12)*	166	*1.31 (1.18, 1.45)*	348	*1.08 (1.02, 1.14)*
aPD; q-value§		*1.10 (-0.39, 2.59); 1.00*		*0.01 (-1.53, 1.54); 1.00*		*6.80 (1.33, 12.26); 0.63*		*1.28 (-1.00, 3.57); 1.00*

Table S12, continued.

In a situation where you think force or violence is justified to advance an important political objective…How willing would you personally be to use force or violence in each of these ways?	Population Estimates by Party Category for 2025*							
	No MAGA Affiliation							
	Non-MAGA, leans or not strong Republican (n = 1083)		Non-MAGA, Independent (n = 932)		Non-MAGA, leans or not strong Democrat (n = 1849)		Non-MAGA, strong Democrat (n = 1332)	
	Unweighted n	Weighted % (95% CI) *Mean score (95% CI)*	Unweighted n	Weighted % (95% CI) *Mean score (95% CI)*	Unweighted n	Weighted % (95% CI) *Mean score (95% CI)*	Unweighted n	Weighted % (95% CI) *Mean score (95% CI)*
To damage property								
Not asked the question†	213	24.3 (20.9, 28.1)	218	26.8 (23.1, 30.9)	434	24.4 (21.8, 27.1)	362	25.4 (22.6, 28.5)
Not willing (1)	787	68.4 (64.5, 72.2)	600	60.0 (55.7, 64.2)	1208	62.6 (59.5, 65.5)	826	62.3 (58.8, 65.6)
Somewhat willing (2)	64	5.2 (3.8, 7.3)	76	8.6 (6.5, 11.3)	157	9.2 (7.5, 11.2)	120	10.1 (8.0, 12.7)
Very or completely willing (3)	14	1.6 (0.8, 3.2)	25	3.2 (2.0, 5.3)	41	3.4 (2.3, 4.9)	18	1.9 (1.1, 3.2)
Non-response	3	0.4 (0.1, 1.2)	6	1.4 (0.6, 3.2)	6	0.5 (0.2, 1.5)	2	0.3 (0.1, 1.4)
Mean score‡	865	*1.11 (1.08, 1.15)*	701	*1.21 (1.16, 1.26)*	1406	*1.21 (1.17, 1.25)*	964	*1.19 (1.15, 1.23)*
aPD; q-value§		*0.56 (-1.17, 2.30); 1.00*		*0.97 (-0.95, 2.88); 1.00*		*1.18 (-0.53, 2.90); 1.00*		*Reference*
To threaten or intimidate a person								
Not asked the question†	213	24.3 (20.9, 28.1)	218	26.8 (23.1, 30.9)	434	24.4 (21.8, 27.1)	362	25.4 (22.6, 28.5)
Not willing (1)	764	66.3 (62.3, 70.0)	604	60.0 (55.6, 64.2)	1230	64.6 (61.6, 67.5)	874	66.4 (63.0, 69.7)
Somewhat willing (2)	91	7.7 (5.9, 10.1)	68	8.7 (6.4, 11.7)	148	8.4 (6.8, 10.3)	76	6.3 (4.6, 8.6)
Very or completely willing (3)	10	1.3 (0.6, 2.9)	26	3.2 (1.9, 5.2)	28	2.2 (1.4, 3.4)	13	1.6 (0.9, 3.1)
Non-response	3	0.4 (0.1, 1.2)	9	1.4 (0.6, 3.0)	6	0.5 (0.1, 1.5)	3	0.2 (0.0, 0.6)
Mean score‡	865	*1.14 (1.10, 1.18)*	698	*1.21 (1.16, 1.26)*	1406	*1.17 (1.14, 1.20)*	963	*1.13 (1.09, 1.17)*
aPD; q-value§		*0.67 (-0.94, 2.28); 1.00*		*1.49 (-0.48, 3.47); 1.00*		*0.65 (-0.84, 2.14); 1.00*		*Reference*
To injure a person								
Not asked the question†	213	24.3 (20.9, 28.1)	218	26.8 (23.1, 30.9)	434	24.4 (21.8, 27.1)	362	25.4 (22.6, 28.5)
Not willing (1)	793	68.9 (65.0, 72.6)	621	61.6 (57.3, 65.8)	1273	67.0 (64.1, 69.9)	900	67.7 (64.3, 70.9)
Somewhat willing (2)	57	5.0 (3.5, 6.9)	62	8.4 (6.1, 11.4)	113	6.9 (5.4, 8.8)	52	4.8 (3.3, 6.9)
Very or completely willing (3)	13	1.3 (0.5, 3.0)	19	2.1 (1.1, 3.8)	20	1.2 (0.7, 2.1)	13	2.0 (1.1, 3.7)
Non-response	5	0.5 (0.2, 1.3)	5	1.1 (0.4, 2.8)	6	0.5 (0.1, 1.5)	1	0.1 (0.0, 0.7)
Mean score‡	863	*1.10 (1.06, 1.14)*	702	*1.17 (1.13, 1.22)*	1406	*1.12 (1.10, 1.15)*	965	*1.12 (1.08, 1.16)*
aPD; q-value§		*0.21 (-1.55, 1.97); 1.00*		*0.10 (-1.66, 1.87); 1.00*		*-0.41 (-1.82, 1.01); 1.00*		*Reference*
To kill a person								
Not asked the question†	213	24.3 (20.9, 28.1)	218	26.8 (23.1, 30.9)	434	24.4 (21.8, 27.1)	362	25.4 (22.6, 28.5)
Not willing (1)	826	72.2 (68.3, 75.8)	643	64.3 (60.0, 68.4)	1328	70.2 (67.3, 73.0)	928	70.0 (66.7, 73.2)
Somewhat willing (2)	28	1.7 (1.1, 2.7)	36	4.7 (3.0, 7.3)	56	3.4 (2.3, 4.9)	27	3.1 (1.8, 5.1)
Very or completely willing (3)	11	1.4 (0.6, 3.2)	23	3.0 (1.8, 5.1)	22	1.4 (0.9, 2.4)	9	1.4 (0.7, 2.8)
Non-response	3	0.4 (0.1, 1.2)	5	1.1 (0.4, 2.8)	6	0.6 (0.2, 1.6)	2	0.1 (0.0, 0.6)
Mean score‡	865	*1.06 (1.03, 1.09)*	702	*1.15 (1.10, 1.20)*	1406	*1.08 (1.06, 1.11)*	964	*1.08 (1.04, 1.11)*
aPD; q-value§		*0.89 (-0.78, 2.55); 1.00*		*1.80 (-0.04, 3.63); 1.00*		*0.41 (-0.84, 1.67); 1.00*		*Reference*

* Among respondents to both the 2024 and 2025 surveys (n=7767). Prevalences for 2024 among respondents to that survey were reported previously: Wintemute GJ, Velasquez B, Robinson SL, Tomsich EA, Wright MA, Shev AB. The MAGA movement and political violence in 2024: findings from a nationally representative survey. Inj Epidemiol. 2025;12(1):78. Respondents who did not answer the question "In general...to advance an important political objective that you support" in 2024 (n = 58) or 2025 (n = 57) were not asked these questions.

† Respondents answered “never justified” to all prior questions on the use of force or violence to advance specific political objectives were not asked questions on their personal willingness to use political violence.

‡ Mean scores in 2024 and 2025 were scored as indicated in the response lines for individual questions, with non-responses excluded.

§ Adjusted prevalence differences (aPDs) are absolute percentage point (pp) differences for “very or completely willing” responses and are adjusted for age, race and ethnicity, gender, income, education, Census division, marital status, homeownership, rurality, firearm ownership, alcohol consumption, military service, and history of non-traffic arrest. Q-values, also known as FDR-adjusted (or FDR-corrected) p-values, represent the probability that the given difference would be a false discovery; they represent the expected proportion of “false positives” that would be seen among the collection of all differences whose q-values were at or below the given q-value. Item non-responses are not reported in the tables but are included in the prevalence calculations.

Table S13. Party/MAGA affiliation and 2024-2025 change in personal willingness to commit political violence, by type of violence

In a situation where you think force or violence is justified to advance an important political objective...How willing would you personally be to use force or violence in each of these ways?	**Mean Differences* by Party Category 2024-2025**							
	**MAGA Affiliation**						**No MAGA Affiliation**	
	**MAGA Republican (n = 1183)**		**MAGA Supporter, Republican (n = 565)**		**MAGA Supporter, Non-Republican (n = 197)**		**Non-MAGA, strong Republican (n = 417)**	
	**Unweighted n**	**Weighted mean (95% CI)** ***Mean score (95% CI)***	**Unweighted n**	**Weighted mean (95% CI)** ***Mean score (95% CI)***	**Unweighted n**	**Weighted mean (95% CI)** ***Mean score (95% CI)***	**Unweighted n**	**Weighted mean (95% CI)** ***Mean score (95% CI)***
To damage property								
Not asked the question†	1179	0.2 (-3.0, 3.4)	563	1.7 (-3.4, 6.9)	195	-8.3 (-20.0, 3.5)	415	-0.5 (-6.7, 5.7)
Not willing (1)	1179	2.1 (-2.0, 6.3)	563	-3.5 (-11.0, 4.0)	195	12.1 (0.1, 24.0)	415	1.1 (-6.6, 8.8)
Somewhat willing (2)	1179	-0.8 (-3.5, 1.9)	563	0.1 (-5.0, 5.3)	195	-2.6 (-9.1, 3.9)	415	-2.0 (-7.0, 3.1)
Very or completely willing (3)	1179	-1.4 (-3.0, 0.2)	563	1.7 (-0.6, 3.9)	195	0.4 (-5.9, 6.7)	415	0.7 (-2.9, 4.3)
Non-response	1179	-0.1 (-0.8, 0.5)	563	0.0 (-0.8, 0.7)	195	-1.6 (-4.6, 1.4)	415	0.6 (-0.6, 1.9)
Mean score‡	972	*-0.039 (-0.082, 0.003)*	462	*0.043 (-0.042, 0.127)*	136	*-0.033 (-0.182, 0.115)*	302	*0.009 (-0.086, 0.104)*
To threaten or intimidate a person								
Not asked the question†	1179	0.2 (-3.0, 3.4)	563	1.7 (-3.4, 6.9)	195	-8.3 (-20.0, 3.5)	415	-0.5 (-6.7, 5.7)
Not willing (1)	1179	1.3 (-2.9, 5.5)	563	2.5 (-4.7, 9.7)	195	13.2 (1.2, 25.1)	415	1.5 (-6.0, 9.0)
Somewhat willing (2)	1179	-1.4 (-4.3, 1.4)	563	-3.6 (-8.2, 1.0)	195	-5.0 (-12.0, 2.0)	415	-1.1 (-6.3, 4.2)
Very or completely willing (3)	1179	-0.1 (-1.4, 1.2)	563	-1.1 (-3.4, 1.2)	195	1.7 (-4.1, 7.5)	415	-0.6 (-3.2, 2.1)
Non-response	1179	0.0 (-0.6, 0.7)	563	0.5 (-0.4, 1.4)	195	-1.6 (-4.6, 1.4)	415	0.6 (-0.6, 1.9)
Mean score‡	971	*-0.018 (-0.058, 0.023)*	460	*-0.085 (-0.163, -0.007)*	136	*-0.041 (-0.174, 0.091)*	302	*-0.015 (-0.088, 0.057)*
To injure a person								
Not asked the question†	1179	0.2 (-3.0, 3.4)	563	1.7 (-3.4, 6.9)	195	-8.3 (-20.0, 3.5)	415	-0.5 (-6.7, 5.7)
Not willing (1)	1179	0.3 (-3.7, 4.3)	563	0.6 (-6.4, 7.7)	195	6.8 (-5.0, 18.6)	415	3.4 (-4.0, 10.8)
Somewhat willing (2)	1179	0.5 (-1.8, 2.7)	563	-2.0 (-6.5, 2.5)	195	4.1 (-3.3, 11.5)	415	-2.9 (-7.9, 2.1)
Very or completely willing (3)	1179	-1.2 (-2.6, 0.3)	563	-0.5 (-2.3, 1.3)	195	-0.8 (-7.6, 6.0)	415	-0.6 (-3.7, 2.6)
Non-response	1179	0.1 (-0.8, 1.1)	563	0.1 (-0.6, 0.9)	195	-1.8 (-4.8, 1.2)	415	0.6 (-0.7, 1.8)
Mean score‡	970	*-0.021 (-0.059, 0.018)*	460	*-0.048 (-0.119, 0.023)*	135	*0.019 (-0.111, 0.149)*	301	*-0.044 (-0.118, 0.031)*
To kill a person								
Not asked the question†	1179	0.2 (-3.0, 3.4)	563	1.7 (-3.4, 6.9)	195	-8.3 (-20.0, 3.5)	415	-0.5 (-6.7, 5.7)
Not willing (1)	1179	0.8 (-3.0, 4.5)	563	-1.2 (-7.8, 5.3)	195	10.9 (-1.4, 23.1)	415	3.9 (-3.2, 11.0)
Somewhat willing (2)	1179	0.2 (-2.1, 2.5)	563	1.4 (-2.2, 5.0)	195	-3.6 (-9.0, 1.7)	415	-2.3 (-6.5, 1.9)
Very or completely willing (3)	1179	-0.8 (-2.5, 1.0)	563	-1.8 (-4.0, 0.5)	195	3.1 (-0.1, 6.2)	415	-1.7 (-5.1, 1.7)
Non-response	1179	-0.4 (-1.5, 0.6)	563	-0.2 (-0.9, 0.6)	195	-2.0 (-5.1, 1.1)	415	0.6 (-0.7, 1.8)
Mean score‡	971	*-0.017 (-0.049, 0.015)*	460	*-0.026 (-0.095, 0.044)*	135	*0.019 (-0.096, 0.133)*	301	*-0.056 (-0.128, 0.017)*

Table S13, continued.

In a situation where you think force or violence is justified to advance an important political objective...How willing would <u>you personally</u> be to use force or violence in each of these ways?	Mean Differences* by Party Category 2024-2025							
	No MAGA Affiliation							
	Non-MAGA, leans or not strong Republican (n = 1083)		Non-MAGA, Independent (n = 932)		Non-MAGA, leans or not strong Democrat (n = 1849)		Non-MAGA, strong Democrat (n = 1332)	
	**Unweighted n**	**Weighted mean (95% CI)** ***Mean score (95% CI)***	**Unweighted n**	**Weighted mean (95% CI)** ***Mean score (95% CI)***	**Unweighted n**	**Weighted mean (95% CI)** ***Mean score (95% CI)***	**Unweighted n**	**Weighted mean (95% CI)** ***Mean score (95% CI)***
To damage property								
Not asked the question†	1078	0.0 (-4.3, 4.3)	920	-5.6 (-10.8, -0.4)	1845	-6.5 (-9.6, -3.4)	1326	-11.4 (-15.0, -7.8)
Not willing (1)	1078	0.8 (-3.9, 5.6)	920	4.8 (-0.8, 10.4)	1845	5.2 (1.6, 8.8)	1326	10.4 (5.9, 14.8)
Somewhat willing (2)	1078	-1.8 (-4.1, 0.5)	920	0.1 (-2.9, 3.1)	1845	0.0 (-2.3, 2.2)	1326	1.4 (-1.3, 4.2)
Very or completely willing (3)	1078	0.8 (-0.4, 2.0)	920	-0.1 (-2.1, 1.8)	1845	1.0 (-0.4, 2.4)	1326	-0.3 (-1.6, 0.9)
Non-response	1078	0.2 (-0.3, 0.7)	920	0.8 (-0.4, 1.9)	1845	0.4 (-0.1, 0.8)	1326	0.0 (-0.7, 0.7)
Mean score‡	759	*-0.003 (-0.044, 0.038)*	570	*-0.014 (-0.079, 0.052)*	1126	*0.007 (-0.036, 0.050)*	721	*-0.020 (-0.080, 0.040)*
To threaten or intimidate a person								
Not asked the question†	1078	0.0 (-4.3, 4.3)	920	-5.6 (-10.8, -0.4)	1845	-6.5 (-9.6, -3.4)	1326	-11.4 (-15.0, -7.8)
Not willing (1)	1078	-1.5 (-6.2, 3.3)	920	5.0 (-0.6, 10.5)	1845	4.9 (1.5, 8.3)	1326	11.9 (7.8, 16.1)
Somewhat willing (2)	1078	0.8 (-1.8, 3.5)	920	0.0 (-3.4, 3.4)	1845	0.8 (-1.1, 2.7)	1326	0.0 (-2.2, 2.1)
Very or completely willing (3)	1078	0.4 (-0.7, 1.6)	920	0.2 (-1.7, 2.1)	1845	0.6 (-0.6, 1.8)	1326	-0.4 (-1.8, 1.1)
Non-response	1078	0.2 (-0.3, 0.7)	920	0.4 (-0.7, 1.6)	1845	0.3 (-0.2, 0.8)	1326	-0.1 (-0.7, 0.4)
Mean score‡	759	*0.019 (-0.021, 0.060)*	566	*-0.009 (-0.076, 0.057)*	1124	*0.000 (-0.037, 0.036)*	721	*-0.032 (-0.087, 0.023)*
To injure a person								
Not asked the question†	1078	0.0 (-4.3, 4.3)	920	-5.6 (-10.8, -0.4)	1845	-6.5 (-9.6, -3.4)	1326	-11.4 (-15.0, -7.8)
Not willing (1)	1078	-1.3 (-6.1, 3.4)	920	4.3 (-1.2, 9.8)	1845	5.8 (2.4, 9.1)	1326	10.5 (6.5, 14.5)
Somewhat willing (2)	1078	0.3 (-1.9, 2.4)	920	1.8 (-0.8, 4.4)	1845	1.4 (-0.4, 3.1)	1326	0.7 (-1.1, 2.5)
Very or completely willing (3)	1078	0.8 (-0.4, 1.9)	920	-0.9 (-2.7, 0.9)	1845	-0.9 (-2.0, 0.2)	1326	0.4 (-0.9, 1.8)
Non-response	1078	0.3 (-0.2, 0.8)	920	0.3 (-0.7, 1.4)	1845	0.4 (-0.1, 0.9)	1326	-0.3 (-0.8, 0.3)
Mean score‡	756	*0.018 (-0.024, 0.060)*	569	*-0.006 (-0.073, 0.061)*	1126	*-0.032 (-0.068, 0.003)*	722	*-0.006 (-0.053, 0.042)*
To kill a person								
Not asked the question†	1078	0.0 (-4.3, 4.3)	920	-5.6 (-10.8, -0.4)	1845	-6.5 (-9.6, -3.4)	1326	-11.4 (-15.0, -7.8)
Not willing (1)	1078	0.0 (-4.4, 4.5)	920	5.2 (-0.3, 10.6)	1845	6.7 (3.4, 10.1)	1326	11.1 (7.2, 15.0)
Somewhat willing (2)	1078	-0.5 (-1.6, 0.5)	920	0.2 (-2.1, 2.4)	1845	-0.8 (-2.3, 0.6)	1326	0.8 (-0.8, 2.5)
Very or completely willing (3)	1078	0.5 (-0.4, 1.5)	920	-0.3 (-1.9, 1.4)	1845	0.2 (-0.8, 1.2)	1326	-0.1 (-1.5, 1.2)
Non-response	1078	0.0 (-0.6, 0.5)	920	0.5 (-0.5, 1.5)	1845	0.4 (-0.1, 1.0)	1326	-0.4 (-1.0, 0.2)
Mean score‡	756	*0.000 (-0.032, 0.032)*	570	*-0.010 (-0.065, 0.045)*	1125	*-0.019 (-0.051, 0.014)*	719	*-0.019 (-0.068, 0.029)*

* Among respondents to both the 2024 and 2025 surveys (n=7767). Respondents who did not answer the question "In general...to advance an important political objective that you support" in 2024 (n = 58) or 2025 (n = 57) were not asked these questions.

† Respondents answered “never justified” to all prior questions on the use of force or violence to advance specific political objectives were not asked questions on their personal willingness to use political violence.

‡ To assess population-level change from 2024 to 2025, we computed within-individual change scores for each item and then calculated year-to-year population-level change scores based on the means of aggregated within-individual change scores. Mean change scores have a range from -2 to 2 (with 0 indicating no change).

Table S14. Party/MAGA affiliation and 2025 prevalence of personal willingness to engage in political violence, by target population

In a situation where you think force or violence is justified to advance an important political objective...How willing would <u>you personally</u> be to use force or violence in each of these ways?	**Population Estimates by Party Category for 2025***							
	**MAGA Affiliation**						**No MAGA Affiliation**	
	**MAGA Republican (n = 1183)**		**MAGA Supporter, Republican (n = 565)**		**MAGA Supporter, Non-Republican (n = 197)**		**Non-MAGA, strong Republican (n = 417)**	
	**Unweighted n**	**Weighted % (95% CI)** ***Mean score (95% CI)***	**Unweighted n**	**Weighted % (95% CI)** ***Mean score (95% CI)***	**Unweighted n**	**Weighted % (95% CI)** ***Mean score (95% CI)***	**Unweighted n**	**Weighted % (95% CI)** ***Mean score (95% CI)***
An elected federal or state government official								
Not asked the question†	128	11.7 (9.5, 14.4)	71	14.4 (10.7, 19.3)	27	19.7 (12.9, 28.7)	66	18.6 (13.7, 24.9)
Not willing (1)	957	79.6 (76.1, 82.7)	457	76.5 (70.8, 81.4)	138	64.8 (54.7, 73.7)	322	75.0 (68.4, 80.6)
Somewhat willing (2)	69	5.4 (3.9, 7.6)	25	5.5 (3.2, 9.3)	15	8.9 (4.0, 18.4)	16	4.1 (2.0, 8.3)
Very or completely willing (3)	16	2.4 (1.1, 4.9)	4	1.1 (0.4, 3.3)	11	6.0 (2.9, 12.0)	6	1.2 (0.5, 2.8)
Non-response	10	0.9 (0.4, 1.8)	7	2.5 (0.9, 6.6)	4	0.7 (0.2, 2.2)	5	1.1 (0.3, 3.7)
Mean score‡	1042	*1.12 (1.07, 1.16)*	486	*1.09 (1.05, 1.14)*	164	*1.26 (1.13, 1.39)*	344	*1.08 (1.04, 1.12)*
aPD; q-value§		*1.32 (-0.89, 3.53); 1.00*		*-0.14 (-2.05, 1.77); 1.00*		*3.17 (-1.45, 7.78); 1.00*		*0.02 (-1.59, 1.63); 1.00*
An elected local government official								
Not asked the question†	128	11.7 (9.5, 14.4)	71	14.4 (10.7, 19.3)	27	19.7 (12.9, 28.7)	66	18.6 (13.7, 24.9)
Not willing (1)	958	79.4 (75.9, 82.4)	456	77.2 (71.6, 81.9)	145	62.7 (52.4, 72.0)	326	74.9 (68.3, 80.6)
Somewhat willing (2)	61	5.2 (3.8, 7.2)	25	4.8 (2.8, 8.2)	10	9.9 (4.7, 19.7)	14	3.6 (1.6, 7.9)
Very or completely willing (3)	18	2.6 (1.3, 5.1)	6	1.9 (0.6, 5.3)	11	7.4 (3.2, 16.0)	5	1.2 (0.5, 3.0)
Non-response	15	1.1 (0.6, 1.9)	6	1.7 (0.5, 5.0)	2	0.4 (0.1, 1.7)	4	1.7 (0.5, 5.2)
Mean score‡	1037	*1.12 (1.08, 1.16)*	487	*1.10 (1.05, 1.16)*	166	*1.31 (1.15, 1.47)*	345	*1.08 (1.03, 1.12)*
aPD; q-value§		*2.07 (-0.24, 4.39); 1.00*		*1.21 (-1.44, 3.86); 1.00*		*3.24 (-2.39, 8.87); 1.00*		*0.47 (-1.31, 2.26); 1.00*
An election worker, such as a poll worker or vote counter								
Not asked the question†	128	11.7 (9.5, 14.4)	71	14.4 (10.7, 19.3)	27	19.7 (12.9, 28.7)	66	18.6 (13.7, 24.9)
Not willing (1)	982	80.1 (76.6, 83.2)	473	79.2 (73.6, 83.9)	143	64.5 (54.2, 73.5)	330	76.4 (69.9, 81.8)
Somewhat willing (2)	32	4.4 (2.6, 7.2)	12	3.7 (1.8, 7.7)	11	7.1 (3.0, 16.0)	9	2.0 (1.0, 3.9)
Very or completely willing (3)	21	2.4 (1.4, 4.2)	2	0.3 (0.1, 1.4)	12	8.5 (4.0, 17.1)	6	1.8 (0.5, 5.9)
Non-response	17	1.4 (0.8, 2.4)	6	2.3 (0.8, 6.5)	2	0.4 (0.1, 1.7)	4	1.2 (0.4, 3.9)
Mean score‡	1035	*1.11 (1.07, 1.14)*	487	*1.05 (1.02, 1.09)*	166	*1.30 (1.14, 1.46)*	345	*1.07 (1.01, 1.13)*
aPD; q-value§		*2.13 (0.30, 3.96); 0.85*		*-0.07 (-1.46, 1.31); 1.00*		*5.81 (-0.29, 11.9); 1.00*		*1.50 (-0.87, 3.87); 1.00*
A public health official								
Not asked the question†	128	11.7 (9.5, 14.4)	71	14.4 (10.7, 19.3)	27	19.7 (12.9, 28.7)	66	18.6 (13.7, 24.9)
Not willing (1)	972	80.4 (77.0, 83.4)	462	77.1 (71.4, 81.9)	140	60.0 (49.8, 69.4)	326	74.7 (68.1, 80.4)
Somewhat willing (2)	46	3.4 (2.4, 4.7)	26	6.5 (3.9, 10.6)	7	5.2 (1.9, 13.1)	14	3.5 (1.6, 7.4)
Very or completely willing (3)	19	3.3 (1.8, 6.0)	1	0.5 (0.1, 3.6)	18	13.7 (7.5, 23.7)	5	2.1 (0.7, 6.2)
Non-response	15	1.2 (0.7, 2.1)	4	1.5 (0.4, 5.1)	3	1.5 (0.3, 6.5)	4	1.0 (0.3, 3.7)
Mean score‡	1037	*1.11 (1.07, 1.16)*	489	*1.09 (1.05, 1.13)*	165	*1.41 (1.22, 1.61)*	345	*1.10 (1.03, 1.16)*
aPD; q-value§		*1.88 (-0.75, 4.50); 1.00*		*-1.01 (-3.06, 1.04); 1.00*		*9.15 (1.47, 16.83); 0.77*		*0.75 (-1.99, 3.49); 1.00*
A member of the military or National Guard								
Not asked the question†	128	11.7 (9.5, 14.4)	71	14.4 (10.7, 19.3)	27	19.7 (12.9, 28.7)	66	18.6 (13.7, 24.9)
Not willing (1)	977	80.4 (76.9, 83.5)	460	78.6 (73.2, 83.2)	141	63.8 (53.7, 72.8)	325	74.9 (68.3, 80.5)
Somewhat willing (2)	33	3.8 (2.3, 6.1)	20	4.0 (2.2, 7.2)	13	9.8 (4.7, 19.4)	16	4.9 (2.5, 9.1)
Very or completely willing (3)	25	2.9 (1.6, 5.3)	4	1.6 (0.5, 5.2)	12	6.4 (3.2, 12.4)	5	0.7 (0.3, 1.7)
Non-response	17	1.2 (0.7, 2.0)	9	1.4 (0.6, 2.9)	2	0.4 (0.1, 1.7)	3	0.9 (0.2, 3.8)
Mean score‡	1035	*1.11 (1.06, 1.15)*	484	*1.08 (1.03, 1.14)*	166	*1.28 (1.15, 1.41)*	346	*1.08 (1.04, 1.12)*
aPD‡		*2.12 (-0.11, 4.35); 1.00*		*0.84 (-1.65, 3.33); 1.00*		*3.40 (-1.4, 8.19); 1.00*		*-0.15 (-1.54, 1.24); 1.00*

Table S14, continued.

In a situation where you think force or violence is justified to advance an important political objective...How willing would you personally be to use force or violence in each of these ways?	Population Estimates by Party Category for 2025*							
	MAGA Affiliation						No MAGA Affiliation	
	MAGA Republican (n = 1183)		MAGA Supporter, Republican (n = 565)		MAGA Supporter, Non-Republican (n = 197)		Non-MAGA, strong Republican (n = 417)	
	Unweighted n	Weighted % (95% CI) *Mean score (95% CI)*	Unweighted n	Weighted % (95% CI) *Mean score (95% CI)*	Unweighted n	Weighted % (95% CI) *Mean score (95% CI)*	Unweighted n	Weighted % (95% CI) *Mean score (95% CI)*
A police officer								
Not asked the question†	128	11.7 (9.5, 14.4)	71	14.4 (10.7, 19.3)	27	19.7 (12.9, 28.7)	66	18.6 (13.7, 24.9)
Not willing (1)	982	80.6 (77.0, 83.7)	465	77.8 (72.1, 82.7)	143	65.7 (55.7, 74.4)	333	76.6 (70.0, 82.2)
Somewhat willing (2)	40	4.4 (2.9, 6.7)	21	6.2 (3.5, 10.7)	8	3.5 (1.5, 8.3)	8	1.9 (0.7, 5.2)
Very or completely willing (3)	15	1.6 (0.7, 3.4)	2	0.2 (0.1, 1.0)	14	9.7 (4.9, 18.4)	5	2.1 (0.7, 6.2)
Non-response	15	1.7 (0.7, 3.9)	5	1.2 (0.3, 4.8)	3	1.5 (0.3, 6.5)	3	0.8 (0.2, 3.9)
Mean score‡	1037	*1.09 (1.05, 1.12)*	488	*1.08 (1.04, 1.12)*	165	*1.29 (1.13, 1.45)*	346	*1.07 (1.01, 1.14)*
aPD; q-value§		*0.37 (-1.34, 2.08); 1.00*		*-0.97 (-2.29, 0.34); 1.00*		*6.84 (0.41, 13.27); 1.00*		*1.09 (-1.39, 3.58); 1.00*
A person who does not share your race or ethnicity								
Not asked the question†	128	11.7 (9.5, 14.4)	71	14.4 (10.7, 19.3)	27	19.7 (12.9, 28.7)	66	18.6 (13.7, 24.9)
Not willing (1)	989	81.5 (78.1, 84.4)	473	80.4 (75.0, 84.8)	145	66.1 (56.1, 74.8)	334	77.9 (71.5, 83.2)
Somewhat willing (2)	35	3.8 (2.3, 6.2)	11	2.6 (1.2, 5.9)	9	7.3 (3.1, 16.4)	12	2.8 (1.2, 6.4)
Very or completely willing (3)	11	1.8 (0.9, 3.5)	3	0.8 (0.2, 3.1)	12	6.6 (3.3, 12.9)	1	0.4 (0.1, 2.5)
Non-response	17	1.3 (0.7, 2.2)	6	1.7 (0.6, 5.0)	2	0.4 (0.1, 1.7)	2	0.3 (0.1, 1.3)
Mean score‡	1035	*1.08 (1.05, 1.12)*	487	*1.05 (1.01, 1.09)*	166	*1.26 (1.13, 1.39)*	347	*1.04 (1.01, 1.08)*
aPD; q-value§		*0.84 (-1.01, 2.69); 1.00*		*-0.12 (-2.04, 1.79); 1.00*		*3.19 (-1.88, 8.25); 1.00*		*-0.67 (-2.17, 0.83); 1.00*
A person who does not share your religion								
Not asked the question†	128	11.7 (9.5, 14.4)	71	14.4 (10.7, 19.3)	27	19.7 (12.9, 28.7)	66	18.6 (13.7, 24.9)
Not willing (1)	990	81.5 (78.0, 84.5)	472	79.6 (74.1, 84.2)	144	64.5 (54.3, 73.4)	334	77.6 (71.2, 82.9)
Somewhat willing (2)	39	4.3 (2.7, 6.8)	12	2.1 (1.1, 3.9)	12	8.7 (4.1, 17.6)	9	1.9 (1.0, 3.9)
Very or completely willing (3)	10	1.6 (0.6, 4.1)	2	1.4 (0.3, 5.8)	10	6.8 (3.0, 14.6)	3	1.5 (0.3, 6.2)
Non-response	13	0.9 (0.5, 1.7)	7	2.4 (0.9, 6.5)	2	0.4 (0.1, 1.7)	3	0.4 (0.1, 1.3)
Mean score‡	1039	*1.09 (1.05, 1.13)*	486	*1.06 (1.01, 1.11)*	166	*1.28 (1.13, 1.42)*	346	*1.06 (1.01, 1.12)*
aPD; q-value§		*1.46 (-0.52, 3.43); 1.00*		*1.36 (-0.99, 3.70); 1.00*		*4.21 (-1.54, 9.95); 1.00*		*1.18 (-1.12, 3.48); 1.00*
A person who does not share your political beliefs								
Not asked the question†	128	11.7 (9.5, 14.4)	71	14.4 (10.7, 19.3)	27	19.7 (12.9, 28.7)	66	18.6 (13.7, 24.9)
Not willing (1)	971	80.2 (76.7, 83.2)	467	78.1 (72.4, 82.8)	142	61.3 (51.2, 70.6)	328	76.0 (69.4, 81.5)
Somewhat willing (2)	50	5.2 (3.5, 7.8)	19	4.5 (2.5, 8.0)	13	12.4 (6.4, 22.8)	13	3.9 (1.8, 8.2)
Very or completely willing (3)	12	1.5 (0.8, 3.0)	3	1.5 (0.4, 5.3)	11	6.2 (3.1, 12.3)	4	1.0 (0.4, 2.9)
Non-response	19	1.3 (0.8, 2.3)	4	1.5 (0.4, 5.1)	2	0.4 (0.1, 1.7)	4	0.4 (0.1, 1.3)
Mean score‡	1033	*1.10 (1.06, 1.13)*	489	*1.09 (1.04, 1.14)*	166	*1.31 (1.18, 1.44)*	345	*1.07 (1.03, 1.12)*
aPD; q-value§		*-0.02 (-1.91, 1.88); 1.00*		*0.07 (-2.58, 2.73); 1.00*		*2.23 (-2.62, 7.08); 1.00*		*-0.73 (-2.56, 1.10); 1.00*

Table S14, continued.

In a situation where you think force or violence is justified to advance an important political objective...How willing would you personally be to use force or violence in each of these ways?	Population Estimates by Party Category for 2025*							
	No MAGA Affiliation							
	Non-MAGA, leans or not strong Republican (n = 1083)		Non-MAGA, Independent (n = 932)		Non-MAGA, leans or not strong Democrat (n = 1849)		Non-MAGA, strong Democrat (n = 1332)	
	Unweighted n	Weighted % (95% CI) *Mean score (95% CI)*	Unweighted n	Weighted % (95% CI) *Mean score (95% CI)*	Unweighted n	Weighted % (95% CI) *Mean score (95% CI)*	Unweighted n	Weighted % (95% CI) *Mean score (95% CI)*
An elected federal or state government official								
Not asked the question†	213	24.3 (20.9, 28.1)	218	26.8 (23.1, 30.9)	434	24.4 (21.8, 27.1)	362	25.4 (22.6, 28.5)
Not willing (1)	818	71.1 (67.2, 74.7)	638	63.4 (59.0, 67.5)	1295	68.4 (65.5, 71.2)	903	68.1 (64.7, 71.3)
Somewhat willing (2)	37	3.0 (2.0, 4.5)	38	5.9 (4.0, 8.7)	84	4.4 (3.3, 5.7)	39	4.0 (2.6, 6.2)
Very or completely willing (3)	6	1.0 (0.4, 2.6)	23	2.3 (1.4, 3.8)	22	2.0 (1.2, 3.4)	15	1.7 (0.9, 3.1)
Non-response	7	0.6 (0.3, 1.4)	8	1.6 (0.7, 3.5)	11	0.8 (0.4, 1.9)	9	0.8 (0.3, 1.8)
Mean score‡	861	*1.07 (1.04, 1.10)*	699	*1.15 (1.10, 1.19)*	1401	*1.11 (1.08, 1.14)*	957	*1.10 (1.07, 1.14)*
aPD; q-value§		*-0.19 (-1.85, 1.48); 1.00*		*0.25 (-1.46, 1.96); 1.00*		*-0.03 (-1.58, 1.51); 1.00*		*Reference*
An elected local government official								
Not asked the question†	213	24.3 (20.9, 28.1)	218	26.8 (23.1, 30.9)	434	24.4 (21.8, 27.1)	362	25.4 (22.6, 28.5)
Not willing (1)	822	71.2 (67.3, 74.9)	647	64.7 (60.4, 68.8)	1311	69.2 (66.3, 72.0)	907	68.4 (65.0, 71.6)
Somewhat willing (2)	27	2.3 (1.4, 3.8)	30	4.0 (2.5, 6.2)	64	3.2 (2.3, 4.4)	37	3.4 (2.2, 5.2)
Very or completely willing (3)	7	1.2 (0.5, 3.0)	18	2.6 (1.4, 4.6)	25	2.2 (1.3, 3.5)	16	2.3 (1.2, 4.1)
Non-response	12	0.9 (0.5, 1.7)	12	1.9 (1.0, 3.8)	12	1.0 (0.5, 2.2)	6	0.6 (0.2, 1.6)
Mean score‡	856	*1.06 (1.03, 1.10)*	695	*1.13 (1.08, 1.18)*	1400	*1.10 (1.07, 1.13)*	960	*1.11 (1.07, 1.15)*
aPD; q-value§		*0.35 (-1.54, 2.23); 1.00*		*-0.19 (-2.33, 1.96); 1.00*		*-0.25 (-2.01, 1.50); 1.00*		*Reference*
An election worker, such as a poll worker or vote counter								
Not asked the question†	213	24.3 (20.9, 28.1)	218	26.8 (23.1, 30.9)	434	24.4 (21.8, 27.1)	362	25.4 (22.6, 28.5)
Not willing (1)	833	72.0 (68.1, 75.6)	654	64.5 (60.2, 68.6)	1353	71.0 (68.1, 73.7)	930	70.0 (66.7, 73.2)
Somewhat willing (2)	24	2.3 (1.3, 3.8)	29	4.3 (2.8, 6.5)	33	2.4 (1.6, 3.7)	21	2.4 (1.4, 4.2)
Very or completely willing (3)	3	0.7 (0.2, 2.4)	13	2.4 (1.2, 4.7)	17	1.6 (0.9, 2.8)	10	1.3 (0.6, 2.7)
Non-response	8	0.7 (0.3, 1.5)	11	2.0 (1.0, 3.9)	9	0.6 (0.3, 1.6)	5	0.8 (0.3, 2.1)
Mean score‡	860	*1.05 (1.02, 1.08)*	696	*1.13 (1.08, 1.18)*	1403	*1.07 (1.05, 1.10)*	961	*1.07 (1.04, 1.10)*
aPD; q-value§		*0.31 (-1.22, 1.83); 1.00*		*0.83 (-1.16, 2.82); 1.00*		*0.25 (-1.17, 1.68); 1.00*		*Reference*
A public health official								
Not asked the question†	213	24.3 (20.9, 28.1)	218	26.8 (23.1, 30.9)	434	24.4 (21.8, 27.1)	362	25.4 (22.6, 28.5)
Not willing (1)	826	71.3 (67.4, 75.0)	652	64.9 (60.6, 69.0)	1347	70.5 (67.6, 73.3)	929	69.8 (66.4, 72.9)
Somewhat willing (2)	27	2.8 (1.7, 4.5)	23	3.6 (2.2, 5.8)	36	2.7 (1.8, 4.1)	14	1.2 (0.6, 2.6)
Very or completely willing (3)	4	0.7 (0.2, 2.4)	19	2.6 (1.4, 4.6)	18	1.7 (1.0, 2.9)	16	2.6 (1.5, 4.6)
Non-response	11	0.8 (0.4, 1.6)	13	2.1 (1.1, 3.8)	11	0.8 (0.3, 1.7)	7	0.9 (0.4, 2.2)
Mean score‡	857	*1.06 (1.03, 1.08)*	694	*1.12 (1.08, 1.17)*	1401	*1.08 (1.05, 1.11)*	959	*1.09 (1.05, 1.13)*
aPD; q-value§		*-0.99 (-2.86, 0.87); 1.00*		*-0.85 (-3.07, 1.37); 1.00*		*-1.37 (-3.12, 0.37); 1.00*		*Reference*
A member of the military or National Guard								
Not asked the question†	213	24.3 (20.9, 28.1)	218	26.8 (23.1, 30.9)	434	24.4 (21.8, 27.1)	362	25.4 (22.6, 28.5)
Not willing (1)	824	71.8 (67.9, 75.4)	632	63.4 (59.1, 67.5)	1324	69.4 (66.5, 72.2)	904	68.0 (64.6, 71.3)
Somewhat willing (2)	35	3.2 (2.0, 5.0)	44	5.2 (3.6, 7.7)	51	3.0 (2.1, 4.3)	40	3.8 (2.5, 5.7)
Very or completely willing (3)	3	0.1 (0.0, 0.4)	21	2.6 (1.5, 4.5)	25	2.5 (1.5, 3.9)	14	1.8 (0.9, 3.4)
Non-response	6	0.6 (0.2, 1.4)	10	1.8 (0.9, 3.8)	12	0.7 (0.3, 1.6)	8	1.0 (0.4, 2.5)
Mean score‡	862	*1.05 (1.03, 1.06)*	697	*1.15 (1.10, 1.19)*	1400	*1.11 (1.07, 1.14)*	958	*1.10 (1.06, 1.14)*
aPD‡		*-0.97 (-2.19, 0.25); 1.00*		*0.39 (-1.52, 2.29); 1.00*		*0.30 (-1.34, 1.95); 1.00*		*Reference*

Table S14, continued.

In a situation where you think force or violence is justified to advance an important political objective...How willing would you personally be to use force or violence in each of these ways?	**Population Estimates by Party Category for 2025***							
	**No MAGA Affiliation**							
	**Non-MAGA, leans or not strong Republican (n = 1083)**		**Non-MAGA, Independent (n = 932)**		**Non-MAGA, leans or not strong Democrat (n = 1849)**		**Non-MAGA, strong Democrat (n = 1332)**	
	**Unweighted n**	**Weighted % (95% CI)** ***Mean score (95% CI)***	**Unweighted n**	**Weighted % (95% CI)** ***Mean score (95% CI)***	**Unweighted n**	**Weighted % (95% CI)** ***Mean score (95% CI)***	**Unweighted n**	**Weighted % (95% CI)** ***Mean score (95% CI)***
A police officer								
Not asked the question†	213	24.3 (20.9, 28.1)	218	26.8 (23.1, 30.9)	434	24.4 (21.8, 27.1)	362	25.4 (22.6, 28.5)
Not willing (1)	810	70.1 (66.2, 73.8)	615	59.9 (55.5, 64.1)	1287	66.9 (63.9, 69.8)	886	66.7 (63.3, 69.9)
Somewhat willing (2)	36	3.6 (2.4, 5.5)	56	7.7 (5.4, 10.8)	84	5.5 (4.1, 7.2)	58	5.7 (4.1, 7.9)
Very or completely willing (3)	9	1.0 (0.4, 2.5)	24	3.8 (2.3, 6.2)	28	2.4 (1.5, 3.8)	15	1.6 (0.8, 2.9)
Non-response	13	1.0 (0.5, 1.8)	12	1.8 (0.9, 3.5)	13	0.9 (0.4, 1.9)	7	0.6 (0.2, 1.6)
Mean score‡	855	*1.07 (1.04, 1.11)*	695	*1.21 (1.15, 1.27)*	1399	*1.14 (1.10, 1.17)*	959	*1.12 (1.08, 1.15)*
aPD; q-value§		*-0.13 (-1.68, 1.42); 1.00*		*1.77 (-0.39, 3.93); 1.00*		*0.62 (-0.88, 2.12); 1.00*		*Reference*
A person who does not share your race or ethnicity								
Not asked the question†	213	24.3 (20.9, 28.1)	218	26.8 (23.1, 30.9)	434	24.4 (21.8, 27.1)	362	25.4 (22.6, 28.5)
Not willing (1)	834	72.3 (68.4, 75.9)	662	66.3 (62.0, 70.4)	1353	71.4 (68.5, 74.1)	935	70.8 (67.5, 73.9)
Somewhat willing (2)	17	1.4 (0.7, 2.8)	26	4.2 (2.5, 6.7)	29	1.9 (1.2, 3.0)	8	1.0 (0.5, 2.3)
Very or completely willing (3)	7	1.1 (0.5, 2.8)	13	1.6 (0.8, 3.2)	20	1.7 (1.0, 3.0)	14	1.9 (1.0, 3.5)
Non-response	10	0.8 (0.4, 1.6)	6	1.1 (0.4, 2.8)	10	0.6 (0.2, 1.6)	9	0.8 (0.4, 1.9)
Mean score‡	858	*1.05 (1.02, 1.08)*	701	*1.10 (1.06, 1.14)*	1402	*1.07 (1.04, 1.10)*	957	*1.07 (1.03, 1.10)*
aPD; q-value§		*0.03 (-1.74, 1.80); 1.00*		*-0.68 (-2.42, 1.06); 1.00*		*-0.33 (-1.92, 1.26); 1.00*		*Reference*
A person who does not share your religion								
Not asked the question†	213	24.3 (20.9, 28.1)	218	26.8 (23.1, 30.9)	434	24.4 (21.8, 27.1)	362	25.4 (22.6, 28.5)
Not willing (1)	835	72.4 (68.5, 76.0)	662	66.1 (61.8, 70.2)	1347	70.9 (68.0, 73.6)	938	71.4 (68.1, 74.4)
Somewhat willing (2)	18	1.5 (0.7, 2.8)	28	3.6 (2.2, 5.8)	35	2.5 (1.6, 3.8)	12	1.7 (0.8, 3.2)
Very or completely willing (3)	7	1.1 (0.4, 2.7)	9	1.7 (0.8, 3.7)	18	1.6 (0.9, 2.8)	9	1.0 (0.4, 2.2)
Non-response	8	0.7 (0.3, 1.5)	8	1.7 (0.8, 3.8)	12	0.7 (0.3, 1.6)	7	0.6 (0.2, 1.6)
Mean score‡	860	*1.05 (1.02, 1.08)*	699	*1.10 (1.05, 1.14)*	1400	*1.07 (1.05, 1.10)*	959	*1.05 (1.02, 1.07)*
aPD; q-value§		*0.94 (-0.60, 2.48); 1.00*		*0.33 (-1.28, 1.93); 1.00*		*0.54 (-0.77, 1.85); 1.00*		*Reference*
A person who does not share your political beliefs								
Not asked the question†	213	24.3 (20.9, 28.1)	218	26.8 (23.1, 30.9)	434	24.4 (21.8, 27.1)	362	25.4 (22.6, 28.5)
Not willing (1)	830	71.8 (67.9, 75.4)	646	64.5 (60.2, 68.6)	1320	69.2 (66.2, 72.0)	916	68.8 (65.4, 72.0)
Somewhat willing (2)	27	2.7 (1.6, 4.4)	35	4.5 (2.9, 6.8)	64	4.0 (2.9, 5.5)	27	2.7 (1.6, 4.4)
Very or completely willing (3)	4	0.6 (0.2, 2.3)	17	2.7 (1.5, 4.8)	20	1.8 (1.1, 3.1)	17	2.4 (1.4, 4.3)
Non-response	7	0.6 (0.3, 1.4)	9	1.4 (0.7, 3.1)	8	0.6 (0.2, 1.6)	6	0.6 (0.2, 1.7)
Mean score‡	861	*1.05 (1.02, 1.08)*	698	*1.14 (1.09, 1.19)*	1404	*1.10 (1.07, 1.13)*	960	*1.10 (1.06, 1.14)*
aPD; q-value§		*-1.16 (-2.96, 0.64); 1.00*		*-0.29 (-2.48, 1.91); 1.00*		*-0.92 (-2.69, 0.84); 1.00*		*Reference*

* Among respondents to both the 2024 and 2025 surveys (n=7767). Prevalences for 2024 among respondents to that survey were reported previously: Wintemute GJ, Velasquez B, Robinson SL, Tomsich EA, Wright MA, Shev AB. The MAGA movement and political violence in 2024: findings from a nationally representative survey. Inj Epidemiol. 2025;12(1):78. Respondents who did not answer the question "In general...to advance an important political objective that you support" in 2024 (n = 58) or 2025 (n = 57) were not asked these questions.

† Respondents answered “never justified” to all prior questions on the use of force or violence to advance specific political objectives were not asked questions on their personal willingness to use political violence.

‡ Mean scores in 2024 and 2025 were scored as indicated in the response lines for individual questions, with non-responses excluded.

§ Adjusted prevalence differences (aPDs) are absolute percentage point (pp) differences for “very or completely willing” responses and are adjusted for age, race and ethnicity, gender, income, education, Census division, marital status, homeownership, rurality, firearm ownership, alcohol consumption, military service, and history of non-traffic arrest. Q-values, also known as FDR-adjusted (or FDR-corrected) p-values, represent the probability that the given difference would be a false discovery; they represent the expected proportion of “false positives” that would be seen among the collection of all differences whose q-values were at or below the given q-value. Item non-responses are not reported in the tables but are included in the prevalence calculations.

Table S15. Party/MAGA affiliation and 2024-2025 change in personal willingness to engage in political violence, by target population

In a situation where you think force or violence is justified to advance an important political objective...How willing would you personally be to use force or violence in each of these ways?	Mean Differences* by Party Category 2024-2025							
	MAGA Affiliation						No MAGA Affiliation	
	MAGA Republican (n = 1183)		MAGA Supporter, Republican (n = 565)		MAGA Supporter, Non-Republican (n = 197)		Non-MAGA, strong Republican (n = 417)	
	Unweighted n	Weighted mean (95% CI) *Mean score (95% CI)*	Unweighted n	Weighted mean (95% CI) *Mean score (95% CI)*	Unweighted n	Weighted mean (95% CI) *Mean score (95% CI)*	Unweighted n	Weighted mean (95% CI) *Mean score (95% CI)*
An elected federal or state government official								
Not asked the question†	1179	0.2 (-3.0, 3.4)	563	1.7 (-3.4, 6.9)	195	-8.3 (-20.0, 3.5)	415	-0.5 (-6.7, 5.7)
Not willing (1)	1179	0.8 (-3.2, 4.8)	563	-2.3 (-8.8, 4.2)	195	10.9 (-0.6, 22.4)	415	2.2 (-4.7, 9.1)
Somewhat willing (2)	1179	-1.8 (-4.7, 1.0)	563	-0.1 (-3.7, 3.6)	195	1.9 (-3.9, 7.8)	415	-0.7 (-4.4, 2.9)
Very or completely willing (3)	1179	0.6 (-1.6, 2.8)	563	-0.4 (-2.1, 1.3)	195	-3.6 (-8.8, 1.7)	415	-1.7 (-4.5, 1.0)
Non-response	1179	0.2 (-0.5, 0.9)	563	1.0 (-0.9, 2.9)	195	-1.0 (-4.1, 2.1)	415	0.7 (-0.7, 2.2)
Mean score‡	967	*-0.003 (-0.048, 0.042)*	457	*-0.012 (-0.069, 0.045)*	135	*-0.082 (-0.179, 0.015)*	297	*-0.038 (-0.097, 0.021)*
An elected local government official								
Not asked the question†	1179	0.2 (-3.0, 3.4)	563	1.7 (-3.4, 6.9)	195	-8.3 (-20.0, 3.5)	415	-0.5 (-6.7, 5.7)
Not willing (1)	1179	0.5 (-3.5, 4.6)	563	-2.6 (-8.8, 3.7)	195	7.9 (-4.9, 20.7)	415	1.8 (-4.9, 8.5)
Somewhat willing (2)	1179	-1.3 (-3.6, 1.0)	563	-0.5 (-3.6, 2.7)	195	1.8 (-2.7, 6.2)	415	-0.9 (-4.6, 2.7)
Very or completely willing (3)	1179	0.3 (-1.8, 2.5)	563	1.1 (-1.2, 3.4)	195	-0.1 (-6.7, 6.5)	415	-1.5 (-3.7, 0.8)
Non-response	1179	0.2 (-0.7, 1.0)	563	0.2 (-0.6, 1.0)	195	-1.3 (-4.4, 1.7)	415	1.1 (-1.0, 3.1)
Mean score‡	958	*-0.001 (-0.051, 0.048)*	458	*0.022 (-0.043, 0.086)*	136	*0.031 (-0.161, 0.222)*	298	*-0.043 (-0.098, 0.012)*
An election worker, such as a poll worker or vote counter								
Not asked the question†	1179	0.2 (-3.0, 3.4)	563	1.7 (-3.4, 6.9)	195	-8.3 (-20.0, 3.5)	415	-0.5 (-6.7, 5.7)
Not willing (1)	1179	-1.2 (-5.3, 2.9)	563	-5.0 (-11.1, 1.1)	195	10.7 (-0.5, 22.0)	415	1.5 (-5.4, 8.3)
Somewhat willing (2)	1179	0.3 (-2.3, 3.0)	563	2.1 (-0.7, 4.9)	195	1.9 (-5.5, 9.4)	415	-2.2 (-5.8, 1.4)
Very or completely willing (3)	1179	0.0 (-2.1, 2.1)	563	0.2 (-0.4, 0.7)	195	-3.1 (-9.3, 3.2)	415	0.4 (-2.2, 2.9)
Non-response	1179	0.6 (-0.3, 1.5)	563	1.0 (-0.8, 2.7)	195	-1.3 (-4.4, 1.7)	415	0.9 (-0.6, 2.4)
Mean score‡	961	*0.005 (-0.041, 0.050)*	459	*0.034 (-0.005, 0.072)*	136	*-0.104 (-0.238, 0.030)*	298	*0.001 (-0.054, 0.055)*
A public health official								
Not asked the question†	1179	0.2 (-3.0, 3.4)	563	1.7 (-3.4, 6.9)	195	-8.3 (-20.0, 3.5)	415	-0.5 (-6.7, 5.7)
Not willing (1)	1179	1.7 (-2.4, 5.8)	563	-3.0 (-9.2, 3.3)	195	6.5 (-6.8, 19.9)	415	0.8 (-6.2, 7.9)
Somewhat willing (2)	1179	-2.2 (-4.1, -0.3)	563	2.7 (-0.7, 6.0)	195	-1.8 (-9.8, 6.2)	415	-0.4 (-3.7, 2.9)
Very or completely willing (3)	1179	0.1 (-2.3, 2.5)	563	-1.5 (-3.6, 0.6)	195	3.8 (-4.9, 12.6)	415	-0.7 (-2.3, 1.0)
Non-response	1179	0.2 (-0.7, 1.0)	563	0.1 (-0.7, 0.8)	195	-0.2 (-4.0, 3.5)	415	0.7 (-0.7, 2.1)
Mean score‡	958	*-0.022 (-0.079, 0.035)*	460	*-0.004 (-0.065, 0.056)*	135	*0.069 (-0.184, 0.323)*	298	*-0.002 (-0.056, 0.052)*
A member of the military or National Guard								
Not asked the question†	1179	0.2 (-3.0, 3.4)	563	1.7 (-3.4, 6.9)	195	-8.3 (-20.0, 3.5)	415	-0.5 (-6.7, 5.7)
Not willing (1)	1179	1.1 (-2.6, 4.8)	563	-4.0 (-10.5, 2.6)	195	8.0 (-4.5, 20.4)	415	1.9 (-5.3, 9.1)
Somewhat willing (2)	1179	-1.4 (-3.6, 0.8)	563	1.0 (-1.9, 4.0)	195	6.7 (-0.8, 14.1)	415	0.0 (-4.0, 4.0)
Very or completely willing (3)	1179	-0.1 (-1.9, 1.7)	563	1.3 (-0.7, 3.3)	195	-5.0 (-10.9, 0.9)	415	-2.0 (-4.4, 0.4)
Non-response	1179	0.2 (-0.8, 1.1)	563	-0.1 (-2.1, 1.9)	195	-1.3 (-4.4, 1.7)	415	0.5 (-0.9, 1.9)
Mean score‡	956	*-0.014 (-0.047, 0.019)*	455	*0.048 (-0.016, 0.111)*	136	*-0.056 (-0.186, 0.074)*	298	*-0.053 (-0.116, 0.009)*

Table S15, continued.

In a situation where you think force or violence is justified to advance an important political objective...How willing would you personally be to use force or violence in each of these ways?	Mean Differences* by Party Category 2024-2025							
	MAGA Affiliation						No MAGA Affiliation	
	MAGA Republican (n = 1183)		MAGA Supporter, Republican (n = 565)		MAGA Supporter, Non-Republican (n = 197)		Non-MAGA, strong Republican (n = 417)	
	**Unweighted n**	**Weighted mean (95% CI)** ***Mean score (95% CI)***	**Unweighted n**	**Weighted mean (95% CI)** ***Mean score (95% CI)***	**Unweighted n**	**Weighted mean (95% CI)** ***Mean score (95% CI)***	**Unweighted n**	**Weighted mean (95% CI)** ***Mean score (95% CI)***
A police officer								
Not asked the question†	1179	0.2 (-3.0, 3.4)	563	1.7 (-3.4, 6.9)	195	-8.3 (-20.0, 3.5)	415	-0.5 (-6.7, 5.7)
Not willing (1)	1179	0.4 (-3.6, 4.4)	563	-4.3 (-10.7, 2.0)	195	11.2 (-0.8, 23.1)	415	4.5 (-2.6, 11.6)
Somewhat willing (2)	1179	-0.6 (-3.4, 2.1)	563	3.4 (-0.4, 7.2)	195	-4.1 (-11.3, 3.0)	415	-3.6 (-7.0, -0.2)
Very or completely willing (3)	1179	-0.8 (-3.1, 1.5)	563	-0.6 (-1.8, 0.6)	195	2.8 (-2.8, 8.4)	415	-0.9 (-2.7, 1.0)
Non-response	1179	0.8 (-0.8, 2.4)	563	-0.2 (-2.6, 2.2)	195	-1.5 (-6.1, 3.0)	415	0.4 (-0.9, 1.8)
Mean score‡	959	*-0.011 (-0.052, 0.030)*	458	*0.017 (-0.029, 0.064)*	135	*0.051 (-0.076, 0.178)*	298	*-0.053 (-0.109, 0.002)*
A person who does not share your race or ethnicity								
Not asked the question†	1179	0.2 (-3.0, 3.4)	563	1.7 (-3.4, 6.9)	195	-8.3 (-20.0, 3.5)	415	-0.5 (-6.7, 5.7)
Not willing (1)	1179	0.6 (-3.4, 4.5)	563	-2.9 (-9.3, 3.5)	195	9.1 (-3.5, 21.7)	415	3.4 (-3.3, 10.1)
Somewhat willing (2)	1179	-0.1 (-2.3, 2.1)	563	0.7 (-1.5, 3.0)	195	3.4 (-4.7, 11.5)	415	-1.2 (-4.1, 1.7)
Very or completely willing (3)	1179	-1.3 (-3.6, 1.0)	563	0.2 (-1.5, 1.9)	195	-2.2 (-5.7, 1.4)	415	-1.3 (-2.9, 0.4)
Non-response	1179	0.6 (-0.2, 1.4)	563	0.2 (-2.3, 2.7)	195	-2.1 (-5.5, 1.3)	415	-0.4 (-1.4, 0.5)
Mean score‡	960	*-0.030 (-0.079, 0.018)*	457	*0.015 (-0.038, 0.068)*	136	*-0.049 (-0.191, 0.094)*	298	*-0.032 (-0.076, 0.012)*
A person who does not share your religion								
Not asked the question†	1179	0.2 (-3.0, 3.4)	563	1.7 (-3.4, 6.9)	195	-8.3 (-20.0, 3.5)	415	-0.5 (-6.7, 5.7)
Not willing (1)	1179	0.4 (-3.2, 4.0)	563	-4.0 (-10.1, 2.1)	195	4.7 (-7.8, 17.2)	415	2.7 (-4.0, 9.4)
Somewhat willing (2)	1179	-0.1 (-2.5, 2.3)	563	0.7 (-0.7, 2.1)	195	7.5 (1.0, 13.9)	415	-2.7 (-6.3, 0.9)
Very or completely willing (3)	1179	-0.6 (-2.6, 1.4)	563	0.6 (-1.8, 3.0)	195	-2.6 (-10.1, 5.0)	415	0.4 (-2.0, 2.8)
Non-response	1179	0.1 (-0.7, 0.9)	563	0.9 (-0.9, 2.7)	195	-1.3 (-4.4, 1.7)	415	0.0 (-0.7, 0.7)
Mean score‡	961	*-0.011 (-0.044, 0.021)*	457	*0.025 (-0.040, 0.091)*	136	*0.020 (-0.171, 0.210)*	299	*-0.001 (-0.053, 0.051)*
A person who does not share your political beliefs								
Not asked the question†	1179	0.2 (-3.0, 3.4)	563	1.7 (-3.4, 6.9)	195	-8.3 (-20.0, 3.5)	415	-0.5 (-6.7, 5.7)
Not willing (1)	1179	-0.7 (-4.5, 3.1)	563	-3.8 (-10.0, 2.5)	195	5.8 (-5.3, 16.8)	415	1.4 (-5.3, 8.1)
Somewhat willing (2)	1179	0.2 (-2.0, 2.4)	563	1.8 (-1.0, 4.7)	195	3.3 (-2.1, 8.8)	415	0.3 (-3.3, 3.8)
Very or completely willing (3)	1179	-0.3 (-2.0, 1.4)	563	0.1 (-2.5, 2.7)	195	0.5 (-3.0, 4.1)	415	-1.3 (-3.9, 1.3)
Non-response	1179	0.6 (-0.4, 1.5)	563	0.1 (-0.7, 0.8)	195	-1.3 (-4.4, 1.7)	415	0.1 (-0.6, 0.8)
Mean score‡	956	*-0.004 (-0.040, 0.033)*	460	*0.027 (-0.043, 0.097)*	136	*0.039 (-0.039, 0.118)*	298	*-0.014 (-0.068, 0.040)*

Table S15, continued.

In a situation where you think force or violence is justified to advance an important political objective...How willing would you personally be to use force or violence in each of these ways?	Mean Differences* by Party Category 2024-2025							
	No MAGA Affiliation							
	Non-MAGA, leans or not strong Republican (n = 1083)		Non-MAGA, Independent (n = 932)		Non-MAGA, leans or not strong Democrat (n = 1849)		Non-MAGA, strong Democrat (n = 1332)	
	**Unweighted n**	**Weighted mean (95% CI)** *Mean score (95% CI)*	**Unweighted n**	**Weighted mean (95% CI)** *Mean score (95% CI)*	**Unweighted n**	**Weighted mean (95% CI)** *Mean score (95% CI)*	**Unweighted n**	**Weighted mean (95% CI)** *Mean score (95% CI)*
An elected federal or state government official								
Not asked the question†	1078	0.0 (-4.3, 4.3)	920	-5.6 (-10.8, -0.4)	1845	-6.5 (-9.6, -3.4)	1326	-11.4 (-15.0, -7.8)
Not willing (1)	1078	-0.1 (-4.7, 4.6)	920	5.2 (-0.2, 10.5)	1845	5.5 (2.0, 9.0)	1326	9.9 (6.0, 13.9)
Somewhat willing (2)	1078	-0.4 (-2.3, 1.4)	920	0.3 (-2.1, 2.7)	1845	-0.3 (-2.1, 1.6)	1326	1.2 (-0.4, 2.8)
Very or completely willing (3)	1078	0.3 (-0.9, 1.4)	920	-1.0 (-2.9, 1.0)	1845	0.8 (-0.3, 1.9)	1326	0.0 (-1.3, 1.3)
Non-response	1078	0.3 (-0.2, 0.8)	920	1.1 (-0.1, 2.3)	1845	0.5 (-0.2, 1.2)	1326	0.3 (-0.6, 1.1)
Mean score‡	756	*0.001 (-0.044, 0.046)*	567	*-0.024 (-0.077, 0.029)*	1118	*-0.004 (-0.038, 0.031)*	713	*0.008 (-0.029, 0.044)*
An elected local government official								
Not asked the question†	1078	0.0 (-4.3, 4.3)	920	-5.6 (-10.8, -0.4)	1845	-6.5 (-9.6, -3.4)	1326	-11.4 (-15.0, -7.8)
Not willing (1)	1078	-0.4 (-5.3, 4.5)	920	6.3 (0.8, 11.7)	1845	5.6 (2.2, 9.0)	1326	9.7 (5.7, 13.7)
Somewhat willing (2)	1078	-1.1 (-3.1, 0.9)	920	-2.0 (-4.1, 0.1)	1845	-0.8 (-2.4, 0.8)	1326	1.1 (-0.4, 2.7)
Very or completely willing (3)	1078	1.0 (-0.2, 2.1)	920	-0.1 (-2.0, 1.9)	1845	0.8 (-0.4, 2.0)	1326	0.5 (-0.9, 1.9)
Non-response	1078	0.6 (0.0, 1.2)	920	1.4 (0.1, 2.7)	1845	0.9 (0.2, 1.6)	1326	0.0 (-0.8, 0.9)
Mean score‡	751	*0.010 (-0.038, 0.058)*	562	*-0.045 (-0.099, 0.009)*	1123	*-0.011 (-0.047, 0.025)*	713	*0.024 (-0.020, 0.067)*
An election worker, such as a poll worker or vote counter								
Not asked the question†	1078	0.0 (-4.3, 4.3)	920	-5.6 (-10.8, -0.4)	1845	-6.5 (-9.6, -3.4)	1326	-11.4 (-15.0, -7.8)
Not willing (1)	1078	-0.8 (-5.4, 3.8)	920	3.0 (-2.3, 8.3)	1845	6.1 (2.8, 9.3)	1326	10.4 (6.6, 14.3)
Somewhat willing (2)	1078	0.4 (-0.9, 1.7)	920	0.8 (-0.9, 2.6)	1845	0.5 (-0.8, 1.9)	1326	0.9 (-0.5, 2.3)
Very or completely willing (3)	1078	0.3 (-0.8, 1.3)	920	0.2 (-1.7, 2.0)	1845	-0.3 (-1.4, 0.8)	1326	-0.1 (-1.3, 1.0)
Non-response	1078	0.2 (-0.4, 0.8)	920	1.5 (0.2, 2.8)	1845	0.2 (-0.5, 0.9)	1326	0.2 (-0.8, 1.2)
Mean score‡	751	*0.012 (-0.026, 0.050)*	567	*0.020 (-0.031, 0.070)*	1124	*-0.017 (-0.044, 0.010)*	714	*0.006 (-0.031, 0.042)*
A public health official								
Not asked the question†	1078	0.0 (-4.3, 4.3)	920	-5.6 (-10.8, -0.4)	1845	-6.5 (-9.6, -3.4)	1326	-11.4 (-15.0, -7.8)
Not willing (1)	1078	-1.4 (-6.1, 3.2)	920	4.5 (-0.8, 9.7)	1845	6.0 (2.7, 9.3)	1326	10.0 (6.1, 13.9)
Somewhat willing (2)	1078	1.0 (-0.5, 2.5)	920	-1.1 (-3.1, 0.8)	1845	0.0 (-1.6, 1.6)	1326	0.0 (-1.1, 1.2)
Very or completely willing (3)	1078	0.2 (-0.8, 1.1)	920	1.0 (-0.7, 2.7)	1845	0.2 (-0.9, 1.3)	1326	1.0 (-0.3, 2.3)
Non-response	1078	0.3 (-0.4, 1.0)	920	1.3 (0.0, 2.5)	1845	0.3 (-0.4, 1.0)	1326	0.3 (-0.7, 1.4)
Mean score‡	750	*0.021 (-0.016, 0.058)*	565	*0.013 (-0.033, 0.058)*	1119	*-0.013 (-0.045, 0.020)*	714	*0.021 (-0.018, 0.060)*
A member of the military or National Guard								
Not asked the question†	1078	0.0 (-4.3, 4.3)	920	-5.6 (-10.8, -0.4)	1845	-6.5 (-9.6, -3.4)	1326	-11.4 (-15.0, -7.8)
Not willing (1)	1078	0.4 (-4.3, 5.2)	920	3.9 (-1.6, 9.3)	1845	6.1 (2.8, 9.4)	1326	9.2 (5.1, 13.3)
Somewhat willing (2)	1078	-0.3 (-2.3, 1.8)	920	0.5 (-1.4, 2.5)	1845	-0.8 (-2.4, 0.8)	1326	1.2 (-0.5, 2.9)
Very or completely willing (3)	1078	-0.2 (-0.6, 0.1)	920	0.2 (-1.4, 1.7)	1845	0.7 (-0.3, 1.7)	1326	0.6 (-0.4, 1.6)
Non-response	1078	0.0 (-0.5, 0.6)	920	1.0 (-0.4, 2.4)	1845	0.5 (-0.1, 1.1)	1326	0.4 (-0.7, 1.5)
Mean score‡	754	*-0.013 (-0.045, 0.020)*	564	*0.023 (-0.035, 0.080)*	1120	*-0.015 (-0.042, 0.013)*	713	*0.027 (-0.013, 0.067)*

Table S15, continued.

In a situation where you think force or violence is justified to advance an important political objective...How willing would you personally be to use force or violence in each of these ways?	Mean Differences* by Party Category 2024-2025							
	No MAGA Affiliation							
	Non-MAGA, leans or not strong Republican (n = 1083)		Non-MAGA, Independent (n = 932)		Non-MAGA, leans or not strong Democrat (n = 1849)		Non-MAGA, strong Democrat (n = 1332)	
	Unweighted n	Weighted mean (95% CI) *Mean score (95% CI)*	Unweighted n	Weighted mean (95% CI) *Mean score (95% CI)*	Unweighted n	Weighted mean (95% CI) *Mean score (95% CI)*	Unweighted n	Weighted mean (95% CI) *Mean score (95% CI)*
A police officer								
Not asked the question†	1078	0.0 (-4.3, 4.3)	920	-5.6 (-10.8, -0.4)	1845	-6.5 (-9.6, -3.4)	1326	-11.4 (-15.0, -7.8)
Not willing (1)	1078	-1.5 (-6.4, 3.3)	920	2.5 (-2.8, 7.9)	1845	5.0 (1.6, 8.4)	1326	11.1 (7.0, 15.2)
Somewhat willing (2)	1078	0.3 (-1.8, 2.4)	920	0.7 (-2.3, 3.7)	1845	0.7 (-1.2, 2.7)	1326	0.5 (-1.8, 2.8)
Very or completely willing (3)	1078	0.8 (-0.1, 1.7)	920	1.1 (-0.8, 3.0)	1845	0.1 (-1.1, 1.3)	1326	-0.5 (-1.7, 0.7)
Non-response	1078	0.4 (-0.2, 1.1)	920	1.2 (0.1, 2.3)	1845	0.7 (0.1, 1.4)	1326	0.3 (-0.5, 1.0)
Mean score‡	748	*0.025 (-0.019, 0.068)*	565	*0.033 (-0.025, 0.090)*	1121	*-0.013 (-0.049, 0.023)*	716	*-0.026 (-0.074, 0.022)*
A person who does not share your race or ethnicity								
Not asked the question†	1078	0.0 (-4.3, 4.3)	920	-5.6 (-10.8, -0.4)	1845	-6.5 (-9.6, -3.4)	1326	-11.4 (-15.0, -7.8)
Not willing (1)	1078	-0.3 (-4.9, 4.3)	920	5.9 (0.5, 11.3)	1845	6.1 (2.9, 9.3)	1326	10.9 (7.1, 14.8)
Somewhat willing (2)	1078	-0.9 (-2.3, 0.5)	920	-0.1 (-2.2, 1.9)	1845	-0.7 (-2.0, 0.7)	1326	-0.4 (-1.5, 0.7)
Very or completely willing (3)	1078	0.8 (-0.3, 1.9)	920	-0.6 (-1.9, 0.8)	1845	0.7 (-0.3, 1.7)	1326	0.5 (-0.5, 1.4)
Non-response	1078	0.4 (-0.1, 1.0)	920	0.4 (-0.7, 1.4)	1845	0.4 (-0.3, 1.0)	1326	0.4 (-0.5, 1.2)
Mean score‡	752	*0.009 (-0.031, 0.050)*	567	*-0.036 (-0.081, 0.009)*	1125	*-0.007 (-0.032, 0.017)*	713	*-0.014 (-0.044, 0.017)*
A person who does not share your religion								
Not asked the question†	1078	0.0 (-4.3, 4.3)	920	-5.6 (-10.8, -0.4)	1845	-6.5 (-9.6, -3.4)	1326	-11.4 (-15.0, -7.8)
Not willing (1)	1078	-0.2 (-4.8, 4.4)	920	6.0 (0.6, 11.4)	1845	5.3 (2.2, 8.5)	1326	11.7 (7.9, 15.5)
Somewhat willing (2)	1078	-0.4 (-1.7, 0.9)	920	-0.6 (-2.6, 1.3)	1845	0.6 (-0.6, 1.7)	1326	-0.4 (-1.9, 1.1)
Very or completely willing (3)	1078	0.6 (-0.6, 1.7)	920	-1.0 (-3.0, 1.0)	1845	0.3 (-0.8, 1.4)	1326	0.0 (-0.9, 0.9)
Non-response	1078	0.0 (-0.6, 0.7)	920	1.2 (-0.2, 2.5)	1845	0.3 (-0.3, 1.0)	1326	0.1 (-0.7, 0.9)
Mean score‡	751	*0.010 (-0.031, 0.052)*	568	*-0.048 (-0.107, 0.011)*	1121	*-0.004 (-0.026, 0.018)*	714	*-0.019 (-0.052, 0.014)*
A person who does not share your political beliefs								
Not asked the question†	1078	0.0 (-4.3, 4.3)	920	-5.6 (-10.8, -0.4)	1845	-6.5 (-9.6, -3.4)	1326	-11.4 (-15.0, -7.8)
Not willing (1)	1078	-0.9 (-5.6, 3.7)	920	5.1 (-0.2, 10.5)	1845	6.1 (2.8, 9.3)	1326	11.1 (7.1, 15.0)
Somewhat willing (2)	1078	0.5 (-1.0, 2.1)	920	-0.4 (-2.6, 1.7)	1845	-0.1 (-1.5, 1.4)	1326	-0.5 (-2.0, 1.0)
Very or completely willing (3)	1078	0.2 (-0.8, 1.2)	920	0.4 (-1.2, 2.0)	1845	0.4 (-0.6, 1.4)	1326	0.7 (-0.8, 2.2)
Non-response	1078	0.2 (-0.3, 0.8)	920	0.4 (-0.7, 1.6)	1845	0.1 (-0.6, 0.9)	1326	0.1 (-0.7, 1.0)
Mean score‡	755	*0.012 (-0.027, 0.051)*	564	*-0.002 (-0.052, 0.048)*	1125	*-0.016 (-0.042, 0.011)*	714	*0.003 (-0.045, 0.052)*

* Among respondents to both the 2024 and 2025 surveys (n=7767). Respondents who did not answer the question "In general...to advance an important political objective that you support" in 2024 (n = 58) or 2025 (n = 57) were not asked these questions.

† Respondents answered “never justified” to all prior questions on the use of force or violence to advance specific political objectives were not asked questions on their personal willingness to use political violence.

‡ To assess population-level change from 2024 to 2025, we computed within-individual change scores for each item and then calculated year-to-year population-level change scores based on the means of aggregated within-individual change scores. Mean change scores have a range from -2 to 2 (with 0 indicating no change).

Table S16. Party/MAGA affiliation and 2025 prevalence of personal willingness to commit political violence, by social context of violence

You agreed that the use of force or violence could be justified to advance (one/some) of the political objectives we just discussed. In (that/those) (situation/situations), how willing would you personally be to…	Population Estimates by Party Category for 2025*							
	MAGA Affiliation						No MAGA Affiliation	
	MAGA Republican (n = 1183)		MAGA Supporter, Republican (n = 565)		MAGA Supporter, Non-Republican (n = 197)		Non-MAGA, strong Republican (n = 417)	
	Unweighted n	Weighted % (95% CI) *Mean score (95% CI)*	Unweighted n	Weighted % (95% CI) *Mean score (95% CI)*	Unweighted n	Weighted % (95% CI) *Mean score (95% CI)*	Unweighted n	Weighted % (95% CI) *Mean score (95% CI)*
Use force or violence as part of a group of people who share your beliefs								
Not asked the question†	128	11.7 (9.3, 14.2)	71	14.4 (10.2, 18.7)	27	19.5 (11.7, 27.3)	66	18.6 (13.0, 24.2)
Not willing (1)	813	68.2 (64.6, 71.9)	391	67.1 (61.4, 72.7)	120	54.3 (44.5, 64.0)	290	66.4 (59.9, 72.8)
Somewhat willing (2)	196	15.4 (12.5, 18.2)	90	15.5 (11.4, 19.5)	29	13.2 (6.8, 19.7)	46	9.3 (5.7, 12.9)
Very or completely willing (3)	32	3.3 (1.8, 4.7)	8	1.0 (0.2, 1.9)	17	12.0 (4.3, 19.6)	10	3.9 (0.8, 7.0)
Non-response	14	1.4 (0.5, 2.4)	5	2.0 (0.0, 5.0)	4	1.0 (0.0, 2.2)	5	1.8 (0.0, 3.7)
Mean score‡	1169	*1.10 (1.05, 1.15)*	560	*1.03 (0.96, 1.10)*	193	*1.18 (0.98, 1.37)*	412	*0.98 (0.89, 1.08)*
aPD; q-value§		*2.28 (0.12, 4.43); 1.00*		*-0.04 (-1.96, 1.88); 1.00*		*7.81 (0.51, 15.11); 1.00*		*2.3 (-0.91, 5.51); 1.00*
Use force or violence on your own, as an individual								
Not asked the question†	128	11.7 (9.3, 14.2)	71	14.4 (10.2, 18.7)	27	19.5 (11.7, 27.3)	66	18.6 (13.0, 24.2)
Not willing (1)	722	60.9 (57.1, 64.6)	361	61.3 (55.4, 67.1)	113	51.5 (41.8, 61.3)	270	63.0 (56.6, 69.5)
Somewhat willing (2)	259	19.7 (16.8, 22.7)	110	20.9 (15.7, 26.1)	31	15.6 (7.5, 23.7)	66	14.0 (9.6, 18.4)
Very or completely willing (3)	60	6.3 (4.0, 8.5)	19	2.9 (1.1, 4.8)	22	12.3 (5.5, 19.1)	12	3.5 (1.1, 5.9)
Non-response	14	1.4 (0.5, 2.4)	4	0.5 (0.0, 1.1)	4	1.0 (0.0, 2.2)	3	0.9 (0.0, 2.2)
Mean score‡	1169	*1.21 (1.15, 1.27)*	561	*1.12 (1.04, 1.21)*	193	*1.21 (1.02, 1.40)*	414	*1.02 (0.93, 1.12)*
aPD; q-value§		*5.01 (2.24, 7.79); 0.04*		*1.67 (-0.97, 4.31); 1.00*		*7.84 (0.97, 14.70); 0.92*		*2.34 (-0.52, 5.19); 1.00*
Organize a group of people who share your beliefs to use force or violence								
Not asked the question†	128	11.7 (9.3, 14.2)	71	14.4 (10.2, 18.7)	27	19.5 (11.7, 27.3)	66	18.6 (13.0, 24.2)
Not willing (1)	900	75.0 (71.6, 78.4)	441	73.9 (68.3, 79.5)	134	62.4 (52.9, 71.9)	306	70.0 (63.6, 76.3)
Somewhat willing (2)	118	10.0 (7.5, 12.5)	39	6.7 (3.8, 9.7)	20	9.3 (3.7, 15.0)	32	7.5 (3.8, 11.1)
Very or completely willing (3)	25	2.3 (1.1, 3.5)	9	2.9 (0.4, 5.4)	12	7.7 (1.8, 13.7)	10	3.0 (0.6, 5.5)
Non-response	12	1.0 (0.3, 1.6)	5	2.0 (0.0, 5.0)	4	1.0 (0.0, 2.2)	3	0.9 (0.0, 2.2)
Mean score‡	1171	*1.03 (0.99, 1.07)*	560	*0.98 (0.90, 1.06)*	193	*1.05 (0.89, 1.22)*	414	*0.95 (0.86, 1.04)*
aPD; q-value§		*0.48 (-1.58, 2.55); 1.00*		*1.26 (-1.86, 4.39); 1.00*		*3.06 (-2.92, 9.04); 1.00*		*1.18 (-1.72, 4.08); 1.00*

Table S16, continued.

You agreed that the use of force or violence could be justified to advance (one/some) of the political objectives we just discussed. In (that/those) (situation/situations), how willing would <u>you personally</u> be to…	Population Estimates by Party Category for 2025*							
	No MAGA Affiliation							
	Non-MAGA, leans or not strong Republican (n = 1083)		Non-MAGA, Independent (n = 932)		Non-MAGA, leans or not strong Democrat (n = 1849)		Non-MAGA, strong Democrat (n = 1332)	
	Unweighted n	Weighted % (95% CI) *Mean score (95% CI)*	Unweighted n	Weighted % (95% CI) *Mean score (95% CI)*	Unweighted n	Weighted % (95% CI) *Mean score (95% CI)*	Unweighted n	Weighted % (95% CI) *Mean score (95% CI)*
Use force or violence as part of a group of people who share your beliefs								
Not asked the question†	213	24.3 (20.6, 27.9)	218	26.5 (22.7, 30.4)	434	24.3 (21.7, 26.9)	362	25.3 (22.4, 28.3)
Not willing (1)	729	64.5 (60.6, 68.4)	571	58.0 (53.7, 62.3)	1166	62.6 (59.7, 65.6)	801	60.4 (56.9, 63.9)
Somewhat willing (2)	122	9.5 (7.2, 11.7)	100	9.3 (7.0, 11.7)	208	9.7 (8.0, 11.4)	137	10.9 (8.5, 13.4)
Very or completely willing (3)	13	1.0 (0.3, 1.7)	29	4.0 (2.1, 6.0)	33	2.9 (1.7, 4.1)	22	2.6 (1.2, 4.0)
Non-response	6	0.8 (0.1, 1.5)	14	2.1 (0.7, 3.6)	8	0.5 (0.0, 0.9)	10	0.8 (0.2, 1.3)
Mean score‡	1077	*0.87 (0.82, 0.92)*	918	*0.91 (0.84, 0.97)*	1841	*0.91 (0.87, 0.95)*	1322	*0.91 (0.86, 0.96)*
aPD; q-value§		*-0.07 (-1.76, 1.63); 1.00*		*1.39 (-1.10, 3.88); 1.00*		*0.38 (-1.48, 2.24); 1.00*		*Reference*
Use force or violence on your own, as an individual								
Not asked the question†	213	24.3 (20.6, 27.9)	218	26.5 (22.7, 30.4)	434	24.3 (21.7, 26.9)	362	25.3 (22.4, 28.3)
Not willing (1)	692	61.8 (57.9, 65.8)	524	53.7 (49.4, 57.9)	1095	59.5 (56.5, 62.5)	785	60.0 (56.5, 63.4)
Somewhat willing (2)	144	10.6 (8.2, 13.0)	135	13.4 (10.5, 16.3)	269	12.9 (10.9, 15.0)	144	11.2 (8.8, 13.7)
Very or completely willing (3)	27	2.4 (1.1, 3.8)	41	4.4 (2.6, 6.2)	43	2.8 (1.8, 3.8)	33	2.8 (1.6, 4.1)
Non-response	7	0.9 (0.2, 1.7)	14	2.0 (0.6, 3.4)	8	0.5 (0.0, 0.9)	8	0.7 (0.1, 1.2)
Mean score‡	1076	*0.91 (0.86, 0.97)*	918	*0.96 (0.89, 1.02)*	1841	*0.94 (0.90, 0.98)*	1324	*0.92 (0.87, 0.97)*
aPD; q-value§		*1.14 (-0.78, 3.05); 1.00*		*1.56 (-0.85, 3.97); 1.00*		*0.36 (-1.31, 2.04); 1.00*		*Reference*
Organize a group of people who share your beliefs to use force or violence								
Not asked the question†	213	24.3 (20.6, 27.9)	218	26.5 (22.7, 30.4)	434	24.3 (21.7, 26.9)	362	25.3 (22.4, 28.3)
Not willing (1)	787	68.3 (64.4, 72.1)	602	60.0 (55.8, 64.3)	1283	66.5 (63.5, 69.4)	856	64.5 (61.1, 67.9)
Somewhat willing (2)	68	5.6 (3.8, 7.4)	68	7.6 (5.2, 9.9)	96	6.5 (4.8, 8.2)	81	6.3 (4.4, 8.1)
Very or completely willing (3)	9	1.0 (0.1, 2.0)	31	4.0 (2.2, 5.9)	28	2.2 (1.3, 3.2)	23	3.1 (1.5, 4.7)
Non-response	6	0.8 (0.1, 1.5)	13	1.8 (0.5, 3.2)	8	0.5 (0.0, 0.9)	10	0.8 (0.3, 1.4)
Mean score‡	1077	*0.83 (0.78, 0.88)*	919	*0.89 (0.83, 0.95)*	1841	*0.87 (0.83, 0.91)*	1322	*0.87 (0.82, 0.92)*
aPD; q-value§		*-0.96 (-2.94, 1.02); 1.00*		*0.26 (-2.23, 2.75); 1.00*		*-1.14 (-3.10, 0.81); 1.00*		*Reference*

* Among respondents to both the 2024 and 2025 surveys (n=7767). Prevalences for 2024 among respondents to that survey were reported previously: Wintemute GJ, Velasquez B, Robinson SL, Tomsich EA, Wright MA, Shev AB. The MAGA movement and political violence in 2024: findings from a nationally representative survey. Inj Epidemiol. 2025;12(1):78. Respondents who did not answer the question "In general...to advance an important political objective that you support" in 2024 (n = 58) or 2025 (n = 57) were not asked these questions.

† Respondents answered “never justified” to all prior questions on the use of force or violence to advance specific political objectives were not asked questions on their personal willingness to use political violence.

‡ Mean scores in 2024 and 2025 were scored as indicated in the response lines for individual questions, with non-responses excluded.

§ Adjusted prevalence differences (aPDs) are absolute percentage point (pp) differences for “very or completely willing” responses and are adjusted for age, race and ethnicity, gender, income, education, Census division, marital status, homeownership, rurality, firearm ownership, alcohol consumption, military service, and history of non-traffic arrest. Q-values, also known as FDR-adjusted (or FDR-corrected) p-values, represent the probability that the given difference would be a false discovery; they represent the expected proportion of “false positives” that would be seen among the collection of all differences whose q-values were at or below the given q-value. Item non-responses are not reported in the tables but are included in the prevalence calculations.

Table S17. Party/MAGA affiliation and 2024-2025 change in personal willingness to commit political violence, by social context of violence

**You agreed that the use of force or violence could be justified to advance (one/some) of the political objectives we just discussed. In (that/those) (situation/situations), how willing would you personally be to…**	**Mean Differences* by Party Category 2024-2025**							
	**MAGA Affiliation**						**No MAGA Affiliation**	
	**MAGA Republican (n = 1183)**		**MAGA Supporter, Republican (n = 565)**		**MAGA Supporter, Non-Republican (n = 197)**		**Non-MAGA; strong Republican (n = 417)**	
	**Unweighted n**	**Weighted mean (95% CI)** ***Mean score (95% CI)***	**Unweighted n**	**Weighted mean (95% CI)** ***Mean score (95% CI)***	**Unweighted n**	**Weighted mean (95% CI)** ***Mean score (95% CI)***	**Unweighted n**	**Weighted mean (95% CI)** ***Mean score (95% CI)***
Use force or violence as part of a group of people who share your beliefs								
Not asked the question†	1179	0.2 (-3.0, 3.4)	563	1.7 (-3.4, 6.9)	195	-8.3 (-20.0, 3.4)	415	-0.5 (-6.7, 5.7)
Not willing (1)	1179	2.8 (-2.3, 7.8)	563	1.1 (-5.6, 7.8)	195	5.7 (-8.1, 19.5)	415	1.7 (-5.5, 8.9)
Somewhat willing (2)	1179	-3.3 (-7.2, 0.6)	563	-2.3 (-7.8, 3.2)	195	-1.2 (-10.6, 8.1)	415	-2.9 (-6.9, 1.1)
Very or completely willing (3)	1179	0.1 (-1.5, 1.7)	563	-1.2 (-3.1, 0.6)	195	2.7 (-2.7, 8.1)	415	0.6 (-1.8, 2.9)
Non-response	1179	0.5 (-0.6, 1.5)	563	0.6 (-2.9, 4.1)	195	-1.8 (-5.0, 1.4)	415	1.6 (-0.3, 3.4)
Mean score‡	1163	*-0.037 (-0.098, 0.024)*	555	*-0.053 (-0.127, 0.022)*	190	*0.238 (0.023, 0.452)*	412	*-0.025 (-0.109, 0.059)*
Use force or violence on your own, as an individual								
Not asked the question†	1179	0.2 (-3.0, 3.4)	563	1.7 (-3.4, 6.9)	195	-8.3 (-20.0, 3.4)	415	-0.5 (-6.7, 5.7)
Not willing (1)	1179	0.0 (-4.7, 4.7)	563	0.3 (-6.3, 6.8)	195	10.1 (-1.9, 22.1)	415	3.3 (-3.8, 10.4)
Somewhat willing (2)	1179	0.1 (-3.4, 3.5)	563	0.2 (-5.0, 5.4)	195	0.9 (-8.6, 10.5)	415	-1.9 (-7.0, 3.2)
Very or completely willing (3)	1179	0.1 (-1.7, 1.9)	563	-1.5 (-4.2, 1.3)	195	-2.0 (-9.9, 5.9)	415	-3.6 (-7.0, -0.2)
Non-response	1179	0.5 (-0.6, 1.6)	563	-0.9 (-2.8, 1.0)	195	-1.8 (-5.0, 1.4)	415	0.6 (-0.6, 1.9)
Mean score‡	1164	*-0.024 (-0.085, 0.036)*	557	*-0.035 (-0.123, 0.054)*	190	*0.105 (-0.080, 0.290)*	414	*-0.026 (-0.129, 0.077)*
Organize a group of people who share your beliefs to use force or violence								
Not asked the question†	1179	0.2 (-3.0, 3.4)	563	1.7 (-3.4, 6.9)	195	-8.3 (-20.0, 3.4)	415	-0.5 (-6.7, 5.7)
Not willing (1)	1179	2.6 (-1.8, 7.0)	563	1.6 (-4.0, 7.2)	195	8.7 (-4.2, 21.6)	415	3.0 (-3.9, 9.9)
Somewhat willing (2)	1179	-1.8 (-4.9, 1.2)	563	-4.6 (-9.7, 0.5)	195	2.7 (-2.8, 8.3)	415	-3.2 (-7.8, 1.5)
Very or completely willing (3)	1179	-0.9 (-2.4, 0.6)	563	0.7 (-2.6, 3.9)	195	-2.0 (-9.1, 5.1)	415	-1.0 (-3.2, 1.3)
Non-response	1179	0.0 (-0.8, 0.8)	563	0.5 (-3.0, 4.1)	195	-1.8 (-5.0, 1.4)	415	0.6 (-0.6, 1.9)
Mean score‡	1166	*-0.039 (-0.092, 0.014)*	555	*-0.033 (-0.123, 0.058)*	190	*0.110 (-0.086, 0.306)*	414	*-0.018 (-0.110, 0.074)*

Table S17, continued.

You agreed that the use of force or violence could be justified to advance (one/some) of the political objectives we just discussed. In (that/those) (situation/situations), how willing would you personally be to…	Mean Differences* by Party Category 2024-2025							
	No MAGA Affiliation							
	Non-MAGA; leans or not strong Republican (n = 1083)		Non-MAGA; Independent (n = 932)		Non-MAGA; leans or not strong Democrat (n = 1849)		Non-MAGA; strong Democrat (n = 1332)	
	Unweighted n	Weighted mean (95% CI) *Mean score (95% CI)*	Unweighted n	Weighted mean (95% CI) *Mean score (95% CI)*	Unweighted n	Weighted mean (95% CI) *Mean score (95% CI)*	Unweighted n	Weighted mean (95% CI) *Mean score (95% CI)*
Use force or violence as part of a group of people who share your beliefs								
Not asked the question†	1078	0.0 (-4.3, 4.3)	920	-5.6 (-10.8, -0.4)	1845	-6.5 (-9.6, -3.4)	1326	-11.4 (-15.0, -7.8)
Not willing (1)	1078	-0.8 (-5.7, 4.1)	920	4.4 (-1.2, 10.0)	1845	5.9 (2.5, 9.4)	1326	8.8 (4.5, 13.2)
Somewhat willing (2)	1078	0.8 (-1.8, 3.4)	920	0.1 (-2.7, 3.0)	1845	-0.8 (-3.0, 1.4)	1326	2.4 (0.0, 4.9)
Very or completely willing (3)	1078	0.1 (-0.8, 1.0)	920	1.7 (-0.4, 3.8)	1845	1.1 (0.0, 2.2)	1326	-0.8 (-1.8, 0.2)
Non-response	1078	0.3 (-0.1, 0.8)	920	-0.3 (-1.4, 0.9)	1845	-0.2 (-0.6, 0.1)	1326	0.1 (-0.2, 0.5)
Mean score‡	1072	*0.000 (-0.063, 0.063)*	906	*0.078 (-0.004, 0.160)*	1837	*0.094 (0.044, 0.145)*	1316	*0.145 (0.093, 0.197)*
Use force or violence on your own, as an individual								
Not asked the question†	1078	0.0 (-4.3, 4.3)	920	-5.6 (-10.8, -0.4)	1845	-6.5 (-9.6, -3.4)	1326	-11.4 (-15.0, -7.8)
Not willing (1)	1078	-0.2 (-5.3, 5.0)	920	5.0 (-0.9, 10.9)	1845	4.2 (0.8, 7.6)	1326	7.0 (2.8, 11.3)
Somewhat willing (2)	1078	-0.3 (-3.6, 2.9)	920	0.2 (-3.3, 3.8)	1845	2.5 (0.4, 4.5)	1326	3.0 (0.4, 5.7)
Very or completely willing (3)	1078	-0.1 (-1.5, 1.2)	920	1.0 (-1.0, 3.1)	1845	-0.4 (-1.7, 1.0)	1326	0.3 (-0.8, 1.4)
Non-response	1078	0.2 (-0.4, 0.9)	920	-0.4 (-1.5, 0.7)	1845	-0.1 (-0.3, 0.2)	1326	-0.2 (-0.8, 0.4)
Mean score‡	1070	*0.008 (-0.061, 0.077)*	906	*0.065 (-0.015, 0.144)*	1837	*0.092 (0.041, 0.144)*	1317	*0.182 (0.131, 0.233)*
Organize a group of people who share your beliefs to use force or violence								
Not asked the question†	1078	0.0 (-4.3, 4.3)	920	-5.6 (-10.8, -0.4)	1845	-6.5 (-9.6, -3.4)	1326	-11.4 (-15.0, -7.8)
Not willing (1)	1078	-1.1 (-6.0, 3.8)	920	3.7 (-1.8, 9.3)	1845	5.2 (1.9, 8.5)	1326	7.8 (3.8, 11.8)
Somewhat willing (2)	1078	0.6 (-1.6, 2.8)	920	1.0 (-1.7, 3.6)	1845	0.4 (-1.6, 2.4)	1326	2.0 (0.3, 3.6)
Very or completely willing (3)	1078	0.1 (-1.1, 1.4)	920	0.6 (-1.4, 2.7)	1845	0.6 (-0.3, 1.5)	1326	0.3 (-1.1, 1.6)
Non-response	1078	0.4 (-0.1, 0.8)	920	-0.5 (-1.5, 0.5)	1845	0.0 (-0.3, 0.2)	1326	0.2 (-0.2, 0.6)
Mean score‡	1073	*0.011 (-0.045, 0.067)*	909	*0.100 (0.024, 0.177)*	1839	*0.093 (0.047, 0.138)*	1316	*0.173 (0.123, 0.222)*

* Among respondents to both the 2024 and 2025 surveys (n=7767). Respondents who did not answer the question "In general...to advance an important political objective that you support" in 2024 (n = 58) or 2025 (n = 57) were not asked these questions.

† Respondents answered “never justified” to all prior questions on the use of force or violence to advance specific political objectives were not asked questions on their personal willingness to use political violence.

‡ To assess population-level change from 2024 to 2025, we computed within-individual change scores for each item and then calculated year-to-year population-level change scores based on the means of aggregated within-individual change scores. Mean change scores have a range from -2 to 2 (with 0 indicating no change).

Table S18. Party/MAGA affiliation and 2025 prevalence of future firearm possession and use when political violence is perceived as justified

**Thinking now about the future and all the changes it might bring, how likely is it that you will use a gun in any of the following ways in the next few years—in a situation where you think force or violence is justified to advance an important political objective?**	**Population Estimates by Party Category for 2025***							
	**MAGA Affiliation**						**No MAGA Affiliation**	
	**MAGA Republican (n = 1183)**		**MAGA Supporter, Republican (n = 565)**		**MAGA Supporter, Non-Republican (n = 197)**		**Non-MAGA, strong Republican (n = 417)**	
	**Unweighted n**	**Weighted % (95% CI)** ***Mean score (95% CI)***	**Unweighted n**	**Weighted % (95% CI)** ***Mean score (95% CI)***	**Unweighted n**	**Weighted % (95% CI)** ***Mean score (95% CI)***	**Unweighted n**	**Weighted % (95% CI)** ***Mean score (95% CI)***
I will be armed with a gun								
Not likely	710	65.6 (62.1, 69.1)	344	62.9 (57.3, 68.6)	125	66.2 (56.7, 75.7)	285	67.8 (61.6, 74.0)
Somewhat likely	207	16.3 (13.6, 19.0)	107	19.3 (14.6, 24.0)	23	6.4 (2.9, 9.9)	69	18.6 (13.2, 23.9)
Very or extremely likely	252	16.9 (14.4, 19.4)	105	14.1 (10.8, 17.3)	43	22.3 (13.6, 31.0)	61	11.8 (8.0, 15.7)
Non-response	14	1.2 (0.4, 2.0)	9	3.7 (0.2, 7.2)	6	5.1 (0.0, 10.4)	2	1.8 (0.0, 4.3)
Mean score†	1169	*1.51 (1.45, 1.56)*	556	*1.49 (1.42, 1.57)*	191	*1.54 (1.36, 1.72)*	415	*1.43 (1.34, 1.52)*
aPD; q-value‡		*8.70 (5.70, 11.71); <0.0001*		*5.71 (1.98, 9.44); 0.19*		*12.97 (5.25, 20.70); 0.09*		*5.02 (0.92, 9.12); 0.67*
I will carry a gun openly, so that people know I am armed								
Not likely	909	77.9 (74.8, 81.0)	457	80.9 (76.0, 85.9)	151	73.0 (63.7, 82.4)	341	80.4 (75.0, 85.9)
Somewhat likely	147	12.8 (10.1, 15.4)	66	10.4 (6.8, 14.0)	15	5.7 (2.0, 9.3)	46	12.1 (7.5, 16.7)
Very or extremely likely	114	8.3 (6.5, 10.2)	33	5.0 (2.8, 7.1)	24	16.0 (7.7, 24.3)	26	5.1 (2.5, 7.6)
Non-response	13	1.0 (0.3, 1.7)	9	3.7 (0.2, 7.2)	7	5.3 (0.0, 10.6)	4	2.4 (0.0, 5.0)
Mean score†	1170	*1.30 (1.25, 1.34)*	556	*1.21 (1.16, 1.27)*	190	*1.40 (1.23, 1.57)*	413	*1.23 (1.16, 1.30)*
aPD; q-value‡		*5.28 (2.86, 7.70); 0.003*		*2.21 (-0.63, 5.05); 1.00*		*10.21 (2.5, 17.93); 0.43*		*2.86 (-0.12, 5.84); 1.00*
I will threaten someone with a gun								
Not likely	1130	94.4 (92.2, 96.6)	536	94.2 (91.1, 97.3)	173	82.7 (74.4, 91.0)	401	93.1 (89.0, 97.3)
Somewhat likely	25	2.6 (1.2, 3.9)	15	3.0 (0.6, 5.4)	5	3.9 (0.0, 8.8)	10	4.2 (0.9, 7.5)
Very or extremely likely	14	1.9 (0.3, 3.5)	6	0.7 (0.1, 1.3)	12	8.1 (2.7, 13.5)	3	0.6 (0.0, 1.4)
Non-response	14	1.2 (0.4, 1.9)	8	2.1 (0.2, 4.0)	7	5.3 (0.0, 10.6)	3	2.0 (0.0, 4.5)
Mean score†	1169	*1.06 (1.03, 1.10)*	557	*1.04 (1.02, 1.07)*	190	*1.21 (1.09, 1.33)*	414	*1.06 (1.02, 1.09)*
aPD; q-value‡		*1.22 (-0.78, 3.22); 1.00*		*-0.17 (-1.66, 1.32); 1.00*		*5.65 (-0.06, 11.35); 1.00*		*-0.02 (-1.46, 1.43); 1.00*
I will shoot someone with a gun								
Not likely	1091	92.5 (90.3, 94.8)	529	93.3 (90.1, 96.5)	168	83.9 (76.5, 91.3)	393	92.4 (88.3, 96.5)
Somewhat likely	59	4.5 (3.0, 6.1)	23	4.0 (1.5, 6.6)	12	5.1 (1.3, 8.8)	15	4.6 (1.4, 7.9)
Very or extremely likely	19	1.8 (0.3, 3.4)	6	0.7 (0.1, 1.3)	11	5.9 (1.4, 10.4)	5	0.8 (0.0, 1.5)
Non-response	14	1.1 (0.4, 1.8)	7	2.0 (0.0, 3.9)	6	5.1 (0.0, 10.4)	4	2.2 (0.0, 4.8)
Mean score†	1169	*1.08 (1.05, 1.12)*	558	*1.06 (1.03, 1.08)*	191	*1.18 (1.08, 1.28)*	413	*1.06 (1.03, 1.10)*
aPD; q-value‡		*1.16 (-0.88, 3.20); 1.00*		*-0.13 (-1.62, 1.36); 1.00*		*3.67 (-0.82, 8.15); 1.00*		*0.18 (-1.27, 1.64); 1.00*

Table S18, continued.

Thinking now about the future and all the changes it might bring, how likely is it that you will use a gun in any of the following ways in the next few years—in a situation where you think force or violence is justified to advance an important political objective?	**Population Estimates by Party Category for 2025***							
	**No MAGA Affiliation**							
	**Non-MAGA, leans or not strong Republican (n = 1083)**		**Non-MAGA, Independent (n = 932)**		**Non-MAGA, leans or not strong Democrat (n = 1849)**		**Non-MAGA, strong Democrat (n = 1332)**	
	**Unweighted n**	**Weighted % (95% CI)** ***Mean score (95% CI)***	**Unweighted n**	**Weighted % (95% CI)** ***M ean score (95% CI)***	**Unweighted n**	**Weighted % (95% CI)** ***Mean score (95% CI)***	**Unweighted n**	**Weighted % (95% CI)** ***Mean score (95% CI)***
I will be armed with a gun								
Not likely	789	77.2 (73.9, 80.5)	689	74.6 (70.7, 78.4)	1504	84.1 (81.9, 86.3)	1149	87.6 (85.3, 90.0)
Somewhat likely	157	13.1 (10.4, 15.8)	135	14.0 (10.9, 17.1)	215	9.8 (8.1, 11.6)	95	6.3 (4.7, 8.0)
Very or extremely likely	129	8.9 (6.8, 10.9)	89	8.5 (6.1, 10.9)	118	5.4 (3.9, 6.8)	84	5.6 (3.8, 7.4)
Non-response	8	0.8 (0.1, 1.5)	19	3.0 (1.3, 4.6)	12	0.7 (0.1, 1.3)	4	0.4 (0.0, 0.8)
Mean score†	1075	*1.31 (1.26, 1.36)*	913	*1.32 (1.26, 1.37)*	1837	*1.21 (1.17, 1.24)*	1328	*1.18 (1.14, 1.22)*
aPD; q-value‡		*2.99 (0.38, 5.60); 0.92*		*1.47 (-1.24, 4.18); 1.00*		*-0.35 (-2.53, 1.84); 1.00*		*Reference*
I will carry a gun openly, so that people know I am armed								
Not likely	946	88.2 (85.6, 90.7)	797	84.2 (80.9, 87.4)	1708	92.3 (90.5, 94.0)	1255	93.0 (90.8, 95.2)
Somewhat likely	87	7.1 (5.2, 9.0)	71	8.2 (5.8, 10.6)	81	4.2 (2.9, 5.4)	43	3.4 (1.9, 4.8)
Very or extremely likely	41	3.5 (2.0, 5.1)	44	4.5 (2.7, 6.2)	44	2.7 (1.5, 3.8)	27	3.0 (1.4, 4.6)
Non-response	9	1.2 (0.2, 2.1)	20	3.2 (1.5, 4.9)	16	0.9 (0.3, 1.5)	7	0.6 (0.1, 1.1)
Mean score†	1074	*1.14 (1.11, 1.18)*	912	*1.18 (1.13, 1.22)*	1833	*1.10 (1.07, 1.12)*	1325	*1.09 (1.06, 1.13)*
aPD; q-value‡		*1.46 (-0.70, 3.63); 1.00*		*1.49 (-0.85, 3.82); 1.00*		*0.28 (-1.60, 2.17); 1.00*		*Reference*
I will threaten someone with a gun								
Not likely	1055	96.3 (94.5, 98.2)	877	92.2 (89.6, 94.7)	1799	95.9 (94.5, 97.4)	1305	96.8 (95.3, 98.3)
Somewhat likely	12	1.7 (0.2, 3.2)	20	2.7 (1.1, 4.3)	21	2.0 (1.0, 3.1)	12	1.4 (0.3, 2.5)
Very or extremely likely	10	1.2 (0.2, 2.2)	14	1.9 (0.6, 3.1)	15	1.3 (0.5, 2.1)	8	1.3 (0.3, 2.3)
Non-response	6	0.7 (0.1, 1.4)	21	3.2 (1.5, 5.0)	14	0.8 (0.2, 1.4)	7	0.5 (0.1, 0.9)
Mean score†	1077	*1.04 (1.02, 1.07)*	911	*1.07 (1.04, 1.10)*	1835	*1.05 (1.03, 1.07)*	1325	*1.04 (1.02, 1.06)*
aPD; q-value‡		*0.64 (-1.01, 2.29); 1.00*		*0.31 (-1.43, 2.04); 1.00*		*0.09 (-1.34, 1.53); 1.00*		*Reference*
I will shoot someone with a gun								
Not likely	1034	94.8 (92.7, 96.8)	859	90.1 (87.3, 92.9)	1766	94.7 (93.2, 96.3)	1277	95.5 (93.9, 97.2)
Somewhat likely	31	3.1 (1.4, 4.8)	32	4.4 (2.4, 6.3)	47	2.3 (1.3, 3.2)	33	2.6 (1.3, 3.9)
Very or extremely likely	12	1.4 (0.4, 2.4)	21	2.3 (1.0, 3.5)	24	2.3 (1.1, 3.4)	15	1.4 (0.4, 2.3)
Non-response	6	0.7 (0.1, 1.4)	20	3.3 (1.5, 5.0)	12	0.7 (0.1, 1.3)	7	0.5 (0.1, 0.9)
Mean score†	1077	*1.06 (1.03, 1.09)*	912	*1.09 (1.06, 1.12)*	1837	*1.07 (1.04, 1.09)*	1325	*1.05 (1.03, 1.08)*
aPD; q-value‡		*0.78 (-0.90, 2.46); 1.00*		*0.69 (-1.04, 2.41); 1.00*		*0.88 (-0.76, 2.51); 1.00*		*Reference*

* Among respondents to both the 2024 and 2025 surveys (n=7767). Prevalences for 2024 among respondents to that survey were reported previously: Wintemute GJ, Velasquez B, Robinson SL, Tomsich EA, Wright MA, Shev AB. The MAGA movement and political violence in 2024: findings from a nationally representative survey. Inj Epidemiol. 2025;12(1):78.

† Mean scores in 2024 and 2025 were scored as indicated in the response lines for individual questions, with non-responses excluded.

‡ Adjusted prevalence differences (aPDs) are absolute percentage point (pp) differences for “very or extremely likely” responses and are adjusted for age, race and ethnicity, gender, income, education, Census division, marital status, homeownership, rurality, firearm ownership, alcohol consumption, military service, and history of non-traffic arrest. Q-values, also known as FDR-adjusted (or FDR-corrected) p-values, represent the probability that the given difference would be a false discovery; they represent the expected proportion of “false positives” that would be seen among the collection of all differences whose q-values were at or below the given q-value. Item non-responses are not reported in the tables but are included in the prevalence calculations.

Table S19. Party/MAGA affiliation and 2024-2025 change in future firearm possession and use when political violence is perceived as justified

Thinking now about the future and all the changes it might bring, how likely is it that you will use a gun in any of the following ways in the next few years—in a situation where you think force or violence is justified to advance an important political objective?	Mean Differences* by Party Category 2024-2025							
	MAGA Affiliation						No MAGA Affiliation	
	MAGA Republican (n = 1183)		MAGA Supporter, Republican (n = 565)		MAGA Supporter, Non-Republican (n = 197)		Non-MAGA; strong Republican (n = 417)	
	**Unweighted n**	**Weighted mean (95% CI)** ***Mean score (95% CI)***	**Unweighted n**	**Weighted mean (95% CI)** ***Mean score (95% CI)***	**Unweighted n**	**Weighted mean (95% CI)** ***Mean score (95% CI)***	**Unweighted n**	**Weighted mean (95% CI)** ***Mean score (95% CI)***
I will be armed with a gun								
Not likely	1183	5.9 (2.1, 9.7)	565	-2.5 (-7.6, 2.5)	197	4.4 (-3.9, 12.8)	417	-1.3 (-7.7, 5.1)
Somewhat likely	1183	-1.8 (-5.2, 1.6)	565	2.9 (-2.9, 8.6)	197	-10.4 (-19.8, -0.9)	417	6.1 (-0.1, 12.3)
Very likely	1183	-3.8 (-6.8, -0.9)	565	-1.5 (-4.2, 1.3)	197	0.9 (-5.3, 7.1)	417	-3.4 (-7.1, 0.2)
Extremely likely	1183	-0.7 (-1.9, 0.4)	565	0.6 (-3.3, 4.5)	197	2.6 (-2.0, 7.2)	417	0.3 (-1.4, 1.9)
Mean score†	1149	*-0.079 (-0.152, -0.007)*	548	*0.036 (-0.052, 0.123)*	188	*0.025 (-0.141, 0.192)*	413	*-0.060 (-0.182, 0.062)*
I will carry a gun openly, so that people know I am armed								
Not likely	1183	3.1 (-0.2, 6.3)	565	-0.6 (-5.9, 4.8)	197	-1.1 (-9.9, 7.7)	417	0.2 (-6.4, 6.8)
Somewhat likely	1183	-1.2 (-4.5, 2.0)	565	0.9 (-4.3, 6.1)	197	-3.6 (-9.1, 1.9)	417	0.3 (-4.8, 5.5)
Very likely	1183	-0.7 (-3.2, 1.8)	565	0.4 (-1.7, 2.5)	197	1.8 (-5.0, 8.7)	417	-0.2 (-2.4, 2.0)
Extremely likely	1183	-0.7 (-1.7, 0.3)	565	0.5 (-3.4, 4.4)	197	2.8 (-1.8, 7.4)	417	0.8 (-1.0, 2.6)
Mean score†	1151	*-0.039 (-0.094, 0.015)*	548	*-0.006 (-0.097, 0.086)*	187	*0.029 (-0.166, 0.225)*	411	*-0.032 (-0.168, 0.103)*
I will threaten someone with a gun								
Not likely	1183	1.3 (-0.6, 3.2)	565	-0.7 (-4.5, 3.1)	197	-5.1 (-11.7, 1.4)	417	0.2 (-4.4, 4.7)
Somewhat likely	1183	-0.4 (-1.7, 1.0)	565	2.0 (-0.6, 4.6)	197	0.9 (-4.7, 6.5)	417	1.6 (-1.5, 4.8)
Very likely	1183	-0.7 (-2.4, 1.0)	565	-0.4 (-1.9, 1.0)	197	-2.5 (-7.3, 2.3)	417	-1.9 (-4.6, 0.7)
Extremely likely	1183	-0.6 (-1.7, 0.5)	565	-0.8 (-3.2, 1.5)	197	3.3 (-1.2, 7.8)	417	0.5 (-1.2, 2.2)
Mean score†	1151	*-0.003 (-0.034, 0.027)*	550	*0.009 (-0.032, 0.051)*	188	*0.065 (-0.073, 0.204)*	412	*-0.034 (-0.100, 0.033)*
I will shoot someone with a gun								
Not likely	1183	1.1 (-1.1, 3.2)	565	-0.2 (-4.3, 3.8)	197	-1.4 (-10.6, 7.7)	417	1.2 (-3.5, 5.9)
Somewhat likely	1183	-0.1 (-1.8, 1.5)	565	1.5 (-1.5, 4.5)	197	0.6 (-5.3, 6.5)	417	0.7 (-2.6, 3.9)
Very likely	1183	-0.1 (-1.1, 0.9)	565	-0.4 (-1.8, 1.0)	197	-3.7 (-9.1, 1.6)	417	-1.0 (-3.4, 1.5)
Extremely likely	1183	-0.6 (-1.6, 0.5)	565	-0.7 (-3.1, 1.6)	197	3.1 (-1.4, 7.6)	417	0.1 (-2.0, 2.2)
Mean score†	1153	*-0.010 (-0.038, 0.018)*	552	*0.005 (-0.038, 0.048)*	189	*-0.027 (-0.190, 0.136)*	410	*-0.036 (-0.106, 0.035)*

Table S19, continued.

Thinking now about the future and all the changes it might bring, how likely is it that you will use a gun in any of the following ways in the next few years—in a situation where you think force or violence is justified to advance an important political objective?	Mean Differences* by Party Category 2024-2025							
	No MAGA Affiliation							
	Non-MAGA; leans or not strong Republican (n = 1083)		Non-MAGA; Independent (n = 932)		Non-MAGA; leans or not strong Democrat (n = 1849)		Non-MAGA; strong Democrat (n = 1332)	
	Unweighted n	Weighted mean (95% CI) *Mean score (95% CI)*	Unweighted n	Weighted mean (95% CI) *Mean score (95% CI)*	Unweighted n	Weighted mean (95% CI) *Mean score (95% CI)*	Unweighted n	Weighted mean (95% CI) *Mean score (95% CI)*
I will be armed with a gun								
Not likely	1083	-2.4 (-6.0, 1.3)	932	-2.1 (-5.8, 1.6)	1849	-2.4 (-4.9, 0.0)	1332	-0.9 (-3.9, 2.1)
Somewhat likely	1083	0.2 (-2.9, 3.4)	932	0.6 (-2.9, 4.0)	1849	1.0 (-1.2, 3.3)	1332	-0.5 (-3.0, 2.0)
Very likely	1083	1.8 (0.1, 3.6)	932	-0.4 (-3.0, 2.3)	1849	0.5 (-0.8, 1.7)	1332	1.5 (0.1, 3.0)
Extremely likely	1083	-0.5 (-1.5, 0.6)	932	0.9 (-0.5, 2.3)	1849	0.5 (-0.1, 1.0)	1332	-0.2 (-0.8, 0.4)
Mean score†	1067	*0.060 (0.003, 0.117)*	905	*0.026 (-0.042, 0.095)*	1834	*0.035 (-0.008, 0.077)*	1322	*0.018 (-0.033, 0.070)*
I will carry a gun openly, so that people know I am armed								
Not likely	1083	-1.3 (-4.1, 1.5)	932	-1.7 (-4.9, 1.4)	1849	-0.5 (-2.6, 1.5)	1332	-0.2 (-2.3, 2.0)
Somewhat likely	1083	0.5 (-1.7, 2.6)	932	2.1 (-0.2, 4.5)	1849	-0.2 (-2.0, 1.6)	1332	-0.5 (-2.4, 1.3)
Very likely	1083	0.7 (-0.8, 2.1)	932	-0.8 (-2.7, 1.1)	1849	0.6 (-0.6, 1.9)	1332	0.8 (-0.5, 2.1)
Extremely likely	1083	-0.1 (-1.3, 1.2)	932	1.0 (-0.3, 2.4)	1849	0.4 (-0.4, 1.2)	1332	0.0 (-0.7, 0.7)
Mean score†	1067	*0.024 (-0.020, 0.069)*	904	*-0.017 (-0.079, 0.046)*	1829	*0.002 (-0.032, 0.036)*	1318	*0.001 (-0.038, 0.040)*
I will threaten someone with a gun								
Not likely	1083	-1.6 (-3.6, 0.5)	932	-0.9 (-3.1, 1.3)	1849	-0.5 (-2.0, 1.1)	1332	0.4 (-1.4, 2.2)
Somewhat likely	1083	0.8 (-0.8, 2.5)	932	0.4 (-1.5, 2.3)	1849	-0.4 (-1.8, 1.1)	1332	-0.5 (-2.0, 0.9)
Very likely	1083	0.7 (-0.2, 1.6)	932	-0.1 (-1.8, 1.7)	1849	0.2 (-0.6, 1.0)	1332	-0.4 (-1.6, 0.9)
Extremely likely	1083	-0.2 (-1.1, 0.6)	932	1.2 (-0.1, 2.4)	1849	0.5 (-0.1, 1.0)	1332	-0.1 (-0.7, 0.6)
Mean score†	1070	*0.031 (0.004, 0.058)*	902	*-0.016 (-0.049, 0.017)*	1830	*0.005 (-0.014, 0.024)*	1318	*-0.003 (-0.032, 0.026)*
I will shoot someone with a gun								
Not likely	1083	-2.6 (-4.9, -0.2)	932	-1.2 (-3.9, 1.6)	1849	-0.6 (-2.3, 1.1)	1332	-0.1 (-2.1, 1.9)
Somewhat likely	1083	1.5 (-0.4, 3.5)	932	0.5 (-1.7, 2.6)	1849	-0.9 (-2.4, 0.5)	1332	0.4 (-1.5, 2.2)
Very likely	1083	0.9 (0.0, 1.8)	932	-1.0 (-2.4, 0.4)	1849	1.3 (0.2, 2.4)	1332	-0.2 (-1.5, 1.1)
Extremely likely	1083	-0.1 (-0.9, 0.8)	932	1.3 (0.1, 2.6)	1849	0.1 (-0.5, 0.8)	1332	-0.1 (-0.8, 0.5)
Mean score†	1072	*0.041 (0.011, 0.070)*	904	*-0.001 (-0.046, 0.043)*	1829	*0.021 (-0.003, 0.045)*	1318	*-0.006 (-0.042, 0.031)*

* Among respondents to both the 2024 and 2025 surveys (n=7767).

† To assess population-level change from 2024 to 2025, we computed within-individual change scores for each item and then calculated year-to-year population-level change scores based on the means of aggregated within-individual change scores. Mean change scores have a range from -2 to 2 (with 0 indicating no change).
